# Global, regional, and national burden of mental disorders among adolescents and young adults, 1990–2021: a systematic analysis for the Global Burden of Disease Study 2021

**DOI:** 10.1038/s41398-025-03623-w

**Published:** 2025-10-10

**Authors:** Zuxing Wang, Yikai Dou, Xiao Yang, Xiaoyun Guo, Xiaohong Ma, Bo Zhou, Wei Zhang

**Affiliations:** 1 Sichuan Provincial Center for Mental Health, Sichuan Provincial People’s Hospital, University of Electronic Science and Technology of China, Chengdu, China; 2 Mental Health Center and Institute of Psychiatry, West China Hospital, Sichuan University, Chengdu, P. R. China; 3 Shanghai Mental Health Center, Shanghai Jiao Tong University School of medicine, Shanghai, China; 4 Department of Psychiatry, Yale University School of Medicine, New Haven, CT USA; 5 West China Biomedical Big Data Center, West China Hospital of Sichuan University, Chengdu, China

**Keywords:** Psychiatric disorders, Scientific community

## Abstract

Mental disorders among adolescents and young adults (ages 10–24) are a significant public health challenge, contributing to long–term morbidity and substantial societal impact. This study analyzes the prevalence, incidence, and years lived with disability (YLDs) of mental disorders in this age group from 1990–2021, with particular attention to the effects of the COVID–19 pandemic during 2019–2021. Using data from the Global Burden of Disease Study 2021, we estimated the prevalence, incidence, and YLDs, along with age–standardized rates (ASR) and 95% uncertainty intervals (95% UIs) for 2021, stratified by sex and age group. Joinpoint regression was employed to calculate annual percentage change (APC) and average annual percentage change (AAPC), along with their corresponding 95% confidence intervals (CIs). In 2021, the global prevalence of mental disorders among adolescents and young adults was 278.98 million (95% UI: 248.61–312.81), with an ASR of 14,764.94 (95% UI: 2804.87–16,908.09). From 2019–2021, there were significant increases in the prevalence, incidence, and YLDs of mental disorders, especially for depressive and anxiety disorders. High–income regions, including North America, Western Europe, and the Asia Pacific, experienced the highest burdens. Globally, the prevalence of mental disorders was higher in males than females among adolescents aged 10–14, but higher in females among those aged 15–24. Joinpoint regression analysis from 1990–2021 revealed an increased burden in depressive disorders, anxiety disorders, bipolar disorder, eating disorders, autism spectrum disorder, conduct disorder, and idiopathic developmental intellectual disability, while decreases were observed in schizophrenia, ADHD, and other mental disorders. This study highlights the significant impact of COVID–19 on the mental health of adolescents and young adults, revealing disparities by region, age, sex, time period, and cohort. It underscores the need for targeted interventions to address the rising mental health burden in this group.

## Introduction

Mental disorders among adolescents and young adults have become a significant public health concern [1, 2]. Many mental disorders, including depression, anxiety, eating disorders, and psychosis, manifest during childhood and adolescence and often persist into adulthood, leading to long–term morbidity and substantial societal burden [3–5]. Therefore, a comprehensive understanding of the overall state of mental disorders during this critical developmental period—for both sexes, different age groups, and across various locations—is essential.

The COVID–19 pandemic has raised numerous concerns about its direct psychological impacts as well as the long–term economic and social consequences for mental health [6]. Direct impacts include significant health and mortality effects on those infected with the virus [7]. Beyond these direct effects, the pandemic has created an environment where many determinants of mental health are adversely affected [6]. Social restrictions, lockdowns, school and business closures, loss of livelihoods, reduced economic activity, and shifting government priorities in response to the pandemic all have the potential to significantly impact mental health [8–10]. Previous studies have shown that the COVID–19 pandemic significantly increased the prevalence and burden of depressive and anxiety disorders in 2020 [6], and there was also a notable rise in suicide rates during the early stages of the pandemic [11, 12]. This underscores the importance of studying the effects of COVID–19 specifically on mental disorders in adolescents and young adults, as this demographic may experience unique and profound impacts.

The Global Burden of Diseases, Injuries, and Risk Factors Study (GBD) 2021 offers comprehensive insights into the burden of various health conditions, including mental disorders, across diverse regions and countries [13]. Leveraging this latest data, our study delves into the prevalence, incidence, and years lived with disability (YLDs) of mental disorders among adolescents and young adults aged 10–24 years in 2021. We particularly focus on the percentage changes estimated from 2019–2021, a crucial period coinciding with the COVID–19 pandemic. Through the stratified analysis of data by geographic location, age group, and sex, our objective is to elucidate the nuanced trends in mental health among adolescents and young adults, facilitating the identification of critical areas for targeted intervention and support. This study aims to furnish contemporary evidence on the global burden of mental disorders in adolescents and young adults, while exploring the impact of COVID–19 on their mental health.

## Method

### Data source

GBD 2021 estimated incidence, prevalence, mortality, healthy life expectancy, years lived with disability (YLDs), years of life lost (YLLs), and disability–adjusted life years (DALYs) for 371 diseases and injuries across females and males, 25 age groups, 21 regions, 204 countries and territories, and 811 subnational locations [13]. The focus of GBD 2021 was on estimates from 2010–2021, highlighting trends over the past decade and the first two years of the COVID–19 pandemic [13]. In our study, we utilized and analyzed cross–sectional data from 1990–2021, specifically examining the incidence, prevalence, and YLDs of mental disorders in youth during the pandemic years of 2021, sourceNAd from the Global Health Data Exchange (https://ghdx.healthdata.org/gbd-resultstool). The data was categorized by gender, three age groups (10–14, 15–19, 20–24), region, and country, each accompanied by a 95% uncertainty interval (UI).

The mental disorders included in GBD 2021 were depressive disorders (major depressive disorder and dysthymia), anxiety disorders, bipolar disorder, attention–deficit/hyperactivity disorder (ADHD), schizophrenia, autism spectrum disorder (ASD), conduct disorder, idiopathic developmental intellectual disability (IDID), eating disorders (anorexia nervosa and bulimia nervosa), and a residual group of other mental disorders. To allow for comparability in measurement, case definitions predominantly adhered to ICD–10 or DSM–IV–TR criteria, since these were used by the majority of included mental health surveys. The mental disorders included in GBD 2021 and their definitions are further explained in the latest GBD survey.

### Estimation of the changes on incidence, prevalence, and YLDs during the COVID–19 pandemic

The GBD study team calculated the percentage changes in age–standardized rates per 100,000 population for self–harm and interpersonal violence attributable to high alcohol use during 2019–2021. These changes reflect the variation in these rates over the specified time period. The percentage changes were determined by calculating the difference in the natural logarithms of the values at the start and end of the time interval, divided by the number of years in the interval. This calculation was performed 500 times to generate draw–level estimates, with the final estimates derived as the mean across these draws. To ensure robust quantification of uncertainty, uncertainty intervals (UIs) were calculated as the 2.5th and 97.5th percentiles of the draw–level estimates, with uncertainty propagated at every stage of the estimation process [13].

### Joinpoint regression analysis

In our study, we deployed Joinpoint regression analysis—a robust statistical method (Joinpoint Trend Analysis Software. Version 4.9.1.0) widely utilized in epidemiology to delineate temporal trends in disease prevalence or mortality [14, 15]. This approach excels in detecting and quantifying critical inflection points within time–series data. Utilizing this model, we computed the Annual Percent Change (APC) along with its 95% Confidence Interval (CI), enabling us to map prevalence trends over distinct time intervals. To encapsulate the overall trend, we also derived the Average Annual Percent Change (AAPC), summarizing the trend data from 1990–2021. Statistically, an APC or AAPC estimate with a 95% CI lower bound above zero signifies an increasing trend, whereas a 95% CI upper bound below zero indicates a decreasing trend. Stability is implied when the 95% CI for the APC or AAPC includes zero.

### Statistical analysis

The prevalence, incidence, and YLDs were downloaded in terms of both number and age–standardized rate (ASR), along with percentage changes from 2019–2021, inclusive of their 95% UI. The ASR was calculated per 100,000 people using the following formula, based on the world standard population provided in the GBD 2021 [16]:

$${\rm{ASR}}=\frac{{\sum }_{i=1}^{A}{a}_{i}\,{w}_{i}}{{\sum }_{i=1}^{A}\,{w}_{i}}\times 100,000,$$

– where *ai* is the age–specific rate in the *i*th age group, *w* is the number of people in the corresponding *i*th age group among the standard population and *A* is the number of age groups. All analytical procedures and graphical representations were conducted using the World Health Organization’s Health Equity Assessment Toolkit and the statistical computing software R (Version 3.5.2). To ensure methodological rigor and transparency, our study design and reporting align with the Guidelines for Accurate and Transparent Health Estimates Reporting (GATHER) checklist principles [17] (Supplementary Table S1).

## Results

### Global level

As shown in Table 1, globally in 2021, the estimated prevalent cases of mental disorders among young people were 278.98 million (95% UI: 248.61–312.81). The corresponding ASR was 14,764.94 (95% UI: 2,804.87–16,908.09). In terms of incidence, the global incident cases of mental disorders in 2021 were 108.21 million (95% UI: 87.93–132.55), with a ASR of 5718.91 (95% UI: 4334.63–7360.22). Mental disorders accounted for 36.31 million (95% UI: 26.59–48.11) YLDs in 2021, equating to a ASR of 1919.04 (95% UI: 1370.36–2563.95). Globally, individuals aged 10–24 accounted for 25.47% of the prevalence, 24.35% of the incidence, and 23.37% of the YLDs of all mental disorders across all age groups (Supplementary Fig. 1). Additionally, the ranking of YLDs numbers for each mental disorder among all Level 3 causes (173 diseases) within the GBD for the years 1990, 2019, and 2021 in adolescents and young adults. In 2021, the rankings were as follows: anxiety disorders (2nd), depressive disorders (3rd), conduct disorder (7th), ASD (11th), bipolar disorder (23rd), eating disorders (26th), schizophrenia (28th), IDID (31st), other mental disorders (43rd), and ADHD (53rd) (Supplementary Fig. 2).

**Table 1:** The prevalence, incidence and years lived with disability for mental disorders of 10–24 years adolescents and young adults in 2021 and average annual percentage change from 1990–2021 and percentage rate change from 2019–2021 by GBD.

	Prevalence (95% UI)				Incidence (95% UI)				Years lived with disability (95% UI)			
	Counts in million	ASRs per 100,000 people	AAPC^a^ 1990–2021	PC in ASR, 2019–2021	Counts in million	ASRs per 100,000 people	AAPC^a^ 1990–2021	PC in ASR, 2019–2021	Counts in million	ASRs per 100,000 people	AAPC^a^ 1990–2021	PC in ASR, 2019–2021
**Mental disorders**												
Total	278.98 (248.61–312.81)	14,764.94 (12,804.87–16,908.09)	35.12 (29.88–40.35)	0.10 (0.08–0.12)	108.21 (87.93–132.55)	5718.91 (4334.63–7360.22)	38.69 (33.86–43.52)	0.22 (0.20–0.25)	36.31 (26.59–48.11)	1919.04 (1370.36–2563.95)	8.64 (7.67–9.60)	0.13 (0.11–0.16)
Female	138.78 (122.75–157.14)	15,043.21 (12,939.18–17,388.82)	53.07 (46.50–59.64)	0.13 (0.11–0.14)	61.66 (49.37–76.29)	6673.59 (5024.79–8686.00)	45.41 (39.31–51.51)	0.24 (0.22–0.26)	19.29 (13.99–25.66)	2086.88 (1472.10–2808.23)	10.64 (9.44–11.85)	0.15 (0.14–0.18)
Male	140.20 (248.61–312.81)	14,490.42 (12,612.80–16489.74)	17.98 (13.95–22.02)	0.07 (0.06–0.09)	46.55 (38.04–56.73)	4807.09 (3670.35–6124.89)	32.90 (29.20–36.60)	0.20 (0.17–0.22)	17.02 (12.53–22.37)	1758.16 (1265.43–2324.43)	6.80 (6.05–7.55)	0.11 (0.09–0.13)
**Schizophrenia**												
Total	2.00 (1.33–2.78)	104.40 (66.95–154.19)	−0.13 (−0.13–−0.12)	−0.01 (−0.02–0.00)	0.48 (0.34–0.66)	25.36 (15.21–37.90)	−0.03 (−0.03–−0.03)	−0.01 (−0.04–0.02)	1.33 (0.84–2.00)	69.74 (43.52–107.93)	−0.09 (−0.09–−0.08)	−0.01 (−0.04–0.01)
Female	0.92 (0.61–1.28)	97.55 (62.38–144.95)	−0.15 (−0.16–−0.14)	−0.01 (−0.02–0.00)	0.22 (0.16–0.30)	23.83 (14.18–35.77)	−0.03 (−0.03–−0.03)	−0.01 (−0.02–0.01)	0.60 (0.38–0.91)	64.43 (39.82–100.02)	−0.10 (−0.11–−0.09)	−0.02 (−0.05–0.01)
Male	1.08 (0.73–1.51)	110.99 (71.26–163.03)	−0.13 (−0.13–−0.12)	−0.01 (−0.04–0.02)	0.26 (0.18–0.36)	26.83 (16.17–39.94)	−0.03 (−0.03–−0.03)	−0.01 (−0.02–0.00)	0.73 (0.46–1.09)	74.85 (46.53–116.37)	−0.08 (−0.09–0.08)	−0.01 (−0.04–0.02)
**Depressive disorders**												
Total	57.49 (44.13–73.89)	3026.95 (2222.69–4111.09)	20.42 (17.73–23.11)	0.22 (0.18–0.25)	73.80 (53.64–97.62)	3891.39 (2707.49–5475.69)	30.28 (26.01–34.55)	0.28 (0.26–0.31)	10.72 (6.79–15.63)	564.48 (351.19–853.02)	3.94 (3.58–4.31)	0.25 (0.22–0.28)
Female	34.76 (26.76–44.37)	3744.25 (2753.10–5082.42)	24.30 (20.92–27.68)	0.22 (0.19–0.25)	44.86 (32.87–58.71)	4843.55 (3379.82–6830.68)	35.90 (30.49–41.31)	0.28 (0.26–0.31)	6.47 (4.11–9.40)	697.32 (435.06–1050.03)	4.93 (4.46–5.39)	0.25 (0.22–0.28)
Male	22.73 (17.37–29.39)	2341.69 (1708.60–3171.48)	17.89 (14.94–20.84)	0.21 (0.18–0.24)	28.94 (20.85–38.49)	2983.02 (2060.60–4198.26)	25.54 (22.28–28.81)	0.28 (0.26–0.31)	4.25 (2.70–6.26)	437.63 (270.44–663.61)	3.03 (2.76–3.31)	0.25 (0.22–0.28)
**Bipolar disorder**												
Total	7.81 (5.84–10.60)	410.80 (297.43–567.12)	0.63 (0.60–0.65)	−0.00 (−0.01–−0.00)	1.2 (0.89–1.64)	64.15 (42.20–93.32)	0.09 (0.089–0.09)	0.00 (−0.00–0.00)	1.74 (1.10–2.68)	91.31 (55.86–141.39)	0.14 (0.13–0.14)	−0.01 (−0.02–0.00)
Female	3.97 (2.96–5.41)	427.03 (307.94–591.78)	0.58 (0.56–0.61)	−0.01 (−0.02–0.01)	0.61 (0.45–0.83)	66.51 (43.66–96.94)	0.08 (0.07–0.08)	0.00 (−0.00–0.00)	0.88 (0.56–1.36)	94.13 (57.14–146.23)	0.13 (0.129–0.13)	−0.00 (−0.019–−0.00)
Male	3.84 (2.889–5.20)	395.28 (287.749–543.41)	0.67 (0.659–0.69)	−0.00 (−0.009–−0.00)	0.60 (0.449–0.81)	61.91 (40.849–89.78)	0.10 (0.109–0.11)	0.00 (−0.009–0.00)	0.86 (0.549–1.32)	88.62 (54.239–137.48)	0.15 (0.159–0.16)	−0.01 (−0.029–0.01)
**Anxiety disorders**												
Total	93.95 (71.909–120.28)	4967.97 (3639.389–6612.29)	30.87 (27.199–34.56)	0.24 (0.219–0.27)	16.67 (12.029–22.03)	883.50 (586.299–1225.42)	6.28 (5.689–6.89)	0.25 (0.239–0.28)	11.52 (7.279–16.91)	609.44 (377.509–909.05)	2.30 (2.009–2.61)	0.24 (0.219–0.27)
Female	57.33 (43.769–73.15)	6212.87 (4550.239–8239.97)	39.48 (34.839–44.14)	0.24 (0.229–0.27)	9.78 (7.019–13.10)	1063.51 (704.339–1480.72)	7.61 (6.869–8.36)	0.26 (0.239–0.29)	7.00 (4.419–10.25)	758.32 (470.089–1124.26)	2.97 (2.599–3.36)	0.24 (0.219–0.27)
Male	36.62 (28.149–46.81)	3781.58 (2751.039–5067.86)	23.20 (20.419–25.99)	0.23 (0.209–0.26)	6.89 (4.989–9.05)	712.31 (473.539–982.35)	5.10 (4.629–5.58)	0.25 (0.229–0.27)	4.52 (2.849–6.68)	467.59 (287.159–702.17)	1.78 (1.569–2.00)	0.23 (0.209–0.25)
**Eating disorders**												
Total	6.74 (4.649–9.78)	354.66 (223.159–548.83)	1.74 (1.739–1.76)	−0.00 (−0.019–0.00)	7.19 (4.559–10.86)	429.38 (217.649–772.58)	1.78 (1.759–1.82)	0.00 (−0.009–0.01)	1.44 (0.819–2.32)	75.83 (40.759–129.04)	0.37 (0.379–0.38)	−0.01 (−0.029–0.01)
Female	4.31 (3.029–6.11)	463.08 (298.139–696.31)	2.10 (2.079–2.13)	−0.01 (−0.029–−0.00)	3.03 (2.009–4.33)	327.94 (189.589–536.93)	1.51 (1.469–1.56)	−0.00 (−0.019–0.01)	0.92 (0.529–1.48)	98.48 (54.259–163.63)	0.44 (0.449–0.45)	−0.01 (−0.029–0.00)
Male	2.44 (1.609–3.73)	250.78 (148.469–417.31)	1.44 (1.429–1.45)	0.00 (−0.019–0.01)	4.16 (4.989–2.53)	379.84 (204.539–658.39)	1.97 (1.939–2.00)	0.01 (−0.009–0.02)	0.53 (0.289–0.86)	54.12 (27.219–97.89)	0.31 (0.319–0.32)	0.00 (−0.029–0.03)
**Autism spectrum disorders**												
Total	15.69 (18.419–13.23)	831.31 (701.069–975.56)	0.81 (0.799–0.84)	0.00 (−0.009–0.01)	NA	NA	NA	NA	3.00 (2.029–4.21)	158.47 (107.039–223.13)	0.05 (0.059–0.05)	0.00 (−0.019–0.01)
Female	5.03 (4.229–5.98)	547.03 (458.279–649.72)	0.88 (0.859–0.90)	0.00 (−0.009–0.01)	NA	NA	NA	NA	0.95 (0.659–1.34)	103.49 (70.859–145.90)	0.04 (0.049–0.04)	0.00 (−0.019–0.02)
Male	10.66 (9.009–12.45)	1101.70 (930.359–1287.22)	0.59 (0.579–0.60)	0.00 (−0.019–0.00)	NA	NA	NA	NA	2.04 (1.389–2.88)	210.77 (142.979–297.28)	0.05 (0.059–0.05)	−0.00 (−0.019–0.01)
**Attention**−**deficit/hyperactivity disorder**												
Total	41.03 (28.469–57.98)	2179.52 (1495.94–3085.11)	−10.23 (−10.629–−9.97)	0.00 (−0.029–0.02)	0.22 (0.159–0.32)	12.07 (8.209–17.38)	−0.03 (−0.039–−0.03)	0.00 (−0.029–0.03)	0.50 (0.269–0.82)	26.64 (13.879–44.84)	−0.19 (−0.209–−0.19)	0.00 (−0.039–0.02)
Female	11.30 (7.849–16.02)	1232.90 (838.299–1755.38)	−6.07 (−6.159–−5.98)	−0.00 (−0.039–0.02)	0.06 (0.049–0.09)	6.75 (4.549–9.83)	−0.02 (−0.029–−0.02)	0.01 (−0.009–0.02)	0.14 (0.079–0.23)	15.00 (7.869–25.48)	−0.17 (−0.189–−0.16)	−0.01 (−0.019–0.02)
Male	29.72 (20.759–41.93)	3077.44 (2118.649–4358.16)	−14.97 (−15.449–−14.49)	0.00 (−0.029–0.03)	0.16 (0.119–0.24)	17.06 (11.629–24.58)	−0.05 (−0.059–−0.05)	0.00 (−0.039–0.04)	0.36 (0.199–0.60)	37.68 (19.569–63.15)	−0.23 (−0.249–−0.22)	0.00 (−0.029–0.03)
**Conduct disorder**												
Total	33.72 (23.739–44.00)	1802.89 (1225.709–2486.00)	1.73 (1.689–1.78)	0.01 (0.009–0.01)	8.62 (5.589–11.72)	462.60 (276.849–658.37)	0.39 (0.379–0.40)	0.01 (0.019–0.01)	4.10 (2.189–6.58)	219.42 (114.699–356.37)	3.35 (3.209–3.50)	0.00 (0.009–0.01)
Female	11.96 (8.019–16.23)	1318.43 (850.449–1894.47)	2.00 (1.969–2.04)	0.01 (0.019–0.01)	3.08 (1.959–4.50)	341.49 (194.999–521.21)	0.45 (0.439–0.47)	0.01 (0.019–0.01)	1.45 (0.759–2.35)	159.48 (81.339–268.27)	4.03 (3.549–4.53)	0.00 (−0.019–0.02)
Male	21.76 (15.579–27.94)	2260.18 (1560.949–3068.04)	1.20 (1.149–1.25)	0.00 (0.009–0.01)	5.54 (3.709–7.47)	576.58 (343.929–810.30)	0.27 (0.259–0.28)	0.01 (0.009–0.01)	2.65 (1.459–4.18)	275.99 (145.729–444.20)	2.51 (2.239–2.79)	0.00 (−0.019–0.01)
**Idiopathic developmental intellectual disability**												
Total	29.78 (16.809–42.34)	1578.19 (890.299–2246.92)	−6.23 (−6.419–−6.06)	−0.01 (−0.049–0.02)	NA	NA	NA	NA	1.27 (0.599–2.15)	67.30 (31.479–113.81)	0.14 (0.129–0.15)	0.00 (−0.029–0.01)
Female	14.89 (8.919–20.68)	1619.37 (968.929–2250.61)	−4.37 (−4.509–−4.23)	0.00 (−0.039–0.02)	NA	NA	NA	NA	0.63 (0.329–1.04)	68.45 (34.409–113.24)	0.13 (0.119–0.15)	0.00 (−0.029–0.01)
Male	14.88 (7.889–21.66)	1538.97 (815.079–2241.72)	−8.26 (−8.779–−7.76)	−0.01 (−0.049–0.02)	NA	NA	NA	NA	0.64 (0.289–1.10)	66.20 (28.799–114.63)	0.14 (0.129–0.16)	0.00 (−0.029–0.02)
**Other mental disorders**												
Total	9.04 (5.819–12.92)	473.90 (304.939–675.40)	0.09 (0.099–0.10)	−0.01 (−0.019–−0.01)	NA	NA	NA	NA	0.69 (0.409–1.10)	36.4 (20.849–57.76)	−1.61 (−1.619–−1.60)	−0.01 (−0.039–0.01)
Female	3.41 (2.189–4.88)	364.83 (231.769–519.65)	0.08 (0.079–0.08)	−0.01 (−0.019–−0.01)	NA	NA	NA	NA	0.26 (0.149–0.41)	27.77 (15.169–44.27)	−1.06 (−1.069–−1.05)	−0.01 (−0.049–0.02)
Male	5.63 (3.649–8.09)	578.71 (374.539–826.98)	0.06 (0.059–0.07)	−0.01 (−0.019–0.00)	NA	NA	NA	NA	0.43 (0.269–0.69)	44.70 (26.209–70.68)	−2.12 (−2.129–−2.12)	−0.01 (−0.039–0.01)

*AAPC* average annual percentage change, *YLDs* years lived with disability, *UI* uncertainty interval, *ASR* age-standardized rate, *PC* percentage change.

^a^AAPC is expressed as 95% confidence intervals.

**Fig. 1:**
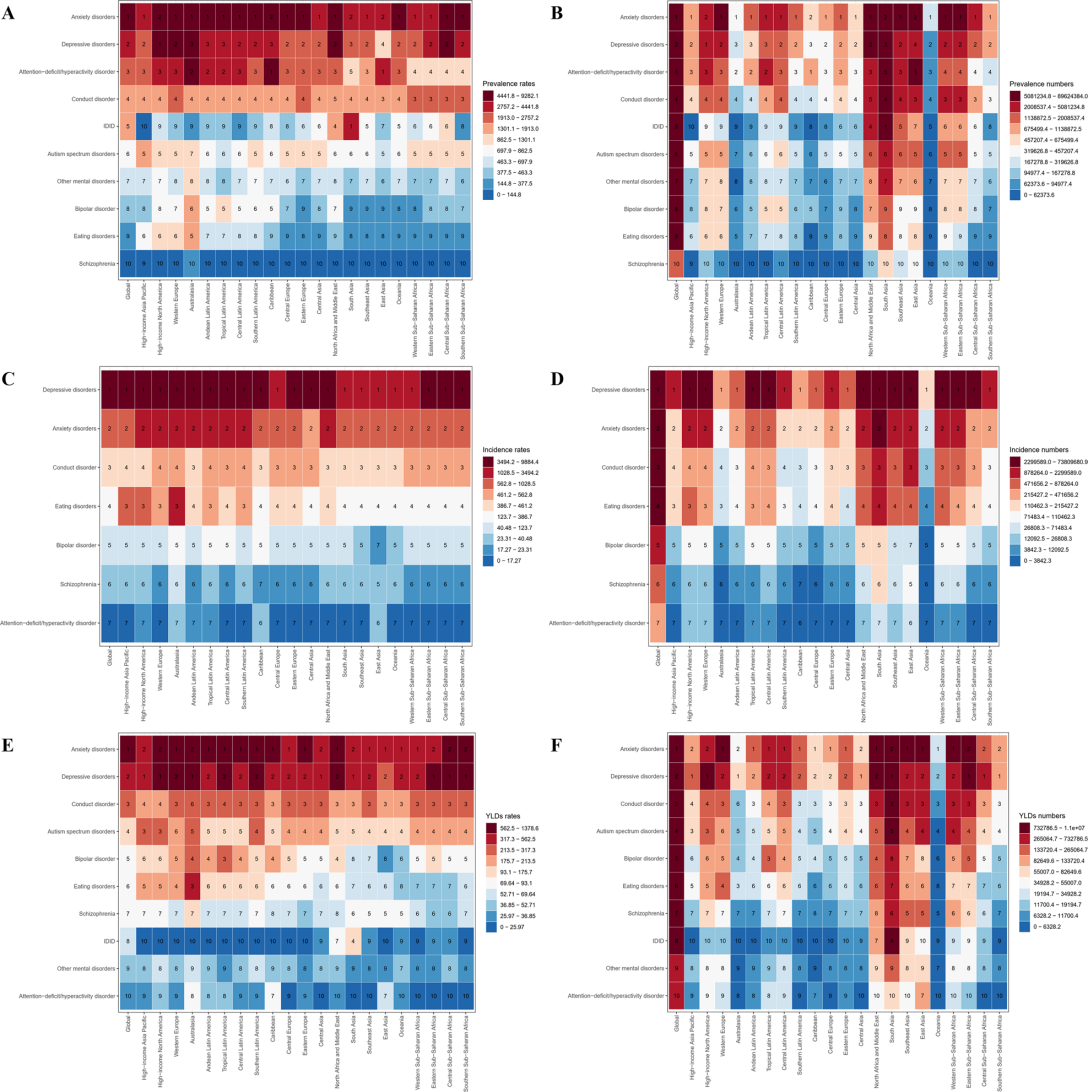
Global and regional rankings of prevalence, incidence, and years lived with disability (YLDs) numbers and age–standardized rates for different mental disorders among adolescents and young adults (10–24 years) in 2021. **A** Age–standardized prevalence rates; **B** Prevalence numbers; **C** Age–standardized incidence rates; **D** Incidence numbers; **E** Age–standardized YLDs rates; **F** YLDs numbers. IDID idiopathic developmental intellectual disability.

As presented in Table 1 and Fig. 1, the top three mental disorders by prevalence among youth globally in 2021 were anxiety disorders (93.95 million; 95% UI: 71.90–120.28), depressive disorders (57.49 million; 95% UI: 44.13–73.89), and ADHD (41.03 million; 95% UI: 28.46–57.98) (Fig. 1A). The corresponding age–standardized prevalence rate (ASPR) per 100000 population were 4967.97 (95% UI: 3639.38–6612.29), 3026.95 (95% UI: 2222.69–4111.09), and 2179.52 (95% UI: 1495.94–3085.11) (Fig. 1B). In terms of incidence, the top three were depressive disorders (73.80 million; 95% UI: 53.64–97.62), anxiety disorders (16.67 million; 95% UI: 12.02–22.03), and conduct disorder (8.62 million; 95% UI: 5.58–11.72) (Fig. 1C), with corresponding age–standardized incidence rate (ASIR) per 100000 population of 3891.39 (95% UI: 2707.49–5475.69), 883.50 (95% UI: 586.29–1225.42), and 462.60 (95% UI: 276.84–658.37) (Fig. 1D). For YLDs, the top three disorders were anxiety disorders (11.52 million; 95% UI: 7.27––16.91), depressive disorders (10.72 million; 95% UI: 6.79–15.63), and conduct disorder (4.10 million; 95% UI: 2.18–6.58) (Fig. 1E), with corresponding ASR of 609.44 (95% UI: 377.50–909.05), 564.48 (95% UI: 351.19–853.02), and 219.42 (95% UI: 114.69–356.37) (Fig. 1F).

As presented in Table 1, and Supplementary Fig. S3–8, the global percentage rate changes in prevalence, incidence, and YLDs for mental disorders among youth from 2019–2021 increased by 0.10% (95% UI: 0.08–0.12), 0.22% (95% UI: 0.20–0.25), and 0.13% (95% UI: 0.11–0.16), respectively. Specifically, depressive disorders and anxiety disorders showed the most significant increases. In addition, globally, the prevalence, incidence, and YLDs of conduct disorder also showed a slight increase from 2019–2021. Schizophrenia, bipolar disorder, eating disorders, and ADHD, did not exhibit significant percentage rate changes in prevalence, incidence, and YLDs from 2019–2021.

### Regional level

As shown in Fig. 1 and Supplementary Tables S2–22, For the 21 regions included in the analysis, in 2021, the ASPR of mental disorders among youth ranked anxiety disorders, depressive disorders, and ADHD as the top three in most regions, except for Southeast Asia, where IDID ranked first, and four regions in sub–Saharan Africa and East Asia, where conduct disorder ranked third (Fig. 1A). In terms of ASIR, depressive disorders and anxiety disorders were the top two in most regions, with conduct disorder ranking third except in High–income Asia Pacific, High–income North America, Western Europe, Australasia, Tropical Latin America, and Southern Latin America, where eating disorders ranked third (Fig. 1C). For age–standardized YLDs rate per 100000 population, anxiety disorders and depressive disorders were the top two in all regions except for Australasia, where eating disorders ranked third, and Tropical Latin America, where bipolar disorder ranked third (Fig. 1E). The distribution of the number of prevalent cases, incident cases, and YLDs of various mental disorders across regions followed the same pattern as their corresponding rates (Fig. 1B, D, F).

As depicted in Supplementary Tables S2–22 and Fig. S3–8, from 2019 and 2021, the prevalence, anxiety and YLDs of anxiety disorders and depressive disorders increased significantly in nearly all regions. Notably, Andean Latin America and Central Latin America experienced a sharp increase in 2020, followed by a slight decline in 2021. Although the rising trend in most regions slowed down in 2021, the prevalence of these disorders continued to increase. Supplementary Fig. S9 illustrate the ASPR of anxiety disorders and depressive disorders from 2019–2021. The distribution of age–standardized prevalence, incidence, and YLDs rates of different mental disorders across the 21 regions in 2021 is shown in Supplementary Fig. S10.

### National level

**Fig. 2:**
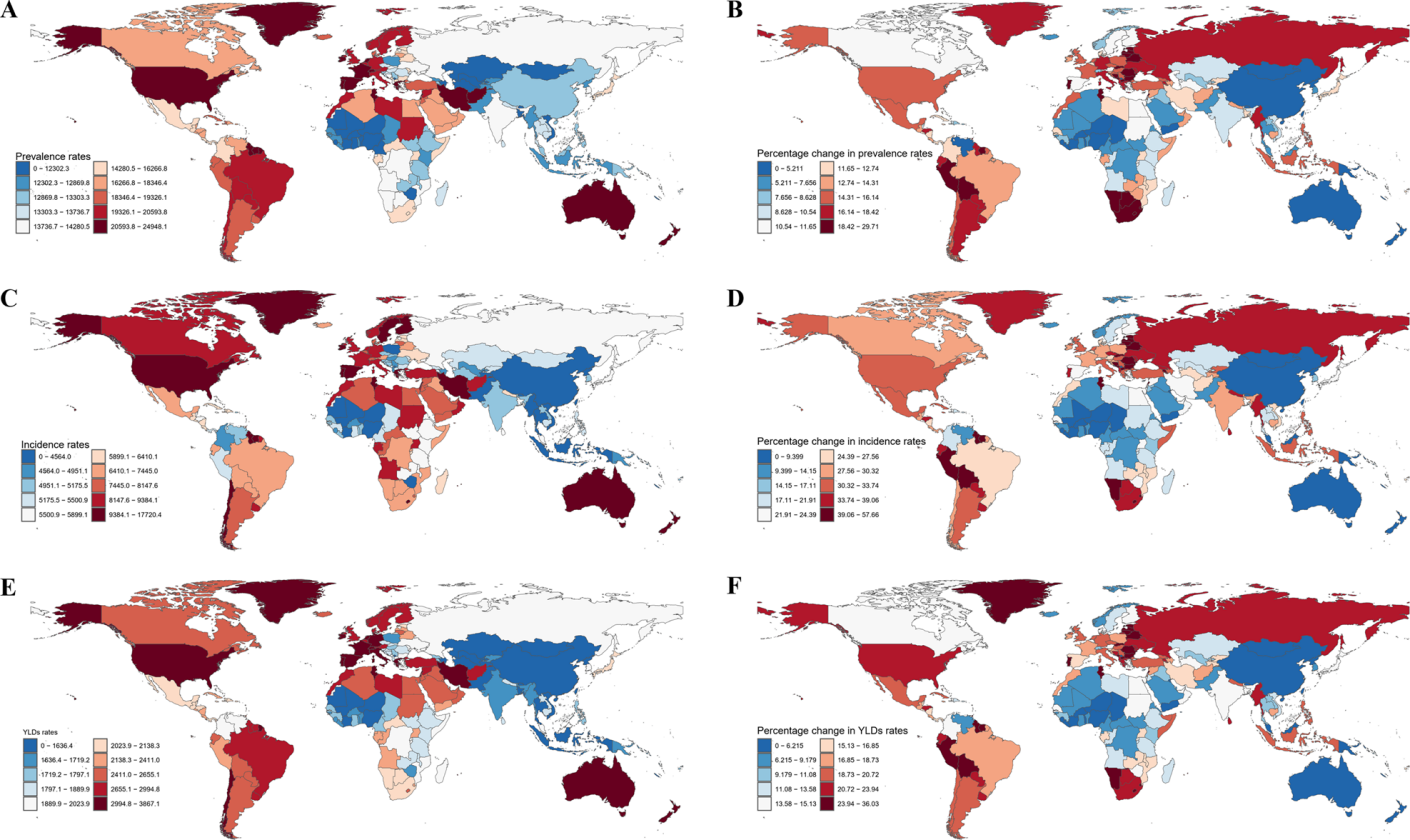
Prevalence, incidence, and years lived with disability (YLDs) rates of mental disorders among individuals aged 10–24 in 204 countries and territories in 2021, along with percentage changes from 2019–2021. **A** Age–standardized prevalence rates; **B** Percentage change in prevalence rates; **C** Age–standardized incidence rates; **D** Percentage change in incidence rates; **E** Age–standardized YLDs rates; **F** Percentage change in YLDs rates.

In 2021, among adolescents and young adults aged 10–24 in 204 countries and territories, the ASPR of mental disorders ranged from approximately 10,407.54 (95% UI: 8790.78–12,327.46) per 100,000 individuals in Vietnam to 24,836.21 (95% UI: 19,975.85–30,611.01) per 100,000 individuals in Portugal (Fig. 2A and Supplementary Table S23). From 2019–2021, the percentage change of mental disorders significantly increased in most countries. Notably, Eswatini (0.30; 95% UI: 0.17–0.43), Belarus (0.29; 95% UI: 0.16–0.44), and Bulgaria (0.28; 95% UI: 0.16–0.43) showed the largest increases (Fig. 2B and Supplementary Table S23).

For incidence, Greenland (17,425.25; 95% UI: 12,045.83–24,524.45), the United States of America (12,770.84; 95% UI: 10,107.28–15,920.96), and Palestine (11,627.35; 95% UI: 7,925.94–16,866.59), exhibited the highest ASIR per 100,000 persons (Fig. 2C and Supplementary Table S23). Conversely, China (2916.95; 95% UI: 2286.09–3592.72), the Democratic People’s Republic of Korea (3051.90; 95% UI: 2280.58–3990.09), and Taiwan (Province of China) (3101.71; 95% UI: 2300.40–4040.82) showed the lowest ASIR per 100,000 persons (Fig. 2C and Supplementary Table S23). From 2019–2021, the percentage change of this population increased in most countries and territories. Specifically, Bulgaria (0.58; 95% UI: 0.35–0.84), Belarus (0.57; 95% UI: 0.32–0.87), and Eswatini (0.57; 95% UI: 0.32–0.85) experienced the most substantial increases (Fig. 2D and Supplementary Table S23).

The highest age–standardized YLDs rates per 100,000 population in 2021 were observed in Greenland (3790.60; 95% UI: 2612.22–5383.60), Portugal (3645.25; 95% UI: 2445.73–5069.82), and Lebanon (3453.12; 95% UI: 2325.73–4894.83) (Fig. 2E and Supplementary Table S23). Conversely, the lowest age–standardized YLDs rates were observed in China (1371.22; 95% UI: 974.24–1823.16)), Vietnam (1375.55; 95% UI: 956.93–1881.73), and Bhutan (1379.17; 95% UI: 955.64–1892.75) (Fig. 2E and Supplementary Table S23). From 2019–2021, the countries with the largest increases in YLDs rates were Belarus (0.36; 95% UI: 0.21–0.55), Eswatini (0.36; 95% UI: 0.20–0.55), and Bulgaria (0.35; 95% UI: 0.21–0.54) (Fig. 2F and Supplementary Table S23). The numbers and percentage changes from 2019–2021 for prevalence, incidence, and YLDs are presented in Supplementary Fig. S11.

### Age and sex patterns

**Fig. 3:**
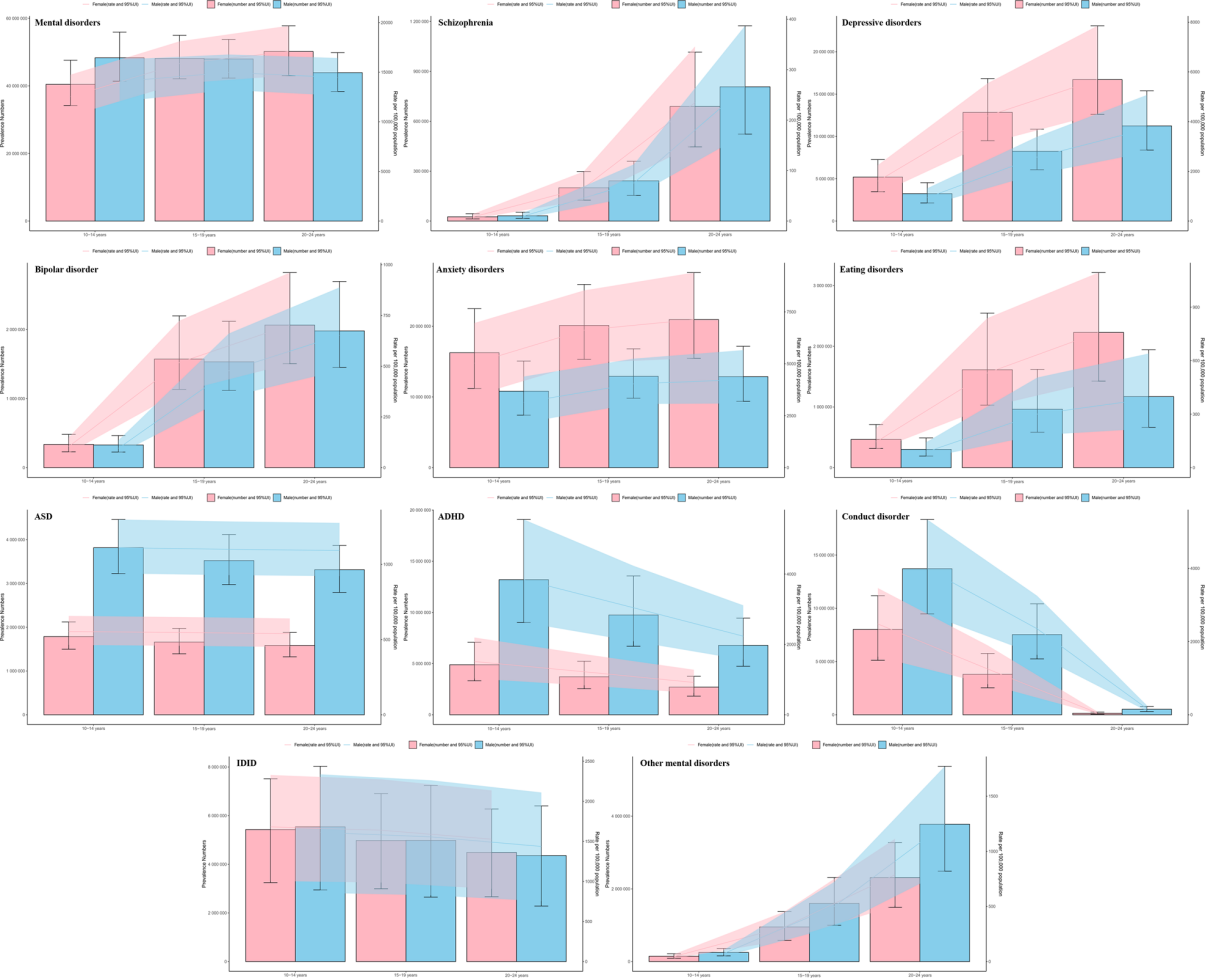
Global prevalence of mental disorders by sex and age group (10–24 years) in 2021. Lines indicate prevalent case with 95% uncertainty intervals for men and women.

As shown in Fig. 3 and Supplementary Tables S24–26, in 2021, globally, the prevalence of mental disorders among adolescents aged 10–14 was higher in males than in females. However, among those aged 15–19 and 20–24, the prevalence was higher in females. Specifically, for schizophrenia, ASD, ADHD, conduct disorder, and other mental disorders, males had a higher prevalence across all three age groups. Conversely, females had higher prevalence rates of depressive disorders, bipolar disorder, anxiety disorders, and eating disorders in all age groups. The prevalence of IDID was comparable between males and females across all ages. Schizophrenia, depressive disorders, bipolar disorder, anxiety disorders, eating disorders, and other mental disorders showed an increasing prevalence. In contrast, ADHD and conduct disorder exhibited a decreasing trend, while ASD and IDID remained relatively stable.

Regarding the incidence of mental disorders in the global 10–24 age group, both males and females showed an upward trend with age. This trend was consistent for depressive disorders and schizophrenia but opposite for anxiety disorders, ADHD, and conduct disorder. The incidence of bipolar disorder and eating disorders first increased and then decreased. Overall, in 2021, the age–specific and gender–specific distribution of the incidence of various mental disorders was similar to that of their prevalence. Interestingly, unlike prevalence, the incidence of eating disorders was higher in males than in females (Supplementary Fig. S12). For YLDs, the distribution and trends of various mental disorders across different age groups and genders mirrored those of their prevalence (Supplementary Fig. S13). The temporal trends of total mental disorders from 1990–2021 across different age groups are depicted in Supplementary Fig. S14. Additionally, the sex disparities in total mental disorders across the 21 regions in 2021 are illustrated in Supplementary Fig. S15.

### Temporal Joinpoint analysis

**Fig. 4:**
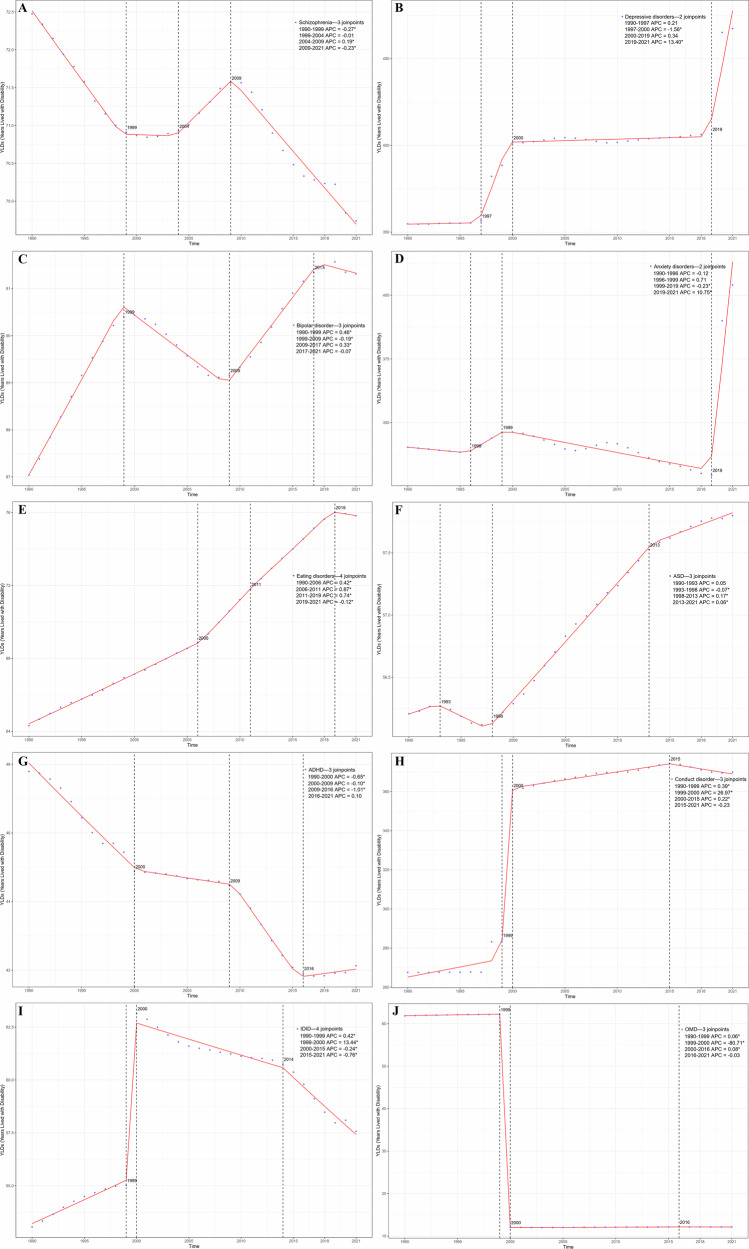
Joinpoint regression analysis in the years age–standardized lived with disability (YLDs) rate of mental disorders among individuals aged 10–24 (1990–2021). **A** Schizophrenia; **B** Depressive disorders; **C** Bipolar disorder; **D** Anxiety disorders; **E** Eating disorders; **F** Autism spectrum disorders (ASD); **G** Attention–deficit/hyperactivity disorder (ADHD); **H** Conduct disorder; **I** Idiopathic developmental intellectual disability (IDID); **J** Other mental disorders (OMD).

As shown in Table 1 and Fig. 4, Joinpoint regression analysis revealed significant trends in the age–standardized YLD rates for various mental disorders among the 10–24 age group between 1990 and 2021. There were significant increases in depressive disorders (AAPC = 3.94; 95% CI: 3.58–4.31), anxiety disorders (AAPC = 2.30; 95% CI: 2.00–2.61), bipolar disorder (AAPC = 0.14; 95% CI: 0.13–0.14), eating disorders (AAPC = 0.37; 95% CI: 0.37–0.38), ASD (AAPC = 0.05; 95% CI: 0.05–0.05), conduct disorder (AAPC = 3.35; 95% CI: 3.20–3.50), and IDID (AAPC = 0.14; 95% CI: 0.12–0.15). Conversely, there were significant decreases in schizophrenia (AAPC = −0.09; 95% CI: −0.09–−0.08), ADHD (AAPC = −0.19; 95% CI: −0.20–−0.19), and other mental disorders (AAPC = −1.61; 95% CI: −1.61–−1.60).

Notably, the age–standardized YLDs rates for depressive disorders showed significant increases during 1997–2000 (APC = 4.39; 95% CI: 2.68–6.12) and 2019–2021 (APC = 10.48; 95% CI: 7.62–13.41). The age–standardized YLD rates for anxiety disorders significantly increased during 2019–2021 (APC = 10.75; 95% CI: 8.93–12.60) but significantly decreased during 1999–2019 (APC = −0.23; 95% CI: −0.33–−0.13). For schizophrenia, significant decreases were observed during 1990–1999 (APC = −0.27; 95% CI: −0.30–−0.24) and 2009–2021 (APC = −0.23; 95% CI: −0.25–−0.21), while a significant increase was noted during 2004–2009 (APC = 0.19; 95% CI: 0.13–0.25). Bipolar disorder showed significant increases during 1990–1999 (APC = 0.46; 95% CI: 0.44–0.49) and 2009–2017 (APC = 0.33; 95% CI: 0.30–0.35), with a significant decrease observed during 1999–2009 (APC = −0.19; 95% CI: −0.21–−0.17). Eating disorders showed significant increases during 1990–2006 (APC = 0.42; 95% CI: 0.41–0.42), 2006–2011 (APC = 0.87; 95% CI: 0.83–0.92), and 2011–2019 (APC = 0.74; 95% CI: 0.71–0.76), with a significant decrease during 2019–2021 (APC = −0.12; 95% CI: −0.22–−0.02). For ASD, significant increases were observed during 1998–2013 (APC = 0.17; 95% CI: 0.16–0.17) and 2013–2021 (APC = 0.06; 95% CI: 0.04–0.07), with a significant decrease during 1993–1998 (APC = −0.07; 95% CI: −0.10–−0.05). ADHD showed significant decreases during 1990–2000 (APC = −0.65; 95% CI: −0.70–−0.61), 2000–2009 (APC = −0.10; 95% CI: −0.16–−0.04), and 2009–2016 (APC = −1.01; 95% CI: −1.12–−0.89). Conduct disorder showed significant increases during 1990–1999 (APC = 0.39; 95% CI: 0.15–0.62), 1999–2000 (APC = 27.00; 95% CI: 23.78–30.24), and 2000–2015 (APC = 0.22; 95% CI: 0.10–0.34). IDID showed significant increases during 1990–1999 (APC = 0.42; 95% CI: 0.32–0.52) and 1999–2000 (APC = 13.44; 95% CI: 12.23–14.67), with significant decreases during 2000–2015 (APC = 0.24; 95% CI: −0.30–−0.19) and 2015–2021 (APC = −0.76; 95% CI: −0.88–−0.65). The APC of ASPR and ASIR for various mental disorders among this age group are shown in Supplementary Fig. S16–17.

## Discussion

This study leverages the comprehensive dataset provided by the GBD 2021 to assess the global and regional burden of mental disorders among adolescents and young adults aged 10–24. The findings highlight the significant prevalence, incidence, and disability burden of mental disorders in this demographic, with notable increases observed during the COVID–19 pandemic.

In 2021, globally, individuals aged 10–24 accounted for 25.47% of the prevalence, 24.35% of the incidence, and 23.37% of the YLDs of all mental disorders across all age groups. UNESCO has declared the COVID–19 pandemic as the most severe disruption to global education in history, leading to a significant number of students dropping out of school. School closures and broader social restrictions have impeded young people’s ability to congregate in physical spaces, adversely affecting their learning and peer interactions. Additionally, during and following economic crises, young people are more likely than older individuals to face unemployment [18]. The higher prevalence compared to incidence and YLDs suggests that many young individuals are living with mental disorders over extended periods, reflecting the chronic nature of these conditions.

Consistent with previous studies, globally, anxiety disorders, depressive disorders, and ADHD were the most prevalent mental disorders among this population [19–21]. Anxiety disorders had the highest prevalence (93.95 million), depressive disorders followed (57.49 million), and ADHD (41.03 million) ranked third. Despite the high prevalence, the incidence data showed a different pattern; depressive disorders had the highest number of new cases (73.80 million), followed by anxiety disorders (16.67 million), and conduct disorder (8.62 million). This indicates a significant number of new depressive disorder cases emerged during the pandemic period, likely attributed to concerns over personal health, limited access to medical resources, overwhelming amounts of pandemic–related information, social isolation, and economic uncertainty [22].

The Joinpoint regression analysis showed an overall increase in the burden of most mental disorders, with a noticeable acceleration between 2019 and 2021. This trend was particularly pronounced for depressive and anxiety disorders, reflecting the significant mental health impact of the COVID–19 pandemic. The stress, uncertainty, and sense of loss experienced during the pandemic—whether from the death of loved ones, loss of employment, disruption of daily routines, or social connections—have been linked to increased levels of anxiety and depression [10, 23, 24]. The interruption of daily life and the enforced isolation due to lockdown measures have further exacerbated these conditions, particularly among adolescents and young adults during their crucial developmental stages. Moreover, previous studies have shown that anxiety and depression disorders increase the risk of suicide [25, 26], highlighting the profound impact of this period on mental health.

The regional analysis uncovers significant disparities in the burden of mental disorders. For example, Southeast Asia’s high prevalence of IDID suggests unique regional challenges that may be related to socio–cultural, economic, or healthcare factors [27]. Similarly, sub–Saharan Africa and East Asia show higher prevalence of conduct disorder. In many regions of sub–Saharan Africa, adolescents are exposed to significant environmental stressors, such as political instability, conflict, and high levels of violence. Exposure to violence and trauma is a well–documented risk factor for the development of conduct disorder [28, 29]. In East Asia, cultural attitudes towards behavior and discipline can influence the prevalence of conduct disorder [30]. In some cultures, strict or harsh disciplinary practices may be more common, potentially leading to higher levels of behavioral issues [31]. Additionally, cultural norms surrounding mental health can impact the recognition and reporting of conduct disorder symptoms [31]. The highest ASPR observed in countries like Portugal and the lowest in Vietnam suggest significant national differences potentially driven by healthcare infrastructure, cultural attitudes towards mental health, and socioeconomic conditions [32, 33]. Mental health education in schools, such as the United Kingdom’s Personal, Social, Health, and Economic Education (PSHE), which integrates mental health into school curricula, which provides free training for school staff to identify and support students with mental health issues, can help address these disparities [34]. Additionally, The Netherlands’ Youth Mental Health Strategy, which focuses on early intervention through programs that offer counseling and therapy in secondary schools, provides a comprehensive model for supporting adolescents’ mental health [35].The COVID–19 pandemic has had a profound impact on the mental health of adolescents and young adults, as evidenced by the significant increases in prevalence, incidence, and YLDs from 2019–2021 [6, 16, 36, 37]. The pandemic’s disruption of daily life, education, social interactions, and economic stability has likely exacerbated mental health issues, particularly depressive and anxiety disorders [6, 38]. Notably, while anxiety disorders remain the most prevalent mental disorder, the incidence of new depressive disorder cases surpassed that of anxiety disorders in all regions during the pandemic. This trend underscores the pandemic’s severe impact on mood and emotional well–being. In addition to depression and anxiety, conduct disorder also saw a significant rise during the pandemic. These disorders are closely linked, as social isolation, disrupted routines, and economic strain exacerbated both behavioral issues and emotional distress [39]. Adolescents struggled to manage emotions and behaviors due to a lack of structure and social support, with conduct disorder emerging alongside mood disorders. Shared factors—social isolation, uncertainty, and economic instability—heightened stress, leading to impulsivity and mood disorders, while limited access to mental health services further worsened these issues [39]. These findings align with global reports of increased mental health issues during the pandemic [40–42]. The sharp increases observed in regions such as Andean Latin America and Central Latin America highlight the varying impacts of the pandemic across different regions, potentially due to differences in pandemic severity, healthcare response, and social support systems. The persistent increase in mental health issues even after the peak of the pandemic [43], and the varying mental health challenges faced by different groups such as the general public [38], individuals who have or had COVID–19 [44], people with pre–existing mental health disorders [45], and healthcare workers [46], underscore the need for long–term mental health support and targeted recovery strategies [37].

The age and sex–specific analysis reveals important trends in the prevalence and incidence of mental disorders. Males aged 10–14 have higher prevalence rates, particularly for disorders like ADHD and conduct disorder, whereas females aged 15–24 exhibit higher rates of depressive and anxiety disorders. This gender disparity in mental health profiles underscores the need for gender–sensitive approaches in mental health interventions. For younger males, school–based interventions like the “Incredible Years Program” could focus on behavioral management, social skills, and parent–teacher training to address ADHD and conduct disorder. Expanding these successful programs globally could help manage disruptive behaviors [47]. For young females, mental health services should target body image, self–esteem, and use strategies like mindfulness and cognitive–behavioral therapy [48]. These interventions would ensure appropriate support for both genders, enhancing overall well–being. The increasing prevalence with age for disorders such as anxiety and depressive disorders suggests that mental health issues may become more pronounced as individuals transition from adolescence to young adulthood. Conversely, the decreasing trend in ADHD and conduct disorder prevalence with age indicates that these conditions may be more prevalent in younger adolescents. The stable prevalence of ASD and IDID across ages highlights the persistent nature of these disorders [49, 50]. Additionally, it is noteworthy that while the incidence of eating disorders is higher in males aged 10–24, the overall prevalence is significantly higher in females, indicating a longer duration or higher persistence of these disorders in females. This gender difference in eating disorders warrants further investigation to understand the underlying factors and inform targeted interventions. Understanding these age and sex patterns is crucial for developing age–appropriate and gender–specific mental health interventions and support systems.

Several limitations must be acknowledged. First, despite incorporating extensive new data, the quality of epidemiological data on mental disorders remains uneven, with many countries lacking high–quality surveys [51]. Second, the reliance on secondary data sources may introduce biases and limit the precision of our estimates. Additionally, while the GBD 2021 study includes post–traumatic stress disorder (PTSD) within the broader category of anxiety disorders, it does not separately categorize acute stress disorder and PTSD. This approach may not fully capture the distinct impact and complexities of these specific stress–related disorders, particularly in the context of a global health crisis like the COVID–19 pandemic [13]. Finally, broader GBD study limitations include incomplete representation of societal impact and the assumption of independent distributions for comorbid conditions, which might not capture the full complexity of mental disorders [52]. These factors highlight the need for ongoing improvements in data collection and methodological approaches.

## Conclusion

This study underscores the growing global burden of mental disorders among adolescents and young adults, particularly for depressive and anxiety disorders. The COVID–19 pandemic further exacerbated these issues from 2019–2021. This study underscores the profound impact of the COVID–19 pandemic on the mental health of adolescents and young adults, highlighting regional and national disparities as well as variations by age, sex, time period, and cohort. These findings emphasize the need for targeted interventions to address the growing burden of mental disorders in this age group.

## Supplementary information

Supplemental material

## Data Availability

Data used in the analyses can be obtained from the Global Health Data Exchange Global Burden of Disease Results Tool (https://ghdx.healthdata.org/gbd-results-tool).
