# Supplementary material for: Global, regional, and national burden of mental disorders among adolescents and young adults, 1990–2021: a systematic analysis for the Global Burden of Disease Study 2021
Source: Transl Psychiatry. 2025 Oct 10;15:397. doi: 10.1038/s41398-025-03623-w (PMC12514266; doi:10.1038/s41398-025-03623-w)

**Global, regional, and national burden of mental disorders among adolescents and young adults, 1990–2021: a systematic analysis for the Global Burden of Disease Study 2021**

**Supplementary table and figure legends**

**Tables**

**Table S2.** Prevalence, Incidence, and Years Lived with Disability of Mental Disorders Among Adolescents and Young Adults (Aged 10-24) in **Andean Latin America** in 2021, and Percentage Change from 2019 to 2021 According to GBD.

**Table S3.** Prevalence, Incidence, and Years Lived with Disability of Mental Disorders Among Adolescents and Young Adults (Aged 10-24) in **Australasia** in 2021, and Percentage Change from 2019 to 2021 According to GBD.

**Table S4.** Prevalence, Incidence, and Years Lived with Disability of Mental Disorders Among Adolescents and Young Adults (Aged 10-24) in **Caribbean** in 2021, and Percentage Change from 2019 to 2021 According to GBD.

**Table S5.** The Prevalence, Incidence, and Years Lived with Disability of Mental Disorders Among Adolescents and Young Adults (Aged 10-24) in **Central Asia** in 2021, and Percentage Change from 2019 to 2021 According to GBD.

**Table S6.** The Prevalence, Incidence, and Years Lived with Disability of Mental Disorders Among Adolescents and Young Adults (Aged 10-24) in **Central Europe** in 2021, and Percentage Change from 2019 to 2021 According to GBD.

**Table S7.** The Prevalence, Incidence, and Years Lived with Disability of Mental Disorders Among Adolescents and Young Adults (Aged 10-24) in **Central Latin America** in 2021, and Percentage Change from 2019 to 2021 According to GBD.

**Table S8.** The Prevalence, Incidence, and Years Lived with Disability of Mental Disorders Among Adolescents and Young Adults (Aged 10-24) in **Central Sub−Saharan Africa** in 2021, and Percentage Change from 2019 to 2021 According to GBD.

**Table S9.** The Prevalence, Incidence, and Years Lived with Disability of Mental Disorders Among Adolescents and Young Adults (Aged 10-24) in **East Asia** in 2021, and Percentage Change from 2019 to 2021 According to GBD.

**Table S10.** The Prevalence, Incidence, and Years Lived with Disability of Mental Disorders Among Adolescents and Young Adults (Aged 10-24) in **Eastern Europe** in 2021, and Percentage Change from 2019 to 2021 According to GBD.

**Table S11.** The Prevalence, Incidence, and Years Lived with Disability of Mental Disorders Among Adolescents and Young Adults (Aged 10-24) in **Eastern Sub−Saharan Africa** in 2021, and Percentage Change from 2019 to 2021 According to GBD.

**Table S12.** The Prevalence, Incidence, and Years Lived with Disability of Mental Disorders Among Adolescents and Young Adults (Aged 10-24) in **High-income Asia Pacific** in 2021, and Percentage Change from 2019 to 2021 According to GBD.

**Table S13.** The Prevalence, Incidence, and Years Lived with Disability of Mental Disorders Among Adolescents and Young Adults (Aged 10-24) in **High-income North America** in 2021, and Percentage Change from 2019 to 2021 According to GBD.

**Table S14.** The Prevalence, Incidence, and Years Lived with Disability of Mental Disorders Among Adolescents and Young Adults (Aged 10-24) in **North Africa and Middle East** in 2021, and Percentage Change from 2019 to 2021 According to GBD.

**Table S15.** The Prevalence, Incidence, and Years Lived with Disability of Mental Disorders Among Adolescents and Young Adults (Aged 10-24) in **Oceania** in 2021, and Percentage Change from 2019 to 2021 According to GBD.

**Table S16.** The Prevalence, Incidence, and Years Lived with Disability of Mental Disorders Among Adolescents and Young Adults (Aged 10-24) in **South Asia** in 2021, and Percentage Change from 2019 to 2021 According to GBD.

**Table S17.** The Prevalence, Incidence, and Years Lived with Disability of Mental Disorders Among Adolescents and Young Adults (Aged 10-24) in **Southeast Asia** in 2021, and Percentage Change from 2019 to 2021 According to GBD.

**Table S18.** The Prevalence, Incidence, and Years Lived with Disability of Mental Disorders Among Adolescents and Young Adults (Aged 10-24) in **Southern Latin America** in 2021, and Percentage Change from 2019 to 2021 According to GBD.

**Table S19.** The Prevalence, Incidence, and Years Lived with Disability of Mental Disorders Among Adolescents and Young Adults (Aged 10-24) in **Southern Sub−Saharan Africa** in 2021, and Percentage Change from 2019 to 2021 According to GBD.

**Table S20.** The Prevalence, Incidence, and Years Lived with Disability of Mental Disorders Among Adolescents and Young Adults (Aged 10-24) in **Tropical Latin America** in 2021, and Percentage Change from 2019 to 2021 According to GBD.

**Table S21.** The Prevalence, Incidence, and Years Lived with Disability of Mental Disorders Among Adolescents and Young Adults (Aged 10-24) in **Western Europe** in 2021, and Percentage Change from 2019 to 2021 According to GBD.

**Table S22.** The Prevalence, Incidence, and Years Lived with Disability of Mental Disorders Among Adolescents and Young Adults (Aged 10-24) in **Western Sub−Saharan Africa** in 2021, and Percentage Change from 2019 to 2021 According to GBD.

**Table S23**. Prevalence, Incidence, and Years Lived with Disability of Mental Disorders Among Adolescents and Young Adults (Aged 10-24) in **204 Countries and Territories** in 2021, and Percentage Change from 2019 to 2021 By GBD.

**Table S24.** Prevalence, Incidence, and Years Lived with Disability of Mental Disorders Among Adolescents and Young Adults (**Aged 10-14**) in 2021, and Percentage Change from 2019 to 2021 According to GBD.

**Table S25.** Prevalence, Incidence, and Years Lived with Disability of Mental Disorders Among Adolescents and Young Adults (**Aged 15-19**) in 2021, and Percentage Change from 2019 to 2021 According to GBD.

**Table S26.** Prevalence, Incidence, and Years Lived with Disability of Mental Disorders Among Adolescents and Young Adults (**Aged 20-24**) in 2021, and Percentage Change from 2019 to 2021 According to GBD.

**Figures**

**Figure S1**. Prevalence, incidence and years lived with disability (YLDs) of mental disorders by age and sex in 2021. (A) Prevalence; (B) Incidence; (C) YLDs.

**Figure S2**. Rankings of YLDs numbers for mental disorders by 10-24 age group for both sexes combined, 1990, 2019, and 2021.

**Figure S3**. Global and regional trends in the **prevalence numbers** of mental disorders among individuals aged 10-24 (1990-2021).

**Figure S4**. Global and regional trends in the **age-standardized prevalence rates** of mental disorders among individuals aged 10-24 (1990-2021).

**Figure S5**. Global and regional trends in the **incidence numbers** of mental disorders f among individuals aged 10-24 (1990-2021).

**Figure S6**. Global and regional trends in the **age-standardized incidence rates** of mental disorders among individuals aged 10-24 (1990-2021).

**Figure S7**. Global and regional trends in the **years lived with disability (YLDs) numbers** of mental disorders among individuals aged 10-24 (1990-2021).

**Figure S8**. Global and regional trends in the **age-standardized years lived with disability (YLDs) rates** of mental disorders among individuals aged 10-24 years (1990-2021).

**Figure S9**. Trends in age-standardized prevalence rates of depressive disorders and anxiety disorders among individuals aged 10-24 across global and 21 regions from 2019 to 2021.

**Figure S10**. Distribution of age-standardized prevalence, incidence, and years lived with disability (YLDs) rates of mental disorders among individuals aged 10-24 years across 21 regions in 2021.

**Figure S11**. Prevalence, incidence, and years lived with disability (YLDs) numbers of mental disorders among individuals aged 10-24 in 204 countries and territories in 2021, along with percentage changes from 2019 to 2021.

**Figure S12**. Global **incidence** of mental disorders by sex and age group (10-24 years) in 2021.

**Figure S13**. Global **years lived with disability (YLDs)** of mental disorders by sex and age group (10-24 years) in 2021.

**Figure S14**. Temporal trends in overall mental disorders across different age groups (10-24 years) from 1990 to 2021.

**Figure S15**. Sex differences in overall mental disorders among individuals aged 10-24 years across 21 regions in 2021.

**Figure S16**. Joinpoint regression analysis in the age-standardized **prevalence** rate of mental disorders among individuals aged 10-24 (1990-2021).

**Figure S17**. Joinpoint regression analysis in the age-standardized **incidence** rate of mental disorders among individuals aged 10-24 (1990-2021).

**Table S2.** Prevalence, Incidence, and Years Lived with Disability of Mental Disorders Among Adolescents and Young Adults (Aged 10-24) in **Andean Latin America** in 2021, and Percentage Change from 2019 to 2021 According to GBD.

|  | Prevalence (95% uncertainty interval) | | | Incidence (95% uncertainty interval) | | | Years lived with disability (95% uncertainty interval) | | |
| --- | --- | --- | --- | --- | --- | --- | --- | --- | --- |
| Cause | Counts in million | Age-standardized rates per 100,000 people | Percentage rate change (2019-2021) | Counts in million | Age-standardized rates per 100,000 people | Percentage rate change (2019-2021) | Counts in million | Age-standardized rates per 100,000 people | Percentage rate change (2019-2021) |
| Mental disorders | | | | | | | | | |
| Total | 3.25 (2.77 to 3.81) | 18,726.45 (15,603.41 to 22,512.31) | 0.20 (0.12 to 0.29) | 1.04 (0.81 to 1.32) | 5,964.14 (4,379.17 to 7,838.30) | 0.43 (0.31 to 0.57) | 0.41 (0.29 to 0.55) | 2,341.13 (1,623.26 to 3,221.99) | 0.27 (0.17 to 0.38) |
| Female | 1.63 (1.37 to 1.96) | 19,368.46 (15,767.20 to 23,803.58) | 0.27 (0.17 to 0.37) | 0.62 (0.48 to 0.80) | 7,318.91 (5,320.90 to 9,807.55) | 0.48 (0.34 to 0.63) | 0.23 (0.16 to 0.31) | 2,681.14 (1,826.01 to 3,712.32) | 0.32 (0.21 to 0.44) |
| Male | 1.61 (1.39 to 1.91) | 18,110.49 (15,158.64 to 21,718.75) | 0.14 (0.08 to 0.22) | 0.42 (0.34 to 0.51) | 4,688.30 (3,500.75 to 6,011.04) | 0.37 (0.25 to 0.49) | 0.18 (0.13 to 0.24) | 2,020.21 (1,414.81 to 2,759.47) | 0.22 (0.13 to 0.32) |
| Schizophrenia | | | | | | | | | |
| Total | 0.02 (0.01 to 0.03) | 92.52 (52.56 to 149.06) | 0.01 (-0.04 to 0.06) | 0.00 (0.00 to 0.00) | 21.69 (11.37 to 35.93) | 0.00 (-0.05 to 0.05) | 0.01 (0.01 to 0.02) | 61.74 (31.59 to 106.10) | 0.00 (-0.19 to 0.21) |
| Female | 0.01 (0.00 to 0.01) | 80.50 (45.46 to 131.17) | 0.00 (-0.07 to 0.09) | 0.00 (0.00 to 0.00) | 18.88 (9.70 to 31.79) | -0.01 (-0.08 to 0.07) | 0.00 (0.00 to 0.01) | 52.92 (25.68 to 94.34) | -0.01 (-0.25 to 0.3) |
| Male | 0.01 (0.01 to 0.02) | 103.92 (59.21 to 167.27) | 0.01 (-0.06 to 0.09) | 0.00 (0.00 to 0.00) | 24.34 (12.86 to 39.87) | 0.00 (-0.06 to 0.08) | 0.01 (0.00 to 0.01) | 70.12 (34.08 to 124.47) | 0.01 (-0.23 to 0.31) |
| Depressive disorders | | | | | | | | | |
| Total | 0.46 (0.34 to 0.64) | 2,617.13 (1,799.32 to 3,707.47) | 0.45 (0.3 to 0.64) | 0.60 (0.41 to 0.87) | 3,425.59 (2,209.14 to 5,067.56) | 0.61 (0.41 to 0.83) | 0.09 (0.05 to 0.13) | 491.99 (288.90 to 773.96) | 0.53 (0.32 to 0.74) |
| Female | 0.29 (0.21 to 0.41) | 3,379.51 (2,311.37 to 4,842.64) | 0.48 (0.32 to 0.7) | 0.39 (0.27 to 0.57) | 4,586.20 (2,926.62 to 6,867.31) | 0.63 (0.41 to 0.87) | 0.06 (0.03 to 0.09) | 645.67 (375.95 to 1,027.81) | 0.56 (0.33 to 0.79) |
| Male | 0.17 (0.13 to 0.23) | 1,898.70 (1,318.27 to 2,657.98) | 0.40 (0.24 to 0.57) | 0.21 (0.14 to 0.30) | 2,333.00 (1,496.20 to 3,445.61) | 0.58 (0.36 to 0.81) | 0.03 (0.02 to 0.05) | 347.20 (204.85 to 547.32) | 0.49 (0.28 to 0.72) |
| Bipolar disorder | | | | | | | | | |
| Total | 0.15 (0.10 to 0.22) | 818.61 (524.51 to 1,243.60) | 0.00 (-0.01 to 0.00) | 0.02 (0.01 to 0.03) | 125.16 (73.96 to 197.62) | 0.00 (0.00 to 0.00) | 0.03 (0.02 to 0.05) | 182.71 (100.68 to 305.35) | -0.01 (0.-09 to 0.07) |
| Female | 0.08 (0.05 to 0.12) | 893.44 (572.31 to 1,342.24) | -0.01 (-0.01 to -0.01) | 0.01 (0.01 to 0.02) | 135.79 (79.58 to 213.36) | 0.00 (0.00 to 0.00) | 0.02 (0.01 to 0.03) | 197.75 (108.87 to 335.82) | -0.01 (-0.11 to 0.1) |
| Male | 0.07 (0.05 to 0.10) | 748.15 (478.17 to 1,151.49) | 0.00 (0.00 to 0.00) | 0.01 (0.01 to 0.02) | 115.25 (67.63 to 184.72) | 0.00 (0.00 to 0.00) | 0.02 (0.01 to 0.03) | 168.55 (90.23 to 282.83) | 0.00 (-0.11 to 0.12) |
| Anxiety disorders | | | | | | | | | |
| Total | 1.43 (0.98 to 2.01) | 8,227.37 (5,398.28 to 12,009.27) | 0.49 (0.28 to 0.75) | 0.25 (0.16 to 0.37) | 1,454.63 (860.34 to 2,210.59) | 0.5 (0.27 to 0.76) | 0.17 (0.10 to 0.27) | 1,011.06 (584.69 to 1,600.87) | 0.49 (0.27 to 0.74) |
| Female | 0.88 (0.60 to 1.23) | 10,351.69 (6,789.86 to 15,070.96) | 0.5 (0.29 to 0.75) | 0.15 (0.09 to 0.22) | 1,738.67 (1,022.39 to 2,654.97) | 0.51 (0.29 to 0.77) | 0.11 (0.06 to 0.16) | 1,266.72 (739.93 to 2,015.21) | 0.5 (0.28 to 0.75) |
| Male | 0.56 (0.37 to 0.78) | 6,230.09 (4,021.19 to 9,305.90) | 0.49 (0.26 to 0.75) | 0.11 (0.07 to 0.16) | 1,188.12 (710.03 to 1,820.02) | 0.5 (0.25 to 0.75) | 0.07 (0.04 to 0.11) | 770.73 (431.74 to 1,241.16) | 0.48 (0.25 to 0.72) |
| Eating disorders | | | | | | | | | |
| Total | 0.10 (0.06 to 0.14) | 533.51 (326.73 to 824.16) | -0.01 (-0.07 to 0.06) | 0.08 (0.05 to 0.11) | 431.03 (237.80 to 731.90) | -0.01 (-0.08 to 0.06) | 0.02 (0.01 to 0.03) | 113.84 (59.11 to 193.12) | -0.02 (-0.1 to 0.08) |
| Female | 0.07 (0.05 to 0.11) | 818.91 (494.13 to 1,253.71) | -0.01 (-0.09 to 0.08) | 0.04 (0.02 to 0.06) | 459.68 (260.07 to 751.53) | -0.02 (-0.11 to 0.08) | 0.02 (0.01 to 0.02) | 174.09 (90.44 to 299.96) | -0.02 (-0.12 to 0.1) |
| Male | 0.02 (0.02 to 0.04) | 263.44 (156.18 to 443.14) | 0.00 (-0.08 to 0.07) | 0.04 (0.02 to 0.06) | 403.96 (207.84 to 718.62) | 0.00 (-0.07 to 0.07) | 0.01 (0.00 to 0.01) | 56.82 (27.76 to 103.14) | -0.01 (-0.15 to 0.17) |
| Autism spectrum disorders | | | | | | | | | |
| Total | 0.13 (0.11 to 0.15) | 731.30 (611.35 to 866.71) | 0.00 (-0.04 to 0.05) | NA | NA | NA | 0.02 (0.02 to 0.03) | 139.93 (94.97 to 197.05) | 0.00 (-0.07 to 0.08) |
| Female | 0.04 (0.03 to 0.05) | 494.93 (411.43 to 591.79) | 0.00 (-0.06 to 0.07) | NA | NA | NA | 0.01 (0.01 to 0.01) | 94.23 (63.19 to 133.59) | 0.01 (-0.11 to 0.13) |
| Male | 0.08 (0.07 to 0.10) | 952.99 (792.65 to 1,126.50) | 0.00 (-0.05 to 0.07) | NA | NA | NA | 0.02 (0.01 to 0.02) | 182.79 (124.47 to 258.94) | 0.00 (-0.09 to 0.12) |
| Attention-deficit/hyperactivity disorder | | | | | | | | | |
| Total | 0.74 (0.51 to 1.05) | 4,336.01 (2,919.41 to 6,206.42) | 0.00 (0.00 to 0.00) | 0.00 (0.00 to 0.01 | 22.59 (15.42 to 32.97) | 0.01 (0.01 to 0.01) | 0.01 (0.00 to 0.02) | 52.92 (27.30 to 88.52) | 0.00 (-0.05 to 0.05) |
| Female | 0.22 (0.15 to 0.31) | 2,637.64 (1,794.68 to 3,813.74) | 0.00 (0.00 to 0.00) | 0.00 (0.00 to 0.00) | 14.12 (9.33 to 21.14) | 0.02 (0.02 to 0.02) | 0.00 (0.00 to 0.00) | 32.02 (16.67 to 53.43) | 0.00 (-0.08 to 0.09) |
| Male | 0.52 (0.36 to 0.74) | 5,926.00 (3,990.72 to 8,563.97) | 0.00 (0.00 to 0.00) | 0.00 (0.00 to 0.00) | 30.48 (20.66 to 44.95) | 0.01 (0.01 to 0.01) | 0.01 (0.00 to 0.01) | 72.49 (36.70 to 123.60) | 0.00 (-0.06 to 0.05) |
| Conduct disorder | | | | | | | | | |
| Total | 0.31 (0.22 to 0.40) | 1,855.94 (1,267.12 to 2,540.44) | 0.00 (0.00 to 0.01) | 0.08 (0.05 to 0.11) | 483.45 (289.54 to 691.87) | 0.01 (0.01 to 0.01) | 0.04 (0.02 to 0.06) | 226.56 (117.86 to 377.75) | 0.00 (-0.05 to 0.06) |
| Female | 0.11 (0.07 to 0.15) | 1,381.60 (892.17 to 1,993.86) | 0.01 (0.00 to 0.01) | 0.03 (0.02 to 0.04) | 365.57 (212.09 to 561.48) | 0.01 (0.01 to 0.02) | 0.01 (0.01 to 0.02) | 167.74 (84.98 to 284.83) | 0.00 (-0.08 to 0.09) |
| Male | 0.20 (0.14 to 0.25) | 2,297.63 (1,583.47 to 3,087.20) | 0.00 (0.00 to 0.00) | 0.05 (0.03 to 0.07) | 593.14 (354.63 to 835.24) | 0.01 (0 to 0.01) | 0.02 (0.01 to 0.04) | 281.33 (145.73 to 463.99) | 0.00 (-0.06 to 0.07) |
| Idiopathic developmental intellectual disability | | | | | | | | | |
| Total | 0.07 (0.02 to 0.13) | 429.27 (109.02 to 728.52) | 0.01 (-0.09 to 0.11) | NA | NA | NA | 0.00 (0.00 to 0.01) | 19.51 (5.47 to 38.91) | 0.01 (-0.11 to 0.14) |
| Female | 0.03 (0.01 to 0.06) | 413.27 (136.12 to 673.96) | 0.01 (-0.09 to 0.11) | NA | NA | NA | 0.00 (0.00 to 0.00) | 18.72 (6.22 to 35.92) | 0.01 (-0.15 to 0.18) |
| Male | 0.04 (0.01 to 0.07) | 444.27 (81.75 to 777.73) | 0.01 (-0.08 to 0.13) | NA | NA | NA | 0.00 (0.00 to 0.00) | 20.26 (4.59 to 41.42) | 0.01 (-0.17 to 0.24) |
| Other mental disorders | | | | | | | | | |
| Total | 0.10 (0.06 to 0.13) | 528.94 (338.96 to 732.21) | 0.00 (0.00 to 0.00) | NA | NA | NA | 0.01 (0.00 to 0.01) | 40.86 (22.38 to 65.94) | 0.00 (-0.13 to 0.13) |
| Female | 0.04 (0.02 to 0.05) | 408.20 (259.10 to 576.09) | -0.01 (-0.01 to -0.01) | NA | NA | NA | 0.00 (0.00 to 0.00) | 31.29 (16.81 to 50.48) | -0.01 (-0.2 to 0.22) |
| Male | 0.06 (0.04 to 0.08) | 643.24 (409.75 to 899.04) | 0.00 (0.00 to 0.00) | NA | NA | NA | 0.00 (0.00 to 0.01) | 49.92 (26.79 to 80.04) | 0.00 (-0.17 to 0.18) |

**Table S3.** Prevalence, Incidence, and Years Lived with Disability of Mental Disorders Among Adolescents and Young Adults (Aged 10-24) in **Australasia** in 2021, and Percentage Change from 2019 to 2021 According to GBD.

|  | Prevalence (95% uncertainty interval) | | | Incidence (95% uncertainty interval) | | | Years lived with disability (95% uncertainty interval) | | |
| --- | --- | --- | --- | --- | --- | --- | --- | --- | --- |
| Cause | Counts in million | Age-standardized rates per 100,000 people | Percentage rate change (2019-2021) | Counts in million | Age-standardized rates per 100,000 people | Percentage rate change (2019-2021) | Counts in million | Age-standardized rates per 100,000 people | Percentage rate change (2019-2021) |
| Mental disorders | | | | | | | | | |
| Total | 1.39 (1.24 to 1.58) | 24,098.09 (20,921.15 to 27,731.83) | 0.03 (-0.03 to 0.10) | 0.61 (0.48 to 0.77) | 10,533.51 (7,881.56 to 13,843.48) | 0.08 (-0,06 to 0.25) | 0.20 (0.14 to 0.26) | 3,350.80 (2,412.39 to 4,498.48) | 0.04 (-0.04 to 0.14) |
| Female | 0.68 (0.59 to 0.77) | 23,974.99 (20,445.54 to 28,277.14) | 0.04 (-0.04 to 0.14) | 0.37 (0.29 to 0.48) | 13,130.39 (9,666.93 to 17,402.18) | 0.08 (-0.07 to 0.27) | 0.11 (0.08 to 0.11) | 3,746.47 (2,644.15 to 5,106.25) | 0.05 (-0.05 to 0.16) |
| Male | 0.72 (0.64 to 0.80) | 24,210.37 (21,082.73 to 27,609.86) | 0.02 (-0.03 to 0.07) | 0.24 (0.19 to 0.31) | 8,077.23 (6,136.58 to 10,579.47) | 0.08 (-0.07 to 0.25) | 0.11 (0.08 to 0.14) | 2,976.28 (2,178.46 to 3,929.50) | 0.03 (-0.04 to 0.12) |
| Schizophrenia | | | | | | | | | |
| Total | 0.01 (0.01 to 0.01) | 193.09 (157.63 to 234.15) | -0.01 (-0.09 to 0.07) | 0.00 (0.00 to 0.00) | 44.62 (35.66 to 55.04) | -0.01 (-0.08 to 0.07) | 0.01 (0.01 to 0.01) | 127.69 (87.14 to 172.37) | -0.02 (-0.18 to 0.17) |
| Female | 0.00 (0.00 to 0.00) | 126.13 (90.50 to 165.55) | -0.01 (-0.14 to 0.11) | 0.00 (0.00 to 0.00) | 29.55 (20.75 to 39.81) | -0.01 (-0.12 to 0.11) | 0.00 (0.00 to 0.00) | 81.91 (49.45 to 124.25) | -0.01 (-0.27 to 0.34) |
| Male | 0.01 (0.01 to 0.01) | 256.58 (212.77 to 304.82) | -0.02 (-0.10 to 0.08) | 0.00 (0.00 to 0.00) | 58.89 (47.91 to 70.52) | -0.01 (-0.11 to 0.09) | 0.00 (0.00 to 0.00) | 171.09 (116.59 to 228.50) | -0.02 (-0.20 to 0.20) |
| Depressive disorders | | | | | | | | | |
| Total | 0.31 90.23 to 0.420 | 5,352.97 (3,798.65 to 7,564.21) | 0.08 (-0.09 to 0.27) | 0.43 (0.30 to 0.58) | 7,350.00 (4,878.43 to 10,520.17) | 0.09 (-0.10 to 0.33) | 0.06 (0.04 to 0.09) | 1,038.63 (634.40 to 1,612.01) | 0.08 (-0.10 to 0.30) |
| Female | 0.20 (0.14 to 0.27) | 6,919.24 (4,827.48 to 9,703.62) | 0.08 (-0.10 to 0.29) | 0.27 (0.19 to 0.38) | 9,621.80 (6,317.41 to 13,825.01) | 0.09 (-0.11 to 0.36) | 0.04 (0.02 to 0.08) | 1,346.26 (809.55 to 2,101.94) | 0.08 (-0.11 to 0.33) |
| Male | 0.12 (0.09 to 0.16) | 3,871.05 (2,722.31 to 5,452.65) | 0.08 (-0.11 to 0.29) | 0.16 (0.11 to 0.22) | 5,201.09 (3,435.86 to 7,583.91) | 0.09 (-0.13 to 0.35) | 0.02 (0.01 to 0.03) | 747.58 (446.11 to 1,163.00) | 0.08 (-0.13 to 0.34) |
| Bipolar disorder | | | | | | | | | |
| Total | 0.08 (0.06 to 0.10) | 1,292.66 (978.97 to 1,658.16) | -0.01 (-0.01 to -0.01) | 0.01 (0.01 to 0.01) | 180.71 (128.63 to 246.29) | 0.01 (0.00 to 0.01) | 0.02 (0.01 to 0.03) | 285.95 (172.20 to 448.51) | -0.01 (-0.08 to 0.07) |
| Female | 0.04 (0.03 to 0.05) | 1,245.26 (941.86 to 1,621.69) | -0.01 (-0.01 to -0.01) | 0.00 (0.00 to 0.01) | 179.21 (124.84 to 248.68) | 0.01 (0.00 to 0.01) | 0.01 (0.00 to 0.01) | 273.00 (159.95 to 441.96) | -0.01 (-0.11 to 0.10) |
| Male | 0.04 (0.03 to 0.05) | 1,337.39 (995.80 to 1,746.88) | -0.01 (-0.01 to -0.01) | 0.01 (0.00 to 0.01) | 182.10 (129.42 to 249.47) | 0.01 (0.01 to 0.01) | 0.01 (0.01 to 0.01) | 298.19 (175.08 to 467.45) | -0.01 (-0.11 to 0.10) |
| Anxiety disorders | | | | | | | | | |
| Total | 0.44(0.31 to 0.610 | 7,513.19 (5,036.12 to 10,831.16) | 0.07 (-0.14 to 0.33) | 0.08 (0.05 to 0.11) | 1,365.84 (835.83 to 1,985.98) | 0.09 (-0.12 to 0.35) | 0.05 (0.03 to 0.08) | 914.14 (532.89 to 1,430.13) | 0.06 (-0.14 to 0.32) |
| Female | 0.26 (0.19 to 0.36) | 9,204.07 (6,156.99 to 13,232.14) | 0.09 (-0.13 to 0.35) | 0.04 (0.03 to 0.06) | 1,591.93 (968.77 to 2,352.34) | 0.10 (-0.11 to 0.38) | 0.03 (0.02 to 0.05) | 1,113.50 (650.38 to 1,724.24) | 0.09 (-0.13 to 0.34) |
| Male | 0.18 (0.12 to 0.25) | 5,913.55 (3,870.86 to 8,593.90) | 0.03 (-0.18 t0 0.31) | 0.03 (0.02 to 0.05) | 1,152.09 (704.92 to 1,703.41) | 0.07 (-0.15 to 0.34) | 0.02 (0.01 to 0.03) | 725.56 (410.91 to 1,146.16) | 0.03 (-0.19 to 0.32) |
| Eating disorders | | | | | | | | | |
| Total | 0.10 (0.07 to 0.13) | 1,644.33 (1,136.05 to 2,367.97) | 0.01 (-0.10 to 0.11) | 0.06 (0.04 to 0.09) | 1,063.08 (610.68 to 1,706.70) | 0.03 (-0.09 to 0.15) | 0.02 (0.01 to 0.03) | 350.49 (207.30 to 563.54) | 001 (-0.12 to 0.12) |
| Female | 0.07 (0.05 to 0.09) | 2,330.21 (1,658.44 to 3,225.84) | -0.03 (-0.14 to 0.10) | 0.04 (0.03 to 0.05) | 1,305.94 (775.06 to 2,076.17) | -0.01 (-0.14 to 0.18) | 0.01 (0.01 to 0.02) | 494.57 (300.20 to 772.45) | -0.03 (-0.16 to 0.11) |
| Male | 0.03 (0.02 to 0.04) | 995.28 (586.54 to 1,539.44) | 0.09 (-0.05 to 0.26) | 0.02 (0.02 to 0.04) | 833.68 (438.86 to 1,411.91) | 0.08 (-0.05 to 0.25) | 0.01 (0.00 to 0.01) | 214.14 (109.71 to 377.16) | 0.10 (-0.08 to 0.29) |
| Autism spectrum disorders | | | | | | | | | |
| Total | 0.07 (0.06 to 0.09) | 1,245.60 (1,039.89 to 1,487.98) | 0.00 (-0.07 to 0.07) | NA | NA | NA | 0.01 (0.01 to 0.02) | 236.27 (161.46 to 331.71) | 0.00 (-0.08 to 0.08) |
| Female | 0.02 (0.02 to 0.03) | 739.79 (613.91 to 899.15) | 0.00 (-0.09 to 0.11) | NA | NA | NA | 0.00 (0.00 to 0.01) | 139.20 (92.44 to 199.20) | 0.00 (-0.12 to 0.15) |
| Male | 0.05 (0.04 to 0.06) | 1,723.76 (1,437.16 to 2,072.67) | 0.00 (-0.09 to 0.10) | NA | NA | NA | 0.01 (0.01 to 0.01) | 328.04 (225.85 to 459.48) | 0.00 (-0.10 to 0.11) |
| Attention-deficit/hyperactivity disorder | | | | | | | | | |
| Total | 0.37 (0.27 to 0.47) | 6,449.47 (4,644.62 to 8,575.38) | 0.00 (-0.02 to 0.02) | 0.00 (0.00 to 0.00) | 35.41 (25.13 to 48.49) | 0.02 (0.00 to 0.04) | 0.00 (0.00 to 0.01) | 78.69 (42.30 to 125.42) | 0.00 (-0.06 to 0.06) |
| Female | 0.10 (0.07 to 0.13) | 3,524.74 (2,462.56 to 4,875.45) | 0.00 (-00.02 to 0.02) | 0.00 (0.00 to 0.00) | 19.44 (13.31 to 28.08) | 0.02 (0.00 to 0.05) | 0.00 (0.00 to 0.00) | 42.81 (22.24 to 71.51) | 0.00 (-0.09 to 0.10) |
| Male | 0.27 (0.20 to 0.34) | 9,212.92 (6,653.10 to 12,143.69) | 0.00 (-0.02 to 0.02) | 0.00 (0.00 to 0.00) | 50.49 (35.94 to 66.65) | 0.02 (0.00 to 0.04) | 0.00 (0.00 to 0.01) | 112.59 (60.45 to 177.03) | 00.00 (-0.06 to 0.07) |
| Conduct disorder | | | | | | | | | |
| Total | 0.11 (0.08 to 0.14) | 1,987.67 (1,381.04 to 2,678.53) | 0.01 (0.01 to 0.01) | 0.03 (0.02 to 0.04) | 493.84 (291.19 to 710.14) | 0.02 (0.02 to 0.02) | 0.01 (0.01 to 0.02) | 241.87 (126.39 to 396.84) | 0.01 (-0.05 to 0.08) |
| Female | 0.04 (0.03 to 0.05) | 1,500.38 (982.40 to 2,146.05) | 0.01 (0.01 to 0.02) | 0.01 (0.01 to 0.01) | 382.51 (224.08 to 579.37) | 0.02 (0.02 to 0.02) | 0.00 (0.00 to 0.01) | 181.82 (91.30 to 313.44) | 0.01 (-0.09 to 0.15) |
| Male | 0.07 (0.05 to 0.09) | 2,447.64 (1,719.63 to 3,254.62) | 0.01 (0.01 to 0.01) | 0.02 (0.01 to 0.02) | 598.90 (356.14 to 855.64) | 0.02 (0.01 to 0.02) | 0.01 (0.00 to 0.01) | 298.56 (158.66 to 481.71) | 0.01 (-0.07 to 0.11) |
| Idiopathic developmental intellectual disability | | | | | | | | | |
| Total | 0.02 (0.00 to 0.03) | 296.57 (50.18 to 597.09) | 0.02 (-0.06 to 0.14) | NA | NA | NA | 0.00 (0.00 to 0.00) | 14.26 (3.23 to 30.07) | 0.02 (-0.13 to 0.27) |
| Female | 0.01 (0.00 to 0.02) | 391.68 (84.40 to 690.03) | 0.05 (-0.02 to 0.13) | NA | NA | NA | 0.00 (0.00 to 0.00) | 19.00 (5.48 to 36.94) | 0.04 (-0.16 to 0.39) |
| Male | 0.01 (0.00 to 0.02) | 206.67 (10.49 to 510.91) | -0.02 (-0.34 to 0.37) | NA | NA | NA | 0.00 (0.00 to 0.00) | 9.78 (0.32 to 25.14) | -0.02 (-0.38 to 0.41) |
| Other mental disorders | | | | | | | | | |
| Total | 0.05 (0.04 to 0.06) | 824.61 (629.97 to 1,047.80) | -0.01 (-0.01 to -0.01) | NA | NA | NA | 0.00 (0.00 to 0.01) | 62.80 (37.96 to 94.00) | -0.02 (-0.15 to 0.14) |
| Female | 0.02 (0.02 to 0.03) | 721.05 (535.76 to 929.04) | -0.01 (-0.01 to -0.01) | NA | NA | NA | 0.00 (0.00 to 0.00) | 54.40 (30.81 to 83.36) | -0.02 (-0.20 to 0.21) |
| Male | 0.03 (0.02 to 0.04) | 922.70 (710.09 to 1,172.69) | -0.01 (-0.01 to -0.01) | NA | NA | NA | 0.00 (0.00 to 0.00) | 70.75 (41.40 to 108.99) | -0.02 (-0.19 to 0.19) |

**Table S4.** Prevalence, Incidence, and Years Lived with Disability of Mental Disorders Among Adolescents and Young Adults (Aged 10-24) in **Caribbean** in 2021, and Percentage Change from 2019 to 2021 According to GBD.

|  | Prevalence (95% uncertainty interval) | | | Incidence (95% uncertainty interval) | | | Years lived with disability (95% uncertainty interval) | | |
| --- | --- | --- | --- | --- | --- | --- | --- | --- | --- |
| Cause | Counts in million | Age-standardized rates per 100,000 people | Percentage rate change (2019-2021) | Counts in million | Age-standardized rates per 100,000 people | Percentage rate change (2019-2021) | Counts in million | Age-standardized rates per 100,000 people | Percentage rate change (2019-2021) |
| Mental disorders | | | | | | | | | |
| Total | 2.10 (1.82 to 2.45) | 18,509.26 (15,538.53 to 21,862.34) | 0.10 (0.06 to 0.14) | 0.70 (0.55 to 0.90) | 6,114.07 (4,483.34 to 8,161.63) | 0.26 (0.18 to 0.36) | 0.24 (0.18 to 0.33) | 2,119.46 (1,497.91 to 2,903.60) | 0.16 (0.10 to 0.22) |
| Female | 1.00 (0.95 to 1.17) | 17,776.00 (14,674.15 to 21,424.88) | 0.14 (0.09 to 0.20) | 0.44 (0.33 to 0.58) | 7,661.35 (5,515.68 to 10,603.88) | 0.29 (0.19 to 0.40) | 0.14 (0.10 to 0.19) | 2,388.01 (1,649.68 to 3,301.60) | 0.19 (0.11 to 0.26) |
| Male | 1.10 (0.94 to 1.28) | 19,216.42 (16,163.80 to 22,813.47) | 0.06 (0.04 to 0.09) | 0.26 (0.21 to 0.36) | 4,595.92 (3,491.40 to 5,913.97) | 0.23 (0.15 to 0.31) | 0.11 (0.08 to 0.14) | 1,855.55 (1,325.89 to 2,517.64) | 0.12 (0.06 to 0.18) |
| Schizophrenia | | | | | | | | | |
| Total | 0.01 (0.01 to 0.02) | 89.09 (51.47 to 143.37) | 0.00 (-0.04 to 0.04) | 0.00 (0.00 to 0.00) | 20.09 (10.58 to 33.34) | 0.00 (-0.04 to 0.04) | 0.01 (0.00 to 0.01) | 59.30 (32.07 to 100.46) | -0.01 (-0.13 to 0.13) |
| Female | 0.00 (0.00 to 0.01) | 77.62 (44.46 to 124.56) | 0.00 (-0.05 to 0.07) | 0.00 (0.00 to 0.00) | 17.57 (9.19 to 29.17) | 0.00 (-0.06 to 0.06) | 0.00 (0.00 to 0.00) | 50.91 (26.42 to 87.73) | 0.00 (-0.18 to 0.22) |
| Male | 0.01 (0.00 to 0.01) | 100.37 (58.10 to 159.24) | -0.01 (-0.05 to 0.05) | 0.00 (0.00 to 0.00) | 22.58 (11.90 to 37.01) | -0.01 (-0.05 to 0.06) | 0.00 (0.00 to 0.01) | 67.57 (35.95 to 115.10) | -0.01 (-0.18 to 0.19) |
| Depressive disorders | | | | | | | | | |
| Total | 0.35 (0.26 to 0.49) | 3,016.43 (2,075.60 to 4,351.99) | 0.28 (0.17 to 0.40) | 0.47 (0.33 to 0.67) | 4,056.83 (2,634.13 to 6,104.58) | 0.35 (0.21 to 0.48) | 0.07 (0.04 to 0.10) | 572.88 (339.18 to 895.98) | 0.31 (0.19 to 0.45) |
| Female | 0.23 (0.16 to 0.32) | 3,978.10 (2,683.69 to 5,842.87) | 0.29 (0.17 to 0.41) | 0.32 (0.21 to 0.46) | 5,528.22 (3,534.50 to 8,420.83) | 0.35 (0.21 to 0.50) | 0.04 (0.03 to 0.07) | 765.84 (447.93 to 1,212.98) | 0.32 (0.18 to 0.47) |
| Male | 0.12 (0.09 to 0.16) | 2,072.72 (1,458.21 to 2,882.21) | 0.25 (0.15 to 0.38) | 0.15 (0.11 to 0.21) | 2,613.82 (1,755.45 to 3,844.28) | 0.34 (0.22 to 0.50) | 0.02 (0.01 to 0.03) | 383.55 (228.14 to 596.01) | 0.30 (0.16 to 0.46) |
| Bipolar disorder | | | | | | | | | |
| Total | 0.10 (0.06 to 0.15) | 821.00 (522.87 to 1,248.73) | 0.00 (-0.01 to 0.00) | 0.01 (0.01 to 0.02) | 125.66 (74.08 to 198.56) | 0.00 (0.00 to 0.00) | 0.02 (0.01 to 0.04) | 181.63 (100.74 to 301.70) | -0.01 (-0.06 to 0.05) |
| Female | 0.05 (0.03 to 0.08) | 895.16 (570.42 to 1,349.82) | 0.00 (-0.01 to 0.00) | 0.01 (0.00 to 0.01) | 136.43 (80.99 to 215.04) | 0.00 (0.00 to 0.00) | 0.01 (0.01 to 0.02) | 196.06 (109.52 to 325.45) | -0.01 (-0.09 to 0.06) |
| Male | 0.04 (0.03 to 0.07) | 748.20 (480.68 to 1,148.18) | 0.00 (-0.01 to 0.00) | 0.01 (0.00 to 0.01) | 115.15 (66.94 to 183.34) | 0.00 (0.00 to 0.00) | 0.01 (0.01 to 0.02) | 167.47 (90.18 to 283.06) | -0.01 (-0.09 to 0.08) |
| Anxiety disorders | | | | | | | | | |
| Total | 0.68 (0.47 to 0.94) | 5,934.71 (3,887.29 to 8,648.41) | 0.26 (0.12 to 0.41) | 0.12 (0.08 to 0.17) | 1,028.62 (632.16 to 1,547.72) | 0.27 90.13 to 0.41) | 0.08 (0.05 to 0.13) | 724.10 (421.70 to 1,146.20) | 0.26 (0.12 to 0.42) |
| Female | 0.42 (0.30 to 0.58) | 7,503.90 (4,885.91 to 10,864.98) | 0.27 (0.13 to 0.42) | 0.07 (0.05 to 0.10) | 1,250.61 (767.96 to 1,882.19) | 0.27 (0.13 to 0.41) | 0.05 (0.03 to 0.08) | 909.98 (531.59 to 1,427.02) | 0.27 (0.12 to 0.43) |
| Male | 0.25 (0.17 to 0.36) | 4,398.01 (2,882.80 to 6,564.94) | 0.26 (0.12 to 0.41) | 0.05 (0.03 to 0.07) | 811.47 (500.67 to 1,221.45) | 0.26 (0.12 to 0.41) | 0.03 (0.02 to 0.05) | 542.09 (309.87 to 870.64) | 0.26 (0.11 to 0.43) |
| Eating disorders | | | | | | | | | |
| Total | 0.05 (0.03 to 0.07) | 398.27 (250.55 to 617.96) | -0.01 (-0.05 to 0.03) | 0.04 (0.03 to 0.06) | 372.96 (204.26 to 636.71) | 0.00 (-0.04 to 0.04) | 0.01 (0.01 to 0.02) | 84.95 (45.26 to 144.93) | -0.01 (-0.07 to 0.07) |
| Female | 0.03 (0.02 to 0.05) | 547.02 (342.65 to 832.09) | -0.01 (-0.07 to 0.04) | 0.02 (0.01 to 0.03) | 348.59 (200.95 to 569.12) | -0.01 (-0.06 to 0.05) | 0.01 (0.00 to 0.01) | 115.73 (61.97 to 195.04) | -0.02 (-0.09 to 0.08) |
| Male | 0.01 (0.01 to 0.02) | 251.97 (145.86 to 421.24) | 0.00 (-0.05 to 0.05) | 0.02 (.01 to 0.04) | 396.77 (202.62 to 701.52) | 0.01 (-0.04 to 0.05) | 0.00 (0.00 to 0.01) | 54.68 (26.88 to 99.32) | 0.00 (-0.10 to 0.13) |
| Autism spectrum disorders | | | | | | | | | |
| Total | 0.08 (0.07 to 0.10) | 723.34 (607.74 to 859.48) | 0.00 (-0.04 to 0.04) | NA | NA | NA | 0.02 (0.01 to 0.02) | 137.37 (94.51 to 192.61) | -0.01 (-0.06 to 0.05) |
| Female | 0.03 (0.02 to 0.03) | 493.55 (407.66 to 591.17) | 0.00 (-0.05 to 0.04) | NA | NA | NA | 0.01 (0.00 to 0.01) | 92.95 (62.65 to 130.68) | -0.01 (-0.08 to 0.08) |
| Male | 0.05 (0.05 to 0.06) | 948.05 (799.57 to 1,123.03) | 0.00 (-0.05 to 0.05) | NA | NA | NA | 0.01 (0.01 to 0.01) | 180.80 (123.35 to 254.31) | 0.00 (-0.08 to 0.08) |
| Attention-deficit/hyperactivity disorder | | | | | | | | | |
| Total | 0.68 (0.47 to 0.92) | 6,065.00 (4,187.40 to 8,581.71) | 0.00 (0.00 to 0.00) | 0.00 (0.00 to 0.00) | 30.97 (21.22 to 45.77) | 0.01 (0.01 to 0.01) | 0.01 (0.00 to 0.01) | 73.88 (39.21 to 123.81) | 0.00 (-0.03 to 0.03) |
| Female | 0.18 (0.13 to 0.26) | 3,339.14 (2,268.45 to 4,847.52) | 0.00 (0.00 to 0.00) | 0.00 (0.00 to 0.00) | 17.83 (11.87 to 26.73) | 0.01 (0.01 to 0.01) | 0.00 (0.00 to 0.00) | 40.48 (20.82 to 69.68) | 0.00 (-0.06 to 0.06) |
| Male | 0.50 (0.35 to 0.68) | 8,727.45 (6,041.84 to 12,411.82) | 0.00 (0.00 to 0.00) | 0.00 (0.00 to 0.00) | 43.72 (30.21 to 64.54) | 0.01 (0.01 to 0.01) | 0.01 (0.00 to 0.01) | 106.50 (56.32 to 179.05) | 0.00 (-0.04 to 0.04) |
| Conduct disorder | | | | | | | | | |
| Total | 0.20 (0.14 to 0..26) | 1,836.54 (1,240.51 to 2,542.31) | 0.00 (0.00 to 0.00) | 0.05 (0.03 to 0.07) | 478.94 (278.36 to 686.11) | 0.01 (0.01 to 0.01) | 0.02 (0.01 to 0.04) | 222.45 (116.16 to 365.13) | 0.00 (-0.04 to 0.04) |
| Female | 0.07 (0.05 to 0.10) | 1,363.74 (877.68 to 1,976.57) | 0.00 (0.00 to 0.00) | 0.02 (0.01 to 0.03) | 362.10 (203.22 to 555.92) | 0.01 (0.01 to 0.01) | 0.01 (0.00 to 0.01) | 163.83 (82.58 to 274.65) | 0.00 (-0.06 to 0.08) |
| Male | 0.13 (0.09 to 0.16) | 2,296.78 (1,577.06 to 3,120.32) | 0.00 (0.00 to 0.00) | 0.03 (0.02 to 0.04) | 592.41 (351.72 to 837.43) | 0.01 (0.00 to 0.01) | 0.02 (0.01 to 0.02) | 279.52 (148.49 to 454.26) | 0.00 (-0.05 to 0.06) |
| Idiopathic developmental intellectual disability | | | | | | | | | |
| Total | 0.06 (0.02 to 0.10) | 535.15 (150.93 to 905.37) | 0.01 (-0.18 to 0.27) | NA | NA | NA | 0.00 (0.00 to 0.01) | 22.44 (5.82 to 44.57) | 0.01 (-0.08 to 0.12) |
| Female | 0.03 (0.01 to 0.05) | 514.73 (170.77 to 851.68) | 0.01 9-0.18 to 0.25) | NA | NA | NA | 0.00 (0.00 to 0.00) | 21.13 (6.10 to 41.80) | 0.01 (-0.11 to 0.14) |
| Male | 0.03 (0.01 to 0.05) | 555.10 (131.53 to 961.65) | 0.02 (-0.18 to 0.29) | NA | NA | NA | 0.00 (0.00 to 0.00) | 23.72 (5.45 to 47.69) | 0.01 9-0.10 to 0.15) |
| Other mental disorders | | | | | | | | | |
| Total | 0.06 (0.04 to 0.09) | 526.67 (337.53 to 728.98) | 0.00 (0.00 to 0.00) | NA | NA | NA | 0.00 (0.00 to 0.01) | 40.45 (22.99 to 63.91) | 0.00 (-0.09 to 0.09) |
| Female | 0.02 (0.02 to 0.03) | 408.20 (259.10 to 576.09) | 0.00 (0.00 to 0.00) | NA | NA | NA | 0.00 (0.00 to 0.00) | 31.11 (16.99 to 49.02) | 0.00 (-0.14 to 0.17) |
| Male | 0.04 (0.02 to 0.05) | 643.24 (409.75 to 899.04) | 0.00 (0.00 to 0.00) | NA | NA | NA | 0.00 (0.00 to 0.00) | 49.64 (27.77 to 79.31) | -0.01 (-0.12 to 0.13) |

**Table S5.** The Prevalence, Incidence, and Years Lived with Disability of Mental Disorders Among Adolescents and Young Adults (Aged 10-24) in **Central Asia** in 2021, and Percentage Change from 2019 to 2021 According to GBD.

|  | Prevalence (95% uncertainty interval) | | | Incidence (95% uncertainty interval) | | | Years lived with disability (95% uncertainty interval) | | |
| --- | --- | --- | --- | --- | --- | --- | --- | --- | --- |
| Cause | Counts in million | Age-standardized rates per 100,000 people | Percentage rate change (2019-2021) | Counts in million | Age-standardized rates per 100,000 people | Percentage rate change (2019-2021) | Counts in million | Age-standardized rates per 100,000 people | Percentage rate change (2019-2021) |
| Mental disorders | | | | | | | | | |
| Total | 2.67 (2.34 to 3.02) | 12,073.73 (10,333.96 to 14,021.48) | 0.10 (0.06 to 0.14) | 1.10 (0.85 to 1.42) | 4,995.34 (3,675.43 to 6,692.63) | 0.10 (0.06 to 0.14) | 0.35 (0.26 to 0.47) | 1,602.12 (1,144.58 to 2,152.58) | 0.13 (0.08 to 0.19) |
| Female | 1.26 (1.09 to 1.44) | 11,715.11 (9,888.20 to 13,828.38) | 0.13 (0.08 to 0.19) | 0.59 (0.45 to 0.78) | 5,533.92 (3,997.59 to 7,572.40) | 0.26 (0.16 to 0.36) | 0.18 (0.13 to 0.24) | 1,660.35 (1,162.20 to 2,277.47) | 0.16 (0.10 to 0.23) |
| Male | 1.41 (1.24 to 1.58) | 12,406.56 (10,661.02 to 14,283.03) | 0.08 (0.05 to 0.11) | 0.51 (0.40 to 0.64) | 4,483.97 (3,322.58 to 5,928.19) | 0.02 (0.13 to 0.31) | 0.18 (0.13 to 0.23) | 1,546.39 (1,112.48 to 2,075.85) | 0.11 (0.06 to 0.17) |
| Schizophrenia | | | | | | | | | |
| Total | 0.02 (0.01 to 0.03) | 77.89 (43.79 to 130.32) | -0.05 (-0.10 to -0.01) | 0.00 (0.00 to 0.01) | 19.51 (10.22 to 32.40) | -0.05 (-0.10 to -0.01) | 0.01 (0.01 to 0.02) | 52.32 (27.56 to 91.89) | -0.05 (-0.18 to 0.10) |
| Female | 0.01 (0.00 to 0.01) | 75.61 (42.60 to 127.63) | -0.05 (-0.13 to 0.01) | 0.00 (0.00 to 0.00) | 19.17 (10.00 to 32.07) | -0.04 (-0.10 to 0.03) | 0.01 (0.00 to 0.01) | 50.12 (25.81 to 87.26) | -0.06 (-0.22 to 0.14) |
| Male | 0.01 (0.01 to 0.01) | 80.05 (45.03 to 133.70) | -0.05 (-0.12 to 0.01) | 0.00 (0.00 to 0.00) | 19.83 (10.41 to 32.96) | -0.04 (-0.10 to 0.03) | 0.01 (0.00 to 0.01) | 54.42 (28.04 to 95.57) | -0.05 (-0.24 to 0.17) |
| Depressive disorders | | | | | | | | | |
| Total | 0.61 (0.45 to 0.84) | 2,790.51 (1,945.14 to 3,870.52) | 0.21 (0.11 to 0.32) | 0.78 (0.54 to 1.09) | 2,790.51 (1,945.14 to 3,870.52) | 0.21 (0.11 to 0.32) | 0.11 (0.07 to 0.17) | 520.08 (315.24 to 806.33) | 0.25 (0.12 to 0.38) |
| Female | 0.34 (0.25 to 0.47) | 3,207.05 (2,219.30 to 4,482.08) | 0.22 (0.10 to 0.33) | 0.44 (0.30 to 0.62) | 3,207.05 (2,219.30 to 4,482.08) | 0.31 (0.17 to 0.44) | 0.06 (0.04 to 0.10) | 598.01 (358.23 to 934.33) | 0.26 (0.13 to 0.39) |
| Male | 0.27 (0.19 to 0.36) | 2,395.26 (1,686.78 to 3,302.15) | 0.20 (0.09 to 0.32) | 0.34 (0.24 to 0.46) | 2,395.26 (1,686.78 to 3,302.15) | 0.29 (0.14 to 0.43) | 0.05 (0.03 to 0.08) | 446.16 (269.71 to 693.72) | 0.24 (0.10 to 0.39) |
| Bipolar disorder | | | | | | | | | |
| Total | 0.08 (0.05 to 0.12) | 376.85 (234.45 to 585.36) | -0.03 (-0.04 to -0.03) | 0.01 (0.01 to 0.02) | 61.10 (34.64 to 98.84) | -0.03 (-0.04 to -0.03) | 0.02 (0.01 to 0.03) | 84.24 (45.10 to 142.98) | -0.04 (-0.12 to 0.05) |
| Female | 0.04 (0.03 to 0.06) | 394.43 (245.77 to 606.41) | -0.03 (-0.04 to -0.03) | 0.01 (0.00 to 0.01) | 63.73 (36.23 to 102.55) | 0.00 (-0.01 to 0.02) | 0.01 (0.01 to 0.02) | 87.47 (46.22 to 148.93) | -0.04 (-0.13 to 0.08) |
| Male | 0.04 (0.03 to 0.06) | 360.15 (223.28 to 561.38) | -0.03 (-0.04 to -0.03) | 0.01 (0.00 to 0.01) | 58.63 (33.10 to 95.04) | 0.00 (-0.01 to 0.02) | 0.01 (0.00 to 0.02) | 81.17 (42.05 to 138.84) | -0.03 (-0.13 to 0.10) |
| Anxiety disorders | | | | | | | | | |
| Total | 0.67 (0.47 to 0.94) | 3,047.87 (2,046.88 to 4,471.38) | 0.29 (0.15 to 0.44) | 0.12 (0.08 to 0.18) | 555.71 (337.19 to 829.91) | 0.29 (0.15 to 0.44) | 0.08 (0.05 to 0.13) | 375.57 (217.32 to 585.10) | 0.28 (0.14 to 0.45) |
| Female | 0.41 (0.29 to 0.57) | 3,832.80 (2,581.23 to 5,589.34) | 0.30 90.16 to 0.44) | 0.07 (0.05 to 0.11) | 682.69 (417.73 to 1,024.87) | 0.31 (0.17 to 0.47) | 0.05 (0.03 to 0.08) | 469.81 (271.84 to 739.63) | 0.30 (0.15 to 0.45) |
| Male | 0.26 (0.18 to 0.37) | 2,306.23 (1,534.76 to 3,384.06) | 0.28 (0.14 to 0.43) | 0.05 (0.03 to 0.07) | 435.91 (261.78 to 653.11) | 0.29 (0.15 to 0.45) | 0.03 (0.02 to 0.05) | 286.54 (163.24 to 453.14) | 0.28 (0.13 to 0.46) |
| Eating disorders | | | | | | | | | |
| Total | 0.06 (0.04 to 0.09) | 291.77 (185.34 to 454.20) | -0.02 (-0.06 to 0.02) | 0.07 (0.04 to 0.10) | 318.56 (175.52 to 547.86) | -0.02 (-0.06 to 0.02) | 0.01 (0.01 to 0.02) | 62.63 (33.62 to 107.30) | -0.12 (-0.10 to 0.06) |
| Female | 0.04 (0.03 to 0.06) | 376.08 (244.61 to 568.61) | -0.02 (-0.07 to 0.03) | 0.03 (0.02 to 0.04) | 266.91 (158.42 to 432.25) | 0.01 (-0.05 to 0.05) | 0.01 (0.00 to 0.01) | 80.52 (43.92 to 132.96) | -0.02 (-0.11 to 0.08) |
| Male | 0.02 (0.02 to 0.04) | 211.72 (125.45 to 354.11) | -0.02 (-0.07 to 0.03) | 0.04 (0.03 to 0.06) | 367.51 (188.19 to 658.13) | 0.00 (-0.07 to 0.06) | 0.01 (0.00 to 0.01) | 45.65 (22.61 to 82.25) | -0.02 (-0.14 to 0.11) |
| Autism spectrum disorders | | | | | | | | | |
| Total | 0.21 (0.18 to 0.25) | 945.67 (794.23 to 1,112.84) | 0.00 (-0.03 to 0.04) | NA | NA | NA | 0.04 (0.03 to 0.06) | 180.86 (123.41 to 255.73) | 0.00 (-0.05 to 0.06) |
| Female | 0.07 (0.06 to 0.08) | 661.36 (549.23 to 779.20) | 0.00 (-0.05 to 0.05) | NA | NA | NA | 0.01 (0.01 to 0.02) | 125.69 (86.26 to 176.07) | 0.00 (-0.08 to 0.08) |
| Male | 0.14 (0.12 to 0.16) | 1,214.09 (1,022.07 to 1,433.21) | 0.00 (-0.05 to 0.05) | NA | NA | NA | 0.03 (0.02 to 0.04) | 232.94 (158.63 to 327.21) | 0.00 (-0.07 to 0.07) |
| Attention-deficit/hyperactivity disorder | | | | | | | | | |
| Total | 0.45 (0.31 to 0.63) | 2,027.90 (1,368.54 to 2,925.70) | 0.01 (0.01 to 0.02) | 0.00 (0.00 to 0.00) | 10.91 (7.34 to 16.19) | 0.01 (0.01 to 0.02) | 0.01 (0.00 to 0.01) | 24.83 (12.58 to 41.90) | 0.02 (-0.04 to 0.07) |
| Female | 0.13 (0.09 to 0.18) | 1,208.16 (810.80 to 1,756.22) | 0.01 (0.01 to 0.02) | 0.00 (0.00 to 0.00) | 6.57 (4.34 to 9.78) | 0.06 (0.06 to 0.06) | 0.00 (0.00 to 0.00) | 14.70 (7.36 to 25.66) | 0.01 (-0.08 to 0.10) |
| Male | 0.32 (0.22 to 0.46) | 2,800.34 (1,894.50 to 4,054.98) | 0.01 (0.01 to 0.02) | 0.00 (0.00 to 0.00) | 14.96 (10.14 to 22.31) | 0.06 (0.06 to 0.06) | 0.00 (0.00 to 0.01) | 34.38 (17.01 to 58.17) | 0.01 (-0.05 to 0.08) |
| Conduct disorder | | | | | | | | | |
| Total | 0.42 (0.30 to 0.56) | 1,894.40 (1,291.90 to 2,614.65) | 0.04 (0.03 to 0.04) | 0.11 (0.07 to 0.15) | 486.41 (292.21 to 690.29) | 0.04 (0.03 to 0.04) | 0.05 (0.03 to 0.08) | 231.51 (119.93 to 381.06) | 0.04 (0.00 to 0.08) |
| Female | 0.16 (0.11 to 0.21) | 1,460.66 (948.22 to 2,101.68) | 0.04 (0.04 to 0.05) | 0.4 (0.03 to 0.06) | 378.58 (220.70 to 567.29) | 0.05 (0.05 to 0.06) | 0.02 (0.01 to 0.03) | 177.46 (90.14 to 298.80) | 0.04 (-0.02 to 0.10) |
| Male | 0.27 (0.19 to 0.35) | 2,301.32 (1,592.76 to 3,147.03) | 0.04 (0.03 to 0.04) | 0.07 (0.04 to 0.09) | 587.26 (352.38 to 828.70) | 0.05 (0.04 to 0.05) | 0.03 (0.02 to 0.05) | 282.21 (146.08 to 461.28) | 0.04 (-0.01 to 0.09) |
| Idiopathic developmental intellectual disability | | | | | | | | | |
| Total | 0.16 (0.06 to 0.26) | 724.46 (265.93 to 1,159.13) | -0.02 (-0.14 to 0.08) | NA | NA | NA | 0.01 (0.00 to 0.01) | 29.27 (9.13 to 56.48) | -0.02 (-0.09 to 0.04) |
| Female | 0.07 (0.03 to 0.11) | 671.92 (274.17 to 1,037.44) | -0.02 (-0.13 to 0.10) | NA | NA | NA | 0.00 (0.00 to 0.01) | 25.38 (8.15 to 48.62) | -0.01 (-0.09 to 0.07) |
| Male | 0.09 (0.03 to 0.14) | 774.03 (251.73 to 1,267.63) | -0.03 (-0.16 to 0.09 | NA | NA | NA | 0.00 (0.00 to 0.01) | 32.96 (10.15 to 64.43) | -0.02 (-0.12 to 0.06) |
| Other mental disorders | | | | | | | | | |
| Total | 0.12 (0.07 to 0.16) | 528.57 (338.73 to 731.69) | -0.04 (-0.04 to -0.04) | NA | NA | NA | 0.01 (0.01 to 0.01) | 40.81 (23.14 to 65.23) | -0.04 (-0.13 0.07) |
| Female | 0.04 (0.03 to 0.06) | 408.20 (259.10 to 576.09) | -0.04 (-0.05 to -0.04) | NA | NA | NA | 0.00 (0.00 to 0.01) | 31.20 (16.84 to 51.58) | -0.04 (-0.18 to 0.13) |
| Male | 0.07 (0.05 to 0.10) | 643.24 (409.75 to 899.04) | -0.04 (-0.04 to -0.04) | NA | NA | NA | 0.01 (0.00 to 0.01) | 49.97 (27.21 to 80.12) | -0.05 (-0.16 to 0.08) |

**Table S6.** Prevalence, Incidence, and Years Lived with Disability of Mental Disorders Among Adolescents and Young Adults (Aged 10-24) in **Central Europe** in 2021, and Percentage Change from 2019 to 2021 According to GBD.

|  | Prevalence (95% uncertainty interval) | | | Incidence (95% uncertainty interval) | | | Years lived with disability (95% uncertainty interval) | | |
| --- | --- | --- | --- | --- | --- | --- | --- | --- | --- |
| Cause | Counts in million | Age-standardized rates per 100,000 people | Percentage rate change (2019-2021) | Counts in million | Age-standardized rates per 100,000 people | Percentage rate change (2019-2021) | Counts in million | Age-standardized rates per 100,000 people | Percentage rate change (2019-2021) |
| Mental disorders | | | | | | | | | |
| Total | 2.43 (2.13 to 2.75) | 13,371.80 (11,416.39 to 15,555.71) | 0.17 (0.13 to 0.21) | 0.86 (0.70 to 1.05) | 13,371.80 (11,416.39 to 15,555.71) | 0.36 (0.29 to 0.43) | 0.32 (0.23 to 0.43) | 1,776.93 (1,266.53 to 2,392.23) | 0.21 (0.16 to 0.26) |
| Female | 1.20 (1.04 to 1.39) | 13,637.76 (11,392.99 to 16,157.26) | 0.22 (0.18 to 0.27) | 0.47 (0.38 to 0.60) | 13,637.76 (11,392.99 to 16,157.26) | 0.40 (0.32 to 0.48) | 0.17 (0.12 to 0.23) | 1,925.07 (1,342.58 to 2,621.27) | 0.26 (0.21 to 0.31) |
| Male | 1.23 (1.08 to 1.37) | 13,119.73 (11,289.06 to 15,096.21) | 0.12 (0.09 to 0.15) | 0.38 (0.31 to 0.46) | 13,119.73 (11,289.06 to 15,096.21) | 0.31 (0.25 to 0.38) | 0.15 (0.11 to 0.20) | 1,637.23 (1,176.97 to 2,176.37) | 0.17 (0.12 to 0.21) |
| Schizophrenia | | | | | | | | | |
| Total | 0.01 (0.01 to 0.02) | 77.76 (45.83 to 124.43) | -0.01 (-0.04 to 0.01) | 0.00 (0.00 to 0.01) | 77.76 (45.83 to 124.43) | -0.01 (-0.03 to 0.02) | 0.01 (0.01 to 0.02) | 52.18 (29.64 to 86.30) | -0.01 (-0.09 to 0.07) |
| Female | 0.01 (0.00 to 0.01) | 75.50 (44.36 to 121.43) | -0.01 (-0.04 to 0.02) | 0.00 (0.00 to 0.00) | 75.50 (44.36 to 121.43) | 0.00 (-0.03 to 0.03) | 0.00 (0.00 to 0.01) | 50.25 (28.26 to 84.26) | -0.01 (-0.13 to 0.11) |
| Male | 0.01 (0.00 to 0.01) | 79.89 (47.23 to 127.00) | -0.01 (-0.05 to 0.02) | 0.00 (0.00 to 0.00) | 79.89 (47.23 to 127.00) | -0.01 (-0.04 to 0.02) | 0.01 (0.00 to 0.01) | 54.00 (30.68 to 89.90) | -0.02 (-0.13 to 0.10) |
| Depressive disorders | | | | | | | | | |
| Total | 0.43 (0.32 to 0.55) | 2,311.00 (1,664.48 to 3,153.76) | 0.34 (0.25 to 0.43) | 0.52 (0.37 to 0.71) | 2,806.44 (1,881.07 to 4,059.24) | 0.50 (0.41 to 0.61) | 0.08 (0.05 to 0.11) | 418.72 (251.61 to 640.93) | 0.42 (0.33 to 0.52) |
| Female | 0.25 (0.18 to 0.32) | 2,747.47 (1,933.57 to 3,808.66) | 0.35 (0.26 to 0.46) | 0.30 (0.21 to 0.42) | 3,413.83 (2,226.14 to 5,075.45) | 0.52 (0.42 to 0.63) | 0.04 (0.03 to 0.07) | 502.13 (295.35 to 778.59) | 0.44 (0.34 to 0.55) |
| Male | 0.18 (0.14 to 0.23) | 1,899.55 (1,391.62 to 2,533.78) | 0.31 (0.23 to 0.41) | 0.21 (0.16 to 0.28) | 2,234.01 (1,553.95 to 3,102.76) | 0.48 (0.39 to 0.59) | 0.03 (0.02 to 0.05) | 340.10 (209.88 to 516.48) | 0.39 (0.30 to 0.51) |
| Bipolar disorder | | | | | | | | | |
| Total | 0.08 (0.05 to 0.11) | 408.60 (279.69 to 591.95) | -0.01 (-0.02 to 0.01) | 0.01 (0.01 to 0.02) | 65.82 (40.85 to 99.68) | 0.00 (0.00 to 0.01) | 0.02 0.01 to 0.03) | 91.45 (53.03 to 148.16) | -0.01 (-0.06 to 0.03) |
| Female | 0.04 (0.03 to 0.06) | 436.48 (298.55 to 630.82) | -0.01 (-0.02 to -0.01) | 0.01 (0.00 to 0.01) | 69.95 (43.60 to 106.22) | 0.00 (0.00 to 0.01) | 0.01 (0.01 to 0.01) | 97.15 (55.49 to 155.33) | -0.01 (-0.07 to 0.05) |
| Male | 0.04 (0.03 to 0.05) | 382.30 (260.34 to 555.38) | -0.01 (-0.02 to -0.01) | 0.01 (0.00 to 0.01) | 61.93 (38.31 to 93.77) | 0.00 (0.00 to 0.01) | 0.01 (0.00 to 0.01) | 86.0 (49.23 to 141.42) | -0.01 (-0.08 to 0.06) |
| Anxiety disorders | | | | | | | | | |
| Total | 0.92 (0.67 to 1,21) | 5,045.72 (3,564.90 to 7,016.18) | 0.43 (0.33 to 0.53) | 0.16 (0.11 to 0.22) | 901.24 (572.93 to 1,296.41) | 0.44 (0.34 to 0.54) | 0.11 (0.07 to 0.17) | 622.22 (373.69 to 938.28) | 0.43 (0.33 to 0.53) |
| Female | 0.57 (0.42 to 0.75) | 6,439.02 (4,528.84 to 8,899.57) | 0.44 (0.34 to 0.54) | 0.10 (0.07 to 0.14) | 1,113.04 (708.56 to 1,600.72) | 0.45 (0.35 to 0.55) | 0.07 (0.04 to 0.10) | 791.36 (479.89 to 1,184.68) | 0.44 (0.34 to 0.54) |
| Male | 0.35 (0.25 to 0.46) | 3,733.54 (2,602.88 to 5,211.83) | 0.42 (0.31 to 0.52) | 0.07 (0.05 to 0.09) | 701.85 (445.56 to 1,002.36) | 0.43 (0.32 to 0.53) | 0.04 (0.03 to 0.07) | 462.94 (272.94 to 706.40) | 0.41 (0.30 to 0.53) |
| Eating disorders | | | | | | | | | |
| Total | 0.07 (0.05 to 0.10) | 370.65 (235.57 to 574.90) | 0.00 (-0.03 to 0.02) | 0.07 (0.05 to 0.11) | 395.26 (215.26 to 680.50) | 0.00 (-0.02 to 0.02) | 0.01 (0.01 to 0.02) | 79.73 (42.57 to 134.39) | -0.01 (-0.05 to 0.03) |
| Female | 0.04 (0.03 to 0.06) | 493.81 (322.60 to 743.22) | 0.00 (-0.03 to 0.03) | 0.03 (0.02 to 0.04) | 343.12 (200.85 to 557.17) | 0.01 (-0.02 to 0.03) | 0.01 (0.01 to 0.02) | 105.90 (58.26 to 175.08) | -0.01 (-0.05 to 0.04) |
| Male | 0.02 (0.02 to 0.04) | 254.52 (149.95 to 427.09) | -0.01 (-0.04 to 0.02) | 0.04 (0.03 to 0.07) | 444.43 (226.28 to 798.46) | 0.00 (-0.03 to 0.03) | 0.02 (0.00 to 0.01) | 55.05 (27.39 to 99.78) | -0.01 (-0.08 to 0.06) |
| Autism spectrum disorders | | | | | | | | | |
| Total | 0.18 (0.15 to 0.22) | 1,015.60 (852.40 to 1,198.24) | 0.00 (-0.02 to 0.02) | NA | NA | NA | 0.04 (0.02 to 0.05) | 194.40 (134.28 to 270.66) | 0.00 (-0.03 to 0.03) |
| Female | 0.06 (0.05 to 0.07) | 696.96 (581.55 to 826.02) | 0.00 (-0.03 to 0.02) | NA | NA | NA | 0.01 (0.01 to 0.02) | 132.86 (89.84 to 183.94) | 0.00 (-0.04 to 0.04) |
| Male | 0.12 (0.10 to 0.14) | 1,315.61 (1,105.97 to 1,551.29) | 0.00 (-0.03 to 0.02) | NA | NA | NA | 0.02 (0.02 to 0.03) | 252.35 (174.57 to 353.22) | 0.00 (-0.04 to 0.03) |
| Attention-deficit/hyperactivity disorder | | | | | | | | | |
| Total | 0.37 (0.26 to 0.53) | 2,063.04 (1,396.69 to 2,950.80) | 0.01 (0.00 to 0.01) | 0.00 (0.00 to 0.00) | 10.95 (7.39 to 16.16) | 0.02 (0.02 to 0.03) | 0.00 (0.00 to 0.01) | 25.24 (12.86 to 42.40) | 0.00 (-0.02 to 0.03) |
| Female | 0.11 (0.07 to 0.15) | 1,226.57 (822.44 to 1,763.51) | 0.01 (0.00 to 0.01) | 0.00 (0.00 to 0.00) | 6.58 (4.40 to 9.68) | 0.02 (0.02 to 0.03) | 0.00 (0.00 to 0.00) | 14.98 (7.55 to 25.21) | 0.00 (-0.05 to 0.06) |
| Male | 0.26 (0.18 to 0.38) | 2,850.23 (1,935.43 to 4,080.31) | 0.00 (0.00 to 0.01) | 0.00 (0.00 to 0.00) | 15.05 (10.35 to 22.12) | 0.02 (0.02 to 0.02) | 0.00 (0.00 to 0.01) | 34.90 (17.80 to 58.84) | 0.00 (-0.03 to 0.04) |
| Conduct disorder | | | | | | | | | |
| Total | 0.34 (0.24 to 0.45) | 1,947.54 (1,340.00 to 2,662.37) | 0.02 (0.02 to 0.02) | 0.09 (0.06 to 0.12) | 500.65 (302.68 to 704.23) | 0.02 (0.02 to 0.02) | 0.04 (0.02 to 0.07) | 238.00 (125.45 to 386.98) | 0.02 (-0.01 to 0.04) |
| Female | 0.13 (0.09 to 0.17) | 1,525.54 (1,001.59 to 2,176.76) | 0.02 (0.02 to 0.02) | 0.03 (0.02 to 0.05) | 396.65 (232.88 to 591.05) | 0.02 (0.02 to 0.03) | 0.02 (0.01 to 0.03) | 185.75 (95.00 to 310.85) | 0.02 (-0.02 to 0.06) |
| Male | 0.21 (0.15 to 0.28) | 2,344.26 (1,633.81 to 3,165.10) | 0.02 (0.02 to 0.02) | 0.05 (0.04 to 0.07) | 598.31 (362.42 to 838.52) | 0.02 (0.02 to 0.02) | 0.03 (0.01 to 0.04) | 287.12 (153.41 to 464.08) | 0.02 (-0.01 to 0.05) |
| Idiopathic developmental intellectual disability | | | | | | | | | |
| Total | 0.07 (0.01 to 0.13) | 387.14 (57.80 to 715.45) | -0.02 (-0.06 to 0.00) | NA | NA | NA | 0.00 (0.00 to 0.01) | 17.32 (2.88 to 36.44) | -0.01 (-0.08 to 0.03) |
| Female | 0.03 (0.01 to 0.05) | 355.37 (67.95 to 624.77) | -0.02 (-0.06 to 0.01) | NA | NA | NA | 0.00 (0.00 to 0.00) | 15.77 (3.25 to 32.43) | -0.02 (-0.09 to 0.06) |
| Male | 0.04 (0.01 to 0.07) | 417.05 (56.31 to 794.82) | -0.03 (-0.08 to 0.01) | NA | NA | NA | 0.00 (0.00 to 0.00) | 18.78 (2.65 to 40.19) | -0.03 (-0.10 to 0.04) |
| Other mental disorders | | | | | | | | | |
| Total | 0.09 (0.06 to 0.13) | 489.15 (313.67 to 694.56) | -0.02 (-0.02 to -0.02) | NA | NA | NA | 0.01 (0.00 to 0.01) | 37.66 (21.65 to 59.84) | -0.02 (-0.09 to 0.03) |
| Female | 0.03 (0.02 to 0.05) | 378.80 (240.93 to 535.34) | -0.02 (-0.02 to -0.02) | NA | NA | NA | 0.00 (0.00 to 0.00) | 28.93 (15.80 to 46.28) | -0.03 (-0.12 to 0.06) |
| Male | 0.06 (0.04 to 0.08) | 593.29 (380.55 to 853.16) | -0.02 (-0.02 to -0.02) | NA | NA | NA | 0.00 (0.00 to 0.01) | 45.90 (26.71 to 73.03) | -0.02 (-0.10 to 0.06) |

**Table S7.** Prevalence, Incidence, and Years Lived with Disability of Mental Disorders Among Adolescents and Young Adults (Aged 10-24) in **Central Latin America** in 2021, and Percentage Change from 2019 to 2021 According to GBD.

|  | Prevalence (95% uncertainty interval) | | | Incidence (95% uncertainty interval) | | | Years lived with disability (95% uncertainty interval) | | |
| --- | --- | --- | --- | --- | --- | --- | --- | --- | --- |
| Cause | Counts in million | Age-standardized rates per 100,000 people | Percentage rate change (2019-2021) | Counts in million | Age-standardized rates per 100,000 people | Percentage rate change (2019-2021) | Counts in million | Age-standardized rates per 100,000 people | Percentage rate change (2019-2021) |
| Mental disorders | | | | | | | | | |
| Total | 10.08 (8.91 to 11.38) | 15,438.76 (13,230.81 to 17,875.02) | 0.14 (0.10 to 0.18) | 3.85 (3.14 to 4.73) | 5,856.86 (4,509.51 to 7,438.26) | 0.28 (0.23 to 0.34) | 1.36 (1.00 to 1.82) | 2,069.11 (1,471.65 to 2,788.37) | 0.17 (0.13 to 0.22) |
| Female | 5.22 (4.57 to 5.94) | 16,022.78 (13,543.65 to 18,813.15) | 0.18 (0.14 to 0.22) | 2.36 (1.89 to 3.00) | 7,185.02 (5,480.19 to 9,329.97) | 0.31 (0.26 to 0.37) | 0.76 (0.55 to 1.03) | 2,324.03 (1,622.94 to 3,179.18) | 0.21 (0.16 to 0.26) |
| Male | 4.86 (4.26 to 5.47) | 14,841.11 (12,771.35 to 17,019.00) | 0.09 (0.07 to 0.13) | 1.49 (1.24 to 1.78) | 4,534.47 (3,524.59 to 5,704.26) | 0.24 (0.18 to 0.29) | 0.60 (0.44 to 0.80) | 1,814.37 (1,307.17 to 2,427.71) | 0.13 (0.09 to 0.18) |
| Schizophrenia | | | | | | | | | |
| Total | 0.06 (0.04 to 0.09) | 92.80 (57.42 to 139.65) | 0.00 (-0.02 to 0.03) | 0.01 (0.01 to 0.02) | 21.98 (12.46 to 34.26) | 0.00 (-0.02 to 0.02) | 0.04 (0.03 to 0.07) | 62.04 (36.92 to 99.16) | 0.00 (-0.07 to 0.08) |
| Female | 0.03 (0.02 to 0.04) | 81.94 (50.37 to 124.03) | 0.00 (-0.04 to 0.03) | 0.01 (0.00 to 0.01) | 19.49 (11.05 to 30.47) | 0.00 (-0.04 to 0.03) | 0.02 (0.01 to 003) | 54.13 (32.23 to 88.14) | -0.01 (-0.11 to 0.11) |
| Male | 0.04 (0.02 to 0.05) | 103.69 (64.31 to 155.35) | 0.01 (-0.03 to 0.04) | 0.01 (0.01 to 0.01) | 24.46 (13.86 to 37.89) | 0.01 (-0.03 to 0.04) | 0.02 (0.01 to 0.04) | 69.97 (41.28 to 111.98) | 0.00 (-0.09 to 0.11) |
| Depressive disorders | | | | | | | | | |
| Total | 1.84 (1.41 to 2.42) | 2,755.63 (1,996.27 to 3,766.72) | 0.30 (0.24 to 0.37 | 2.47 (1.81 to 3.34) | 3,709.68 (2,589.55 to 5,227.17) | 0.38 (0.32 to 0.45) | 0.35 (0.22 to 0.52) | 525.51 (325.19 to 801.34) | 0.34 (0.28 to 0.42) |
| Female | 1.18 (0.90 to 1.56 | 3,546.11 (2,549.77 to 4,910.95) | 0.32 (0..26 to 0.39) | 1.64 (1.20 to 2.22) | 4,939.87 (3,420.21 to 7,005.30) | 0.39 (0.32 to 0.46) | 0.23 (0.14 to 0.34) | 686.67 (420.60 to 1,052.71) | 0.36 (0.28 to 0.43) |
| Male | 0.66 (0.51 to 0.87) | 1,968.43 (1,448.32 to 2,627.74) | 0.27 (0.20 to 0.35) | 0.83 (0.61 to 1.12) | 2,486.39 (1,726.73 to 3,518.33) | 0.36 (0.28 to 0.46) | 0.12 (0.08 to 0.18) | 365.06 (227.77 to 557.77) | 0.32 (0.23 to 0.41) |
| Bipolar disorder | | | | | | | | | |
| Total | 0.55 (0.41 to 0.73) | 818.83 (594.96 to 1,108.50) | 0.00 (0.00 to 0.00) | 0.08 (0.06 to 0.11) | 125.51 (83.68 to 181.89) | 0.00 (0.00 to 0.00) | 0.12 (0.08 to 0.19) | 182.83 (110.71 to 284.87) | 0.00 (-0.03 to 0.03) |
| Female | 0.30 (0.22 to 0.40) | 903.46 (658.04 to 1,213.38) | 0.00 (0.00 to 0.00) | 0.04 (0.03 to 0.06) | 138.04 (92.21 to 197.42) | 0.00 (0.00 to 0.00) | 0.07 (0.04 to 0.10) | 200.51 (120.67 to 309.57) | 0.00 (-0.04 to 0.04) |
| Male | 02.5 (0.18 to 0.33) | 734.52 (531.95 to 1,009.48) | 0.00 (0.00 to 0.00) | 0.04 (0.03 to 0.05) | 113.22 (74.88 to 165.24) | 0.00 (0.00 to 0.00) | 0.06 (0.03 to 0.09) | 165.23 (100.48 to 261.22) | 0.00 (-0.04 to 0.04) |
| Anxiety disorders | | | | | | | | | |
| Total | 3.87 (2.90 to 5.00) | 5,909.71 (4,231.18 to 8,082.95) | 0.31 (0.22 to 0.42) | 0.67 (0.47 to 0.91) | 1,035.00 (671.16 to 1,448.27) | 0.32 (0.23 to 0.43) | 0.48 (0.30 to 0.70) | 727.42 (444.58 to 1,103.72) | 0.31 (0.22 to 0.42) |
| Female | 2.41 (1.82 to 3.10) | 7,389.36 (5,290.64 to 9,960.45) | 0.32 (0.23 to 0.42) | 0.41 (0.29 to 0.56) | 1,271.14 (828.64 to 1,779.02) | 0.33 (0.24 to 0.44) | 0.29 (0.18 to 0.43) | 905.68 (557.04 to 1,367.01) | 0.32 (0.22 to 0.43) |
| Male | 1.46 (1.08 to 1.92) | 4,444.36 (3,136.35 to 6,144.30) | 0.30 (0.20 to 0.42) | 0.26 (0.19 to 0.35) | 801.70 (516.58 to 1,119.71) | 0.31 (0.21 to 0.42) | 0.18 (0.11 to 0.27) | 550.95 (331.16 to 848.44) | 0.30 (0.19 to 0.42) |
| Eating disorders | | | | | | | | | |
| Total | 0.30 (0.21 to 0.45) | 451.41 (278.60 to 703.56) | -0.01 (-0.03 to 0.02) | 0.30 (0.18 to 0.44) | 448.63 (243.11 to 772.51) | -0.01 (-0.03 to 0.01) | 0.06 (0.04 to 0.10) | 96.68 (50.42 to 166.09) | -0.01 (-0.05 to 0.03) |
| Female | 0..21 (0.14 to 0.31) | 618.90 (382.97 to 951.62) | -0.01 (-0.03 to 0.02) | 0.14 (0.09 to 0.19) | 417.23 (237.23 to 684.83) | -0.02 (-0.05 to 0.01) | 0.04 (0.02 to 0.07) | 132.02 (69.47 to 224.18) | -0.01 (-0.06 to 0.04) |
| Male | 0.09 (0.06 to 0.14) | 283.73 (167.50 to 468.70) | -0.01 (-0.04 to 0.02) | 0.16 (0.10 to 0.25) | 479.45 (243.42 to 863.69) | 0.01 (-0.03 to 0.03) | 0.02 (0.01 to 0.03) | 61.31 (31.10 to 109.33) | -0.01 (-0.07 to 0.06) |
| Autism spectrum disorders | | | | | | | | | |
| Total | 0.53 (0.44 to 0.62) | 810.76 (682.20 to 957.40) | 0.00 (-0.02 to 0.02) | NA | NA | NA | 0.10 (0.07 to 0.14) | 155.06 (105.19 to 219.68) | 0.00 (-0.03 to 0.03) |
| Female | 0.18 (0.15 to 0.21) | 543.28 (452.60 to 646.92) | 0.00 (-0.02 to 0.03) | NA | NA | NA | 0.03 (0.02 to 0.05) | 103.32 (71.23 to 146.66) | 0.00 (-0.05 to 0.05) |
| Male | 0.35 (0.30 to 0.41) | 1,074.50 (906.06 to 1,263.10) | 0.00 (-0.02 to 0.03) | NA | NA | NA | 0.07 (0.05 to 0.10) | 206.08 (139.10 to 291.16) | 0.00 (-0.04 to 0.04) |
| Attention-deficit/hyperactivity disorder | | | | | | | | | |
| Total | 1.85 (1.30 to 2.65) | 2,878.27 (1,976.42 to 4,087.20) | -0.01 (-0.01 to -0.01) | 0.01 (0.01 to 0.01) | 15.39 (10.59 to 22.43) | 0.00 (-0.01 to 0.00) | 0.02 (0.01 to 0.04) | 35.18 (18.58 to 59.08) | -0.01 (-0.03 to 0.02) |
| Female | 0.59 (0.41 to 0.83) | 1,833.90 (1,247.66 to 2,626.84) | -0.01 (-0.01 to -0.01) | 0.00 (0.00 to 0.00) | 10.03 (6.74 to 14.85) | 0.00 (-0.01 to 0.00) | 0.01 (0.00 to 0.01) | 22.34 (11.56 to 37.53) | -0.01 (-0.05 to 0.04) |
| Male | 1.27 (0.89 to 1.79) | 3,904.57 (2,685.62 to 5,532.93) | -0.01 (-0.01 to 0.00) | 0.01 (0.00 to 0.01) | 20.58 (14.26 to 29.58) | 0.00 (-0.01 to 0.00) | 0.02 (0.01 to 0.03) | 47.80 (25.16 to 80.47) | -0.01 (-0.04 to 0.02) |
| Conduct disorder | | | | | | | | | |
| Total | 1.20 (0.85 to 1.57) | 1,912.39 (1,321.07 to 2,626.99) | 0.00 (0.00 to 0.00) | 0.31 (0.20 to 0.42) | 500.67 (307.33 to 709.18) | 0.00 (0.00 to 0.00) | 0.15 (0.08 to 0.23) | 233.50 (122.24 to 380.78) | 0.00 (-0.02 to 0.02) |
| Female | 04.5 (0.30 to 0.61) | 1,460.43 (946.53 to 2,097.62) | 0.00 (0.00 to 0.00) | 0.12 (0.07 to 0.17) | 389.24 (224.22 to 586.57) | 0.00 (0.00 to 0.01) | 0.05 (0.03 to 0.09) | 177.41 (91.07 to 295.83) | 0.00 (-0.03 to 0.03) |
| Male | 0.75 (0.54 to 0..95) | 2,352.89 (1,654.55 to 3,143.08) | 0.00 (0.00 to 0.00) | 0.19 (0.13 to 0.26) | 608.68 (371.28 to 859.07) | 0.00 (0.00 to 0.00) | 0.09 (0.05 to 0.14) | 288.17 (154.36 to 465.28) | 0.00 (-0.03 to 0.03) |
| Idiopathic developmental intellectual disability | | | | | | | | | |
| Total | 0.23 (0.04 to 0.42) | 352.46 (59.90 to 642.49) | 0.01 (-0.03 to 0.04) | NA | NA | NA | 0.01 (0.00 to 0.02) | 15.20 (2.50 to 32.01) | 0.00 (-0.05 to 0.05) |
| Female | 0.11 (0.03 to 0.19) | 336.12 (78.70 to 584.17) | 0.01 (-0.03 to 0.05) | NA | NA | NA | 0.00 (0.00 to 0.01) | 14.27 (3.11 to 29.06) | 0.00 (-0.06 to 0.07) |
| Male | 0.12 (0.01 to 0.23) | 368.52 (45.26 to 697.31) | 0.01 (-0.04 to 0.04) | NA | NA | NA | 0.01 (0.00 to 0.01) | 16.13 (2.01 to 34.99) | 0.00 (-0.08 to 0.09) |
| Other mental disorders | | | | | | | | | |
| Total | 0.31 (0.20 to 0.45) | 463.56 (296.09 to 663.06) | 0.00 (0.00 to 0.00) | NA | NA | NA | 0.02 (0.01 to 0.04) | 35.68 (20.27 to 56.53) | 0.00 (-0.06 to 0.06) |
| Female | 0.12 (0.08 to 0.17) | 362.52 (230.30 to 514.35) | 0.00 (-0.01 to 0.00) | NA | NA | NA | 0.01 (0.00 to 0.02) | 27.69 (14.65 to 44.99) | -0.01 (-0.10 to 0.09) |
| Male | 0.19 (0.12 to 0.28) | 564.82 (362.34 to 812.78) | 0.00 (0.00 to 0.00) | NA | NA | NA | 0.01 (0.01 to 0.02) | 43.69 (25.51 to 68.88) | 0.00 (-0.08 to 0.08) |

**Table S8**. Prevalence, Incidence, and Years Lived with Disability of Mental Disorders Among Adolescents and Young Adults (Aged 10-24) in **Central Sub−Saharan Africa** in 2021, and Percentage Change from 2019 to 2021 According to GBD.

|  | Prevalence (95% uncertainty interval) | | | Incidence (95% uncertainty interval) | | | Years lived with disability (95% uncertainty interval) | | |
| --- | --- | --- | --- | --- | --- | --- | --- | --- | --- |
| Cause | Counts in million | Age-standardized rates per 100,000 people | Percentage rate change (2019-2021) | Counts in million | Age-standardized rates per 100,000 people | Percentage rate change (2019-2021) | Counts in million | Age-standardized rates per 100,000 people | Percentage rate change (2019-2021) |
| Mental disorders | | | | | | | | | |
| Total | 6.34 (5.42 to 7.35) | 14,302.08 (11,930.96 to 17,092.46) | 0.07 (0.01 to 0.14) | 3.46 (2.51 to 4.65) | 7,913.31 (5,522.41 to 11,130.48) | 0.13 (0.00 0.27) | 0.93 (0.66 to 1.27) | 2,112.47 (1,482.30 to 2,937.82) | 0.09 (0.01 to 0.17) |
| Female | 3.19 (2.70 to 3.75) | 14,531.30 (11,870.34 to 17,735.45) | 0.08 (0.01 to 0.16) | 1.91 (1.37 to 2.60) | 8,819.02 (6,097.87 to 12,599.09) | 0.14 (0.00 to 0.29) | 0.48 (0.34 to 0.67) | 2,223.19 (1,543.17 to 3,127.89) | 0.10 (0.01 to 0.20) |
| Male | 3.15 (2.73 to 3.59) | 14,071.46 (11,893.31 to 16,556.56) | 0.05 (0.00 to 0.12) | 1.54 (1.13 to 2.07) | 7,014.35 (4,895.03 to 9,773.92) | 0.11 (-0.02 to 0.26) | 0.44 (0.32 to 0.61) | 2,002.34 (1,402.57 to 2,772.90) | 0.07 (-0.01 to 0.17) |
| Schizophrenia | | | | | | | | | |
| Total | 0.03 (0.02 to 0.05) | 79.04 (44.78 to 132.86) | 0.01 (-0.05 to 0.06) | 0.01 (0.01 to 0.01) | 19.66 (10.22 to 32.93) | 0.00 (-0.06 to 0.06) | 0.02 (0.01 to 0.04) | 52.27 (26.53 to 91.40) | 0.00 (-0.20 to 0.23) |
| Female | 0.02 (0.01 to 0.03) | 79.96 (45.35 to 132.82) | 0.00 (-0.09 to 0.07) | 0.00 (0.00 to 0.01) | 20.16 (10.56 to 33.81) | 0.00 (-0.09 to 0.08) | 0.01 (0.01 to 0.02) | 52.39 (26.30 to 92.93) | -0.01 (-0.27 to 0.31) |
| Male | 0.02 (0.01 to 0.02) | 78.13 (44.15 to 129.33) | 0.01 (-0.06 to 0.10) | 0.00 (0.00 to 0.01) | 19.15 (9.91 to 32.28) | 0.01 (-0.05 to 0.09) | 0.01 (0.01 to 0.02) | 52.14 (25.08 to 93.83) | 0.00 (-0.26 to 0.41) |
| Depressive disorders | | | | | | | | | |
| Total | 1.98 (1.41 to 2.78) | 4,624.11 (3,118.97 to 6,681.93) | 0.12 (-0.02 to 0.27) | 2.70 (1.77 to 3.90) | 6,271.44 (3,933.70 to 9,486.15) | 0.14 (-0.03 to 0.32) | 0.38 (0.22 to 0.57) | 879.43 (506.63 to 1,393.32) | 0.13 (-0.02 to 0.30) |
| Female | 1.15 (0.81 to 1.60) | 5,373.22 (3,627.41 to 7,728.99) | 0.12 (-0.02 to 0.27) | 1.56 (1.03 to 2.25) | 7,261.68 (4,563.98 to 10,957.68) | 0.15 (-0.02 to0.34) | 0.22 (0.13 to 0.33) | 1,013.78 (576.44 to 1,612.33) | 0.13 (-0.01 to 0.31) |
| Male | 0.83 (0.58 to 1.18) | 3,880.57 (2,580.30 to 5,689.21) | 0.11 (-0.04 to 0.29) | 1.14 (0.75 to 1.66) | 5,289.23 (3,337.90 to 8,020.08) | 0.14 (-0.05 to0.34) | 0.16 (0.09 to 0.25) | 746.12 (423.50 to 1,204.03) | 0.13 (-0.06 to 0.32) |
| Bipolar disorder | | | | | | | | | |
| Total | 0.17 (0.11 to 0.26) | 410.06 (255.14 to 632.32) | 0.00 (0.00 to 0.00) | 0.03 (0.02 to 0.04) | 66.24 (37.42 to 106.94) | 0.00 (0.00 to 0.00) | 0.04 (0.02 to 0.07) | 90.69 (46.18 to 156.11) | 0.00 (-0.10 to 0.12) |
| Female | 0.09 (0.06 to 0.14) | 421.61 (262.12 to 651.24) | 0.00 (0.00 to 0.00) | 0.02 (0.01 to 0.02) | 67.93 (38.31 to 109.98) | 0.00 (0.00 to 0.00) | 0.02 (0.01 to 0.03) | 92.54 (47.84 to 160.28) | 0.00 (-0.14 to 0.19) |
| Male | 0.09 (0.06 to 0.13) | 398.59 (248.09 to 619.77) | 0.00 (0.00 to 0.00) | 0.01 (0.01 to 0.02) | 64.56 (36.44 to 104.61) | 0.00 (0.00 to 0.00) | 0.02 (0.01 to 0.03) | 88.85 (44.36 to 152.36) | 0.00 (-0.15 to 0.18) |
| Anxiety disorders | | | | | | | | | |
| Total | 2.03 (1.38 to 2.93) | 4,566.35 (2,999.87 to 6,800.69) | 0.14 (-0.04 to 0.34) | 0.37 (0.25 to 0.54) | 819.03 (490.49 to 1,258.18) | 0.00 (0.00 to 0.00) | 0.25 (.015 to 0.39) | 555.61 (320.92 to 895.92) | 0.13 (-0.05 to 0.34) |
| Female | 1.18 (0.80 to 1.71) | 5,341.91 (3,477.79 to 7,932.45) | 0.14 (-0.04 to 0.35) | 0.20 (0.14 to 0.30) | 903.18 (544.03 to 1,383.26) | 0.14 (-0.04 to 0.36) | 0.14 (0.08 to 0.23) | 646.64 (368.38 to 1,041.84) | 0.14 (-0.05 to 0.35) |
| Male | 0.85 (0.58 to 1.23) | 3,798.43 (2,468.95 to 5,673.65) | 0.13 (-0.06 to 0.36) | 0.17 (0.11 to 0.24) | 735.81 (441.73 to 1,128.79) | 0.13 (-0.06 to0.37) | 0.10 (.06 to 0.16) | 465.49 (264.36 to 761.35) | 0.13 (-0.08 to 0.35) |
| Eating disorders | | | | | | | | | |
| Total | 0.09 (0.06 to 0.13) | 211.70 (131.35 to 333.47) | 0.00 (-0.05 to 0.05) | 0.11 (0.07 to 0.17) | 256.96 (138.30 to 436.08) | 0.00 (-0.05 to 0.05) | 0.02 (0.01 to 0.03) | 45.05 (22.82 to 78.22) | 0.00 (-0.12 to 0.14) |
| Female | 0.05 (0.04 to 0.08) | 256.20 (163.32 to 395.25) | 0.00 (-0.06 to 0.08) | 0.04 (0.03 to 0.06) | 201.39 (117.47 to 324.90) | 0.00 (-0.07 to 0.07) | 0.01 (0.01 to 0.02) | 54.09 (27.36 to 91.36) | 0.00 (-0.15 to 0.18) |
| Male | 0.04 (0.02 to 0.06) | 167.49 (98.20 to 283.44) | 0.00 (-0.06 to 0.08) | 0.07 (0.05 to 0.11) | 312.05 (158.03 to 556.67) | -0.01 (-0.08 to 0.07) | 0.01 (0.00 to 0.01) | 36.07 (16.68 to 66.85) | 0.00 (-0.19 to 0.22) |
| Autism spectrum disorders | | | | | | | | | |
| Total | 0.43 (0.36 to 0.51) | 957.88 (801.62 to 1,133.76) | 0.00 (-0.05 to 0.06) | NA | NA | NA | 0.08 (0.06 to 0.11) | 181.31 (122.92 to 256.96) | 0.00 (-0.08 to 0.08) |
| Female | 0.15 (0.13 to 0.18) | 679.35 (564.72 to 809.23) | 0.01 (-0.06 to 0.09) | NA | NA | NA | 0.03 (0.02 to 0.04) | 128.07 (85.56 to 183.78) | 0.01 (-0.10 to 0.14) |
| Male | 0.28 (0.23 to 0.33) | 1,233.58 (1,034.11 to 1,461.31) | 0.00 (-0.08 to 0.07) | NA | NA | NA | 0.05 (0.04 to 0.07) | 234.01 (157.67 to 329.89) | 0.00 (-0.11 to 0.10) |
| Attention-deficit/hyperactivity disorder | | | | | | | | | |
| Total | 0.47 (0.32 to 0.67) | 1,020.31 (680.46 to 1,488.79) | 0.00 (0.00 to 0.00) | 0.00 (0.00 to 0.00) | 5.72 (3.78 to 8.44) | 0.00 (0.00 to 0.00) | 0.01 (0.00 to 0.01) | 12.40 (6.24 to 21.42) | 0.00 (-0.10 to 0.10) |
| Female | 0.14 (0.09 to 0.20) | 593.98 (389.53 to 888.08) | 0.00 (0.00 to 0.00) | 0.00 (0.00 to 0.00) | 3.34 (2.17 to 5.13) | -0.01 (-0.01 to -0.01) | 0.00 (0.00 to 0.00) | 7.18 (3.41 to 13.13) | -0.01 (-0.19 to 0.23) |
| Male | 0.33 (0.23 to 0.49) | 1,441.76 (961.52 to 2,104.29) | 0.00 (0.00 to 0.00) | 0.00 (0.00 to 0.00) | 8.07 (5.32 to 11.94) | -0.01 (-0.01 to -0.01) | 0.00 (0.00 to 0.01) | 17.55 (8.50 to 30.48) | 0.00 (-0.12 to 0.13) |
| Conduct disorder | | | | | | | | | |
| Total | 0.89 (0.61 to 1.16) | 1,855.17 (1,252.59 to 2,560.24) | 0.00 (0.00 to 0.00) | 0.23 (0.15 to 0.32) | 474.27 (283.70 to 683.94) | 0.00 (0.00 to 0.00) | 0.11 (0.06 to 0.17) | 224.39 (116.33 to 366.21) | 0.00 (-0.06 to 0.06) |
| Female | 0.33 (0.22 to 0.45) | 1,406.33 (900.35 to 2,052.55) | 0.00 (0.00 to 0.00) | 0.09 (0.05 to 0.13) | 361.34 (206.13 to 554.73) | 0.00 (0.00 to 0.00) | 0.04 (0.02 to 0.07) | 169.51 (84.12 to 288.11) | 0.00 (-0.09 to 0.10) |
| Male | 0.55 (0.40 to 0.71) | 2,297.86 (1,582.58 to 3,116.07) | 0.00 (0.00 to 0.00) | 01.4 (0.09 to 0.19) | 585.48 (348.60 to 827.77) | 0.00 (0.00 to 0.00) | 0.07 (0.04 to 0.11) | 278.51 (143.26 to 449.27) | 0.00 (-0.07 to 0.07) |
| Idiopathic developmental intellectual disability | | | | | | | | | |
| Total | 0.41 (0.16 to 0.64) | 909.31 (365.87 to 1,414.53) | 0.00 (-0.24 to 0.30) | NA | NA | NA | 0.01 (0.00 to 0.03) | 31.07 (8.21 to 62.83) | 0.00 (-0.12 to 0.12) |
| Female | 0.19 (0.08 to 1.71) | 823.35 (344.82 to 1,263.44) | 0.00 (-0.23 to 0.29) | NA | NA | NA | 0.01 (0.00 to 0.01) | 28.06 (8.07 to 55.37) | 0.00 (-0.15 to 0.14) |
| Male | 0.23 (0.09 to 0.36) | 994.36 (385.90 to 1,568.46) | 0.00 (-0.25 to 0.31) | NA | NA | NA | 0.01 (0.00 to 0.02) | 34.05 (7.76 to 69.64) | 0.00 (-0.13 to 0.17) |
| Other mental disorders | | | | | | | | | |
| Total | 0.22 (01.4 to 0.30) | 526.03 (337.13 to 728.08) | 0.00 (0.00 to 0.00) | NA | NA | NA | 0.02 (0.01 to 0.03) | 40.25 (23.05 to 64.17) | 0.00 (-0.14 to 0.15) |
| Female | 0.08 (0.05 to 0.12) | 408.20 (259.10 to 576.09) | 0.00 (0.00 to 0.00) | NA | NA | NA | 0.01 (0.00 ot 0.01) | 30.92 (16.24 to 51.99) | 0.00 (-0.20 to 0.24) |
| Male | 0.13 (0.09 to 0.19) | 643.24 (409.75 to 899.04) | 0.00 (0.00 to 0.00) | NA | NA | NA | 0.01 (0.01 to 0.02) | 49.54 (27.75 to 79.48) | 0.01 (-0.17 to 0.20) |

**Table S9.** Prevalence, Incidence, and Years Lived with Disability of Mental Disorders Among Adolescents and Young Adults (Aged 10-24) in **East Asia** in 2021, and Percentage Change from 2019 to 2021 According to GBD.

|  | Prevalence (95% uncertainty interval) | | | Incidence (95% uncertainty interval) | | | Years lived with disability (95% uncertainty interval) | | |
| --- | --- | --- | --- | --- | --- | --- | --- | --- | --- |
| Cause | Counts in million | Age-standardized rates per 100,000 people | Percentage rate change (2019-2021) | Counts in million | Age-standardized rates per 100,000 people | Percentage rate change (2019-2021) | Counts in million | Age-standardized rates per 100,000 people | Percentage rate change (2019-2021) |
| Mental disorders | | | | | | | | | |
| Total | 31.52 (27.36 to 35.87) | 12,975.41 (11,038.82 to 15,026.73) | 0.01 (0.00 to 0.07) | 7.10 (5.96 to 8.28) | 2,921.88 (2,289.43 to 3,601.99) | 0.01 (-0.03 to 0.05) | 3.34 (2.43 to 4.37) | 1,374.55 (977.21 to 1,829.63) | 0.01 (-0.03 to 0.05)） |
| Female | 13.20 (11.46 to 15.01) | 11,642.25 (9,870.63 to 13,580.89) | 0.03 (-0.01 to 0.07) | 3.66 (3.05 to 4.32) | 3,225.07 (2,531.38 to 3,989.85) | -0.01 (-0.04 to 0.01) | 1.60 (1.15 to 2.13) | 1,410.80 (982.95 to 1,913.60) | -0.01 (-0.05 to 0.04) |
| Male | 18.33 (15.78 to 21.18) | 14,139.82 (12,002.60 to 16,570.68) | 0.03 (0.00 to 0.07) | 3.44 (2.90 to 4.00) | 2,656.21 (2,070.40 to 3,339.58) | 0.03 (0.00 to 0.06) | 1.74 (1.28 to 2.27) | 1,342.61 (972.49 to 1,769.81) | 0.03 (0.00 to 0.06) |
| Schizophrenia | | | | | | | | | |
| Total | 0.32 (0.23 to 0.43) | 133.31 (91.00 to 181.09) | -0.03 (-0.05 to -0.01) | 0.08 (0.06 to 0.10) | 31.59 (20.62 to 44.00) | -0.01 (-0.03 to 0.01) | 0.22 (0.14 to 0.32) | 90.65 (56.75 to 133.63) | -0.01 (-0.03 to 0.01) |
| Female | 0.15 (0.20 to 0.11) | 133.04 (90.74 to 180.09) | -0.03 (-0.06 to 0.00) | 0.04 (0.03 to 0.05) | 31.70 (20.56 to 44.04) | -0.01 (-0.03 to 0.02) | 0.10 (0.07 to 0.15) | 90.04 (56.18 to 132.60) | -0.01 (-0.03 to 0.02) |
| Male | 0.17 (0.13 to 0.23) | 133.54 (91.67 to 181.09) | -0.02 (-0.06 to 0.00) | 0.04 (0.03 to 0.05) | 31.49 (20.50 to 44.18) | -0.01 (-0.04 to 0.02) | 0.12 (0.08 to 0.17) | 91.19 (57.52 to 135.38) | -0.01 (-0.04 to 0.02) |
| Depressive disorders | | | | | | | | | |
| Total | 3.10 (2.48 to 3.88) | 1,277.98 (963.00 to 1,666.17) | -0.06 (-0.09 to -0.01) | 3.40 (2.55 to 4.43) | 1,402.55 (991.66 to 1,932.98) | -0.06 (-0.10 to -0.01) | 0.54 (0.35 to 0.78) | 220.82 (138.13 to 328.72) | -0.06 (-0.10 to -0.01) |
| Female | 1.86 (1.49 to 2.31) | 1,646.80 (1,244.06 to 2,135.78) | -0.07 (-0.11 to -0.03) | 2.04 (1.54 to 2.64) | 1,805.88 (1,276.38 to 2,482.97) | -0.08 (-0.13 to -0.03) | 0.32 (0.21 to 0.46) | 284.26 (177.66 to 421.02) | -0.08 (-0.13 to -0.03) |
| Male | 1.23 (0.97 to 1.57) | 954.81 (713.04 to 1,242.64) | -0.03 (-0.07 to 0.02) | 1.35 (1.01 to 1.78) | 1,049.37 (737.66 to 1,453.34) | -0.03 (-0.08 to 0.03) | 0.21 (0.14 to 0.31) | 165.25 (103.32 to 245.87) | -0.03 (-0.08 to 0.03) |
| Bipolar disorder | | | | | | | | | |
| Total | 0.35 (0.36 to 0.46) | 145.67 (106.69 to 193.82) | -0.02 (-0.02 to -0.01) | 0.06 (0.04 to 0.07) | 23.21 (15.40 to 33.32) | 0.01 (0.00 to 0.02) | 0.08 (0.05 to 0.12) | 32.94 (19.68 to 52.28) | 0.01 (0.00 to 0.02) |
| Female | 0.17 (0.13 to 0.22) | 150.25 (110.84 to 201.87) | -0.02 (-0.03 to -0.01) | 0.03 (0.02 to 0.04) | 24.04 (15.95 to 34.49) | 0.01 (0.00 to 0.03) | 0.04 (0.02 to 0.06) | 33.80 (20.23 to 53.59) | 0.01 (0.00 to 0.03) |
| Male | 0.18 (0.14 to 0.24) | 141.65 (103.17 to 189.01) | -0.01 (-0..02 to -0.01) | 0.03 (0.02 to 0.04) | 22.49 (14.89 to 32.27) | 0.01 (0.00 to 0.02) | 0.04 (0.02 to 0.06) | 32.18 (19.06 to 50.90) | 0.01 (0.00 to 0.02) |
| Anxiety disorders | | | | | | | | | |
| Total | 10.70 (8.35 to 13.40) | 4,410.63 (3,311.02 to 5,781.41) | 0.08 (0.00 to0.15) | 1.70 (1.27 to 2.23) | 698.84 (469.83 to 962.41) | 0.14 (0.04 to 0.24) | 1.33 (0.86 to 1.97) | 549.46 (341.39 to 817.85) | 0.14 (0.04 to 0.24) |
| Female | 6.29 (4.94 to 7.88) | 5,558.53 (4,185.42 to 7,280.65) | 0.08 (-0.01 to 0.16) | 0.99 (0.73 to 1.30) | 867.97 (581.58 to 1,202.80) | 0.14 (0.04 to 0.26) | 0.78 (0.50 to 1.16) | 691.06 (431.15 to 1,024.39) | 0.14 (0.04 to 0.26) |
| Male | 4.41 (3.43 to 5.58) | 3,406.00 (2,549.58 to 4,467.21) | 0.09 (0.01 to 0.16) | 0.72 (0.54 to 0.92) | 550.80 (371.60 to 759.89) | 0.13 (0.05 to 0.22) | 0.55 (0.35 to 0.82) | 425.54 (264.74 to 631.15) | 0.13 (0.05 to 0.22) |
| Eating disorders | | | | | | | | | |
| Total | 0.62 (0.91 to 0.42) | 256.30 (155.72 to 414.43) | 0.03 (0.01 to 0.05) | 0.85 (0.53 to 1.32) | 352.67 (185.86 to 621.15) | 0.04 (0.02 to 0.07) | 0.13 (0.07 to 0.22) | 55.65 (28.47 to 96.03) | 0.04 (0.02 to 0.07) |
| Female | 0.33 (0.47 to 0.23) | 290.84 (184.24 to 444.57) | 0.02 (0.00 to 0.05) | 0.28 (0.18 to 0.41) | 248.84 (143.10 to 413.18) | 0.03 (0.01 to 0.06) | 0.07 (0.04 to 0.12) | 62.99 (33.42 to 105.78) | 0.03 (0.01 to 0.06) |
| Male | 0.29 (0.19 to 0.46) | 226.05 (131.14 to 383.96) | 0.04 (0.01 to 0.07) | 0.57 (0.34 to 0.91) | 443.49 (221.47 to 798.23) | 0.05 (0.02 to 0.08) | 0.06 (0.03 to 0.11) | 49.21 (24.16 to 91.07) | 0.05 (0.02 to 0.08) |
| Autism spectrum disorders | | | | | | | | | |
| Total | 1.69 (1.41 to 2.01) | 696.39 (581.61 to 828.25) | 0.00 (-0.02 to 0.02) | NA | NA | NA | 0.33 (0.22 to 0.46) | 134.29 (91.37 to 189.89) | 0.00 (0.00 to 0.00) |
| Female | 0.39 (0.32 to 0.46) | 339.39 (279.28 to 409.75) | 0.00 (-0.02 to 0.02) | NA | NA | NA | 0.07 (0.05 to 0.10) | 65.19 (44.05 to 91.50) | 0.00 (0.00 to 0.00) |
| Male | 1.31 (1.09 to 1.55) | 1,008.94 (841.79 to 1,196.23) | 0.00 (-0.02 to 0.03) | NA | NA | NA | 0.25 (0.17 to 0.36) | 194.79 (133.33 to 274.86) | 0.00 (0.00 to 0.00) |
| Attention-deficit/hyperactivity disorder | | | | | | | | | |
| Total | 10.72 (7.63 to 14.86) | 4,406.96 (3,072.89 to 6,212.53) | 0.03 (-0.04 to 0.11) | 0.06 (0.04 to 0.09) | 25.42 (17.49 to 36.49) | 0.03 (-0.05 to 0.01) | 0.13 (0.07 to 0.21) | 54.12 (28.90 to 89.82) | 0.03 (-0.05 to 0.01) |
| Female | 2.75 (1.97 to 3.80) | 2,421.36 (1,683.13 to 3,429.36) | 0.02 (-0.08 to 0.11) | 0.02 (0.01 to 0.02) | 13.51 (9.19 to 19.71) | 0.03 (-0.08 to 0.12) | 0.03 (0.02 to 0.06) | 29.69 (15.82 to 50.15) | 0.03 (-0.08 to 0.12) |
| Male | 7.97 (5.75 to 11.02) | 6,143.60 (4,271.24 to 8,609.76) | 0.04 (-0.06 to 0.13) | 0.05 (0.03 to 0.07) | 35.86 (24.67 to 51.72) | 0.03 (-0.07 to 0.13) | 0.10 (0.05 to 0.16) | 75.48 (40.22 to 126.90) | 0.03 (-0.07 to 0.13) |
| Conduct disorder | | | | | | | | | |
| Total | 3.72 (2.58 to 4.89) | 1,527.90 (1,031.95 to 2,112.00) | 0.03 (0.03 to 0.03) | 0.95 (0.61 to 1.33) | 387.61 (225.28 to 563.35) | 0.03 (0.03 to 0.03) | 0.46 (0.25 to 0.73) | 188.05 (97.62 to 307.66) | 0.03 (0.03 to 0.03) |
| Female | 1.04 (0.68 to 1.49) | 916.66 (587.65 to 1,369.64) | 0.04 (0.03 to 0.04) | 0.27 (0.16 to 0.42) | 233.13 (127.99 to 380.31) | 0.04 (0.04 to 0.04) | 0.13 (0.07 to 0.21) | 112.57 (57.68 to 194.95) | 0.04 (0.04 to 0.04) |
| Male | 2.68 (1.87 to 3.46) | 2,061.32 (1,399.25 to 2,806.36) | 0.03 (0.02 to 0.03) | 0.69 (0.44 to 0.94) | 522.70 (309.06 to 750.74) | 0.03 (0.03 to 0.03) | 0.33 (0.18 to 0.52) | 253.92 (132.37 to 415.97) | 0.03 (0.03 to 0.03) |
| Idiopathic developmental intellectual disability | | | | | | | | | |
| Total | 0.92 (0.27 to 1.57) | 376.83 (111.99 to 647.49) | -0.02 (-0.06 to 0.01) | NA | NA | NA | 0.04 (0.01 to 0.08) | 16.39 (4.42 to 32.66) | -0.02 (-0.06 to 0.02) |
| Female | 0.43 (0.18 to 0.68) | 379.26 (154.50 to 601.30) | -0.01 (-0.06 to 0.03) | NA | NA | NA | 0.02 (0.01 to 0.03) | 16.25 (5.75 to 30.49) | -0.01 (-0.06 to 0.04) |
| Male | 0.49 (0.10 to 0.90) | 374.71 (75.97 to 692.68) | -0.02 (-0.09 to 0.01) | NA | NA | NA | 0.02 (0.00 to 0.04) | 16.51 (3.11 to 34.73) | -0.02 (-0.09 to 0.03) |
| Other mental disorders | | | | | | | | | |
| Total | 1.01 (0.63 to 1.44) | 414.13 (259.32 to 592.06) | -0.03 (-0.03 to -0.03) | NA | NA | NA | 0.08 (0.04 to 0.12) | 32.18 (17.61 to 51.60) | 0.00 (0.00 to 0.00) |
| Female | 0.37 (0.22 to 0.53) | 322.11 (194.94 to 463.67) | -0.04 (-0.04 to -0.04) | NA | NA | NA | 0.03 (0.01 to 0.05) | 24.95 (13.06 to 40.59) | 0.00 (0.00 to 0.00) |
| Male | 0.64 (0.40 to 0.91) | 495.03 (313.40 to 709.22) | -0.03 (-0.03 to -0.03) | NA | NA | NA | 0.05 (0.03 to 0.08) | 38.54 (21.60 to 61.46) | 0.00 (0.00 to 0.00) |

**Table S10**. Prevalence, Incidence, and Years Lived with Disability of Mental Disorders Among Adolescents and Young Adults (Aged 10-24) in **Eastern Europe** in 2021, and Percentage Change from 2019 to 2021 According to GBD.

|  | Prevalence (95% uncertainty interval) | | | Incidence (95% uncertainty interval) | | | Years lived with disability (95% uncertainty interval) | | |
| --- | --- | --- | --- | --- | --- | --- | --- | --- | --- |
| Cause | Counts in million | Age-standardized rates per 100,000 people | Percentage rate change (2019-2021) | Counts in million | Age-standardized rates per 100,000 people | Percentage rate change (2019-2021) | Counts in million | Age-standardized rates per 100,000 people | Percentage rate change (2019-2021) |
| Mental disorders | | | | | | | | | |
| Total | 4,72 (4.21 to 5.27) | 14,362.96 (12,447.51 to 16,408.70) | 0.19 (0.15 to 0.22) | 1.92 (1.55 to 2.35) | 5,877.69 (4,412.05 to 7,582.39) | 0.38 (0.32 to 0.45) | 0.64 (0.47 to 0.84) | 1,949.68 (1,381.57 to 2,605.75) | 0.24 (0.19 to 0.29) |
| Female | 2.33 (2.07 to 2.63) | 14,578.43 (12,537.71 to 16,889.10) | 0.24 (0.20 to 0.28) | 1.02 (0.82 to 1.25) | 6,409.80 (4,810.27 to 8,277.64) | 0.41 (0.35 to 0.48) | 0.33 (0.24 to 0.44) | 2,083.30 (1,449.64 to 2,806.53) | 0.27 (0.22 to 0.33) |
| Male | 2.39 (2.13 to 2.67) | 14,156.31 (12,332.71 to 16,172.78) | 0.14 (0.11 to 0.17) | 0.90 (0.74 to 1.10) | 5,371.17 (4,029.42 to 6,905.58) | 0.35 (0.29 to 0.43) | 0.31 (0.22 to 0.40) | 1,822.42 (1,298.62 to 2,428.68) | 0.20 (0.15 to 0.25) |
| Schizophrenia | | | | | | | | | |
| Total | 0.02 (0.02 to 0.03) | 74.72 (47.66 to 109.53) | -0.03 (-0.06 to -0.01) | 0.01 (0.00 to 0.01) | 18.60 (11.02 to 27.64) | -0.02 (-0.05 to 0.00) | 0.02 (0.01 to 0.02) | 49.93 (29.77 to 78.19) | -0.04 (-0.11 to 0.03) |
| Female | 0.01 (0.01 to 0.02) | 74.03 (47.16 to 109.46) | -0.04 (-0.07 to -0.01) | 0.00 (0.00 to 0.00) | 18.66 (11.06 to 27.74) | -0.02 (-0.05 to 0.01) | 0.01 (0.00 to 0.01) | 48.99 (28.67 to 77.62) | -0.05 (-0.14 to 0.05) |
| Male | 0.01 (0.01 to 0.02) | 75.38 (47.84 to 109.83) | -0.03 (-0.06 to 0.00) | 0.00 (0.00 to 0.00) | 18.54 (11.01 to 27.43) | -0.02 (-0.05 to 0.01) | 0.01 (0.01 to 0.01) | 50.82 (30.31 to 79.72) | -0.04 (-0.13 to 0.07) |
| Depressive disorders | | | | | | | | | |
| Total | 0.95 (0.73 to 1.24) | 2,940.22 (2,117.45 to 4,015.34) | 0.37 (0.29 to 0.46) | 1.25 (0.91 to 1.70) | 3,871.60 (2,589.06 to 5,441.53) | 0.50 (0.43 to 0.60) | 0.18 (0.12 to 0.26) | 554.53 (342.73 to 845.59) | 0.44 (0.35 to 0.53) |
| Female | 0.52 (0.39 to 0.68) | 3,278.29 (2,366.45 to 4,490.96) | 0.37 (0.29 to 0.46) | 0.68 (0.50 to 0.92) | 4,330.62 (2,908.54 to 6,084.43) | 0.51 (0.43 to 0.61) | 0.10 (0.06 to 0.14) | 616.20 (383.07 to 943.54) | 0.44 (0.35 to 0.53) |
| Male | 0.43 (0.33 to 0.57) | 2,618.38 (1,876.48 to 3,555.85) | 0.36 (0.28 to 0.46) | 0.57 (0.42 to 0.78) | 3,434.73 (2,296.46 to 4,865.37) | 0.50 (0.42 to 0.60) | 0.08 (0.05 to 0.12) | 495.82 (303.29 to 764.90) | 0.43 (0.34 to 0.54) |
| Bipolar disorder | | | | | | | | | |
| Total | 0.12 (0.09 to 0.16) | 361.28 (261.36 to 492.38) | -0.03 (-0.03 to --0.02) | 0.02 (0.01 to 0.03) | 60.56 (39.20 to 87.71) | 0.00 (-0.01 to 0.01) | 0.03 (0.02 to 0.04) | 80.74 (48.72 to 126.20) | -0.03 (-0.07 to 0.01) |
| Female | 0.06 (0.04 to 0.08) | 378.23 (273.87 to 514.56) | -0.03 (-0.03 to -0.02) | 0.01 (0.01 to 0.01) | 63.18 (40.89 to 91.63) | 0.00 (-0.01 to 0.01) | 0.01 (0.01 to 0.02) | 84.05 (50.20 to 131.88) | -0.03 (-0.09 to 0.03) |
| Male | 0.06 (0.04 to 0.08) | 345.14 (249.50 to 471.78) | -0.03 (-0.03 to -0.02) | 0.01 (0.01 to 0.01) | 58.07 (37.58 to 84.18) | 0.00 (-0.01 to 0.01) | 0.01 (0.01 to 0.02) | 77.58 (46.59 to 122.38) | -0.03 (-0.09 to 0.04) |
| Anxiety disorders | | | | | | | | | |
| Total | 1.78 (1.42 to 2.23) | 5,418.45 (4,069.09 to 7,113.36) | 0.45 (0.37 to 0.53) | 0.32 (0.23 to 0.43) | 978.56 (655.73 to 1,348.79) | 0.46 (0.39 to 0.55) | 0.22 (0.14 to 0.32) | 668.38 (419.65 to 991.38) | 0.45 (0.37 to 0.53) |
| Female | 1.10 (0.87 to 1.37) | 6,857.56 (5,187.32 to 8,975.90) | 0.46 (0.38 to 0.54) | 0.20 (0.14 to 0.26) | 1,211.93 (817.88 to 1,668.82) | 0.47 (0.39 to 0.57) | 0.13 (0.09 to 0.20) | 842.96 (530.90 to 1,255.16) | 0.46 (0.37 to 0.54) |
| Male | 0.68 (0.54 to 0.86) | 4,049.80 (3,019.53 to 5,363.84) | 0.43 (0.36 to 0.51) | 0.13 (0.09 to 0.17) | 756.71 (501.26 to 1,036.43) | 0.45 (0.37 to 0.53) | 0.08 (0.05 to 0.12) | 502.36 (312.26 to 749.48) | 0.43 (0.35 to -.52) |
| Eating disorders | | | | | | | | | |
| Total | 0.12 (0.08 to 0.17) | 364.49 (231.54 to 558.84) | -0.02 (-0.04 to 0.00) | 0.13 (0.08 to 0.20) | 411.78 (224.60 to 710.54) | 0.00 (-.002 to 0.02) | 0.03 (0.01 to 0.04) | 78.32 (42.46 to 133.16) | -0.02 (-0.06 to 0.02) |
| Female | 0.08 (0.05 to 0.11) | 481.48 (314.69 to 718.71) | -0.02 (-0.04 to 0.00) | 0.06 (0.04 to 0.08) | 350.62 (207.64 to 564.12) | 0.00 (-0.03 to 0.02) | 0.02 (0.01 to 0.03) | 103.14 (57.32 to 170.37) | -0.02 (-0.07 to 0.03) |
| Male | 0.04 (0.03 to 0.06) | 253.10 (148.74 to 425.93) | -0.02 (-0.04 to 0.01) | 0.08 (0.05 to 0.12) | 469.99 (236.66 to 852.05) | 0.00 (-0.03 to 0.02) | 0.01 (0.00 to 0.01) | 54.70 (27.69 to 97.83) | -0.02 (-0.08 to 0.04) |
| Autism spectrum disorders | | | | | | | | | |
| Total | 0.33 (0.27 to 0.39) | 991.29 (832.13 to 1,178.31) | 0.00 (-0.02 to 0.02) | NA | NA | NA | 0.06 (0.04 to 0.09) | 189.68 (129.96 to 266.54) | 0.00 (-0.03 to 0.03) |
| Female | 0.11 (0.09 to 0.13) | 684.29 (569.81 to 815.99) | 0.00 (-0.02 to 0.02) | NA | NA | NA | 0.02 (0.01 to 0.03) | 130.30 (88.78 to 182.08) | 0.00 (-0.04 to 0.04) |
| Male | 0.22 (0.18 to 0.26) | 1,283.16 (1,078.39 to 1,524.90) | 0.00 (-0.02 to 0.02) | NA | NA | NA | 0.04 (0.03 to 0.06) | 246.14 (169.80 to 344.36) | 0.00 (-0.04 to 0.03) |
| Attention-deficit/hyperactivity disorder | | | | | | | | | |
| Total | 0.70 (0.48 to 1.02) | 2,122.03 (1,423.92 to 3,057.19) | 0.01 (0.00 to 0.01) | 0.00 (0.00 to 0.01) | 11.03 (7.31 to 16.37) | 0.04 (0.04 to 0.04) | 0.01 (0.00 to 0.01) | 25.98 (13.46 to 44.41) | 0.01 (-0.03 to 0.02) |
| Female | 0.20 (0.14 to 0.29) | 1,259.76 (829.67 to 1,824.66) | 0.01 (0.00 to 0.01) | 0.00 (0.00 to 0.00) | 6.61 (4.33 to 9.92) | 0.04 (0.04 to 0.04) | 0.00 (0.00 to 0.00) | 15.37 (7.66 to 26.70) | 0.01 (-0.04 to 0.06) |
| Male | 0.50 (0.35 to 0.71) | 2,941.40 (1,992.11 to 4,251.28) | 0.01 (0.00 to 0.01) | 0.00 (0.00 to 0.00) | 15.21 (10.20 to 22.31) | 0.04 (0.04 to 0.04) | 0.01 (0.00 to 0.01) | 36.05 (18.91 to 61.49) | 0.01 (-0.02 to 0.04) |
| Conduct disorder | | | | | | | | | |
| Total | 0.69 (0.50 to 0.90) | 2,042.04 (1,419.50 to 2,768.69) | 0.03 (0.03 to 0.03) | 0.18 (0.12 to 0.24) | 525.57 (317.93 to 743.19) | 0.04 (0.04 to 0.04) | 0.08 (0.05 to 0.13) | 249.83 (132.65 to 402.57) | 0.03 (0.01 to 0.05) |
| Female | 0.27 (0.19 to 0.36) | 1,641.32 (1,089.50 to 2,313.86) | 0.03 (0.03 to 0.03) | 0.07 (0.05 to 0.10) | 428.19 (251.47 to 636.42) | 0.04 (0.04 to 0.04) | 0.03 (0.02 to 0.05) | 199.98 (102.91 to 331.05) | 0.03 (0.00 to 0.06) |
| Male | 0.42 (0.31 to 0.54) | 2,422.33 (1,709.16 to 3,259.57) | 0.03 (0.03 to 0.03) | 0.11 (0.07 to 0.15) | 617.92 (376.10 to 860.66) | 0.04 (0.04 to 0.04) | 0.05 (0.03 to 0.08) | 297.13 (160.69 to 473.06) | 0.03 (0.00 to 0.05) |
| Idiopathic developmental intellectual disability | | | | | | | | | |
| Total | 0.16 (0.04 to 0.29) | 490.33 (115.76 to 863.98) | 0.00 (-0.06 to 0.06) | NA | NA | NA | 0.01 (0.00 to 0.01) | 20.24 (3.72 to 41.76) | 0.00 (-0.05 to 0.04) |
| Female | 0.07 (0.02 to 0.12) | 438.17 (125.58 to 742.43) | 0.00 (-0.05 to 0.06) | NA | NA | NA | 0.00 (0.00 to 0.01) | 17.39 (3.80 to 35.02) | 0.00 (-0.06 to 0.06) |
| Male | 0.09 (0.02 to 0.17) | 539.90 (109.92 to 978.94) | 0.00 (-0.09 to 0.07) | NA | NA | NA | 0.00 (0.00 to 0.01) | 22.94 (3.92 to 48.42) | 0.00 (-0.07 to 0.06) |
| Other mental disorders | | | | | | | | | |
| Total | 0.13 (0.08 to 0.19) | 416.70 (261.62 to 596.11) | -0.04 (-0.04 to -0.04) | NA | NA | NA | 0.01 (0.01 to 0.02) | 32.05 (17.69 to 51.40) | -0.04 (-0.09 to 0.02) |
| Female | 0.05 (0.03 to 0.07) | 326.32 (198.74 to 468.85) | -0.04 (-0.04 to -0.04) | NA | NA | NA | 0.00 (0.00 to 0.01) | 24.91 (13.00 to 40.96) | -0.04 (-0.13 to 0.06) |
| Male | 0.08 (0.05 to 0.12) | 502.85 (319.07 to 720.45) | -0.04 (-0.04 to -0.04) | NA | NA | NA | 0.01 (0.00 to 0.01) | 38.86 (21.69 to 61.88) | -0.04 (-0.11 to 0.04) |

**Table S11.** Prevalence, Incidence, and Years Lived with Disability of Mental Disorders Among Adolescents and Young Adults (Aged 10-24) in **Eastern Sub−Saharan Afric**a in 2021, and Percentage Change from 2019 to 2021 According to GBD.

|  | Prevalence (95% uncertainty interval) | | | Incidence (95% uncertainty interval) | | | Years lived with disability (95% uncertainty interval) | | |
| --- | --- | --- | --- | --- | --- | --- | --- | --- | --- |
| Cause | Counts in million | Age-standardized rates per 100,000 people | Percentage rate change (2019-2021) | Counts in million | Age-standardized rates per 100,000 people | Percentage rate change (2019-2021) | Counts in million | Age-standardized rates per 100,000 people | Percentage rate change (2019-2021) |
| Mental disorders | | | | | | | | | |
| Total | 19.38 (17.15 to 21.91) | 13,423.40 (11,469.33 to 15,579.28) | 0.11 (0.08 to 0.14) | 8.91 (6.92 to 11.30) | 6,202.18 (4,538.53 to 8,226.10) | 0.20 (0.15 to 0.26) | 2.76 (1.99 to 3.64) | 1,923.53 (1,362.30 to 2,595.86) | 0.13 (0.09 to 0.17) |
| Female | 9.86 (8.59 to 11.34) | 13,633.66 (11,412.00 to 16,022.60) | 0.13 (0.09 to 0.16) | 4.92 (3.73 to 6.36) | 6,817.18 (4,909.83 to 9,219.62) | 0.22 (0.16 to 0.28) | 1.45 (1.04 to 1.93) | 2,012.66 (1,404.93 to 2,738.71) | 0.15 (0.11 to 0.19) |
| Male | 9.52 (8.49 to 10.68) | 13,189.68 (11,402.68 to 15,137.43) | 0.08 (0.06 to 0.11) | 3.99 (3.12 to 5.00) | 5,574.73 (4,136.56 to 7,323.48) | 0.19 (0.13 to 0.24) | 1.3 (0.95 to 1.71) | 1,830.90 (1,305.31 to 2,441.78) | 0.11 (0.08 to 0.15) |
| Schizophrenia | | | | | | | | | |
| Total | 0.11 (0.07 to 0.16) | 80.34 (48.21 to 126.64) | 0.01 (-0.01 to 0.04) | 0.03 (0.02 to 0.04) | 20.12 (11.17 to 31.70) | 0.01 (-0.01 to 0.03) | 0.07 (0.04 to 0.11) | 53.31 (30.71 to 87.90) | 0.01 (-0.07 to 0.09) |
| Female | 0.06 (0.04 to 0.08) | 81.54 (48.72 to 128.90) | 0.01 (-0.02 to 0.05) | 0.01 (0.01 to 0.02) | 20.70 (11.54 to 32.75) | 0.01 (-0.02 to 0.04) | 0.04 (0.02 to 0.06) | 53.60 (30.71 to 88.59) | 0.00 (-0.10 to 0.13) |
| Male | 0.05 (0.03 to 0.08) | 79.08 (47.36 to 124.83) | 0.02 (-0.01 to 0.05) | 0.01 (0.01 to 0.02) | 19.51 (10.79 to 30.66) | 0.01 (-0.02 to 0.04) | 0.04 (0.02 to 0.06) | 52.99 (30.41 to 87.93) | 0.01 (-0.10 to 0.13) |
| Depressive disorders | | | | | | | | | |
| Total | 5.09 (3.82 to 6.77) | 3,598.63 (2,584.56 to 4,926.87) | 0.19 (0.13 to 0.26) | 6.32 (4.36 to 8.66) | 4,442.97 (2,934.32 to 6,449.54) | 0.25 (0.18 to 0.33) | 0.93 (0.57 to 1.36) | 654.69 (392.56 to 1,004.82) | 0.22 (0.15 to 0.30) |
| Female | 3.02 (2.26 to 4.00) | 4,226.92 (3,007.86 to 5,791.59) | 0.19 (0.12 to 0.26) | 3.67 (2.53 to 5.12) | 5,107.61 (3,350.06 to 7,463.14) | 0.26 (0.18 to 0.34) | 0.54 (0.33 to 0.80) | 756.36 (445.13 to 1,161.59) | 0.22 (0.13 to 0.30) |
| Male | 2.07 (1.54 to 2.73) | 2,957.52 (2,105.33 to 4,075.57) | 0.19 (0.13 to 0.26) | 2.66 (1.84 to 3.59) | 3,768.36 (2,507.08 to 5,447.91) | 0.25 (0.17 to 0.33) | 0.39 (0.24 to 0.58) | 551.15 (331.80 to 850.31) | 0.22 (0.15 to 0.31) |
| Bipolar disorder | | | | | | | | | |
| Total | 0.65 (0.46 to 0.93) | 463.42 (316.28 to 670.47) | 0.01 (0.01 to 0.01) | 0.11 (0.08 to 0.15) | 75.25 (46.29 to 114.53) | 0.00 (0.00 to 0.00) | 0.14 (0.09 to 0.23) | 102.95 (60.19 to 166.07) | 0.00 (-0.04 to 0.04) |
| Female | 0.34 (0.24 to 0.49) | 478.88 (327.68 to 694.49) | 0.01 (0.01 to 0.01) | 0.06 (0.04 to 0.08) | 77.42 (47.59 to 117.82) | 0.00 (0.00 to 0.00) | 0.08 (0.04 to 0.12) | 105.78 (61.08 to 170.75) | 0.00 (-0.05 to 0.06) |
| Male | 0.31 (0.22 to 0.44) | 447.64 (304.59 to 646.95) | 0.01 (0.01 to 0.01) | 0.05 (0.04 to 0.07) | 73.11 (44.87 to 111.02) | 0.00 (0.00 to 0.00) | 0.07 (0.04 to 0.11) | 100.07 (57.81 to 161.83) | 0.00 (-0.05 to 0.06) |
| Anxiety disorders | | | | | | | | | |
| Total | 6.70 (4.97 to 8.87) | 4,666.64 (3,319.51 to 6,420.21) | 0.23 (0.15 to 0.31) | 1.29 (0.89 to 1.77) | 892.13 (563.71 to 1,275.25) | 0.23 (0.15 to 0.31) | 0.82 (0.50 to 1.22) | 570.81 (346.00 to 872.93) | 0.23 (0.14 to 0.30) |
| Female | 3.98 (2.95 to 5.27) | 5,505.48 (3,904.64 to 7,575.50) | 0.23 (0.15 to 0.32) | 0.74 (0.51 to 1.03) | 1,014.00 (638.53 to 1,452.74) | 0.23 (0.15 to 0.33) | 0.48 (0.30 to 0.72) | 670.79 (408.91 to 1,029.59) | 0.23 (0.15 to 0.31) |
| Male | 2.73 (2.04 to 3.61) | 3,815.83 (2,713.21 to 5,265.67) | 0.22 (0.14 to 0.30) | 0.56 (0.38 to 0.76) | 769.10 (488.10 to 1,095.15) | 0.22 (0.14 to 0.30) | 0.34 (0.20 to 0.50) | 469.47 (283.87 to 718.26) | 0.22 (0.13 to 0.30) |
| Eating disorders | | | | | | | | | |
| Total | 0.30 (0.21 to 0.44) | 214.40 (132.51 to 342.53) | 0.01 (0.00 to 0.04) | 0.40 (0.25 to 0.60) | 275.41 (147.30 to 483.25) | 0.01 (-0.01 to 0.03) | 0.06 (0.04 to 0.11) | 45.77 (23.75 to 78.64) | 0.01 (-0.04 to 0.06) |
| Female | 0.18 (0.13 to 0.26) | 256.89 (161.65 to 399.75) | 0.02 (-0.01 to 0.05) | 0.15 (0.10 to 0.22) | 212.77 (122.63 to 351.56) | 0.01 (-0.02 to 0.03) | 0.04 (0.02 to 0.06) | 54.57 (29.07 to 91.60) | 0.01 (-0.05 to 0.08) |
| Male | 0.12 (0.08 to 0.18) | 170.79 (100.00 to 288.93) | 0.01 (-0.01 to 0.04) | 0.24 (0.14 to 0.38) | 338.97 (170.93 to 613.37) | 0.01 (0.02 to 0.04) | 0.03 (0.01 to 0.04) | 36.73 (17.90 to 66.09) | 0.01 (-0.06 to 0.08) |
| Autism spectrum disorders | | | | | | | | | |
| Total | 1.40 (1.18 to 1.65) | 964.64 (813.31 to 1,130.08) | 0.00 (-0.02 to 0.02) | NA | NA | NA | 0.27 (0.18 to 0.38) | 183.16 (124.96 to 257.30) | 0.00 (-0.03 to 0.03) |
| Female | 0.50 (0.42 to 0.59) | 683.93 (576.42 to 808.75) | 0.00 (-0.02 to 0.03) | NA | NA | NA | 0.09 (0.07 to 0.13) | 129.21 (89.16 to 182.41) | 0.00 (-0.04 to 0.04) |
| Male | 0.91 (0.77 to 1.06) | 1,247.28 (1,055.46 to 1,464.65) | 0.00 (-0.02 to 0.03) | NA | NA | NA | 0.17 (0.12 to 0.24) | 237.48 (161.39 to 331.19) | 0.00 (-0.04 to 0.03) |
| Attention-deficit/hyperactivity disorder | | | | | | | | | |
| Total | 1.52 (1.04 to 2.20) | 1,030.88 (688.37 to 1,487.90) | 0.00 (0.00 to 0.00) | 0.01 (0.01 to 0.01) | 5.71 (3.75 to 8.47) | -0.01 (-0.01 to -0.01) | 0.02 (0.01 to 0.03) | 12.54 (6.47 to 21.36) | 0.00 (-0.04 to 0.04) |
| Female | 0.44 (0.30 to 0.65) | 599.90 (392.88 to 890.38) | 0.00 (0.00 to 0.00) | 0.00 (0.00 to 0.00) | 3.32 (2.18 to 5.09) | -0.01 (-0.01 to -0.01) | 0.01 (0.00 to 0.01) | 7.27 (3.70 to 12.55) | 0.00 (-0.07 to 0.07) |
| Male | 1.08 (0.74 to 1.55) | 1,462.10 (978.47 to 2,104.07) | 0.00 (0.00 to 0.00) | 0.01 (0.00 to 0.01) | 8.04 (5.35 to 11.79) | -0.02 (-0.02 to -0.01) | 0.01 (0.01 to 0.02) | 17.80 (9.12 to 30.29) | 0.00 (-0.05 to 0.04) |
| Conduct disorder | | | | | | | | | |
| Total | 2.92 (2.06 to 3.75) | 1,922.56 (1,329.10 to 2,621.42) | -0.01 (-0.01 to -0.01) | 0.75 (0.50 to 1.03) | 490.59 (300.85 to 695.25) | -0.01 (-0.01 to -0.01) | 0.35 (0.19 to 0.56) | 233.10 (123.47 to 377.77) | -0.01 (-0.03 to 0.01) |
| Female | 1.12 (0.76 to 1.48) | 1,481.69 (957.06 to 2,109.19) | -0.01 (-0.01 to -0.01) | 0.29 (0.18 to 0.42) | 381.37 (217.45 to 582.64) | -0.01 (-0.01 to -0.01) | 0.13 (0.07 to 0.22) | 179.07 (91.62 to 297.70) | -0.01 (-0.04 to 0.03) |
| Male | 1.80 (1.31 to 2.30) | 2,358.37 (1,663.55 to 3,163.06) | -0.01 (-0.01 to -0.01) | 0.46 (0.32 to 0.62) | 597.64 (365.26 to 849.57) | -0.01 (-0.01 to -0.01) | 0.22 (0.12 to 0.35) | 286.51 (154.31 to 460.65) | -0.01 (-0.04 to 0.02) |
| Idiopathic developmental intellectual disability | | | | | | | | | |
| Total | 1.12 (0.41 to 1.83) | 765.19 (276.45 to 1,255.36) | -0.01 (-0.09 to 0.08) | NA | NA | NA | 0.04 (0.01 to 0.09) | 30.72 (8.28 to 60.28) | 0.00 (-0.04 to 0.04) |
| Female | 0.51 (0.21 to 0.82) | 702.37 (284.73 to 1,126.16) | 0.00 (-0.08 to 0.09) | NA | NA | NA | 0.02 (0.01 to 0.04) | 27.64 (8.22 to 54.00) | 0.00 (-0.05 to 0.05) |
| Male | 0.60 (0.20 to 1.01) | 828.31 (268.75 to 1,386.38) | -0.01 (-0.09 to 0.07) | NA | NA | NA | 0.02 (0.01 to 0.05) | 33.81 (8.57 to 66.97) | -0.01 (-0.05 to 0.05) |
| Other mental disorders | | | | | | | | | |
| Total | 0.66 (0.42 to 0.93) | 476.74 (304.50 to 677.04) | 0.01 (0.01 to 0.01) | NA | NA | NA | 0.05 (0.03 to 0.08) | 36.49 (20.48 to 58.14) | 0.01 (-0.05 to 0.07) |
| Female | 0.26 (0.17 to 0.37) | 374.06 (237.92 to 529.93) | 0.01 (0.01 to 0.01) | NA | NA | NA | 0.02 (0.01 to 0.03) | 28.36 (15.69 to 45.08) | 0.00 (-0.09 to 0.10) |
| Male | 0.40 (0.25 to 0.57) | 582.87 (373.88 to 840.42) | 0.01 (0.01 to 0.01) | NA | NA | NA | 0.03 (0.02 to 0.05) | 44.89 (25.26 to 71.61) | 0.01 (-0.05 to 0.08) |

**Table S12.** Prevalence, Incidence, and Years Lived with Disability of Mental Disorders Among Adolescents and Young Adults (Aged 10-24) in **High−income Asia Pacific** in 2021, and Percentage Change from 2019 to 2021 According to GBD.

|  | Prevalence (95% uncertainty interval) | | | Incidence (95% uncertainty interval) | | | Years lived with disability (95% uncertainty interval) | | |
| --- | --- | --- | --- | --- | --- | --- | --- | --- | --- |
| Cause | Counts in million | Age-standardized rates per 100,000 people | Percentage rate change (2019-2021) | Counts in million | Age-standardized rates per 100,000 people | Percentage rate change (2019-2021) | Counts in million | Age-standardized rates per 100,000 people | Percentage rate change (2019-2021) |
| Mental disorders | | | | | | | | | |
| Total | 3.78 (3.37 to 4.21) | 14,382.59 (12,517.49 to 16,338.62) | 0.10 (0.07 to 0.13) | 1.47 (1.20 to 1.79) | 5,532.94 (4,291.28 to 6,924.54) | 0.21 (0.15 to 0.27) | 0.54 (0.40 to 0.70) | 2,009.56 (1,458.02 to 2,659.69) | 0.12 (0.08 to 0.16) |
| Female | 1.74 (1.54 to 1.95) | 13,487.25 (11,626.05 to 15,556.51) | 0.13 (0.09 to 0.18) | 0.82 (0.66 to 1.00) | 6,278.03 (4,868.20 to 7,915.30) | 0.23 (0.16 to 0.30) | 0.27 (0.20 to 0.36) | 2,057.10 (1,472.49 to 2,750.11) | 0.14 (0.10 to 0.20) |
| Male | 2.04 (1.81 to 2.27) | 15,229.06 (13,223.17 to 17,303.37) | 0.07 (0.05 to 0.10) | 0.65 (0.54 to 0.79) | 4,828.56 (3,735.70 to 6,078.65) | 0.19 (0.13 to 0.25) | 0.27 (0.20 to 0.34) | 1,964.56 (1,441.67 to 2,575.56) | 0.09 (0.06 to 0.13) |
| Schizophrenia | | | | | | | | | |
| Total | 0.03 (0.02 to 0.04) | 91.42 (56.80 to 137.70) | 0.00 (-0.03 to 0.04) | 0.01 (0.00 to 0.01) | 23.75 (13.81 to 35.73) | 0.00 (-0.04 to 0.03) | 0.02 (0.01 to 0.03) | 61.33 (35.95 to 98.10) | -0.01 (-0.01 to 0.10) |
| Female | 0.01 (0.01 to 0.02) | 85.00 (52.13 to 129.21) | 0.00 (-0.05 to 0.04) | 0.00 (0.00 to 0.00) | 21.97 (12.66 to 33.62) | -0.01 (-0.05 to 0.04) | 0.01 (0.00 to 0.01) | 56.45 (33.08 to 91.34) | -0.01 (-0.03 to 0.16) |
| Male | 0.01 (0.01 to 0.02) | 97.49 (61.77 to 146.25) | 0.00 (-0.04 to 0.05) | 0.00 (0.00 to 0.01) | 25.44 (15.06 to 38.04) | 0.00 (-0.04 to 0.05) | 0.01 (0.01 to 0.02) | 65.96 (38.33 to 106.81) | 0.00 (-0.12 to 0.13) |
| Depressive disorders | | | | | | | | | |
| Total | 0.75 (0.58 to 0.95) | 2,710.29 (2,028.06 to 3,549.56) | 0.22 (0.14 to 0.31) | 0.98 (0.72 to 1.30) | 3,564.99 (2,503.17 to 4,844.92) | 0.27 (0.19 to 0.37) | 0.14 (0.09 to 0.21) | 516.63 (324.41 to 772.84) | 0.24 (0.16 to 0.34) |
| Female | 0.43 (0.33 to 0.54) | 3,172.19 (2,371.15 to 4,154.76) | 0.23 (0.15 to 0.33) | 0.57 (0.42 to 0.75) | 4,230.83 (2,975.39 to 5,741.21) | 0.28 (0.18 to 0.40) | 0.08 (0.05 to 0.12) | 604.92 (379.40 to 900.89) | 0.26 (0.16 to 0.37) |
| Male | 0.32 (0.25 to 0.40) | 2,273.38 (1,688.34 to 2,969.79) | 0.20 (0.12 to 0.28) | 0.41 (0.30 to 0.54) | 2,935.40 (2,048.46 to 3,997.78) | 0.25 (0.17 to 0.35) | 0.06 (0.04 to 0.09) | 433.10 (269.70 to 651.70) | 0.23 (0.14 to 0.32) |
| Bipolar disorder | | | | | | | | | |
| Total | 0.12 (0.09 to 0.16) | 420.57 (304.82 to 567.70) | -0.01 (-0.01 to -0.01) | 0.02 (0.01 to 0.02) | 65.06 (43.35 to 93.06) | 0.00 (0.00 to 0.00) | 0.03 (0.02 to 0.04) | 94.13 (56.73 to 147.30) | -0.01 (-0.07 to 0.04) |
| Female | 0.06 (0.04 to 0.08) | 429.65 (306.30 to 587.85) | -0.01 (-0.01 to 0.00) | 0.01 (0.01 to 0.01) | 68.64 (44.70 to 99.96) | 0.00 (0.00 to 0.00) | 0.01 (0.01 to 0.02) | 95.56 (57.58 to 151.46) | -0.01 (-0.08 to 0.06) |
| Male | 0.06 (0.04 to 0.08) | 411.94 (302.46 to 551.30) | -0.01 (-0.01 to -0.01) | 0.01 (0.01 to 0.01) | 61.67 (41.94 to 87.25) | 0.00 (0.00 to 0.00) | 0.01 (0.01 to 0.02) | 92.77 (54.57 to 145.81) | -0.01 (-0.08 to 0.07) |
| Anxiety disorders | | | | | | | | | |
| Total | 1.15 (0.88 to 1.49 ) | 4,391.93 (3,195.77 to 5,882.66) | 0.26 (0.15 to 0.37) | 0.19 (0.14 to 0.26) | 754.68 (499.92 to 1,050.20) | 0.30 (0.19 to 0.41) | 0.14 (0.09 to 0.21) | 542.23 (335.60 to 822.64) | 0.26 (0.14 to 0.37) |
| Female | 0.68 (0.52 to 0.88) | 5,306.36 (3,839.54 to 7,102.51) | 0.27 (0.16 to 0.38) | 0.12 (0.08 to 0.16) | 920.16 (604.86 to 1,284.73) | 0.31 (0.20 to 0.43) | 0.08 (0.05 to 0.12) | 652.54 (404.75 to 984.89) | 0.27 (0.15 to 0.39) |
| Male | 0.47 (0.36 to 0.61) | 3,527.28 (2,580.01 to 4,737.76) | 0.24 (0.14 to 0.34) | 0.08 (0.06 to 0.10) | 598.21 (399.46 to 835.67) | 0.28 (0.17 to 0.39) | 0.06 (0.04 to 0.09) | 437.93 (269.44 to 671.45) | 0.24 (0.12 to 0.35) |
| Eating disorders | | | | | | | | | |
| Total | 0.22 (0.16 to 0.30) | 783.73 (520.17 to 1,156.73) | -0.01 (-0.04 to 0.02) | 0.16 (0.10 to 0.23) | 610.28 (343.09 to 1,025.20) | 0.00 (-0.02 to 0.03) | 0.05 (0.03 to 0.08) | 168.86 (95.19 to 278.92) | -0.01 (-0.05 to 0.03) |
| Female | 0.15 (0.11 to 0.20) | 1,090.40 (746.38 to 1,584.02) | -0.01 (-0.04 to 0.03) | 0.08 (0.06 to 0.11) | 640.00 (381.53 to 1,029.88) | -0.01 (-0.05 to 0.03) | 0.03 (0.02 to 0.05) | 234.48 (135.21 to 382.07) | -0.01 (-0.05 to 0.04) |
| Male | 0.07 (0.05 to 0.11) | 493.85 (299.26 to 805.73) | -0.01 (-0.06 to 0.03) | 0.08 (0.05 to 0.12) | 582.21 (302.33 to 1,026.27) | 0.01 (-0.03 to 0.05) | 0.02 (0.01 to 0.02) | 106.83 (55.22 to 189.44) | -0.02 (-0.09 to 0.06) |
| Autism spectrum disorders | | | | | | | | | |
| Total | 0.43 (0.36 to 0.50) | 1,630.72 (1,374.26 to 1,915.32) | 0.01 (-0.02 to 0.03) | NA | NA | NA | 0.08 (0.06 to 0.11) | 312.00 (214.24 to 437.57) | 0.00 (-0.03 to 0.04) |
| Female | 0.12 (0.10 to 0.15) | 981.34 (825.43 to 1,155.54) | 0.01 (-0.03 to 0.04) | NA | NA | NA | 0.02 (0.02 to 0.03) | 186.61 (128.75 to 258.75) | 0.00 (-0.04 to 0.05) |
| Male | 0.30 (0.25 to 0.35) | 2,244.80 (1,890.71 to 2,634.08) | 0.01 (-0.03 to 0.04) | NA | NA | NA | 0.06 (0.04 to 0.08) | 430.58 (296.63 to 606.04) | 0.01 (-0.04 to 0.05) |
| Attention-deficit/hyperactivity disorder | | | | | | | | | |
| Total | 0.69 (0.48 to 0.97) | 2,708.00 (1,833.77 to 3,888.09) | 0.02 (-0.02 to 0.05) | 0.00 (0.00 to 0.01) | 15.14 (10.29 to 22.48) | 0.05 (0.01 to 0.09) | 0.01 (0.00 to 0.01) | 33.16 (16.93 to 56.20) | 0.01 (-0.03 to 0.07) |
| Female | 0.16 (0.11 to 0.23) | 1,288.38 (871.06 to 1,846.92) | 0.02 (-0.03 to 0.07) | 0.00 (0.00 to 0.00) | 7.14 (4.79 to 10.62) | 0.05 (0.00 to 0.11) | 0.00 (0.00 to 0.00) | 15.72 (8.11 to 26.70) | 0.02 (-0.06 to 0.10) |
| Male | 0.53 (0.36 to 0.74) | 4,050.39 (2,743.70 to 5,855.82) | 0.02 (-0.03 to 0.07) | 0.00 (0.00 to 0.00) | 22.73 (15.25 to 33.11) | 0.05 (0.00 to 0.10) | 0.01 (0.00 to 0.01) | 49.65 (24.84 to 85.19) | 0.01 (-0.04 to 0.08) |
| Conduct disorder | | | | | | | | | |
| Total | 0.46 (0.33 to 0.59) | 1,919.15 (1,309.71 to 2,605.65) | 0.01 (0.00 to 0.01) | 0.12 (0.08 to 0.16) | 499.04 (301.60 to 706.43) | 0.02 (0.01 to 0.03) | 0.06 (0.03 to 0.09) | 234.67 (122.94 to 377.93) | 0.01 (-0.02 to 0.03) |
| Female | 0.17 (0.12 to 0.23) | 1,462.13 (964.85 to 2,087.59) | 0.01 (0.00 to 0.02) | 0.04 (0.03 to 0.06) | 389.30 (227.77 to 584.84) | 0.02 (0.01 to 0.03) | 0.02 (0.01 to 0.03) | 178.03 (90.16 to 298.39) | 0.01 (-0.03 to 0.06) |
| Male | 0.29 (0.21 to 0.37) | 2,350.98 (1,637.42 to 3,153.75) | 0.01 (0.00 to 0.01) | 0.07 (0.05 to 0.10) | 602.90 (366.32 to 847.22) | 0.02 (0.01 to 0.03) | 0.04 (0.02 to 0.06) | 288.18 (152.38 to 460.66) | 0.01 (-0.03 to 0.04) |
| Idiopathic developmental intellectual disability | | | | | | | | | |
| Total | 0.02 (0.00 to 0.07) | 80.62 (2.99 to 268.93) | -0.17 (-0.56 to -0.08) | NA | NA | NA | 0.00 (0.00 to 0.00) | 3.92 (0.07 to 12.66) | -0.17 (-0.62 to -0.07) |
| Female | 0.00 (0.00 to 0.02) | 30.09 (0.00 to 127.16) | -0.16 (-1.00 to 0.00) | NA | NA | NA | 0.00 (0.00 to 0.00) | 1.29 (0.00 to 5.74) | -0.16 (-1.00 to 0.01) |
| Male | 0.02 (0.00 to 0.05) | 128.41 (5.82 to 403.92) | -0.17 (-0.56 to -0.08) | NA | NA | NA | 0.00 (0.00 to 0.00) | 6.41 (0.14 to 19.45) | -0.17 (-0.62 to -0.06) |
| Other mental disorders | | | | | | | | | |
| Total | 0.16 (0.11 to 0.22) | 552.96 (366.10 to 760.66) | -0.01 (-0.01 to -0.01) | NA | NA | NA | 0.01 (0.01 to 0.02) | 42.63 (24.42 to 67.97) | -0.02 (-0.08 to 0.06) |
| Female | 0.06 (0.04 to 0.08) | 410.42 (262.49 to 575.90) | -0.01 (-0.01 to -0.01) | NA | NA | NA | 0.00 (0.00 to 0.01) | 31.51 (17.77 to 51.51) | -0.01 (-0.13 to 0.11) |
| Male | 0.10 (0.07 to 0.14) | 687.83 (456.65 to 933.27) | -0.01 (-0.02 to -0.01) | NA | NA | NA | 0.01 (0.00 to 0.01) | 53.15 (30.50 to 83.50) | -0.02 (-0.11 to 0.09) |

**Table S13.** Prevalence, Incidence, and Years Lived with Disability of Mental Disorders Among Adolescents and Young Adults (Aged 10-24) in **High−income North America** in 2021, and Percentage Change from 2019 to 2021 According to GBD.

|  | Prevalence (95% uncertainty interval) | | | Incidence (95% uncertainty interval) | | | Years lived with disability (95% uncertainty interval) | | |
| --- | --- | --- | --- | --- | --- | --- | --- | --- | --- |
| Cause | Counts in million | Age-standardized rates per 100,000 people | Percentage rate change (2019-2021) | Counts in million | Age-standardized rates per 100,000 people | Percentage rate change (2019-2021) | Counts in million | Age-standardized rates per 100,000 people | Percentage rate change (2019-2021) |
| Mental disorders | | | | | | | | | |
| Total | 15.64 (14.13 to 17.33) | 21,723.90 (19,114.17 to 24,557.57) | 0.15 (0.12 to 0.18) | 8.94 (7.42 to 10.69) | 12,401.45 (9,757.56 to 15,467.33) | 0.33 (0.29 to 0.39) | 2.30 (1.67 to 3.04) | 3,176.20 (2,275.39 to 4,198.77) | 0.21 (0.17 to 0.25) |
| Female | 8.08 (7.27 to 8.96) | 22,829.57 (20,050.49 to 25,879.54) | 0.20 (0.17 to 0.23) | 5.73 (4.73 to 6.89) | 16,289.91 (12,809.67 to 20,457.08) | 0.35 (0.30 to 0.40) | 1.34 (0.96 to 1.77) | 3,761.83 (2,657.78 to 5,014.87) | 0.24 (0.21 to 0.29) |
| Male | 7.56 (6.71 to 8.52) | 20,664.45 (17,988.90 to 23,694.81) | 0.10 (0.06 to 0.14) | 3.20 (2.64 to 3.87) | 8,681.22 (6,842.16 to 10,820.45) | 0.31 (0.26 to 0.36) | 0.97 (0.71 to 1.26) | 2,615.70 (1,898.78 to 3,434.29) | 0.17 (0.13 to 0.20) |
| Schizophrenia | | | | | | | | | |
| Total | 0.10 (0.07 to 0.13) | 129.75 (89.29 to 178.08) | 0.01 (-0.02 to 0.03) | 0.03 (0.02 to 0.03) | 33.26 (21.85 to 46.28) | 0.01 (-0.02 to 0.03) | 0.06 (0.04 to 0.09) | 84.42 (53.42 to 126.47) | 0.00 (-0.06 to 0.06) |
| Female | 0.05 (0.03 to 0.06) | 125.38 (85.13 to 173.13) | 0.01 (-0.02 to 0.04) | 0.01 (0.01 to 0.02) | 32.54 (21.41 to 45.52) | 0.02 (-0.01 to 0.05) | 0.03 (0.02 to 0.04) | 80.40 (50.28 to 120.11) | 0.00 (-0.08 to 0.10) |
| Male | 0.05 (0.04 to 0.07) | 133.94 (92.47 to 182.99) | 0.00 (-0.03 to 0.03) | 0.01 (0.01 to 0.02) | 33.95 (22.18 to 47.12) | 0.00 (-0.03 to 0.02) | 0.03 (0.02 to 0.05) | 88.28 (55.78 to 132.22) | -0.01 (-0.09 to 0.08) |
| Depressive disorders | | | | | | | | | |
| Total | 5.02 (4.01 to 6.09) | 6,870.26 (5,303.03 to 8,820.33) | 0.35 (0.29 to 0.40) | 7.05 (5.51 to 8.91) | 9,734.21 (7,250.54 to 12,737.40) | 0.40 (0.34 to 0.46) | 0.99 (0.67 to 1.44) | 1,356.69 (887.05 to 2,002.38) | 0.37 (0.31 to 0.43) |
| Female | 3.32 (2.66 to 4.06) | 9,339.55 (7,233.75 to 12,022.31) | 0.35 (0.30 to 0.41) | 4.69 (3.66 to 5.83) | 13,315.65 (9,955.09 to 17,394.32) | 0.40 (0.34 to 0.47) | 0.66 (0.45 to 0.95) | 1,847.86 (1,215.58 to 2,722.67) | 0.37 (0.31 to 0.43) |
| Male | 1.69 (1.34 to 2.14) | 4,507.76 (3,433.76 to 5,805.14) | 0.34 (0.27 to 0.40) | 2.35 (1.81 to 3.00) | 6,307.90 (4,647.93 to 8,342.26) | 0.40 (0.33 to 0.47) | 0.33 (0.22 to 0.49) | 886.79 (568.07 to 1,313.96) | 0.37 (0.30 to 0.43) |
| Bipolar disorder | | | | | | | | | |
| Total | 0.56 (0.51 to 0.62) | 764.00 (687.83 to 841.29) | 0.01 (-0.01 to 0.02) | 0.07 (0.07 to 0.08) | 106.38 (93.07 to 120.94) | 0.00 (-0.02 to 0.01) | 0.12 (0.08 to 0.18) | 168.13 (108.88 to 242.46) | 0.00 (-0.03 to 0.04) |
| Female | 0.25 (0.23 to 0.28) | 699.24 (623.28 to 776.70) | 0.01 (-0.01 to 0.03) | 0.03 (0.03 to 0.04) | 98.96 (84.89 to 114.95) | 0.00 (-0.02 to 0.02) | 0.05 (0.04 to 0.08) | 152.29 (97.59 to 221.42) | 0.00 (-0.04 to 0.05) |
| Male | 0.31 (0.28 to 0.34) | 825.95 (746.25 to 903.77) | 0.00 (-0.02 to 0.02) | 0.04 (0.04 to 0.04) | 113.46 (100.20 to 127.81) | 0.00 (-0.02 to 0.02) | 0.07 (0.04 to 0.10) | 183.28 (119.27 to 262.39) | 0.00 (-0.05 to 0.05) |
| Anxiety disorders | | | | | | | | | |
| Total | 4.87 (3.88 to 6.02) | 6,685.14 (4,989.44 to 8,613.57) | 0.30 (0.24 to 0.38) | 0.98 (0.68 to 1.30) | 1,368.48 (914.25 to 1,850.79) | 0.30 (0.23 to 0.38) | 0.59 (0.38 to 0.85) | 810.01 (518.86 to 1,189.31) | 0.30 (0.23 to 0.38) |
| Female | 3.04 (2.41 to 3.75) | 8,512.88 (6,383.65 to 10,964.05) | 0.31 (0.24 to 0.39) | 0.60 (0.41 to 0.79) | 1,701.33 (1,142.51 to 2,323.41) | 0.32 (0.25 to 0.39) | 0.37 (0.24 to 0.53) | 1,025.19 (655.80 to 1,500.26) | 0.31 (0.24 to 0.38) |
| Male | 1.83 (1.42 to 2.28) | 4,935.04 (3,634.44 to 6,419.46) | 0.29 (0.22 to 0.37) | 0.39 (0.26 to 0.51) | 1,049.91 (705.07 to 1,425.90) | 0.28 (0.20 to 0.37) | 0.22 (0.15 to 0.32) | 603.97 (381.82 to 893.85) | 0.29 (0.21 to 0.37) |
| Eating disorders | | | | | | | | | |
| Total | 0.62 (0.43 to 0.89) | 831.19 (535.51 to 1,273.80) | -0.01 (-0.02 to 0.01) | 0.48 (0.30 to 0.71) | 658.72 (356.47 to 1,133.53) | 0.00 (-0.02 to 0.01) | 0.13 (0.07 to 0.21) | 175.92 (96.73 to 297.36) | -0.01 (-0.04 to 0.02) |
| Female | 0.45 (0.32 to 0.64) | 1,251.75 (828.43 to 1,906.49) | -0.01 (-0.03 to 0.01) | 0.27 (0.18 to 0.39) | 762.98 (424.31 to 1,283.00) | -0.02 (-0.04 to 0.01) | 0.10 (0.06 to 0.15) | 264.04 (146.85 to 445.32) | -0.01 (-0.05 to 0.02) |
| Male | 0.16 (0.11 to 0.25) | 428.48 (252.66 to 720.59) | 0.00 (-0.02 to 0.03) | 0.21 (0.13 to 0.32) | 559.01 (285.96 to 1,009.17) | 0.01 (-0.01 to 0.04) | 0.03 (0.02 to 0.06) | 91.55 (46.11 to 164.99) | 0.00 (-0.05 to 0.05) |
| Autism spectrum disorders | | | | | | | | | |
| Total | 0.83 (0.70 to 0.97) | 1,159.60 (977.19 to 1,366.87) | 0.00 (-0.01 to 0.02) | NA | NA | NA | 0.16 (0.11 to 0.22) | 219.52 (151.34 to 307.40) | 0.00 (-0.03 to 0.03) |
| Female | 0.26 (0.22 to 0.31) | 746.95 (623.26 to 883.76) | 0.00 (-0.02 to 0.02) | NA | NA | NA | 0.05 (0.03 to 0.07) | 140.21 (96.16 to 195.27) | 0.00 (-0.04 to 0.04) |
| Male | 0.57 (0.48 to 0.66) | 1,554.50 (1,307.15 to 1,826.17) | 0.00 (-0.02 to 0.03) | NA | NA | NA | 0.11 (0.07 to 0.15) | 295.41 (203.20 to 413.60) | 0.00 (-0.03 to 0.03) |
| Attention-deficit/hyperactivity disorder | | | | | | | | | |
| Total | 2.98 (2.03 to 4.27) | 4,257.60 (2,822.88 to 6,097.30) | -0.01 (-0.07 to 0.07) | 0.02 (0.01 to 0.02) | 23.93 (15.88 to 35.40) | -0.02 (-0.09 to 0.05) | 0.04 (0.02 to 0.06) | 51.90 (26.06 to 88.28) | -0.01 (-0.08 to 0.07) |
| Female | 0.73 (0.50 to 1.05) | 2,118.73 (1,408.62 to 3,060.02) | -0.01 (-0.11 to 0.10) | 0.00 (0.00 to 0.01) | 11.63 (7.70 to 17.35) | -0.02 (-0.12 to 0.09) | 0.01 (0.00 to 0.01) | 25.67 (13.19 to 44.78) | -0.01 (-0.12 to 0.10) |
| Male | 2.26 (1.53 to 3.21) | 6,303.65 (4,170.40 to 9,098.58) | -0.01 (-0.10 to 0.10) | 0.01 (0.01 to 0.02) | 35.70 (23.76 to 52.68) | -0.02 (-0.11 to 0.08) | 0.03 (0.01 to 0.05) | 77.00 (38.54 to 131.39) | -0.01 (-0.10 to 0.10) |
| Conduct disorder | | | | | | | | | |
| Total | 1.24 (0.88 to 1.64) | 1,836.54 (1,243.02 to 2,533.35) | -0.01 (-0.01 to 0.00) | 0.32 (0.20 to 0.44) | 476.46 (282.12 to 693.12) | -0.01 (-0.01 to -0.01) | 0.15 (0.08 to 0.24) | 222.75 (116.22 to 366.55) | -0.01 (-0.03 to 0.01) |
| Female | 0.45 (0.30 to 0.62) | 1,377.71 (876.06 to 2,003.44) | -0.01 (-0.01 to 0.00) | 0.12 (0.07 to 0.18) | 366.83 (204.46 to 567.56) | -0.01 (-0.01 to -0.01) | 0.05 (0.03 to 0.09) | 165.94 (83.60 to 284.38) | -0.01 (-0.04 to 0.02) |
| Male | 0.79 (0.57 to 1.02) | 2,275.15 (1,574.69 to 3,097.42) | -0.01 (-0.01 to 0.00) | 0.20 (0.13 to 0.26) | 581.29 (341.91 to 820.78) | -0.01 (-0.01 to -0.01) | 0.10 (0.05 to 0.15) | 277.06 (147.00 to 447.74) | -0.01 (-0.03 to 0.02) |
| Idiopathic developmental intellectual disability | | | | | | | | | |
| Total | 0.35 (0.06 to 0.64) | 490.55 (84.37 to 895.30) | 0.00 (-0.01 to 0.02) | NA | NA | NA | 0.02 (0.00 to 0.03) | 24.78 (6.82 to 45.89) | 0.00 (-0.05 to 0.04) |
| Female | 0.14 (0.03 to 0.25) | 412.21 (91.52 to 729.30) | 0.00 (-0.01 to 0.02) | NA | NA | NA | 0.01 (0.00 to 0.01) | 20.44 (6.21 to 37.27) | 0.00 (-0.06 to 0.07) |
| Male | 0.21 (0.03 to 0.38) | 565.51 (82.97 to 1,050.56) | 0.00 (-0.03 to 0.02) | NA | NA | NA | 0.01 (0.00 to 0.02) | 28.93 (7.90 to 53.83) | -0.01 (-0.06 to 0.07) |
| Other mental disorders | | | | | | | | | |
| Total | 0.62 (0.43 to 0.83) | 817.66 (569.66 to 1,100.03) | 0.01 (0.00 to 0.01) | NA | NA | NA | 0.05 (0.03 to 0.07) | 62.08 (37.23 to 96.52) | 0.00 (-0.04 to 0.05) |
| Female | 0.20 (0.13 to 0.27) | 529.80 (356.99 to 732.56) | 0.01 (0.01 to 0.01) | NA | NA | NA | 0.01 (0.01 to 0.02) | 39.78 (23.90 to 62.65) | 0.00 (-0.07 to 0.08) |
| Male | 0.42 (0.30 to 0.56) | 1,093.51 (769.60 to 1,466.12) | 0.01 (0.00 to 0.01) | NA | NA | NA | 0.03 (0.02 to 0.05) | 83.44 (50.14 to 129.03) | 0.00 (-0.05 to 0.06) |

**Table S14.** Prevalence, Incidence, and Years Lived with Disability of Mental Disorders Among Adolescents and Young Adults (Aged 10-24) in **North Africa and Middle East** in 2021, and Percentage Change from 2019 to 2021 According to GBD.

|  | Prevalence (95% uncertainty interval) | | | Incidence (95% uncertainty interval) | | | Years lived with disability (95% uncertainty interval) | | |
| --- | --- | --- | --- | --- | --- | --- | --- | --- | --- |
| Cause | Counts in million | Age-standardized rates per 100,000 people | Percentage rate change (2019-2021) | Counts in million | Age-standardized rates per 100,000 people | Percentage rate change (2019-2021) | Counts in million | Age-standardized rates per 100,000 people | Percentage rate change (2019-2021) |
| Mental disorders | | | | | | | | | |
| Total | 31.56 (27.35 to 36.31) | 19,460.64 (16,518.89 to 22,810.79) | 0.11 (0.08 to 0.15) | 13.53 (10.25 to 17.44) | 8,351.26 (5,993.90 to 11,325.21) | 0.21 (0.16 to 0.27) | 4.35 (3.10 to 5.84) | 2,682.57 (1,864.62 to 3,654.80) | 0.14 (0.10 to 0.19) |
| Female | 16.07 (13.85 to 18.91) | 20,479.92 (17,040.88 to 24,474.36) | 0.14 (0.09 to 0.18) | 7.78 (5.87 to 10.15) | 9,912.18 (7,026.49 to 13,597.67) | 0.23 (0.17 to 0.30) | 2.36 (1.67 to 3.17) | 3,014.98 (2,057.66 to 4,135.85) | 0.16 (0.11 to 0.21) |
| Male | 15.49 (13.50 to 17.77) | 18,500.05 (15,876.97 to 21,513.18) | 0.09 (0.06 to 0.12) | 5.76 (4.43 to 7.29) | 6,885.34 (5,012.74 to 9,191.28) | 0.20 (0.14 to 0.26) | 1.98 (1.42 to 2.66) | 2,370.08 (1,670.38 to 3,214.12) | 0.12 (0.08 to 0.17) |
| Schizophrenia | | | | | | | | | |
| Total | 0.16 (0.10 to 0.24) | 96.94 (57.57 to 156.30) | -0.01 (-0.04 to 0.01) | 0.04 (0.02 to 0.06) | 23.59 (12.98 to 38.12) | -0.01 (-0.03 to 0.01) | 0.10 (0.06 to 0.17) | 64.55 (36.56 to 106.84) | -0.02 (-0.09 to 0.06) |
| Female | 0.07 (0.04 to 0.11) | 92.26 (54.59 to 149.85) | -0.01 (-0.05 to 0.02) | 0.02 (0.01 to 0.03) | 22.48 (12.33 to 36.57) | 0.00 (-0.04 to 0.03) | 0.05 (0.03 to 0.08) | 60.73 (34.03 to 103.14) | -0.01 (-0.10 to 0.09) |
| Male | 0.08 (0.05 to 0.13) | 101.35 (60.24 to 161.67) | -0.02 (-0.06 to 0.02) | 0.02 (0.01 to 0.03) | 24.64 (13.55 to 39.49) | -0.02 (-0.05 to 0.02) | 0.06 (0.03 to 0.09) | 68.15 (38.61 to 114.69) | -0.03 (-0.12 to 0.09) |
| Depressive disorders | | | | | | | | | |
| Total | 7.31 (5.29 to 9.82) | 4,519.69 (3,101.58 to 6,396.30) | 0.21 (0.15 to 0.28) | 9.84 (6.78 to 13.66) | 6,081.86 (3,913.09 to 8,938.01) | 0.25 (0.19 to 0.34) | 1.41 (0.84 to 2.15) | 871.45 (505.10 to 1,374.01) | 0.23 (0.16 to 0.31) |
| Female | 4.39 (3.20 to 5.94) | 5,599.23 (3,831.77 to 7,980.11) | 0.21 (0.15 to 0.29) | 5.95 (4.15 to 8.26) | 7,596.63 (4,853.06 to 11,203.97) | 0.26 (0.19 to 0.35) | 0.85 (0.50 to 1.29) | 1,080.10 (621.46 to 1,700.70) | 0.23 (0.16 to 0.32) |
| Male | 2.92 (2.10 to 3.88) | 3,505.85 (2,411.09 to 4,952.12) | 0.20 (0.14 to 0.27) | 3.89 (2.63 to 5.38) | 4,659.66 (3,000.11 to 6,882.81) | 0.25 (0.17 to 0.34) | 0.56 (0.34 to 0.86) | 675.51 (395.20 to 1,057.04) | 0.22 (0.15 to 0.31) |
| Bipolar disorder | | | | | | | | | |
| Total | 1.13 (0.76 to 1.67) | 700.53 (466.29 to 1,044.30) | -0.01 (-0.01 to -0.01) | 0.17 (0.12 to 0.25) | 700.53 (466.29 to 1,044.30) | 0.00 (0.00 to 0.01) | 0.25 (0.15 to 0.41) | 155.37 (89.72 to 253.00) | -0.01 (-0.05 to 0.02) |
| Female | 0.62 (0.42 to 0.92) | 790.05 (526.07 to 1,176.03) | -0.01 (-0.01 to -0.01) | 0.09 (0.06 to 0.13) | 790.05 (526.07 to 1,176.03) | 0.00 (0.00 to 0.01) | 0.14 (0.08 to 0.22) | 173.71 (99.95 to 285.81) | -0.01 (-0.06 to 0.04) |
| Male | 0.51 (0.35 to 0.77) | 616.45 (403.18 to 923.92) | -0.01 (-0.01 to -0.01) | 0.08 (0.05 to 0.12) | 616.45 (403.18 to 923.92) | 0.00 (0.00 to 0.01) | 0.11 (0.07 to 0.19) | 138.14 (79.35 to 224.83) | -0.01 (-0.06 to 0.05) |
| Anxiety disorders | | | | | | | | | |
| Total | 13.25 (9.68 to 18.16) | 8,169.82 (5,691.43 to 11,376.86) | 0.22 (0.13 to 0.31) | 2.06 (1.49 to 2.80) | 1,268.05 (808.12 to 1,821.04) | 0.22 (0.13 to 0.31) | 1.62 (1.00 to 2.40) | 1,001.07 (604.50 to 1,530.39) | 0.22 (0.12 to 0.31) |
| Female | 7.95 (5.79 to 10.74) | 10,130.51 (7,027.74 to 14,063.53) | 0.22 (0.13 to 0.32) | 1.19 (0.84 to 1.65) | 1,512.34 (959.84 to 2,205.77) | 0.22 (0.12 to 0.31) | 0.97 (0.60 to 1.43) | 1,234.53 (739.61 to 1,887.53) | 0.22 (0.12 to 0.32) |
| Male | 5.30 (3.84 to 7.40) | 6,327.94 (4,411.03 to 8,971.38) | 0.22 (0.12 to 0.32) | 0.87 (0.63 to 1.18) | 1,038.70 (678.10 to 1,487.90) | 0.23 (0.14 to 0.31) | 0.65 (0.40 to 0.99) | 781.76 (466.04 to 1,218.60) | 0.22 (0.12 to 0.32) |
| Eating disorders | | | | | | | | | |
| Total | 0.65 (0.44 to 0.95) | 402.21 (247.23 to 637.18) | -0.03 (-0.07 to -0.01) | 0.68 (0.44 to 0.99) | 418.88 (226.80 to 731.55) | -0.01 (-0.03 to 0.02) | 0.14 (0.08 to 0.23) | 85.85 (45.01 to 149.42) | -0.04 (-0.08 to 0.01) |
| Female | 0.42 (0.28 to 0.60) | 531.47 (330.11 to 825.81) | -0.05 (-0.09 to -0.01) | 0.28 (0.19 to 0.40) | 361.30 (204.79 to 605.95) | -0.02 (-0.06 to 0.02) | 0.09 (0.05 to 0.14) | 112.80 (58.77 to 195.39) | -0.05 (-0.11 to 0.01) |
| Male | 0.23 (0.16 to 0.35) | 280.65 (167.61 to 470.35) | -0.01 (-0.04 to 0.02) | 0.40 (0.24 to 0.60) | 472.95 (242.40 to 852.19) | 0.00 (-0.03 to 0.03) | 0.05 (0.03 to 0.08) | 60.51 (30.81 to 109.88) | -0.01 (-0.08 to 0.06) |
| Autism spectrum disorders | | | | | | | | | |
| Total | 1.31 (1.10 to 1.55) | 808.26 (679.79 to 952.54) | 0.00 (-0.02 to 0.02) | NA | NA | NA | 0.25 (0.17 to 0.35) | 153.85 (104.00 to 216.47) | 0.00 (-0.03 to 0.04) |
| Female | 0.44 (0.36 to 0.52) | 555.21 (461.55 to 660.59) | 0.00 (-0.03 to 0.03) | NA | NA | NA | 0.08 (0.06 to 0.12) | 104.92 (71.97 to 146.95) | 0.00 (-0.05 to 0.05) |
| Male | 0.88 (0.74 to 1.03) | 1,045.73 (884.62 to 1,230.78) | 0.00 (-0.03 to 0.03) | NA | NA | NA | 0.17 (0.11 to 0.24) | 199.76 (135.21 to 282.39) | 0.00 (-0.05 to 0.05) |
| Attention-deficit/hyperactivity disorder | | | | | | | | | |
| Total | 3.81 (2.69 to 5.28) | 2,346.35 (1,616.94 to 3,331.77) | 0.00 (-0.03 to 0.02) | 0.02 (0.01 to 0.03) | 12.62 (8.65 to 18.57) | 0.02 (-0.01 to 0.04) | 0.05 (0.02 to 0.08) | 28.67 (14.87 to 48.06) | 0.00 (-0.04 to 0.04) |
| Female | 1.06 (0.75 to 1.45) | 1,351.60 (923.56 to 1,920.20) | -0.01 (-0.04 to 0.04) | 0.01 (0.00 to 0.01) | 7.28 (4.93 to 10.68) | 0.01 (-0.02 to 0.06) | 0.01 (0.01 to 0.02) | 16.43 (8.45 to 28.09) | -0.01 (-0.07 to 0.06) |
| Male | 2.75 (1.95 to 3.83) | 3,279.18 (2,251.29 to 4,669.57) | 0.00 (-0.03 to 0.03) | 0.01 (0.01 to 0.02) | 17.63 (12.10 to 25.84) | 0.02 (-0.02 to 0.05) | 0.03 (0.02 to 0.06) | 40.15 (20.67 to 67.53) | 0.00 (-0.04 to 0.05) |
| Conduct disorder | | | | | | | | | |
| Total | 2.79 (1.97 to 3.63) | 1,708.37 (1,151.85 to 2,349.15) | 0.01 (0.01 to 0.01) | 0.72 (0.46 to 0.99) | 439.11 (255.08 to 641.32) | 0.01 (0.01 to 0.02) | 0.34 (0.18 to 0.54) | 207.68 (108.28 to 333.69) | 0.01 (-0.02 to 0.03) |
| Female | 0.89 (0.59 to 1.23) | 1,129.78 (719.66 to 1,657.85) | 0.01 (0.01 to 0.01) | 0.23 (0.14 to 0.36) | 294.34 (160.02 to 469.15) | 0.01 (0.01 to 0.02) | 0.11 (0.06 to 0.18) | 136.62 (69.83 to 232.44) | 0.01 (-0.03 to 0.06) |
| Male | 1.90 (1.36 to 2.45) | 2,249.96 (1,552.99 to 3,027.56) | 0.01 (0.01 to 0.01) | 0.49 (0.32 to 0.66) | 574.59 (342.36 to 827.40) | 0.01 (0.01 to 0.01) | 0.23 (0.13 to 0.37) | 274.20 (143.48 to 439.75) | 0.01 (-0.02 to 0.04) |
| Idiopathic developmental intellectual disability | | | | | | | | | |
| Total | 2.98 (1.66 to 4.21) | 1,832.91 (1,024.30 to 2,595.11) | 0.02 (-0.05 to 0.09) | NA | NA | NA | 0.12 (0.05 to 0.21) | 74.50 (33.44 to 128.93) | 0.02 (-0.02 to 0.06) |
| Female | 1.28 (0.73 to 1.80) | 1,624.59 (934.08 to 2,295.70) | 0.02 (-0.05 to 0.09) | NA | NA | NA | 0.05 (0.02 to 0.09) | 64.91 (29.94 to 112.85) | 0.02 (-0.03 to 0.07) |
| Male | 1.70 (0.93 to 2.43) | 2,028.31 (1,114.08 to 2,902.43) | 0.02 (-0.05 to 0.10) | NA | NA | NA | 0.07 (0.03 to 0.12) | 83.49 (37.05 to 145.42) | 0.02 (-0.02 to 0.07) |
| Other mental disorders | | | | | | | | | |
| Total | 0.83 (0.53 to 1.15) | 515.99 (330.05 to 718.92) | -0.01 (-0.01 to -0.01) | NA | NA | NA | 0.06 (0.04 to 0.10) | 39.60 (22.92 to 63.35) | -0.01 (-0.08 to 0.05) |
| Female | 0.31 (0.20 to 0.44) | 398.42 (253.56 to 561.13) | -0.01 (-0.01 to -0.01) | NA | NA | NA | 0.02 (0.01 to 0.04) | 30.22 (16.74 to 48.76) | -0.01 (-0.12 to 0.08) |
| Male | 0.52 (0.33 to 0.73) | 626.69 (399.65 to 882.05) | -0.01 (-0.01 to -0.01) | NA | NA | NA | 0.04 (0.02 to 0.06) | 48.42 (27.18 to 77.27) | -0.01 (-0.09 to 0.07) |

**Table S15.** Prevalence, Incidence, and Years Lived with Disability of Mental Disorders Among Adolescents and Young Adults (Aged 10-24) in **Oceania** in 2021, and Percentage Change from 2019 to 2021 According to GBD.

|  | Prevalence (95% uncertainty interval) | | | Incidence (95% uncertainty interval) | | | Years lived with disability (95% uncertainty interval) | | |
| --- | --- | --- | --- | --- | --- | --- | --- | --- | --- |
| Cause | Counts in million | Age-standardized rates per 100,000 people | Percentage rate change (2019-2021) | Counts in million | Age-standardized rates per 100,000 people | Percentage rate change (2019-2021) | Counts in million | Age-standardized rates per 100,000 people | Percentage rate change (2019-2021) |
| Mental disorders | | | | | | | | | |
| Total | 0.53 (0.45 to 0.62) | 13,260.28 (11,012.55 to 15,897.34) | 0.06 (-0.02 to 0.14) | 0.19 (0.15 to 0.25) | 4,759.47 (3,481.64 to 6,556.86) | 0.10 (-0.03 to 0.25) | 0.07 (0.05 to 0.09) | 1,692.64 (1,165.13 to 2,348.62) | 0.07 (-0.02 to 0.18) |
| Female | 0.25 (0.21 to 0.30) | 13,057.03 (10,567.89 to 16,161.29) | 0.08 (-0.03 to 0.19) | 0.10 (0.07 to 0.13) | 4,970.52 (3,580.36 to 6,782.87) | 0.11 (-0.03 to 0.27) | 0.03 (0.02 to 0.05) | 1,752.86 (1,170.91 to 2,472.24) | 0.09 (-0.03 to 0.22) |
| Male | 0.28 (0.24 to 0.33) | 13,440.52 (11,288.23 to 15,908.73) | 0.05 (-0.02 to 0.11) | 0.10 (0.07 to 0.13) | 4,567.68 (3,317.07 to 6,313.38) | 0.10 (-0.04 to 0.25) | 0.03 (0.02 to 0.05) | 1,637.46 (1,148.10 to 2,267.81) | 0.06 (-0.03 to 0.16) |
| Schizophrenia | | | | | | | | | |
| Total | 0.00 (0.00 to 0.00) | 113.42 (64.43 to 185.82) | -0.01 (-0.07 to 0.07) | 0.00 (0.00 to 0.00) | 27.39 (14.57 to 45.58) | 0.00 (-0.07 to 0.07) | 0.00 (0.00 to 0.01) | 75.74 (40.77 to 131.57) | -0.02 (-0.19 to 0.23) |
| Female | 0.00 (0.00 to 0.00) | 104.76 (59.66 to 171.09) | -0.01 (-0.13 to 0.08) | 0.00 (0.00 to 0.00) | 25.26 (13.46 to 41.48) | -0.01 (-0.13 to 0.08) | 0.00 (0.00 to 0.00) | 69.21 (34.37 to 119.86) | -0.02 (-0.28 to 0.35) |
| Male | 0.00 (0.00 to 0.00) | 121.42 (68.65 to 200.52) | 0.00 (-0.10 to 0.10) | 0.00 (0.00 to 0.00) | 29.34 (15.61 to 49.03) | 0.00 (-0.10 to 0.11) | 0.00 (0.00 to 0.00) | 81.76 (40.80 to 145.47) | -0.01 (-0.25 to 0.31) |
| Depressive disorders | | | | | | | | | |
| Total | 0.10 (0.07 to 0.14) | 2,449.92 (1,683.49 to 3,497.68) | 0.09 (-0.06 to 0.26) | 0.13 (0.08 to 0.18) | 3,168.24 (2,008.73 to 4,825.11) | 0.12 (-0.07 to 0.34) | 0.02 (0.01 to 0.03) | 459.13 (260.37 to 736.22) | 0.10 (-0.08 to 0.31) |
| Female | 0.05 (0.04 to 0.07) | 2,698.92 (1,858.18 to 3,828.80) | 0.08 (-0.06 to 0.25) | 0.06 (0.04 to 0.09) | 3,391.74 (2,107.43 to 5,098.77) | 0.11 (-0.08 to 0.33) | 0.01 (0.01 to 0.01) | 496.57 (278.83 to 794.66) | 0.10 (-0.09 to 0.33) |
| Male | 0.05 (0.03 to 0.07) | 2,223.16 (1,512.39 to 3,235.01) | 0.10 (-0.06 to 0.28) | 0.06 (0.04 to 0.09) | 2,966.00 (1,892.90 to 4,587.84) | 0.12 (-0.08 to 0.35) | 0.01 (0.01 to 0.01) | 425.15 (240.71 to 685.24) | 0.11 (-0.10 to 0.33) |
| Bipolar disorder | | | | | | | | | |
| Total | 0.01 (0.00 to 0.01) | 194.03 (119.18 to 303.61) | -0.01 (-0.01 to -0.01) | 0.00 (0.00 to 0.00) | 32.58 (17.83 to 53.63) | 0.00 (-0.01 to 0.00) | 0.00 (0.00 to 0.00) | 43.40 (21.21 to 77.37) | -0.01 (-0.19 to 0.21) |
| Female | 0.00 (0.00 to 0.01) | 195.14 (118.58 to 308.97) | -0.01 (-0.01 to -0.01) | 0.00 (0.00 to 0.00) | 32.99 (18.12 to 55.20) | 0.00 (-0.01 to 0.00) | 0.00 (0.00 to 0.00) | 43.47 (20.41 to 79.49) | 0.00 (-0.23 to 0.30) |
| Male | 0.00 (0.00 to 0.01) | 192.99 (117.39 to 296.85) | -0.01 (-0.01 to -0.01) | 0.00 (0.00 to 0.00) | 32.20 (17.76 to 52.46) | 0.00 (-0.01 to 0.00) | 0.00 (0.00 to 0.00) | 43.34 (19.69 to 77.49) | -0.01 (-0.25 to 0.29) |
| Anxiety disorders | | | | | | | | | |
| Total | 0.20 (0.14 to 0.30) | 5,063.23 (3,256.76 to 7,648.88) | 0.14 (-0.09 to 0.39) | 0.03 (0.02 to 0.05) | 836.30 (496.58 to 1,283.37) | 0.15 (-0.08 to 0.39) | 0.03 (0.01 to 0.04) | 623.79 (339.84 to 1,021.62) | 0.14 (-0.10 to 0.40) |
| Female | 0.12 (0.08 to 0.18) | 6,332.00 (4,050.44 to 9,428.83) | 0.14 (-0.09 to 0.40) | 0.02 (0.01 to 0.03) | 1,020.75 (605.51 to 1,573.93) | 0.15 (-0.09 to 0.41) | 0.01 (0.01 to 0.02) | 776.86 (422.03 to 1,269.90) | 0.14 (-0.10 to 0.42) |
| Male | 0.08 (0.05 to 0.12) | 3,905.70 (2,468.74 to 5,873.18) | 0.13 (-0.08 to 0.37) | 0.01 (0.01 to 0.02) | 668.25 (396.47 to 1,029.81) | 0.14 (-0.08 to 0.39) | 0.01 (0.01 to 0.02) | 484.15 (260.43 to 814.59) | 0.13 (-0.12 to 0.41) |
| Eating disorders | | | | | | | | | |
| Total | 0.01 (0.01 to 0.01) | 191.80 (118.54 to 300.40) | 0.00 (-0.08 to 0.08) | 0.01 (0.01 to 0.02) | 249.70 (133.54 to 425.44) | 0.00 (-0.07 to 0.07) | 0.00 (0.00 to 0.00) | 41.14 (21.44 to 71.28) | 0.00 (-0.14 to 0.18) |
| Female | 0.00 (0.00 to 0.01) | 225.67 (143.96 to 342.12) | 0.00 (-0.09 to 0.11) | 0.00 (0.00 to 0.01) | 185.90 (108.43 to 301.51) | 0.00 (-0.08 to 0.10) | 0.00 (0.00 to 0.00) | 48.12 (25.12 to 81.96) | 0.00 (-0.19 to 0.22) |
| Male | 0.00 (0.00 to 0.01) | 160.74 (93.70 to 266.69) | 0.00 (-0.09 to 0.09) | 0.01 (0.00 to 0.01) | 307.99 (154.21 to 546.72) | 0.00 (-0.09 to 0.10) | 0.00 (0.00 to 0.00) | 34.74 (15.84 to 63.65) | -0.01 (-0.23 to 0.25) |
| Autism spectrum disorders | | | | | | | | | |
| Total | 0.03 (0.02 to 0.03) | 719.94 (605.76 to 862.65) | 0.00 (-0.06 to 0.06) | NA | NA | NA | 0.01 (0.00 to 0.01) | 137.26 (93.38 to 191.57) | 0.00 (-0.09 to 0.10) |
| Female | 0.01 (0.01 to 0.01) | 465.67 (383.49 to 559.92) | 0.00 (-0.08 to 0.09) | NA | NA | NA | 0.00 (0.00 to 0.00) | 88.32 (58.38 to 125.01) | 0.00 (-0.12 to 0.18) |
| Male | 0.02 (0.02 to 0.02) | 951.70 (797.27 to 1,139.49) | 0.00 (-0.09 to 0.08) | NA | NA | NA | 0.00 (0.00 to 0.01) | 181.88 (123.42 to 256.49) | 0.00 (-0.13 to 0.13) |
| Attention-deficit/hyperactivity disorder | | | | | | | | | |
| Total | 0.09 (0.06 to 0.13) | 2,215.24 (1,510.01 to 3,193.78) | 0.00 (0.00 to 0.00) | 0.00 (0.00 to 0.00) | 12.49 (8.54 to 18.62) | 0.02 (0.02 to 0.02) | 0.00 (0.00 to 0.00) | 27.02 (13.54 to 46.45) | 0.00 (-0.08 to 0.09) |
| Female | 0.02 (0.02 to 0.03) | 1,287.70 (861.83 to 1,851.42) | 0.00 (0.00 to 0.01) | 0.00 (0.00 to 0.00) | 7.17 (4.71 to 10.71) | 0.02 (0.02 to 0.02) | 0.00 (0.00 to 0.00) | 15.68 (7.76 to 27.23) | 0.01 (-0.12 to 0.18) |
| Male | 0.06 (0.05 to 0.09) | 3,058.47 (2,066.29 to 4,461.84) | 0.00 (0.00 to 0.00) | 0.00 (0.00 to 0.00) | 17.30 (11.70 to 25.87) | 0.02 (0.02 to 0.02) | 0.00 (0.00 to 0.00) | 37.33 (18.75 to 64.99) | 0.00 (-0.10 to 0.11) |
| Conduct disorder | | | | | | | | | |
| Total | 0.07 (0.05 to 0.09) | 1,709.07 (1,171.46 to 2,362.79) | 0.01 (0.01 to 0.01) | 0.02 (0.01 to 0.02) | 432.78 (259.91 to 627.22) | 0.02 (0.01 to 0.02) | 0.01 (0.00 to 0.01) | 208.47 (105.83 to 346.04) | 0.01 (-0.06 to 0.08) |
| Female | 0.02 (0.02 to 0.03) | 1,217.78 (781.53 to 1,779.90) | 0.01 (0.01 to 0.01) | 0.01 (0.00 to 0.01) | 306.71 (170.26 to 481.75) | 0.02 (0.01 to 0.02) | 0.00 (0.00 to 0.00) | 148.35 (72.55 to 259.32) | 0.01 (-0.10 to 0.13) |
| Male | 0.05 (0.03 to 0.06) | 2,153.40 (1,485.72 to 2,950.42) | 0.01 (0.01 to 0.01) | 0.01 (0.01 to 0.02) | 546.60 (336.06 to 776.84) | 0.02 (0.01 to 0.02) | 0.01 (0.00 to 0.01) | 262.85 (137.78 to 431.91) | 0.01 (-0.08 to 0.09) |
| Idiopathic developmental intellectual disability | | | | | | | | | |
| Total | 0.03 (0.01 to 0.05) | 797.46 (313.25 to 1,269.13) | -0.01 (-0.24 to 0.29) | NA | NA | NA | 0.00 (0.00 to 0.00) | 35.86 (13.12 to 65.17) | -0.01 (-0.14 to 0.10) |
| Female | 0.02 (0.01 to 0.02) | 779.94 (339.18 to 1,208.50) | -0.01 (-0.24 to 0.27) | NA | NA | NA | 0.00 (0.00 to 0.00) | 34.98 (13.21 to 63.35) | -0.01 (-0.16 to 0.15) |
| Male | 0.02 (0.01 to 0.03) | 813.40 (286.56 to 1,316.46) | -0.01 (-0.26 to 0.30) | NA | NA | NA | 0.00 (0.00 to 0.00) | 36.67 (12.56 to 67.00) | -0.02 (-0.18 to 0.14) |
| Other mental disorders | | | | | | | | | |
| Total | 0.02 (0.01 to 0.03) | 530.56 (339.99 to 734.50) | -0.01 (-0.01 to -0.01) | NA | NA | NA | 0.00 (0.00 to 0.00) | 40.82 (22.55 to 67.37) | -0.01 (-0.18 to 0.18) |
| Female | 0.01 (0.00 to 0.01) | 408.20 (259.10 to 576.09) | -0.01 (-0.01 to -0.01) | NA | NA | NA | 0.00 (0.00 to 0.00) | 31.30 (16.05 to 51.83) | -0.01 (-0.24 to 0.31) |
| Male | 0.01 (0.01 to 0.02) | 643.24 (409.75 to 899.04) | -0.01 (-0.01 to -0.01) | NA | NA | NA | 0.00 (0.00 to 0.00) | 49.59 (26.14 to 83.34) | -0.02 (-0.21 to 0.21) |

**Table S16**. Prevalence, Incidence, and Years Lived with Disability of Mental Disorders Among Adolescents and Young Adults (Aged 10-24) in **South Asia** in 2021, and Percentage Change from 2019 to 2021 According to GBD.

|  | Prevalence (95% uncertainty interval) | | | Incidence (95% uncertainty interval) | | | Years lived with disability (95% uncertainty interval) | | |
| --- | --- | --- | --- | --- | --- | --- | --- | --- | --- |
| Cause | Counts in million | Age-standardized rates per 100,000 people | Percentage rate change (2019-2021) | Counts in million | Age-standardized rates per 100,000 people | Percentage rate change (2019-2021) | Counts in million | Age-standardized rates per 100,000 people | Percentage rate change (2019-2021) |
| Mental disorders | | | | | | | | | |
| Total | 72.03 (63.33 to 81.79) | 13,648.31 (11,723.93 to 15,757.15) | 0.09 (0.07 to 0.11) | 26.35 (21.47 to 32.67) | 4,958.01 (3,755.70 to 6,429.84) | 0.26 (0.22 to 0.31) | 8.69 (6.36 to 11.35) | 1,637.22 (1,168.78 to 2,181.73) | 0.14 (0.11 to 0.18) |
| Female | 35.74 (31.34 to 40.90) | 13,955.94 (11,970.52 to 16,150.25) | 0.11 (0.09 to 0.14) | 14.39 (11.60 to 18.06) | 5,573.59 (4,167.87 to 7,300.37) | 0.30 (0.24 to 0.35) | 4.42 (3.18 to 5.83) | 1,715.19 (1,212.56 to 2,304.31) | 0.17 (0.13 to 0.21) |
| Male | 36.29 (31.85 to 40.92) | 13,350.14 (11,494.50 to 15,373.29) | 0.07 (0.05 to 0.09) | 11.96 (9.68 to 14.55) | 4,376.07 (3,334.37 to 5,610.07) | 0.23 (0.18 to 0.28) | 4.27 (3.13 to 5.56) | 1,562.93 (1,122.98 to 2,079.21) | 0.11 (0.08 to 0.14) |
| Schizophrenia | | | | | | | | | |
| Total | 0.58 (0.38 to 0.81) | 105.67 (68.19 to 154.25) | 0.00 (-0.02 to 0.02) | 0.14 (0.10 to 0.19) | 25.70 (15.34 to 38.26) | 0.00 (-0.02 to 0.02) | 0.38 (0.24 to 0.58) | 70.36 (42.14 to 109.73) | 0.00 (-0.06 to 0.06) |
| Female | 0.24 (0.16 to 0.34) | 91.61 (59.12 to 134.79) | 0.00 (-0.02 to 0.03) | 0.06 (0.04 to 0.08) | 22.20 (13.24 to 33.14) | 0.00 (-0.03 to 0.03) | 0.16 (0.10 to 0.24) | 60.08 (35.85 to 94.56) | -0.01 (-0.08 to 0.08) |
| Male | 0.33 (0.22 to 0.47) | 119.09 (77.17 to 172.93) | 0.00 (-0.03 to 0.03) | 0.08 (0.06 to 0.11) | 29.02 (17.33 to 42.92) | 0.00 (-0.03 to 0.02) | 0.22 (0.14 to 0.34) | 80.17 (48.50 to 124.99) | 0.00 (-0.08 to 0.08) |
| Depressive disorders | | | | | | | | | |
| Total | 14.59 (11.41 to 18.64) | 2,714.21 (2,011.98 to 3,664.80) | 0.26 (0.20 to 0.32) | 18.28 (13.44 to 24.48) | 3,411.63 (2,382.34 to 4,774.01) | 0.35 (0.29 to 0.42) | 2.67 (1.68 to 3.87) | 496.53 (310.42 to 750.66) | 0.31 (0.24 to 0.37) |
| Female | 8.53 (6.65 to 10.96) | 3,265.26 (2,421.31 to 4,408.53) | 0.26 (0.20 to 0.32) | 10.70 (7.92 to 14.35) | 4,115.17 (2,878.17 to 5,784.27) | 0.36 (0.29 to 0.43) | 1.55 (0.97 to 2.26) | 594.21 (371.93 to 895.26) | 0.31 (0.24 to 0.38) |
| Male | 6.06 (4.68 to 7.76) | 2,193.91 (1,618.56 to 2,955.36) | 0.25 (0.19 to 0.31) | 7.58 (5.54 to 10.13) | 2,748.77 (1,913.88 to 3,852.13) | 0.34 (0.28 to 0.41) | 1.12 (0.70 to 1.63) | 404.37 (250.23 to 613.91) | 0.30 (0.23 to 0.37) |
| Bipolar disorder | | | | | | | | | |
| Total | 1.29 (0.95 to 1.75) | 238.42 (170.50 to 330.52) | 0.00 (0.00 to 0.01) | 0.21 (0.16 to 0.29) | 40.50 (25.79 to 59.35) | 0.00 (0.00 to 0.00) | 0.29 (0.18 to 0.44) | 53.13 (31.68 to 83.75) | 0.00 (-0.05 to 0.04) |
| Female | 0.57 (0.42 to 0.78) | 218.02 (155.21 to 302.67) | 0.00 (0.00 to 0.01) | 0.09 (0.07 to 0.13) | 36.96 (23.54 to 54.37) | 0.00 (0.00 to 0.00) | 0.13 (0.08 to 0.19) | 48.08 (28.63 to 75.56) | 0.00 (-0.06 to 0.08) |
| Male | 0.72 (0.52 to 0.97) | 257.76 (184.47 to 356.63) | 0.00 (0.00 to 0.01) | 0.12 (0.09 to 0.16) | 43.82 (27.98 to 64.13) | 0.00 (0.00 to 0.00) | 0.16 (0.10 to 0.25) | 57.92 (34.28 to 91.08) | 0.00 (-0.07 to 0.06) |
| Anxiety disorders | | | | | | | | | |
| Total | 16.95 (13.18 to 21.19) | 3,189.04 (2,353.15 to 4,202.71) | 0.29 (0.22 to 0.36) | 3.48 (2.38 to 4.62) | 658.17 (437.11 to 902.18) | 0.29 (0.22 to 0.36) | 2.07 (1.32 to 2.97) | 389.89 (239.70 to 571.16) | 0.28 (0.22 to 0.36) |
| Female | 10.21 (8.03 to 12.76) | 3,967.29 (2,946.60 to 5,199.45) | 0.29 (0.23 to 0.37) | 1.99 (1.36 to 2.69) | 780.52 (516.11 to 1,074.62) | 0.29 (0.22 to 0.37) | 1.24 (0.80 to 1.79) | 481.88 (297.09 to 705.22) | 0.29 (0.22 to 0.38) |
| Male | 6.74 (5.12 to 8.54) | 2,458.67 (1,783.63 to 3,272.62) | 0.28 (0.21 to 0.35) | 1.48 (1.02 to 1.97) | 543.67 (360.58 to 742.84) | 0.28 (0.21 to 0.36) | 0.83 (0.53 to 1.20) | 303.59 (185.65 to 450.42) | 0.27 (0.20 to 0.35) |
| Eating disorders | | | | | | | | | |
| Total | 1.54 (1.04 to 2.29) | 286.25 (176.24 to 452.84) | 0.01 (0.00 to 0.03) | 1.93 (1.19 to 2.95) | 361.35 (192.00 to 628.92) | 0.01 (-0.01 to 0.02) | 0.33 (0.18 to 0.53) | 61.12 (31.91 to 106.31) | 0.01 (-0.02 to 0.05) |
| Female | 0.92 (0.63 to 1.34) | 349.67 (220.08 to 536.17) | 0.01 (-0.01 to 0.04) | 0.74 (0.48 to 1.07) | 286.18 (163.56 to 468.34) | 0.01 (-0.01 to 0.04) | 0.19 (0.11 to 0.32) | 74.15 (39.90 to 123.70) | 0.01 (-0.03 to 0.06) |
| Male | 0.63 (0.40 to 0.97) | 226.13 (131.55 to 386.68) | 0.02 (-0.01 to 0.04) | 1.19 (0.70 to 1.89) | 432.04 (216.23 to 776.98) | 0.00 (-0.03 to 0.02) | 0.13 (0.07 to 0.22) | 48.77 (24.26 to 89.00) | 0.01 (-0.05 to 0.07) |
| Autism spectrum disorders | | | | | | | | | |
| Total | 3.87 (3.26 to 4.52) | 735.65 (619.61 to 859.36) | 0.00 (-0.01 to 0.01) | NA | NA | NA | 0.74 (0.50 to 1.04) | 140.01 (95.59 to 197.23) | 0.00 (-0.03 to 0.03) |
| Female | 1.28 (1.07 to 1.52) | 502.51 (420.59 to 599.51) | 0.00 (-0.02 to 0.03) | NA | NA | NA | 0.24 (0.17 to 0.34) | 94.74 (65.29 to 133.27) | 0.00 (-0.04 to 0.04) |
| Male | 2.59 (2.18 to 3.02) | 954.02 (803.75 to 1,112.74) | 0.00 (-0.02 to 0.02) | NA | NA | NA | 0.50 (0.34 to 0.70) | 182.42 (124.50 to 256.20) | 0.00 (-0.04 to 0.03) |
| Attention-deficit/hyperactivity disorder | | | | | | | | | |
| Total | 5.93 (4.04 to 8.47) | 1,137.87 (757.51 to 1,632.72) | 0.00 (0.00 to 0.00) | 0.03 (0.02 to 0.05) | 6.16 (4.10 to 9.10) | -0.01 (-0.01 to 0.00) | 0.07 (0.04 to 0.12) | 13.87 (7.03 to 23.45) | 0.00 (-0.03 to 0.03) |
| Female | 1.68 (1.15 to 2.43) | 667.79 (441.83 to 967.16) | 0.00 (0.00 to 0.00) | 0.01 (0.01 to 0.01) | 3.60 (2.36 to 5.37) | -0.01 (-0.01 to -0.01) | 0.02 (0.01 to 0.03) | 8.08 (4.05 to 14.04) | 0.00 (-0.06 to 0.05) |
| Male | 4.25 (2.89 to 5.99) | 1,576.57 (1,046.02 to 2,263.24) | 0.00 (0.00 to 0.00) | 0.02 (0.01 to 0.03) | 8.50 (5.57 to 12.48) | -0.01 (-0.01 to 0.00) | 0.05 (0.03 to 0.09) | 19.28 (9.69 to 32.69) | 0.00 (-0.04 to 0.04) |
| Conduct disorder | | | | | | | | | |
| Total | 8.98 (6.17 to 11.88) | 1,760.72 (1,192.77 to 2,455.51) | 0.00 (-0.01 to 0.00) | 2.28 (1.46 to 3.09) | 454.50 (265.99 to 654.81) | -0.01 (-0.01 to -0.01) | 1.09 (0.58 to 1.74) | 213.80 (111.05 to 350.25) | -0.01 (-0.02 to 0.01) |
| Female | 3.10 (2.04 to 4.27) | 1,266.78 (799.73 to 1,863.07) | 0.00 (-0.01 to 0.00) | 0.79 (0.49 to 1.16) | 328.95 (185.48 to 505.74) | -0.01 (-0.01 to -0.01) | 0.37 (0.19 to 0.61) | 152.57 (76.84 to 260.51) | -0.01 (-0.04 to 0.03) |
| Male | 5.88 (4.15 to 7.68) | 2,218.15 (1,509.71 to 3,055.19) | 0.00 (-0.01 to 0.00) | 1.49 (0.97 to 2.01) | 570.24 (333.77 to 815.28) | -0.01 (-0.01 to -0.01) | 0.72 (0.38 to 1.14) | 270.50 (140.35 to 436.96) | -0.01 (-0.03 to 0.02) |
| Idiopathic developmental intellectual disability | | | | | | | | | |
| Total | 20.30 (13.09 to 27.44) | 3,868.20 (2,494.91 to 5,226.23) | 0.00 (-0.05 to 0.03) | NA | NA | NA | 0.87 (0.46 to 1.40) | 166.44 (87.21 to 269.02) | 0.00 (-0.01 to 0.02) |
| Female | 10.47 (6.94 to 13.92) | 4,125.41 (2,742.15 to 5,478.96) | 0.00 (-0.03 to 0.03) | NA | NA | NA | 0.45 (0.25 to 0.71) | 176.51 (95.94 to 281.19) | 0.00 (-0.02 to 0.03) |
| Male | 9.83 (6.09 to 13.51) | 3,627.55 (2,247.65 to 4,987.61) | -0.01 (-0.05 to 0.04) | NA | NA | NA | 0.43 (0.21 to 0.70) | 157.03 (78.82 to 257.95) | 0.00 (-0.02 to 0.03) |
| Other mental disorders | | | | | | | | | |
| Total | 2.27 (1.43 to 3.26) | 418.30 (262.96 to 598.47) | 0.00 (0.00 to 0.01) | NA | NA | NA | 0.17 (0.09 to 0.28) | 32.07 (17.51 to 51.56) | 0.00 (-0.05 to 0.05) |
| Female | 0.87 (0.53 to 1.25) | 328.37 (200.67 to 471.64) | 0.01 (0.00 to 0.01) | NA | NA | NA | 0.07 (0.04 to 0.11) | 24.90 (13.35 to 40.49) | 0.00 (-0.08 to 0.08) |
| Male | 1.40 (0.89 to 2.00) | 503.83 (319.85 to 721.84) | 0.00 (0.00 to 0.01) | NA | NA | NA | 0.11 (0.06 to 0.17) | 38.89 (22.10 to 62.75) | 0.00 (-0.06 to 0.07) |

**Table S17.** Prevalence, Incidence, and Years Lived with Disability of Mental Disorders Among Adolescents and Young Adults (Aged 10-24) in **Southeast Asia** in 2021, and Percentage Change from 2019 to 2021 According to GBD.

|  | Prevalence (95% uncertainty interval) | | | Incidence (95% uncertainty interval) | | | Years lived with disability (95% uncertainty interval) | | |
| --- | --- | --- | --- | --- | --- | --- | --- | --- | --- |
| Cause | Counts in million | Age-standardized rates per 100,000 people | Percentage rate change (2019-2021) | Counts in million | Age-standardized rates per 100,000 people | Percentage rate change (2019-2021) | Counts in million | Age-standardized rates per 100,000 people | Percentage rate change (2019-2021) |
| Mental disorders | | | | | | | | | |
| Total | 21.49 (19.21 to 24.09) | 12,546.36 (10,833.21 to 14,446.59) | 0.13 (0.10 to 0.16) | 7.90 (6.42 to 9.74) | 4,599.47 (3,482.40 to 5,919.95) | 0.27 (0.21 to 0.32) | 2.83 (2.06 to 3.74) | 1,644.17 (1,166.10 to 2,213.48) | 0.17 (0.13 to 0.21) |
| Female | 10.76 (9.48 to 12.23) | 12,842.22 (10,888.21 to 15,040.42) | 0.17 (0.13 to 0.21) | 4.19 (3.39 to 5.21) | 4,991.09 (3,746.54 to 6,477.03) | 0.29 (0.23 to 0.35) | 1.47 (1.05 to 1.98) | 1,747.03 (1,223.12 to 2,370.65) | 0.20 (0.16 to 0.24) |
| Male | 10.73 (9.56 to 12.02) | 12,259.80 (10,636.40 to 14,067.02) | 0.10 (0.08 to 0.12) | 3.71 (3.04 to 4.56) | 4,225.51 (3,189.49 to 5,473.31) | 0.24 (0.19 to 0.3) | 1.36 (1.00 to 1.77) | 1,545.71 (1,104.78 to 2,071.73) | 0.14 (0.10 to 0.18) |
| Schizophrenia | | | | | | | | | |
| Total | 0.23 (0.15 to 0.32) | 126.56 (80.75 to 187.76) | -0.02 (-0.05 to 0.01) | 0.05 (0.04 to 0.07) | 30.38 (17.84 to 45.89) | -0.02 (-0.04 to 0.00) | 0.15 (0.10 to 0.23) | 85.28 (50.78 to 136.57) | -0.02 (-0.07 to 0.03) |
| Female | 0.10 (0.07 to 0.14) | 115.68 (73.54 to 171.77) | -0.03 (-0.07 to 0.00) | 0.02 (0.02 to 0.03) | 27.78 (16.30 to 42.00) | -0.03 (-0.06 to 0.00) | 0.07 (0.04 to 0.10) | 77.22 (45.85 to 122.23) | -0.03 (-0.10 to 0.05) |
| Male | 0.12 (0.08 to 0.18) | 137.02 (87.43 to 202.86) | -0.01 (-0.05 to 0.02) | 0.03 (0.04 to 0.02) | 32.88 (19.31 to 49.60) | -0.01 (-0.04 to 0.02) | 0.08 (0.05 to 0.13) | 93.02 (55.07 to 149.93) | -0.01 (-0.08 to 0.06) |
| Depressive disorders | | | | | | | | | |
| Total | 3.98 (3.05 to 5.22) | 2,285.91 (1,634.27 to 3,138.92) | 0.26 (0.19 to 0.33) | 5.02 (3.58 to 6.87) | 2,894.85 (1,911.72 to 4,150.12) | 0.35 (0.28 to 0.42) | 0.74 (0.47 to 1.09) | 427.62 (258.59 to 652.43) | 0.30 (0.23 to 0.38) |
| Female | 2.23 (1.71 to 2.95) | 2,615.09 (1,871.26 to 3,584.73) | 0.25 (0.19 to 0.33) | 2.76 (1.97 to 3.8) | 3,260.19 (2,152.21 to 4,664.58) | 0.35 (0.28 to 0.42) | 0.41 (0.26 to 0.6) | 482.33 (291.76 to 741.18) | 0.30 (0.23 to 0.38) |
| Male | 1.76 (1.34 to 2.29) | 1,971.49 (1,399.71 to 2,702.28) | 0.27 (0.19 to 0.33) | 2.26 (1.60 to 3.08) | 2,546.63 (1,696.45 to 3,651.69) | 0.35 (0.27 to 0.42) | 0.33 (0.21 to 0.50) | 375.41 (227.97 to 576.31) | 0.31 (0.23 to 0.38) |
| Bipolar disorder | | | | | | | | | |
| Total | 0.41 (0.29 to 0.57) | 230.41 (156.66 to 329.80) | 0.00 (0.00 to 0.00) | 0.07 (0.05 to 0.09) | 38.70 (23.75 to 57.86) | 0.00 (0.00 to 0.00) | 0.09 (0.05 to 0.14) | 51.67 (30.23 to 83.43) | 0.00 (-0.05 to 0.04) |
| Female | 0.20 (0.14 to 0.28) | 233.55 (158.90 to 334.15) | 0.00 (0.00 to 0.00) | 0.03 (0.02 to 0.05) | 39.32 (24.13 to 58.82) | 0.00 (0.00 to 0.00) | 0.04 (0.03 to 0.07) | 52.08 (30.36 to 83.86) | 0.00 (-0.06 to 0.06) |
| Male | 0.20 (0.15 to 0.29) | 227.40 (154.58 to 324.48) | 0.00 (0.00 to 0.00) | 0.03 (0.02 to 0.05) | 38.10 (23.36 to 57.03) | 0.00 (0.00 to 0.00) | 0.05 (0.03 to 0.07) | 51.27 (30.05 to 82.90) | -0.01 (-0.07 to 0.07) |
| Anxiety disorders | | | | | | | | | |
| Total | 7.99 (6.17 to 10.24) | 4,642.54 (3,384.46 to 6,214.73) | 0.32 (0.26 to 0.40) | 1.45 (1.03 to 1.97) | 851.32 (554.08 to 1,186.33) | 0.33 (0.26 to 0.40) | 0.99 (0.64 to 1.47) | 572.91 (358.26 to 862.30) | 0.32 (0.25 to 0.39) |
| Female | 5.00 (3.84 to 6.39) | 5,946.33 (4,353.83 to 7,972.58) | 0.33 (0.26 to 0.41) | 0.89 (0.63 to 1.21) | 1,067.88 (695.67 to 1,492.43) | 0.33 (0.26 to 0.41) | 0.61 (0.40 to 0.91) | 730.72 (457.82 to 1,095.79) | 0.33 (0.26 to 0.41) |
| Male | 2.99 (2.27 to 3.87) | 3,398.33 (2,462.73 to 4,567.09) | 0.31 (0.25 to 0.39) | 0.56 (0.40 to 0.76) | 644.89 (418.08 to 897.99) | 0.32 (0.25 to 0.39) | 0.37 (0.24 to 0.55) | 422.32 (261.42 to 636.89) | 0.31 (0.24 to 0.39) |
| Eating disorders | | | | | | | | | |
| Total | 0.43 (0.29 to 0.62) | 243.86 (151.14 to 384.09) | 0.02 (0.00 to 0.03) | 0.54 (0.34 to 0.83) | 310.28 (166.69 to 540.41) | 0.01 (-0.01 to 0.03) | 0.09 (0.05 to 0.15) | 52.54 (27.53 to 89.79) | 0.02 (-0.02 to 0.06) |
| Female | 0.25 (0.17 to 0.35) | 286.54 (182.64 to 435.45) | 0.02 (-0.01 to 0.04) | 0.20 (0.13 to 0.28) | 234.58 (136.96 to 380.95) | 0.01 (-0.02 to 0.03) | 0.05 (0.03 to 0.09) | 61.43 (33.02 to 101.57) | 0.02 (-0.04 to 0.07) |
| Male | 0.18 (0.12 to 0.28) | 202.99 (119.15 to 340.21) | 0.02 (-0.01 to 0.04) | 0.34 (0.20 to 0.54) | 382.60 (193.72 to 693.23) | 0.01 (-0.01 to 0.04) | 0.04 (0.02 to 0.07) | 44.02 (21.85 to 80.05) | 0.01 (-0.05 to 0.09) |
| Autism spectrum disorders | | | | | | | | | |
| Total | 1.24 (1.04 to 1.46) | 723.29 (607.04 to 855.18) | 0.00 (-0.02 to 0.01) | NA | NA | NA | 0.24 (0.16 to 0.33) | 138.62 (94.25 to 195.51) | 0.00 (-0.03 to 0.02) |
| Female | 0.41 (0.34 to 0.49) | 487.58 (406.17 to 584.91) | 0.00 (-0.02 to 0.02) | NA | NA | NA | 0.08 (0.05 to 0.11) | 92.97 (63.19 to 131.96) | 0.00 (-0.04 to 0.04) |
| Male | 0.83 (0.70 to 0.98) | 947.91 (797.77 to 1,117.01) | 0.00 (-0.03 to 0.01) | NA | NA | NA | 0.16 (0.11 to 0.22) | 182.13 (125.03 to 256.41) | 0.00 (-0.04 to 0.03) |
| Attention-deficit/hyperactivity disorder | | | | | | | | | |
| Total | 3.12 (2.16 to 4.35) | 1,842.73 (1,247.05 to 2,646.89) | 0.00 (0.00 to 0.00) | 0.02 (0.01 to 0.02) | 10.11 (6.79 to 14.91) | 0.00 (0.00 to 0.00) | 0.04 (0.02 to 0.06) | 22.55 (11.55 to 38.70) | 0 (-0.03 to 0.02) |
| Female | 0.91 (0.63 to 1.29) | 1,101.93 (735.91 to 1,618.44) | 0.00 (0.00 to 0.00) | 0.00 (0.00 to 0.01) | 6.00 (4.00 to 9.01) | 0.00 (0.00 to 0.00) | 0.01 (0.01 to 0.02) | 13.42 (6.88 to 23.11) | 0 (-0.05 to 0.04) |
| Male | 2.21 (1.54 to 3.07) | 2,547.50 (1,729.65 to 3,641.75) | 0.00 (0.00 to 0.00) | 0.01 (0.01 to 0.02) | 13.99 (9.38 to 20.58) | 0.00 (0.00 to 0.00) | 0.03 (0.01 to 0.05) | 31.23 (15.97 to 53.74) | 0 (-0.03 to 0.03) |
| Conduct disorder | | | | | | | | | |
| Total | 3.03 (2.15 to 3.95) | 1,832.36 (1,251.24 to 2,537.22) | 0.00 (0.00 to 0.00) | 0.76 (0.50 to 1.03) | 463.83 (281.06 to 667.29) | 0.00 (0.00 to 0.00) | 0.37 (0.20 to 0.59) | 223.99 (117.40 to 367.51) | 0 (-0.02 to 0.02) |
| Female | 1.13 (0.77 to 1.52) | 1,411.90 (917.94 to 2,043.76) | 0.00 (0.00 to 0.00) | 0.28 (0.17 to 0.41) | 355.34 (208.98 to 536.75) | 0.00 (0.00 to 0.01) | 0.14 (0.07 to 0.22) | 171.84 (88.82 to 289.58) | 0 (-0.03 to 0.03) |
| Male | 1.90 (1.37 to 2.44) | 2,231.13 (1,556.66 to 3,027.08) | 0.00 (0.00 to 0.00) | 0.48 (0.32 to 0.65) | 566.42 (342.43 to 799.06) | 0.00 (0.00 to 0.00) | 0.23 (0.13 to 0.37) | 273.45 (145.77 to 440.77) | 0 (-0.02 to 0.02) |
| Idiopathic developmental intellectual disability | | | | | | | | | |
| Total | 1.39 (0.63 to 2.14) | 817.62 (367.56 to 1,254.66) | -0.03 (-0.08 to 0.02) | NA | NA | NA | 0.06 (0.02 to 0.11) | 33.83 (13.07 to 62.57) | -0.03 (-0.06 to 0.00) |
| Female | 0.78 (0.40 to 1.14) | 940.32 (484.58 to 1,374.26) | -0.03 (-0.08 to 0.02) | NA | NA | NA | 0.03 (0.01 to 0.06) | 37.79 (15.76 to 68.25) | -0.03 (-0.07 to 0.01) |
| Male | 0.61 (0.22 to 1.00) | 700.73 (256.44 to 1,140.81) | -0.03 (-0.09 to 0.02) | NA | NA | NA | 0.03 (0.01 to 0.05) | 30.07 (9.97 to 57.88) | -0.03 (-0.08 to 0.01) |
| Other mental disorders | | | | | | | | | |
| Total | 0.81 (0.52 to 1.16) | 454.47 (290.14 to 650.82) | 0.00 (0.00 to 0.00) | NA | NA | NA | 0.06 (0.04 to 0.10) | 35.17 (19.89 to 56.26) | 0.00 (-0.06 to 0.05) |
| Female | 0.31 (0.19 to 0.44) | 354.66 (224.28 to 505.84) | 0.00 (-0.01 to 0.00) | NA | NA | NA | 0.02 (0.01 to 0.04) | 27.23 (14.83 to 43.91) | -0.01 (-0.08 to 0.08) |
| Male | 0.50 (0.32 to 0.72) | 550.27 (352.97 to 790.56) | 0.00 (0.00 to 0.00) | NA | NA | NA | 0.04 (0.02 to 0.06) | 42.79 (24.55 to 68.18) | 0.00 (-0.07 to 0.06) |

**Table S18.** Prevalence, Incidence, and Years Lived with Disability of Mental Disorders Among Adolescents and Young Adults (Aged 10-24) in **Southern Latin America** in 2021, and Percentage Change from 2019 to 2021 According to GBD.

|  | Prevalence (95% uncertainty interval) | | | Incidence (95% uncertainty interval) | | | Years lived with disability (95% uncertainty interval) | | |
| --- | --- | --- | --- | --- | --- | --- | --- | --- | --- |
| Cause | Counts in million | Age-standardized rates per 100,000 people | Percentage rate change (2019-2021) | Counts in million | Age-standardized rates per 100,000 people | Percentage rate change (2019-2021) | Counts in million | Age-standardized rates per 100,000 people | Percentage rate change (2019-2021) |
| Mental disorders | | | | | | | | | |
| Total | 2.92 (2.50 to 3.42) | 18,954.34 (15,845.17 to 22,448.66) | 0.17 (0.07 to 0.3) | 1.29 (1.03 to 1.60) | 8,350.64 (6,380.59 to 10,807.87) | 0.31 (0.15 to 0.47) | 0.42 (0.30 to 0.57) | 2,730.55 (1,897.30 to 3,728.62) | 0.20 (0.09 to 0.34) |
| Female | 1.58 (1.32 to 1.88) | 20,637.90 (16,920.36 to 25,165.94) | 0.22 (0.10 to 0.38) | 0.82 (0.65 to 1.02) | 10,781.91 (8,000.90 to 14,189.88) | 0.33 (0.18 to 0.51) | 0.25 (0.17 to 0.34) | 3,196.96 (2,170.11 to 4,410.00) | 0.24 (0.11 to 0.40) |
| Male | 1.35 (1.17 to 1.55) | 17,311.94 (14,708.96 to 20,266.40) | 0.12 (0.04 to 0.22) | 0.47 (0.38 to 0.57) | 5,989.02 (4,559.41 to 7,703.81) | 0.27 (0.12 to 0.42) | 0.18 (0.13 to 0.24) | 2,276.78 (1,601.04 to 3,065.67) | 0.15 (0.05 to 0.28) |
| Schizophrenia | | | | | | | | | |
| Total | 0.02 (0.01 to 0.03) | 98.26 (55.72 to 164.88) | -0.01 (-0.07 to 0.06) | 0.00 (0.00 to 0.01) | 24.53 (12.84 to 40.80) | 0.00 (-0.07 to 0.06) | 0.01 (0.01 to 0.02) | 65.08 (33.69 to 110.51) | -0.02 (-0.19 to 0.19) |
| Female | 0.01 (0.00 to 0.01) | 90.62 (49.70 to 152.23) | -0.01 (-0.12 to 0.09) | 0.00 (0.00 to 0.00) | 22.79 (11.68 to 38.21) | -0.01 (-0.11 to 0.11) | 0.00 (0.00 to 0.01) | 59.16 (29.06 to 104.69) | -0.03 (-0.26 to 0.27) |
| Male | 0.01 (0.01 to 0.01) | 105.71 (61.40 to 175.38) | 0.00 (-0.09 to 0.10) | 0.00 (0.00 to 0.00) | 26.22 (13.92 to 43.62) | 0.00 (-0.09 to 0.10) | 0.01 (0.00 to 0.01) | 70.85 (35.27 to 122.37) | -0.01 (-0.21 to 0.29) |
| Depressive disorders | | | | | | | | | |
| Total | 0.67 (0.50 to 0.86) | 4,224.28 (3,006.31 to 5,702.86) | 0.33 (0.15 to 0.51) | 0.92 (0.66 to 1.23) | 5,871.48 (4,001.22 to 8,198.97) | 0.38 (0.18 to 0.59) | 0.13 (0.08 to 0.19) | 829.34 (508.29 to 1,257.93) | 0.32 (0.11 to 0.56) |
| Female | 0.44 (0.32 to 0.56) | 5,619.59 (3,961.26 to 7,691.35) | 0.35 (0.16 to 0.55) | 0.62 (0.45 to 0.81) | 7,984.50 (5,422.97 to 11,171.32) | 0.39 (0.17 to 0.62) | 0.09 (0.05 to 0.13) | 1,113.88 (672.56 to 1,705.02) | 0.36 (0.16 to 0.59) |
| Male | 0.23 (0.17 to 0.29) | 2,868.37 (2,065.52 to 3,819.79) | 0.29 (0.11 to 0.48) | 0.30 (0.22 to 0.41) | 3,819.31 (2,604.28 to 5,375.70) | 0.36 (0.13 to 0.59) | 0.04 (0.03 to 0.07) | 552.87 (336.65 to 852.27) | 0.35 (0.17 to 0.55) |
| Bipolar disorder | | | | | | | | | |
| Total | 0.11 (0.07 to 0.17) | 700.04 (454.93 to 1,052.12) | -0.01 (-0.01 to -0.01) | 0.02 (0.01 to 0.02) | 102.83 (63.13 to 161.99) | 0.00 (0.00 to 0.00) | 0.02 (0.01 to 0.04) | 155.22 (87.18 to 255.47) | -0.01 (-0.10 to 0.09) |
| Female | 0.06 (0.04 to 0.09) | 719.55 (453.21 to 1,083.50) | -0.01 (-0.01 to -0.01) | 0.01 (0.01 to 0.01) | 109.56 (64.24 to 174.92) | 0.00 (0.00 to 0.00) | 0.01 (0.01 to 0.02) | 157.89 (84.41 to 263.50) | -0.02 (-0.14 to 0.13) |
| Male | 0.05 (0.04 to 0.08) | 680.86 (443.58 to 999.37) | -0.01 (-0.01 to -0.01) | 0.01 (0.00 to 0.01) | 96.25 (60.22 to 149.62) | 0.00 (0.00 to 0.00) | 0.01 (0.01 to 0.02) | 152.59 (81.75 to 254.65) | -0.01 (-0.14 to 0.13) |
| Anxiety disorders | | | | | | | | | |
| Total | 1.24 (0.82 to 1.73) | 8,018.79 (5,131.46 to 11,590.21) | 0.35 (0.05 to 0.67) | 0.20 (0.13 to 0.29) | 1,329.94 (805.67 to 1,994.80) | 0.36 (0.05 to 0.69) | 0.15 (0.09 to 0.23) | 983.16 (548.68 to 1,533.60) | 0.34 (0.04 to 0.67) |
| Female | 0.80 (0.54 to 1.10) | 10,456.24 (6,762.51 to 14,980.48) | 0.36 (0.07 to 0.67) | 0.13 (0.08 to 0.18) | 1,704.89 (1,020.60 to 2,553.51) | 0.37 (0.05 to 0.75) | 0.10, (0.06 to 0.15) | 1,276.40 (724.95 to 1,970.70) | 0.36 (0.05 to 0.66) |
| Male | 0.44 (0.29 to 0.62) | 5,649.77 (3,598.12 to 8,386.36) | 0.32 (0.01 to 0.69) | 0.07 (0.05 to 0.11) | 966.08 (580.38 to 1,468.96) | 0.33 (0.02 to 0.68) | 0.05 (0.03 to 0.09) | 698.19 (378.81 to 1,132.91) | 0.32 (-0.01 to 0.72) |
| Eating disorders | | | | | | | | | |
| Total | 0.11 (0.07 to 0.15) | 662.16 (421.58 to 1,013.60) | -0.02 (-0.07 to 0.04) | 0.08 (0.05 to 0.11) | 507.28 (279.29 to 860.63) | -0.01 (-0.07 to 0.06) | 0.02 (0.01 to 0.04) | 141.49 (74.95 to 239.60) | -0.02 (-0.11 to 0.07) |
| Female | 0.07 (0.05 to 0.1) | 937.87 (610.82 to 1,450.71) | -0.02 (-0.09 to 0.06) | 0.04 (0.03 to 0.06) | 558.03 (321.38 to 916.21) | -0.01 (-0.10 to 0.08) | 0.02 (0.01 to 0.03) | 199.73 (106.13 to 341.58) | -0.02 (-0.13 to 0.10) |
| Male | 0.03 (0.02 to 0.05) | 393.67 (231.67 to 656.83) | -0.02 (-0.09 to 0.06) | 0.04 (0.02 to 0.06) | 458.10 (237.08 to 815.85) | 0.00 (-0.08 to 0.08) | 0.01 (0.00 to 0.01) | 84.79 (41.57 to 150.74) | -0.02 (-0.17 to 0.15) |
| Autism spectrum disorders | | | | | | | | | |
| Total | 0.17 (0.14 to 0.20) | 1,126.10 (944.30 to 1,325.20) | 0.01 (-0.04 to 0.07) | NA | NA | NA | 0.03 (0.02 to 0.05) | 214.77 (145.78 to 302.93) | 0.00 (-0.06 to 0.09) |
| Female | 0.05 (0.04 to 0.06) | 703.83 (581.97 to 838.31) | 0.00 (-0.08 to 0.08) | NA | NA | NA | 0.01 (0.01 to 0.01) | 133.22 (89.31 to 187.17) | 0.00 (-0.12 to 0.14) |
| Male | 0.12 (0.10 to 0.14) | 1,535.79 (1,283.28 to 1,816.30) | 0.01 (-0.05 to 0.09) | NA | NA | NA | 0.02 (0.02 to 0.03) | 293.89 (200.00 to 415.24) | 0.01 (-0.08 to 0.12) |
| Attention-deficit/hyperactivity disorder | | | | | | | | | |
| Total | 0.39 (0.27 to 0.54) | 2,599.95 (1,756.41 to 3,752.92) | 0.00 (0.00 to 0.01) | 0.00 (0.00 to 0.00) | 15.37 (10.22 to 22.20) | 0.02 (0.02 to 0.02) | 0.00 (0.00 to 0.01) | 31.76 (16.33 to 53.62) | 0.00 (-0.07 to 0.08) |
| Female | 0.11 (0.07 to 0.15) | 1,437.43 (964.59 to 2,071.83) | 0.00 (0.00 to 0.00) | 0.00 (0.00 to 0.00) | 8.35 (5.53 to 12.35) | 0.02 (0.02 to 0.02) | 0.00 (0.00 to 0.00) | 17.46 (9.06 to 29.94) | 0.00 (-0.13 to 0.15) |
| Male | 0.28 (0.20 to 0.40) | 3,725.72 (2,498.70 to 5,383.98) | 0.00 (0.00 to 0.00) | 0.00 (0.00 to 0.00) | 22.13 (14.68 to 32.32) | 0.02 (0.02 to 0.02) | 0.00 (0.00 to 0.01) | 45.61 (23.23 to 78.28) | 0.00 (-0.08 to 0.10) |
| Conduct disorder | | | | | | | | | |
| Total | 0.28 (0.20 to 0.36) | 1,937.01 (1,317.06 to 2,666.28) | 0.01 (0.00 to 0.01) | 0.07 (0.05 to 0.10) | 499.22 (300.11 to 709.78) | 0.01 (0.01 to 0.02) | 0.03 (0.02 to 0.05) | 236.12 (122.74 to 384.59) | 0.01 (-0.06 to 0.07) |
| Female | 0.11 (0.07 to 0.14) | 1,497.64 (972.30 to 2,173.56) | 0.01 (0.01 to 0.01) | 0.03 (0.02 to 0.04) | 393.80 (219.60 to 588.33) | 0.02 (0.01 to 0.02) | 0.01 (0.01 to 0.02) | 181.49 (91.43 to 307.57) | 0.01 (-0.09 to 0.11) |
| Male | 0.18 (0.13 to 0.22) | 2,361.60 (1,646.91 to 3,175.26) | 0.01 (0.00 to 0.01) | 0.04 (0.03 to 0.06) | 600.93 (365.27 to 855.31) | 0.01 (0.01 to 0.02) | 0.02 (0.01 to 0.03) | 288.92 (149.90 to 470.96) | 0.00 (-0.08 to 0.09) |
| Idiopathic developmental intellectual disability | | | | | | | | | |
| Total | 0.07 (0.01 to 0.13) | 463.57 (77.05 to 847.52) | 0.01 (-0.03 to 0.07) | NA | NA | NA | 0.00 (0.00 to 0.01) | 23.15 (6.08 to 44.20) | 0.01 (-0.12 to 0.16) |
| Female | 0.03 (0.01 to 0.05) | 416.73 (101.48 to 728.91) | 0.02 (-0.04 to 0.08) | NA | NA | NA | 0.00 (0.00 to 0.00) | 20.61 (5.93 to 39.72) | 0.02 (-0.15 to 0.26) |
| Male | 0.04 (0.01 to 0.07) | 509.00 (66.99 to 963.59) | 0.01 (-0.07 to 0.09) | NA | NA | NA | 0.00 (0.00 to 0.00) | 25.62 (6.47 to 50.85) | 0.00 (-0.20 to 0.23) |
| Other mental disorders | | | | | | | | | |
| Total | 0.11 (0.07 to 0.15) | 656.50 (434.75 to 900.90) | -0.01 (-0.01 to -0.01) | NA | NA | NA | 0.01 (0.00 to 0.01) | 50.46 (29.41 to 80.62) | -0.01 (-0.14 to 0.15) |
| Female | 0.04 (0.02 to 0.06) | 487.32 (310.77 to 688.16) | -0.01 (-0.01 to -0.01) | NA | NA | NA | 0.00 (0.00 to 0.00) | 37.13 (19.56 to 62.42) | -0.02 (-0.23 to 0.24) |
| Male | 0.07 (0.05 to 0.09) | 821.59 (549.92 to 1,130.72) | -0.01 (-0.01 to -0.01) | NA | NA | NA | 0.01 (0.00 to 0.01) | 63.46 (35.40 to 99.93) | -0.01 (-0.16 to 0.19) |

**Table S19.** Prevalence, Incidence, and Years Lived with Disability of Mental Disorders Among Adolescents and Young Adults (Aged 10-24) in **Southern Sub−Saharan Africa** in 2021, and Percentage Change from 2019 to 2021 According to GBD.

|  | Prevalence (95% uncertainty interval) | | | Incidence (95% uncertainty interval) | | | Years lived with disability (95% uncertainty interval) | | |
| --- | --- | --- | --- | --- | --- | --- | --- | --- | --- |
| Cause | Counts in million | Age-standardized rates per 100,000 people | Percentage rate change (2019-2021) | Counts in million | Age-standardized rates per 100,000 people | Percentage rate change (2019-2021) | Counts in million | Age-standardized rates per 100,000 people | Percentage rate change (2019-2021) |
| Mental disorders | | | | | | | | | |
| Total | 3.03 (2.68 to 3.43) | 13,920.16 (11,936.82 to 16,052.51) | 0.19 (0.14 to 0.23) | 1.44 (1.14 to 1.77) | 6,598.18 (4,992.52 to 8,521.41) | 0.34 (0.27 to 0.42) | 0.44 (0.32 to 0.59) | 2,041.10 (1,449.90 to 2,748.90) | 0.22 (0.17 to 0.27) |
| Female | 1.57 (1.36 to 1.80) | 14,453.20 (12,244.14 to 16,914.98) | 0.22 (0.17 to 0.28) | 0.80 (0.63 to 0.99) | 7,352.98 (5,526.65 to 9,580.47) | 0.37 (0.29 to 0.45) | 0.24 (0.17 to 0.31) | 2,178.39 (1,526.33 to 2,951.92) | 0.25 (0.19 to 0.31) |
| Male | 1.47 (1.31 to 1.63) | 13,390.59 (11,608.63 to 15,388.67) | 0.15 (0.11 to 0.19) | 0.64 (0.52 to 0.79) | 5,850.64 (4,437.47 to 7,511.85) | 0.31 (0.24 to 0.39) | 0.21 (0.15 to 0.27) | 1,905.00 (1,363.49 to 2,552.84) | 0.18 (0.14 to 0.23) |
| Schizophrenia | | | | | | | | | |
| Total | 0.02 (0.01 to 0.03) | 81.74 (51.23 to 123.37) | -0.01 (-0.04 to 0.01) | 0.00 (0.00 to 0.01) | 20.43 (11.96 to 31.05) | -0.01 (-0.04 to 0.02) | 0.01 (0.01 to 0.02) | 54.33 (32.36 to 86.89) | -0.01 (-0.12 to 0.1) |
| Female | 0.01 (0.01 to 0.01) | 82.78 (51.35 to 124.20) | -0.03 (-0.07 to 0.01) | 0.00 (0.00 to 0.00) | 20.96 (12.32 to 31.82) | -0.02 (-0.06 to 0.02) | 0.01 (0.00 to 0.01) | 54.38 (31.42 to 87.75) | -0.03 (-0.16 to 0.13) |
| Male | 0.01 (0.01 to 0.01) | 80.70 (50.58 to 122.36) | 0.00 (-0.04 to 0.04) | 0.00 (0.00 to 0.00) | 19.89 (11.54 to 30.32) | 0.00 (-0.03 to 0.04) | 0.01 (0.00 to 0.01) | 54.28 (32.12 to 87.89) | 0.00 (-0.14 to 0.15) |
| Depressive disorders | | | | | | | | | |
| Total | 0.78 (0.59 to 1.00) | 3,581.49 (2,620.07 to 4,773.88) | 0.32 (0.24 to 0.40) | 1.01 (0.74 to 1.34) | 4,640.90 (3,206.59 to 6,468.04) | 0.43 (0.34 to 0.54) | 0.14 (0.09 to 0.21) | 666.32 (413.25 to 997.13) | 0.37 (0.28 to 0.48) |
| Female | 0.46 (0.35 to 0.58) | 4,219.18 (3,087.70 to 5,648.96) | 0.32 (0.23 to 0.40) | 0.59 (0.43 to 0.78) | 5,448.33 (3,754.23 to 7,540.12) | 0.43 (0.33 to 0.54) | 0.08 (0.05 to 0.12) | 778.96 (484.49 to 1,169.84) | 0.37 (0.27 to 0.48) |
| Male | 0.32 (0.25 to 0.42) | 2,949.69 (2,151.09 to 3,924.58) | 0.32 (0.24 to 0.41) | 0.42 (0.30 to 0.56) | 3,841.53 (2,660.63 to 5,437.01) | 0.43 (0.33 to 0.54) | 0.06 (0.04 to 0.09) | 554.76 (340.38 to 829.86) | 0.38 (0.28 to 0.49) |
| Bipolar disorder | | | | | | | | | |
| Total | 0.09 (0.06 to 0.12) | 398.07 (279.57 to 556.97) | -0.01 (-0.01 to -0.01) | 0.01 (0.01 to 0.02) | 65.91 (41.70 to 97.48) | 0.00 (0.00 to 0.01) | 0.02 (0.01 to 0.03) | 88.25 (51.81 to 139.07) | -0.01 (-0.07 to 0.05) |
| Female | 0.04 (0.03 to 0.06) | 404.95 (284.35 to 566.69) | -0.01 (-0.01 to -0.01) | 0.01 (0.01 to 0.01) | 66.82 (42.24 to 99.02) | 0.00 (0.00 to 0.01) | 0.01 (0.01 to 0.02) | 89.06 (51.99 to 141.00) | -0.01 (-0.09 to 0.09) |
| Male | 0.04 (0.03 to 0.06) | 391.24 (273.87 to 546.41) | -0.01 (-0.01 to -0.01) | 0.01 (0.01 to 0.01) | 65.02 (40.99 to 96.04) | 0.00 (0.00 to 0.01) | 0.01 (0.01 to 0.01) | 87.45 (50.78 to 138.90) | -0.01 (-0.09 to 0.08) |
| Anxiety disorders | | | | | | | | | |
| Total | 1.20 (0.93 to 1.50) | 5,519.32 (4,092.73 to 7,277.42) | 0.38 (0.27 to 0.50) | 0.22 (0.15 to 0.29) | 989.09 (641.52 to 1,381.90) | 0.39 (0.28 to 0.51) | 0.15 (0.09 to 0.21) | 675.19 (420.51 to 992.19) | 0.38 (0.27 to 0.50) |
| Female | 0.72 (0.56 to 0.90) | 6,617.17 (4,915.55 to 8,683.21) | 0.39 (0.28 to 0.52) | 0.12 (0.09 to 0.16) | 1,116.82 (727.16 to 1,558.04) | 0.40 (0.29 to 0.53) | 0.09 (0.06 to 0.13) | 805.12 (503.49 to 1,190.81) | 0.39 (0.28 to 0.52) |
| Male | 0.49 (0.37 to 0.62) | 4,433.63 (3,227.85 to 5,920.17) | 0.37 (0.25 to 0.49) | 0.09 (0.07 to 0.13) | 862.96 (556.52 to 1,206.39) | 0.37 (0.26 to 0.50) | 0.06 (0.04 to 0.09) | 546.71 (335.97 to 808.07) | 0.36 (0.24 to 0.50) |
| Eating disorders | | | | | | | | | |
| Total | 0.07 (0.05 to 0.11) | 325.05 (200.18 to 517.20) | -0.02 (-0.05 to 0.01) | 0.08 (0.05 to 0.12) | 373.47 (199.60 to 652.64) | -0.01 (-0.04 to 0.02) | 0.02 (0.01 to 0.02) | 69.33 (36.51 to 118.52) | -0.02 (-0.07 to 0.04) |
| Female | 0.04 (0.03 to 0.06) | 398.33 (251.56 to 600.83) | -0.01 (-0.05 to 0.02) | 0.03 (0.02 to 0.05) | 297.94 (173.61 to 485.88) | 0.00 (-0.05 to 0.04) | 0.01 (0.01 to 0.01) | 84.41 (45.04 to 140.76) | -0.02 (-0.09 to 0.06) |
| Male | 0.03 (0.02 to 0.04) | 252.34 (146.96 to 430.21) | -0.03 (-0.06 to 0.01) | 0.05 (0.03 to 0.08) | 448.29 (224.88 to 814.19) | -0.01 (-0.05 to 0.03) | 0.01 (0.00 to 0.01) | 54.38 (26.67 to 99.19) | -0.03 (-0.11 to 0.05) |
| Autism spectrum disorders | | | | | | | | | |
| Total | 0.21 (0.18 to 0.25) | 975.11 (821.73 to 1,154.32) | 0.00 (-0.02 to 0.03) | NA | NA | NA | 0.04 (0.03 to 0.06) | 185.28 (126.99 to 260.61) | 0.00 (-0.04 to 0.03) |
| Female | 0.07 (0.06 to 0.09) | 688.82 (575.98 to 820.87) | 0.00 (-0.03 to 0.04) | NA | NA | NA | 0.01 (0.01 to 0.02) | 129.90 (88.90 to 182.41) | 0.00 (-0.06 to 0.06) |
| Male | 0.14 (0.12 to 0.16) | 1,258.22 (1,059.17 to 1,477.80) | 0.00 (-0.03 to 0.03) | NA | NA | NA | 0.03 (0.02 to 0.04) | 240.04 (163.11 to 337.40) | 0.00 (-0.05 to 0.04) |
| Attention-deficit/hyperactivity disorder | | | | | | | | | |
| Total | 0.23 (0.16 to 0.33) | 1,043.18 (693.13 to 1,502.90) | 0.00 (0.00 to 0.01) | 0.00 (0.00 to 0.00) | 5.68 (3.70 to 8.45) | 0.01 (0.01 to 0.01) | 0.00 (0.00 to 0.00) | 12.72 (6.40 to 22.01) | 0.00 (-0.06 to 0.06) |
| Female | 0.07 (0.04 to 0.10) | 604.23 (395.90 to 886.08) | 0.00 (0.00 to 0.01) | 0.00 (0.00 to 0.00) | 3.30 (2.16 to 5.04) | 0.01 (0.01 to 0.01) | 0.00 (0.00 to 0.00) | 7.35 (3.63 to 13.29) | 0.00 (-0.09 to 0.11) |
| Male | 0.16 (0.11 to 0.23) | 1,476.82 (986.82 to 2,126.74) | 0.00 (0.00 to 0.00) | 0.00 (0.00 to 0.00) | 8.02 (5.26 to 11.81) | 0.01 (0.01 to 0.01) | 0.00 (0.00 to 0.00) | 18.03 (9.08 to 30.80) | 0.00 (-0.07 to 0.06) |
| Conduct disorder | | | | | | | | | |
| Total | 0.43 (0.30 to 0.56) | 1,965.33 (1,338.17 to 2,709.37) | 0.01 (0.01 to 0.01) | 0.11 (0.07 to 0.15) | 502.70 (307.40 to 717.67) | 0.01 (0.01 to 0.01) | 0.05 (0.03 to 0.08) | 238.91 (124.67 to 390.54) | 0.01 (-0.02 to 0.04) |
| Female | 0.17 (0.11 to 0.23) | 1,545.15 (1,016.16 to 2,212.13) | 0.01 (0.01 to 0.01) | 0.04 (0.03 to 0.06) | 398.82 (228.77 to 598.71) | 0.01 (0.01 to 0.01) | 0.02 (0.01 to 0.03) | 186.87 (94.04 to 313.44) | 0.01 (-0.04 to 0.06) |
| Male | 0.26 (0.19 to 0.33) | 2,379.74 (1,651.40 to 3,194.73) | 0.01 (0.01 to 0.01) | 0.07 (0.05 to 0.09) | 604.93 (366.86 to 848.16) | 0.01 (0.01 to 0.01) | 0.03 (0.02 to 0.05) | 290.23 (154.68 to 469.31) | 0.01 (-0.03 to 0.05) |
| Idiopathic developmental intellectual disability | | | | | | | | | |
| Total | 0.08 (0.02 to 0.15) | 378.30 (79.42 to 693.32) | 0.02 (-0.14 to 0.21) | NA | NA | NA | 0.00 (0.00 to 0.01) | 16.72 (3.26 to 35.52) | 0.02 (-0.06 to 0.12) |
| Female | 0.04 (0.01 to 0.07) | 362.23 (91.38 to 632.38) | 0.02 (-0.14 to 0.20) | NA | NA | NA | 0.00 (0.00 to 0.00) | 15.86 (3.66 to 32.35) | 0.02 (-0.09 to 0.17) |
| Male | 0.04 (0.01 to 0.08) | 394.17 (69.68 to 752.95) | 0.01 (-0.13 to 0.24) | NA | NA | NA | 0.00 (0.00 to 0.00) | 17.57 (2.85 to 37.90) | 0.02 (-0.09 to 0.18) |
| Other mental disorders | | | | | | | | | |
| Total | 0.10 (0.06 to 0.14) | 444.23 (283.18 to 634.76) | -0.01 (-0.01 to -0.01) | NA | NA | NA | 0.01 (0.00 to 0.01) | 34.04 (18.88 to 53.94) | -0.01 (-0.09 to 0.08) |
| Female | 0.04 (0.02 to 0.05) | 348.98 (219.45 to 500.37) | -0.01 (-0.02 to -0.01) | NA | NA | NA | 0.00 (0.00 to 0.00) | 26.48 (14.27 to 42.36) | -0.02 (-0.14 to 0.12) |
| Male | 0.06 (0.04 to 0.08) | 538.81 (343.79 to 773.13) | -0.01 (-0.01 to -0.01) | NA | NA | NA | 0.00 (0.00 to 0.01) | 41.55 (23.12 to 65.91) | -0.01 (-0.10 to 0.10) |

**Table S20**. Prevalence, Incidence, and Years Lived with Disability of Mental Disorders Among Adolescents and Young Adults (Aged 10-24) in **Tropical Latin America** in 2021, and Percentage Change from 2019 to 2021 According to GBD.

|  | Prevalence (95% uncertainty interval) | | | Incidence (95% uncertainty interval) | | | Years lived with disability (95% uncertainty interval) | | |
| --- | --- | --- | --- | --- | --- | --- | --- | --- | --- |
| Cause | Counts in million | Age-standardized rates per 100,000 people | Percentage rate change (2019-2021) | Counts in million | Age-standardized rates per 100,000 people | Percentage rate change (2019-2021) | Counts in million | Age-standardized rates per 100,000 people | Percentage rate change (2019-2021) |
| Mental disorders | | | | | | | | | |
| Total | 10.26 (9.08 to 11.59) | 20,051.39 (17,208.63 to 23,077.94) | 0.14 (0.10 to 0.18) | 3.70 (2.99 to 4.51) | 7,128.88 (5,550.56 to 8,894.60) | 0.25 (0.18 to 0.33) | 1.34 (0.96 to 1.79) | 2,594.90 (1,827.58 to 3,494.54) | 0.18 (0.13 to 0.23) |
| Female | 5.53 (4.87 to 6.26) | 21,766.89 (18,619.91 to 25,265.49) | 0.18 (0.13 to 0.23) | 2.36 (1.89 to 2.92) | 9,164.76 (7,038.47 to 11,665.96) | 0.27 (0.18 to 0.36) | 0.79 (0.56 to 1.06) | 3,086.21 (2,163.62 to 4,188.88) | 0.20 (0.15 to 0.26) |
| Male | 4.72 (4.12 to 5.37) | 18,366.32 (15,717.86 to 21,255.39) | 0.10 (0.05 to 0.15) | 1.34 (1.10 to 1.59) | 5,140.86 (4,043.19 to 6,355.61) | 0.22 (0.16 to 0.29) | 0.55 (0.40 to 0.73) | 2,114.72 (1,502.14 to 2,812.34) | 0.14 (0.10 to 0.19) |
| Schizophrenia | | | | | | | | | |
| Total | 0.05 (0.03 to 0.07) | 93.08 (60.27 to 134.91) | 0.00 (-0.02 to 0.03) | 0.01 (0.01 to 0.02) | 22.21 (13.51 to 32.86) | 0.00 (-0.03 to 0.02) | 0.03 (0.02 to 0.05) | 61.47 (38.21 to 94.26) | -0.01 (-0.08 to 0.08) |
| Female | 0.02 (0.02 to 0.03) | 81.84 (52.77 to 117.91) | -0.01 (-0.04 to 0.02) | 0.01 (0.00 to 0.01) | 19.62 (11.78 to 29.05) | -0.01 (-0.04 to 0.02) | 0.01 (0.01 to 0.02) | 53.15 (32.72 to 82.15) | -0.02 (-0.13 to 0.11) |
| Male | 0.03 (0.02 to 0.04) | 104.09 (67.96 to 150.98) | 0.01 (-0.03 to 0.04) | 0.01 (0.00 to 0.01) | 24.74 (15.10 to 36.66) | 0.00 (-0.03 to 0.04) | 0.02 (0.01 to 0.03) | 69.64 (42.71 to 107.86) | 0.00 (-0.10 to 0.11) |
| Depressive disorders | | | | | | | | | |
| Total | 1.69 (1.29 to 2.20) | 3,151.00 (2,334.65 to 4,258.73) | 0.24 (0.15 to 0.35) | 2.31 (1.73 to 3.10) | 4,346.51 (3,092.56 to 6,004.72) | 0.31 (0.19 to 0.42) | 0.32 (0.21 to 0.48) | 602.07 (381.00 to 908.27) | 0.27 (0.16 to 0.38) |
| Female | 1.14 (0.87 to 1.49) | 4,319.21 (3,158.57 to 5,911.41) | 0.25 (0.14 to 0.37) | 1.61 (1.19 to 2.18) | 6,147.67 (4,334.87 to 8,561.02) | 0.31 (0.18 to 0.43) | 0.22 (0.14 to 0.33) | 835.59 (526.30 to 1,265.66) | 0.27 (0.15 to 0.40) |
| Male | 0.54 (0.42 to 0.71) | 2,009.31 (1,500.23 to 2,649.72) | 0.23 (0.15 to 0.33) | 0.70 (0.52 to 0.92) | 2,587.73 (1,828.04 to 3,577.20) | 0.31 (0.21 to 0.43) | 0.10 (0.06 to 0.15) | 373.87 (235.53 to 563.86) | 0.27 (0.17 to 0.39) |
| Bipolar disorder | | | | | | | | | |
| Total | 0.62 (0.48 to 0.78) | 1,164.61 (883.28 to 1,483.02) | -0.01 (-0.01 to -0.01) | 0.09 (0.07 to 0.11) | 173.68 (121.85 to 237.30) | 0.00 (0.00 to 0.00) | 0.14 (0.09 to 0.21) | 257.23 (158.90 to 392.54) | -0.01 (-0.04 to 0.02) |
| Female | 0.33 (0.26 to 0.42) | 1,263.32 (956.12 to 1,603.89) | -0.01 (-0.01 to -0.01) | 0.05 (0.04 to 0.06) | 187.53 (131.74 to 256.00) | 0.00 (0.00 to 0.00) | 0.07 (0.05 to 0.11) | 276.08 (169.00 to 423.61) | -0.01 (-0.05 to 0.03) |
| Male | 0.29 (0.22 to 0.36) | 1,068.21 (809.82 to 1,365.56) | -0.01 (-0.01 to -0.01) | 0.04 (0.03 to 0.05) | 160.28 (112.27 to 219.75) | 0.00 (0.00 to 0.00) | 0.06 (0.04 to 0.10) | 238.83 (146.54 to 366.39) | -0.01 (-0.05 to 0.03) |
| Anxiety disorders | | | | | | | | | |
| Total | 4.70 (3.74 to 5.88) | 9,131.22 (6,912.37 to 11,794.82) | 0.32 (0.23 to 0.40) | 0.80 (0.55 to 1.08) | 1,584.92 (1,041.39 to 2,176.90) | 0.33 (0.24 to 0.41) | 0.57 (0.37 to 0.82) | 1,111.80 (697.18 to 1,627.34) | 0.31 (0.23 to 0.40) |
| Female | 3.07 (2.42 to 3.84) | 12,039.60 (9,126.77 to 15,552.11) | 0.32 (0.24 to 0.40) | 0.49 (0.34 to 0.69) | 1,979.84 (1,310.96 to 2,748.09) | 0.33 (0.24 to 0.42) | 0.37 (0.24 to 0.53) | 1,456.50 (919.56 to 2,113.28) | 0.31 (0.23 to 0.40) |
| Male | 1.64 (1.28 to 2.06) | 6,297.39 (4,666.98 to 8,241.27) | 0.32 (0.23 to 0.41) | 0.31 (0.21 to 0.40) | 1,201.07 (781.60 to 1,646.22) | 0.32 (0.23 to 0.42) | 0.20 (0.13 to 0.30) | 775.98 (483.72 to 1,152.75) | 0.32 (0.23 to 0.41) |
| Eating disorders | | | | | | | | | |
| Total | 0.25 (0.17 to 0.36) | 468.26 (292.52 to 712.62) | -0.02 (-0.04 to 0.01) | 0.24 (0.15 to 0.36) | 471.66 (258.21 to 805.36) | -0.01 (-0.03 to 0.01) | 0.05 (0.03 to 0.09) | 99.46 (52.86 to 166.10) | -0.02 (-0.06 to 0.03) |
| Female | 0.16 (0.11 to 0.24) | 620.98 (396.72 to 922.10) | -0.02 (-0.04 to 0.02) | 0.11 (0.07 to 0.15) | 423.12 (247.96 to 681.53) | -0.01 (-0.04 to 0.03) | 0.03 (0.02 to 0.06) | 131.06 (71.22 to 213.73) | -0.02 (-0.07 to 0.04) |
| Male | 0.09 (0.06 to 0.13) | 318.82 (190.91 to 526.16) | -0.01 (-0.04 to 0.02) | 0.14 (0.08 to 0.21) | 518.87 (264.46 to 928.36) | -0.01 (-0.05 to 0.03) | 0.02 (0.01 to 0.03) | 68.55 (35.04 to 121.33) | -0.01 (-0.08 to 0.05) |
| Autism spectrum disorders | | | | | | | | | |
| Total | 0.33 (0.28 to 0.40) | 660.18 (553.03 to 784.24) | 0.00 (-0.02 to 0.02) | NA | NA | NA | 0.06 (0.04 to 0.09) | 125.28 (84.81 to 175.93) | 0.00 (-0.03 to 0.03) |
| Female | 0.11 (0.09 to 0.13) | 442.19 (365.71 to 531.42) | 0.00 (-0.03 to 0.03) | NA | NA | NA | 0.02 (0.01 to 0.03) | 82.98 (56.69 to 117.82) | 0.00 (-0.06 to 0.05) |
| Male | 0.22 (0.19 to 0.26) | 871.92 (732.76 to 1,033.44) | 0.00 (-0.02 to 0.02) | NA | NA | NA | 0.04 (0.03 to 0.06) | 166.36 (112.67 to 233.63) | 0.00 (-0.04 to 0.04) |
| Attention-deficit/hyperactivity disorder | | | | | | | | | |
| Total | 2.14 (1.49 to 3.03) | 4,298.35 (2,926.52 to 6,159.29) | 0.00 (-0.10 to 0.09) | 0.01 (0.01 to 0.01) | 21.82 (14.80 to 32.21) | 0.02 (-0.08 to 0.12) | 0.03 (0.01 to 0.04) | 52.30 (27.40 to 89.27) | -0.01 (-0.10 to 0.09) |
| Female | 0.67 (0.46 to 0.97) | 2,732.89 (1,863.25 to 4,013.08) | -0.01 (-0.13 to 0.13) | 0.00 (0.00 to 0.00) | 14.23 (9.70 to 21.28) | 0.01 (-0.13 to 0.16) | 0.01 (0.00 to 0.01) | 33.03 (17.40 to 55.42) | -0.01 (-0.14 to 0.13) |
| Male | 1.47 (1.01 to 2.08) | 5,816.61 (3,913.05 to 8,362.82) | 0.00 (-0.12 to 0.14) | 0.01 (0.00 to 0.01) | 29.11 (19.74 to 42.99) | 0.02 (-0.11 to 0.16) | 0.02 (0.01 to 0.03) | 70.99 (36.60 to 119.08) | 0.00 (-0.13 to 0.14) |
| Conduct disorder | | | | | | | | | |
| Total | 0.91 (0.65 to 1.20) | 1,944.13 (1,335.67 to 2,667.61) | 0.01 (0.00 to 0.01) | 0.23 (0.15 to 0.31) | 508.09 (307.75 to 718.76) | 0.02 (0.01 to 0.02) | 0.11 (0.06 to 0.18) | 235.59 (121.76 to 382.86) | 0.01 (-0.02 to 0.03) |
| Female | 0.34 (0.23 to 0.45) | 1,470.82 (939.84 to 2,105.70) | 0.01 (0.00 to 0.01) | 0.09 (0.06 to 0.13) | 392.76 (224.24 to 599.83) | 0.02 (0.01 to 0.02) | 0.04 (0.02 to 0.07) | 176.63 (89.58 to 297.08) | 0.01 (-0.03 to 0.05) |
| Male | 0.58 (0.42 to 0.74) | 2,401.01 (1,662.14 to 3,252.42) | 0.01 (0.00 to 0.01) | 0.15 (0.10 to 0.19) | 619.06 (379.18 to 867.44) | 0.01 (0.01 to 0.02) | 0.07 (0.04 to 0.11) | 292.51 (154.57 to 476.54) | 0.00 (-0.03 to 0.04) |
| Idiopathic developmental intellectual disability | | | | | | | | | |
| Total | 0.20 (0.05 to 0.35) | 402.33 (97.85 to 689.63) | 0.01 (-0.03 to 0.04) | NA | NA | NA | 0.01 (0.00 to 0.02) | 18.42 (5.04 to 35.92) | 0.01 (-0.05 to 0.07) |
| Female | 0.09 (0.03 to 0.15) | 368.64 (109.33 to 618.49) | 0.00 (-0.03 to 0.03) | NA | NA | NA | 0.00 (0.00 to 0.01) | 16.92 (5.49 to 32.22) | 0.00 (-0.06 to 0.07) |
| Male | 0.11 (0.02 to 0.19) | 435.00 (89.01 to 761.08) | 0.01 (-0.03 to 0.06) | NA | NA | NA | 0.01 (0.00 to 0.01) | 19.87 (4.84 to 39.55) | 0.01 (-0.05 to 0.07) |
| Other mental disorders | | | | | | | | | |
| Total | 0.22 (0.14 to 0.32) | 409.02 (255.78 to 584.54) | -0.01 (-0.01 to -0.01) | NA | NA | NA | 0.02 (0.01 to 0.03) | 31.27 (17.33 to 50.19) | -0.01 (-0.07 to 0.06) |
| Female | 0.09 (0.05 to 0.13) | 321.59 (194.53 to 463.13) | -0.01 (-0.01 to -0.01) | NA | NA | NA | 0.01 (0.00 to 0.01) | 24.26 (12.75 to 39.90) | -0.01 (-0.12 to 0.12) |
| Male | 0.14 (0.09 to 0.20) | 494.70 (312.90 to 708.67) | -0.01 (-0.01 to -0.01) | NA | NA | NA | 0.01 (0.01 to 0.02) | 38.14 (21.56 to 61.34) | -0.01 (-0.09 to 0.08) |

**Table S21.** Prevalence, Incidence, and Years Lived with Disability of Mental Disorders Among Adolescents and Young Adults (Aged 10-24) in **Western Europe** in 2021, and Percentage Change from 2019 to 2021 According to GBD.

|  | Prevalence (95% uncertainty interval) | | | Incidence (95% uncertainty interval) | | | Years lived with disability (95% uncertainty interval) | | |
| --- | --- | --- | --- | --- | --- | --- | --- | --- | --- |
| Cause | Counts in million | Age-standardized rates per 100,000 people | Percentage rate change (2019-2021) | Counts in million | Age-standardized rates per 100,000 people | Percentage rate change (2019-2021) | Counts in million | Age-standardized rates per 100,000 people | Percentage rate change (2019-2021) |
| Mental disorders | | | | | | | | | |
| Total | 15.11 (13.26 to 17.20) | 20,916.93 (17,839.55 to 24,301.23) | 0.14 (0.11 to 0.18) | 6.45 (5.14 to 8.02) | 8,903.60 (6,672.74 to 11,503.49) | 0.28 (0.22 to 0.35) | 2.18 (1.57 to 2.92) | 3,003.03 (2,127.61 to 4,064.26) | 0.18 (0.14 to 0.22) |
| Female | 8.24 (7.11 to 9.52) | 23,430.55 (19,626.12 to 27,713.54) | 0.18 (0.14 to 0.22) | 4.07 (3.17 to 5.13) | 11,545.91 (8,495.60 to 15,203.55) | 0.29 (0.22 to 0.36) | 1.28 (0.90 to 1.73) | 3,615.75 (2,508.70 to 4,962.93) | 0.20 (0.15 to 0.25) |
| Male | 6.87 (6.06 to 7.74) | 18,552.89 (16,055.10 to 21,363.24) | 0.11 (0.08 to 0.14) | 2.39 (1.93 to 2.88) | 6,414.91 (4,867.87 to 8,160.22) | 0.27 (0.21 to 0.34) | 0.91 (0.66 to 1.20) | 2,426.20 (1,733.49 to 3,256.08) | 0.15 (0.11 to 0.20) |
| Schizophrenia | | | | | | | | | |
| Total | 0.07 (0.04 to 0.10) | 87.46 (55.97 to 134.43) | 0.00 (-0.03 to 0.02) | 0.02 (0.01 to 0.02) | 20.96 (12.38 to 32.24) | 0.00 (-0.03 to 0.02) | 0.04 (0.03 to 0.07) | 57.96 (35.52 to 92.28) | -0.01 (-0.08 to 0.07) |
| Female | 0.03 (0.02 to 0.04) | 77.48 (49.24 to 119.92) | 0.00 (-0.04 to 0.03) | 0.01 (0.00 to 0.01) | 18.27 (10.61 to 28.41) | 0.00 (-0.03 to 0.04) | 0.02 (0.01 to 0.03) | 50.56 (29.81 to 80.68) | -0.01 (-0.11 to 0.12) |
| Male | 0.04 (0.03 to 0.05) | 96.82 (61.98 to 147.63) | 0.00 (-0.04 to 0.03) | 0.01 (0.01 to 0.01) | 23.49 (14.02 to 35.91) | 0.00 (-0.03 to 0.03) | 0.03 (0.02 to 0.04) | 64.90 (39.19 to 104.11) | -0.01 (-0.10 to 0.10) |
| Depressive disorders | | | | | | | | | |
| Total | 3.42 (2.55 to 4.44) | 4,630.43 (3,264.16 to 6,438.04) | 0.28 (0.21 to 0.36) | 4.53 (3.25 to 6.04) | 6,176.64 (4,107.59 to 8,766.86) | 0.35 (0.28 to 0.45) | 0.65 (0.42 to 0.97) | 888.14 (538.16 to 1,365.61) | 0.32 (0.24 to 0.41) |
| Female | 2.21 (1.64 to 2.88) | 6,172.10 (4,296.18 to 8,644.37) | 0.28 (0.21 to 0.37) | 2.98 (2.13 to 4) | 8,385.84 (5,560.42 to 12,015.67) | 0.35 (0.27 to 0.44) | 0.42 (0.27 to 0.64) | 1,190.26 (717.38 to 1,829.82) | 0.31 (0.23 to 0.41) |
| Male | 1.21 (0.91 to 1.56) | 3,179.38 (2,256.64 to 4,352.21) | 0.28 (0.21 to 0.37) | 1.55 (1.11 to 2.07) | 4,096.41 (2,776.31 to 5,743.12) | 0.36 (0.28 to 0.46) | 0.23 (0.15 to 0.34) | 603.76 (367.62 to 926.69) | 0.33 (0.24 to 0.42) |
| Bipolar disorder | | | | | | | | | |
| Total | 0.60 (0.44 to 0.81) | 801.80 (582.34 to 1,094.25) | 0.00 (0.00 to 0.00) | 0.08 (0.06 to 0.11) | 115.39 (79.15 to 166.32) | 0.00 (0.00 to 0.00) | 0.13 (0.08 to 0.21) | 177.41 (107.19 to 278.49) | -0.01 (-0.04 to 0.02) |
| Female | 0.27 (0.20 to 0.37) | 904.18 (656.35 to 1,232.56) | 0.00 (-0.01 to 0) | 0.05 (0.03 to 0.06) | 132.35 (89.33 to 191.87) | 0.00 (0.00 to 0.00) | 0.07 (0.04 to 0.11) | 198.35 (119.01 to 314.42) | -0.01 (-0.05 to 0.04) |
| Male | 0.33 (0.24 to 0.44) | 705.60 (514.19 to 960.33) | 0.00 (0.00 to 0.00) | 0.04 (0.03 to 0.05) | 99.40 (68.90 to 141.58) | 0.00 (0.00 to 0.00) | 0.06 (0.04 to 0.09) | 157.74 (94.69 to 245.70) | -0.01 (-0.05 to 0.04) |
| Anxiety disorders | | | | | | | | | |
| Total | 6.75 (5.04 to 8.88) | 9,358.41 (6,736.93 to 12,677.89) | 0.28 (0.19 to 0.36) | 1.05 (0.76 to 1.39) | 1,478.71 (970.09 to 2,057.67) | 0.29 (0.20 to 0.37) | 0.83 (0.52 to 1.22) | 1,145.33 (700.24 to 1,697.81) | 0.27 (0.19 to 0.36) |
| Female | 4.36 (3.27 to 5.72) | 12,451.91 (8,975.38 to 16,921.27) | 0.28 (0.19 to 0.36) | 0.64 (0.46 to 0.85) | 1,864.79 (1,232.10 to 2,616.36) | 0.29 (0.20 to 0.37) | 0.53 (0.33 to 0.77) | 1,515.84 (928.61 to 2,243.54) | 0.27 (0.19 to 0.36) |
| Male | 2.39 (1.76 to 3.15) | 6,441.90 (4,557.72 to 8,776.44) | 0.27 (0.18 to 0.36) | 0.41 (0.29 to 0.54) | 1,114.06 (730.51 to 1,551.99) | 0.29 (0.19 to 0.38) | 0.30 (0.19 to 0.44) | 795.99 (484.04 to 1,201.41) | 0.27 (0.17 to 0.37) |
| Eating disorders | | | | | | | | | |
| Total | 0.65 (0.47 to 0.91) | 879.89 (580.74 to 1,314.46) | -0.01 (-0.03 to 0.01) | 0.39 (0.26 to 0.54) | 543.52 (302.10 to 915.00) | 0.00 (-0.03 to 0.02) | 0.14 (0.08 to 0.22) | 187.52 (105.56 to 310.57) | -0.01 (-0.04 to 0.02) |
| Female | 0.49 (0.35 to 0.69) | 1,365.21 (913.00 to 2,041.07) | -0.01 (-0.04 to 0.02) | 0.24 (0.16 to 0.34) | 698.02 (394.78 to 1,158.45) | 0.00 (-0.04 to 0.03) | 0.10 (0.06 to 0.16) | 289.89 (162.41 to 481.66) | -0.01 (-0.05 to 0.02) |
| Male | 0.16 (0.11 to 0.24) | 423.45 (263.86 to 674.93) | -0.01 (-0.03 to 0.02) | 0.15 (0.10 to 0.22) | 397.82 (214.33 to 693.78) | 0.00 (-0.03 to 0.03) | 0.03 (0.02 to 0.06) | 91.24 (48.30 to 157.06) | -0.01 (-0.06 to 0.04) |
| Autism spectrum disorders | | | | | | | | | |
| Total | 0.68 (0.58 to 0.80) | 949.11 (798.15 to 1,113.48) | 0.00 (-0.02 to 0.02) | NA | NA | NA | 0.13 (0.09 to 0.18) | 180.58 (123.89 to 251.97) | -0.01 (-0.03 to 0.03) |
| Female | 0.18 (0.15 to 0.21) | 503.40 (420.20 to 602.04) | -0.01 (-0.03 to 0.02) | NA | NA | NA | 0.03 (0.02 to 0.05) | 94.92 (64.31 to 134.66) | -0.01 (-0.06 to 0.04) |
| Male | 0.51 (0.43 to 0.59) | 1,369.39 (1,150.46 to 1,603.78) | 0.00 (-0.03 to 0.03) | NA | NA | NA | 0.10 (0.07 to 0.13) | 261.36 (180.18 to 362.87) | -0.01 (-0.04 to 0.03) |
| Attention-deficit/hyperactivity disorder | | | | | | | | | |
| Total | 2.00 (1.39 to 2.81) | 2,837.61 (1,890.71 to 4,020.16) | 0.00 (-0.03 to 0.03) | 0.01 (0.01 to 0.02) | 18.27 (12.26 to 26.79) | 0.00 (-0.03 to 0.03) | 0.02 (0.01 to 0.04) | 34.62 (17.75 to 58.06) | 0.00 (-0.04 to 0.03) |
| Female | 0.54 (0.37 to 0.76) | 1,569.76 (1,052.99 to 2,235.29) | 0.00 (-0.04 to 0.04) | 0.00 (0.00 to 0.00) | 9.69 (6.35 to 14.42) | 0.01 (-0.04 to 0.05) | 0.01 (0.00 to 0.01) | 19.02 (9.83 to 32.50) | 0.00 (-0.05 to 0.06) |
| Male | 1.46 (1.02 to 2.03) | 4,035.22 (2,687.14 to 5,716.56) | 0 (-0.03 to 0.03) | 0.01 (0.01 to 0.01) | 26.40 (17.78 to 38.80) | 0.00 (-0.03 to 0.03) | 0.02 (0.01 to 0.03) | 49.35 (25.14 to 82.55) | 0.00 (-0.05 to 0.04) |
| Conduct disorder | | | | | | | | | |
| Total | 1.49 (1.08 to 1.89) | 2,171.57 (1,511.54 to 2,916.76) | 0.00 (0.00 to 0.01) | 0.37 (0.25 to 0.49) | 550.13 (336.04 to 771.93) | 0.01 (0.01 to 0.01) | 0.18 (0.10 to 0.29) | 263.93 (140.22 to 426.27) | 0.00 (-0.02 to 0.02) |
| Female | 0.55 (0.38 to 0.73) | 1,666.02 (1,089.41 to 2,353.44) | 0.01 (0.00 to 0.01) | 0.14 (0.09 to 0.2) | 436.94 (249.47 to 649.10) | 0.01 (0.01 to 0.01) | 0.07 (0.03 to 0.11) | 201.09 (102.29 to 333.23) | 0.00 (-0.03 to 0.04) |
| Male | 0.94 (0.69 to 1.18) | 2,649.52 (1,883.53 to 3,494.87) | 0.00 (0.00 to 0.00) | 0.23 (0.16 to 0.29) | 657.33 (409.28 to 913.35) | 0.01 (0.00 to 0.01) | 0.11 (0.06 to 0.18) | 323.35 (175.06 to 515.75) | 0.00 (-0.03 to 0.03) |
| Idiopathic developmental intellectual disability | | | | | | | | | |
| Total | 0.31 (0.09 to 0.55) | 434.82 (121.28 to 768.39) | 0.01 (-0.05 to 0.08) | NA | NA | NA | 0.01 (0.00 to 0.03) | 20.73 (6.51 to 39.41) | 0.01 (-0.04 to 0.09) |
| Female | 0.16 (0.06 to 0.26) | 451.12 (166.12 to 737.53) | 0.00 (-0.06 to 0.09) | NA | NA | NA | 0.01 (0.00 to 0.01) | 21.59 (8.11 to 38.85) | 0.00 (-0.07 to 0.09) |
| Male | 0.16 (0.03 to 0.30) | 419.44 (80.13 to 805.26) | 0.02 (-0.07 to 0.11) | NA | NA | NA | 0.01 (0.00 to 0.01) | 19.91 (4.87 to 40.11) | 0.02 (-0.06 to 0.12) |
| Other mental disorders | | | | | | | | | |
| Total | 0.47 (0.31 to 0.64) | 611.61 (404.74 to 838.14) | 0.00 (0.00 to 0.00) | NA | NA | NA | 0.04 (0.02 to 0.06) | 46.80 (27.40 to 73.21) | -0.01 (-0.06 to 0.05) |
| Female | 0.17 (0.11 to 0.24) | 452.46 (292.24 to 639.37) | -0.01 (-0.01 to -0.01) | NA | NA | NA | 0.01 (0.01 to 0.02) | 34.24 (19.51 to 55.36) | -0.01 (-0.09 to 0.08) |
| Male | 0.30 (0.20 to 0.41) | 761.06 (514.94 to 1,037.56) | 0.00 (0.00 to 0.00) | NA | NA | NA | 0.02 (0.01 to 0.04) | 58.60 (34.19 to 90.87) | -0.01 (-0.08 to 0.07) |

**Table S22.** Prevalence, Incidence, and Years Lived with Disability of Mental Disorders Among Adolescents and Young Adults (Aged 10-24) in **Western Sub−Saharan Africa** in 2021, and Percentage Change from 2019 to 2021 According to GBD.

|  | Prevalence (95% uncertainty interval) | | | Incidence (95% uncertainty interval) | | | Years lived with disability (95% uncertainty interval) | | |  |  |  |  |  |  |  |  |
| --- | --- | --- | --- | --- | --- | --- | --- | --- | --- | --- | --- | --- | --- | --- | --- | --- | --- |
| Cause | Counts in million | Age-standardized rates per 100,000 people | Percentage rate change (2019-2021) | Counts in million | Age-standardized rates per 100,000 people | Percentage rate change (2019-2021) | Counts in million | Age-standardized rates per 100,000 people | Percentage rate change (2019-2021) |  |  |  |  |  |  |  |  |
| Mental disorders | | | | | | | | | |  |  |  |  |  |  |  |  |
| Total | 18.74 (16.50 to 21.29) | 11,672.23 (9,985.61 to 13,549.68) | 0.04 (0.02 to 0.06) | 7.40 (5.91 to 9.28) | 4,643.23 (3,469.76 to 6,122.17) | 0.07 (0.04 to 0.10) | 2.60 (1.89 to 3.40) | 1,629.23 (1,156.47 to 2,179.33) | 0.05 (0.03 to 0.07) |  |  |  |  |  |  |  |  |
| Female | 9.64 (8.45 to 11.09) | 11,733.43 (9,888.81 to 13,752.16) | 0.05 (0.03 to 0.07) | 4.25 (3.32 to 5.41) | 5,200.34 (3,800.31 to 7,000.06) | 0.07 (0.04 to 0.11) | 1.39 (1.00 to 1.85) | 1,706.22 (1,195.22 to 2,312.45) | 0.05 (0.03 to 0.08) |  |  |  |  |  |  |  |  |
| Male | 9.10 (8.02 to 10.23) | 11,590.89 (10,013.83 to 13,317.09) | 0.03 (0.02 to 0.05) | 3.15 (2.56 to 3.85) | 4,046.01 (3,072.04 to 5,203.07) | 0.06 (0.03 to 0.09) | 1.21 (0.88 to 1.57) | 1,545.56 (1,110.83 to 2,053.84) | 0.04 (0.02 to 0.06) |  |  |  |  |  |  |  |  |
| Schizophrenia | | | | | | | | | |  |  |  |  |  |  |  |  |
| Total | 0.13 (0.08 to 0.19) | 87.05 (53.70 to 134.61) | 0.00 (-0.01 to 0.01) | 0.03 (0.02 to 0.05) | 21.67 (12.34 to 33.59) | 0.00 (-0.01 to 0.01) | 0.09 (0.05 to 0.13) | 57.87 (34.17 to 93.37) | 0.00 (-0.05 to 0.05) |  |  |  |  |  |  |  |  |
| Female | 0.08 (0.05 to 0.11) | 96.11 (58.95 to 148.74) | 0.00 (-0.02 to 0.02) | 0.02 (0.01 to 0.03) | 24.41 (13.99 to 37.55) | 0.00 (-0.02 to 0.02) | 0.05 (0.03 to 0.08) | 63.22 (36.74 to 101.86) | 0.00 (-0.06 to 0.07) |  |  |  |  |  |  |  |  |
| Male | 0.05 (0.03 to 0.08) | 77.10 (47.25 to 119.94) | 0.00 (-0.02 to 0.02) | 0.01 (0.01 to 0.02) | 18.68 (10.50 to 29.31) | 0.00 (-0.02 to 0.02) | 0.04 (0.02 to 0.06) | 52.01 (30.42 to 84.46) (0.00 to 0.00) | 0.00 (-0.08 to 0.09) |  |  |  |  |  |  |  |  |
| Depressive disorders | | | | | | | | | |  |  |  |  |  |  |  |  |
| Total | 4.07 (3.14 to 5.31) | 2,631.10 (1,875.45 to 3,588.66) | 0.06 (0.03 to 0.10) | 4.81 (3.44 to 6.60) | 3,079.35 (2,064.64 to 4,499.16) | 0.09 (0.04 to 0.13) | 0.72 (0.45 to 1.05) | 464.22 (282.55 to 712.18) | 0.07 (0.03 to 0.12) |  |  |  |  |  |  |  |  |
| Female | 2.53 (1.94 to 3.32) | 3,164.75 (2,261.62 to 4,323.52) | 0.06 (0.03 to 0.10) | 3.01 (2.16 to 4.12) | 3,729.86 (2,487.64 to 5,504.17) | 0.09 (0.04 to 0.14) | 0.45 (0.28 to 0.66) | 557.34 (336.09 to 861.44) | 0.08 (0.03 to 0.12) |  |  |  |  |  |  |  |  |
| Male | 1.54 (1.18 to 1.99) | 2,058.95 (1,476.70 to 2,787.49) | 0.06 (0.03 to 0.10) | 1.80 (1.29 to 2.45) | 2,385.18 (1,602.06 to 3,444.86) | 0.09 (0.04 to 0.14) | 0.27 (0.17 to 0.40) | 364.53 (224.50 to 557.51) | 0.07 (0.03 to 0.12) |  |  |  |  |  |  |  |  |
| Bipolar disorder | | | | | | | | | |  |  |  |  |  |  |  |  |
| Total | 0.57 (0.41 to 0.81) | 371.67 (254.94 to 535.10) | 0.00 (0.00 to 0.00) | 0.10 (0.07 to 0.14) | 61.22 (37.53 to 92.27) | 0.00 (0.00 to 0.00) | 0.13 (0.08 to 0.20) | 82.55 (48.17 to 132.61) | 0.00 (-0.03 to 0.03) |  |  |  |  |  |  |  |  |
| Female | 0.30 (0.21 to 0.43) | 379.19 (260.62 to 546.86) | 0.00 (0.00 to 0.00) | 0.05 (0.04 to 0.07) | 62.24 (38.42 to 93.70) | 0.00 (0.00 to 0.00) | 0.07 (0.04 to 0.11) | 83.51 (48.37 to 134.02) | 0.00 (-0.04 to 0.05) |  |  |  |  |  |  |  |  |
| Male | 0.27 (0.19 to 0.38) | 363.62 (248.80 to 524.00) | 0.00 (0.00 to 0.00) | 0.05 (0.03 to 0.07) | 60.17 (36.71 to 90.33) | 0.00 (0.00 to 0.00) | 0.06 (0.04 to 0.10) | 81.54 (47.98 to 131.67) | 0.00 (-0.04 to 0.05) |  |  |  |  |  |  |  |  |
| Anxiety disorders | | | | | | | | | |  |  |  |  |  |  |  |  |
| Total | 6.43 (4.78 to 8.44) | 4,009.97 (2,885.35 to 5,463.79) | 0.09 (0.04 to 0.15) | 1.10 (0.81 to 1.45) | 678.52 (440.74 to 943.97) | 0.09 (0.04 to 0.15) | 0.79 (0.49 to 1.17) | 490.49 (297.92 to 743.65) | 0.09 (0.04 to 0.15) |  |  |  |  |  |  |  |  |
| Female | 3.90 (2.95 to 5.12) | 4,727.24 (3,416.25 to 6,410.89) | 0.10 (0.05 to 0.15) | 0.62 (0.46 to 0.82) | 748.66 (486.80 to 1,047.28) | 0.10 (0.05 to 0.15) | 0.47 (0.29 to 0.71) | 574.87 (349.50 to 867.70) | 0.10 (0.04 to 0.16) |  |  |  |  |  |  |  |  |
| Male | 2.53 (1.91 to 3.31) | 3,255.82 (2,328.85 to 4,472.43) | 0.09 (0.04 to 0.15) | 0.48 (0.35 to 0.63) | 605.83 (392.37 to 844.01) | 0.09 (0.04 to 0.15) | 0.32 (0.19 to 0.47) | 401.83 (242.60 to 613.07) | 0.09 (0.04 to 0.15) |  |  |  |  |  |  |  |  |
| Eating disorders | | | | | | | | | |  |  |  |  |  |  |  |  |
| Total | 0.40 (0.27 to 0.58) | 258.14 (160.08 to 410.20) | 0.00 (-0.01 to 0.01) | 0.49 (0.31 to 0.75) | 312.46 (167.36 to 540.70) | 0.00 (-0.01 to 0.01) | 0.08 (0.05 to 0.14) | 55.05 (28.70 to 95.10) | 0.00 (-0.03 to 0.03) |  |  |  |  |  |  |  |  |
| Female | 0.25 (0.17 to 0.35) | 309.46 (195.60 to 477.18) | 0.00 (-0.02 to 0.02) | 0.20 (0.13 to 0.29) | 246.94 (142.19 to 407.06) | 0.00 (-0.01 to 0.02) | 0.05 (0.03 to 0.08) | 65.57 (34.95 to 109.05) | 0.00 (-0.04 to 0.05) |  |  |  |  |  |  |  |  |
| Male | 0.15 (0.10 to 0.23) | 202.79 (118.23 to 344.15) | 0.00 (-0.02 to 0.02) | 0.29 (0.17 to 0.46) | 382.26 (192.21 to 687.43) | 0.00 (-0.02 to 0.01) | 0.03 (0.02 to 0.05) | 43.71 (21.47 to 79.88) | 0.00 (-0.06 to 0.04) |  |  |  |  |  |  |  |  |
| Autism spectrum disorders | | | | | | | | | |  |  |  |  |  |  |  |  |
| Total | 1.54 (1.30 to 1.80) | 951.76 (801.35 to 1,119.27) | 0.00 (-0.01 to 0.01) | NA | NA | NA | 0.29 (0.20 to 0.41) | 180.78 (124.27 to 253.26) | 0.00 (-0.02 to 0.02) |  |  |  |  |  |  |  |  |
| Female | 0.56 (0.47 to 0.66) | 678.06 (568.56 to 801.69) | 0.00 (-0.02 to 0.01) | NA | NA | NA | 0.11 (0.07 to 0.15) | 127.79 (88.43 to 180.02) | 0.00 (-0.03 to 0.03) |  |  |  |  |  |  |  |  |
| Male | 0.98 (0.82 to 1.15) | 1,240.33 (1,046.67 to 1,456.74) | 0.00 (-0.02 to 0.02) | NA | NA | NA | 0.19 (0.13 to 0.26) | 236.65 (161.52 to 330.93) | 0.00 (-0.03 to 0.02) |  |  |  |  |  |  |  |  |
| Attention-deficit/hyperactivity disorder | | | | | | | | | |  |  |  |  |  |  |  |  |
| Total | 1.77 (1.18 to 2.59) | 1,077.10 (710.50 to 1,571.16) | 0.00 (0.00 to 0.00) | 0.01 (0.01 to 0.02) | 5.97 (3.90 to 8.89) | -0.01 (-0.01 to -0.01) | 0.02 (0.01 to 0.04) | 13.11 (6.65 to 22.24) | 0.00 (-0.03 to 0.03) |  |  |  |  |  |  |  |  |
| Female | 0.50 (0.34 to 0.74) | 601.68 (397.09 to 888.90) | 0.00 (0.00 to 0.00) | 0.00 (0.00 to 0.00) | 3.31 (2.17 to 5.05) | -0.01 (-0.01 to -0.01) | 0.01 (0.00 to 0.01) | 7.29 (3.70 to 12.63) | 0.00 (-0.05 to 0.05) |  |  |  |  |  |  |  |  |
| Male | 1.27 (0.84 to 1.84) | 1,574.67 (1,033.07 to 2,301.41) | 0.00 (0.00 to 0.00) | 0.01 (0.01 to 0.01) | 8.66 (5.71 to 12.78) | -0.01 (-0.01 to -0.01) | 0.02 (0.01 to 0.03) | 19.21 (9.63 to 32.27) | 0.00 (-0.03 to 0.03) |  |  |  |  |  |  |  |  |
| Conduct disorder | | | | | | | | | |  |  |  |  |  |  |  |  |
| Total | 3.23 (2.26 to 4.23) | 1,884.03 (1,285.43 to 2,586.86) | 0.00 (0.00 to 0.00) | 0.84 (0.55 to 1.16) | 484.04 (285.60 to 686.06) | -0.01 (-0.01 to 0.00) | 0.39 (0.21 to 0.63) | 228.05 (118.99 to 372.04) | 0.00 (-0.02 to 0.01) |  |  |  |  |  |  |  |  |
| Female | 1.29 (0.87 to 1.76) | 1,487.18 (945.80 to 2,130.14) | 0.00 (0.00 to 0.00) | 0.34 (0.21 to 0.49) | 384.92 (218.80 to 581.51) | -0.01 (-0.01 to 0.00) | 0.16 (0.08 to 0.25) | 178.80 (90.41 to 299.69) | 0.00 (-0.03 to 0.02) |  |  |  |  |  |  |  |  |
| Male | 1.93 (1.38 to 2.45) | 2,294.25 (1,596.95 to 3,103.15) | 0.00 (0.00 to 0.00) | 0.51 (0.34 to 0.68) | 585.23 (351.63 to 824.39) | 0.00 (-0.01 to 0.00) | 0.24 (0.13 to 0.37) | 278.95 (148.73 to 449.99) | 0.00 (-0.02 to 0.02) |  |  |  |  |  |  |  |  |
| Idiopathic developmental intellectual disability | | | | | | | | | |  |  |  |  |  |  |  |  |
| Total | 0.82 (0.20 to 1.47) | 506.32 (125.33 to 909.27) | 0.00 (-0.06 to 0.07) | NA | NA | NA | 0.04 (0.01 to 0.07) | 21.55 (4.78 to 44.47) | 0.00 (-0.03 to 0.05) |  |  |  |  |  |  |  |  |
| Female | 0.40 (0.11 to 0.69) | 479.91 (130.13 to 832.38) | 0.00 (-0.07 to 0.07) | NA | NA | NA | 0.02 (0.00 to 0.03) | 20.13 (4.89 to 40.41) | 0.00 (-0.05 to 0.05) |  |  |  |  |  |  |  |  |
| Male | 0.42 (0.09 to 0.78) | 534.0 (117.91 to 988.94) | 0.00 (-0.06 to 0.08) | NA | NA | NA | 0.02 (0.00 to 0.04) | 23.04 (4.57 to 48.01) | 0.00 (-0.04 to 0.07) |  |  |  |  |  |  |  |  |
| Other mental disorders | | | | | | | | | |  |  |  |  |  |  |  |  |
| Total | 0.69 (0.44 to 1.00) | 463.34 (295.94 to 661.09) | 0.00 (0.00 to 0.00) | NA | NA | NA | 0.05 (0.03 to 0.09) | 35.56 (20.02 to 56.98) | 0.00 (-0.04 to 0.05) |  |  |  |  |  |  |  |  |
| Female | 0.28 (0.18 to 0.40) | 364.11 (231.38 to 516.48) | 0.00 (0.00 to 0.00) | NA | NA | NA | 0.02 (0.01 to 0.03) | 27.69 (14.95 to 44.40) | 0.00 (-0.06 to 0.07) |  |  |  |  |  |  |  |  |
| Male | 0.41 (0.26 to 0.59) | 571.03 (366.31 to 822.16) | 0.00 (0.00 to 0.00) | NA | NA | NA | 0.03 (0.02 to 0.05) | 44.09 (25.49 to 69.97) | 0.00 (-0.05 to 0.05) |  |  |  |  |  |  |  |  |

**Table S23**. Prevalence, Incidence, and Years Lived with Disability of Mental Disorders Among Adolescents and Young Adults (Aged 10-24) in **204 Countries and Territories** in 2021, and Percentage Change from 2019 to 2021 By GBD*.

|  | Prevalence (95% uncertainty interval) | | | Incidence (95% uncertainty interval) | | | Years lived with disability (95% uncertainty interval) | | |
| --- | --- | --- | --- | --- | --- | --- | --- | --- | --- |
| Nations | Counts in 100,000 people** | Age-standardized rates per 100,000 people | Percentage rate change (2019-2021) | Counts in 100,000 people** | Age-standardized rates per 100,000 people | Percentage rate change (2019-2021) | Counts in 100,000 people** | Age-standardized rates per 100,000 people | Percentage rate change (2019-2021) |
| **Afghanistan** | | | | | | | | | |
| Total | 22.27 (18.58 to 26.74) | 20,976.05 (17,154.66 to 25,455.94) | 0.13 (0.02 to 0.25) | 9.68 (13.02 to 7.12) | 9,169.68 (6,245.74 to 12,916.37) | 0.27 (0.06 to 0.50) | 2.99 (4.05 to 2.06) | 2,828.29 (1,900.06 to 3,909.53) | 0.18 (0.05 to 0.32) |
| Female | 11.28 (9.18 to 13.82) | 21,939.65 (17,521.16 to 27,253.88) | 0.16 (0.03 to 0.31) | 5.42 (7.35 to 3.90) | 10,574.37 (7,084.66 to 15,044.09) | 0.28 (0.07 to 0.53) | 1.61 (2.20 to 1.08) | 3,137.28 (2,071.50 to 4,376.17) | 0.20 (0.05 to 0.38) |
| Male | 10.99 (9.26 to 13.08) | 20,052.97 (16,673.29 to 23,964.26) | 0.10 (0.00 to 0.21) | 4.26 (5.70 to 3.22) | 7,840.03 (5,516.69 to 10,965.66) | 0.25 (0.05 to 0.48) | 1.38 (1.85 to 0.95) | 2,534.81 (1,738.95 to 3,497.58) | 0.15 (0.03 to 0.29) |
| **Albania** | | | | | | | | | |
| Total | 0.74 (0.62 to 0.88) | 13,840.13 (11,453.52 to 16,888.37) | 0.18 (0.07 to 0.29) | 0.26 (0.34 to 0.19) | 4,785.56 (3,462.88 to 6,518.16) | 0.38 (0.19 to 0.62) | 0.10 (0.13 to 0.07) | 1,822.07 (1,257.13 to 2,536.12) | 0.22 (0.10 to 0.37) |
| Female | 0.37 (0.31 to 0.46) | 14,549.53 (11,709.09 to 18,259.39) | 0.24 (0.11 to 0.38) | 0.16 (0.22 to 0.12) | 6,100.67 (4,301.63 to 8,634.06) | 0.43 (0.19 to 0.70) | 0.05 (0.07 to 0.04) | 2,061.27 (1,374.76 to 2,945.33) | 0.28 (0.12 to 0.46) |
| Male | 0.37 (0.32 to 0.43) | 13,201.82 (11,119.43 to 15,740.19) | 0.12 (0.04 to 0.21) | 0.10 (0.13 to 0.08) | 3,601.36 (2,682.49 to 4,731.03) | 0.31 (0.15 to 0.52) | 0.05 (0.06 to 0.03) | 1,606.72 (1,130.05 to 2,201.70) | 0.17 (0.06 to 0.31) |
| **Algeria** | | | | | | | | | |
| Total | 18.19 (15.40 to 21.61) | 17,980.74 (14,868.40 to 21,675.92) | 0.07 (-0.03 to 0.19) | 7.58 (10.47 to 5.57) | 7,513.39 (5,135.69 to 10,839.37) | 0.13 (-0.02 to 0.33) | 2.52 (3.36 to 1.72) | 2,501.29 (1,684.17 to 3,436.26) | 0.09 (-0.03 to 0.23) |
| Female | 9.31 (7.71 to 11.32) | 18,866.37 (15,063.84 to 23,421.35) | 0.08 (-0.05 to 0.23) | 4.33 (6.04 to 3.12) | 8,779.03 (5,883.79 to 12,926.07) | 0.14 (-0.04 to 0.35) | 1.37 (1.87 to 0.92) | 2,781.42 (1,805.51 to 3,903.42) | 0.09 (-0.05 to 0.26) |
| Male | 8.88 (7.64 to 10.25) | 17,122.71 (14,359.51 to 20,151.10) | 0.06 (-0.02 to 0.17) | 3.26 (4.35 to 2.41) | 6,297.45 (4,440.17 to 8,928.44) | 0.13 (-0.03 to 0.33) | 1.15 (1.54 to 0.81) | 2,231.58 (1,530.28 to 3,042.02) | 0.08 (-0.03 to 0.22) |
| **Ameican Samoa** | | | | | | | | | |
| Total | 0.02 (0.01 to 0.02) | 12,861.81 (10,475.00 to 15,715.83) | 0.08 (-0.02 to 0.18) | 0.01 (0.01 to 0.00) | 4,251.50 (3,080.75 to 5,696.29) | 0.15 (-0.01 to 0.31) | 0.00 (0.00 to 0.00) | 1,651.84 (1,125.58 to 2,293.37) | 0.10 (-0.02 to 0.23) |
| Female | 0.01 (0.01 to 0.01) | 12,731.55 (10,084.11 to 16,157.89) | 0.11 (-0.02 to 0.25) | 0.00 (0.00 to 0.00) | 4,506.17 (3,206.13 to 6,160.28) | 0.17 (-0.02 to 0.35) | 0.00 (0.00 to 0.00) | 1,722.80 (1,149.94 to 2,473.05) | 0.12 (-0.02 to 0.28) |
| Male | 0.01 (0.01 to 0.01) | 12,971.50 (10,812.01 to 15,418.87) | 0.06 (-0.01 to 0.14) | 0.00 (0.00 to 0.00) | 4,011.26 (2,919.24 to 5,379.55) | 0.13 (-0.02 to 0.31) | 0.00 (0.00 to 0.00) | 1,583.93 (1,090.28 to 2,192.36) | 0.08 (-0.02 to 0.20) |
| **Andorra** | | | | | | | | | |
| Total | 0.03 (0.02 to 0.03) | 20,570.60 (16,777.73 to 25,045.76) | 0.15 (0.04 to 0.27) | 0.01 (0.02 to 0.01) | 8,663.60 (6,008.95 to 12,067.15) | 0.30 (0.09 to 0.53) | 0.00 (0.01 to 0.00) | 2,982.62 (2,041.58 to 4,225.28) | 0.19 (0.05 to 0.32) |
| Female | 0.01 (0.01 to 0.02) | 22,835.64 (18,118.39 to 28,517.01) | 0.18 (0.04 to 0.33) | 0.01 (0.01 to 0.01) | 11,168.54 (7,544.67 to 15,820.50) | 0.31 (0.10 to 0.56) | 0.00 (0.00 to 0.00) | 3,549.30 (2,356.93 to 5,067.54) | 0.21 (0.05 to 0.35) |
| Male | 0.01 (0.01 to 0.01) | 18,474.49 (15,460.65 to 21,984.67) | 0.11 (0.02 to 0.21) | 0.00 (0.01 to 0.00) | 6,337.47 (4,516.11 to 8,640.92) | 0.27 (0.07 to 0.49) | 0.00 (0.00 to 0.00) | 2,456.93 (1,698.19 to 3,427.07) | 0.15 (0.02 to 0.28) |
| **Angola** | | | | | | | | | |
| Total | 14.89 (12.45 to 17.51) | 14,441.86 (11,794.19 to 17,492.59) | 0.10 (0.00 to 0.21) | 8.66 (11.85 to 6.22) | 8,587.52 (5,874.96 to 12,353.13) | 0.17 (-0.02 to 0.40) | 2.27 (3.11 to 1.55) | 2,230.92 (1,507.19 to 3,176.35) | 0.12 (-0.00 to 0.25) |
| Female | 7.79 (6.38 to 9.35) | 14,846.43 (11,743.22 to 18,464.43) | 0.12 (0.00 to 0.24) | 4.94 (6.78 to 3.49) | 9,590.41 (6,410.26 to 14,105.30) | 0.19 (-0.03 to 0.46) | 1.23 (1.73 to 0.84) | 2,366.00 (1,545.04 to 3,437.12) | 0.14 (-0.00 to 0.30) |
| Male | 7.10 (6.05 to 8.17) | 14,005.73 (11,664.50 to 16,684.56) | 0.08 (-0.01 to 0.18) | 3.72 (5.14 to 2.72) | 7,528.56 (5,249.38 to 10,805.21) | 0.15 (-0.04 to 0.39) | 1.04 (1.42 to 0.72) | 2,087.60 (1,415.78 to 2,932.45) | 0.10 (-0.02 to 0.23) |
| **Antigua and Barbuda** | | | | | | | | | |
| Total | 0.04 (0.03 to 0.04) | 18,076.35 (14,917.88 to 21,621.73) | 0.10 (0.02 to 0.19) | 0.01 (0.01 to 0.01) | 5,459.75 (3,927.09 to 7,614.47) | 0.27 (0.10 to 0.48) | 0.00 (0.01 to 0.00) | 2,064.09 (1,416.79 to 2,873.89) | 0.16 (0.04 to 0.28) |
| Female | 0.02 (0.01 to 0.02) | 17,293.27 (13,879.60 to 21,515.24) | 0.15 (0.04 to 0.27) | 0.01 (0.01 to 0) | 6,846.65 (4,725.51 to 9,816.83) | 0.30 (0.09 to 0.54) | 0.00 (0.00 to 0.00) | 2,064.09 (1,416.79 to 2,873.89) | 0.19 (0.04 to 0.34) |
| Male | 0.02 (0.02 to 0.02) | 18,831.53 (15,647.34 to 22,752.18) | 0.06 (0.01 to 0.13) | 0.00 (0.01 to 0.00) | 4,124.28 (3,117.32 to 5,531.27) | 0.22 (0.07 to 0.40) | 0.00 (0.00 to 0.00) | 2,064.09 (1,416.79 to 2,873.89) | 0.12 (0.01 to 0.24) |
| **Argentina** | | | | | | | | | |
| Total | 19.88 (16.87 to 23.57) | 18,514.44 (15,329.46 to 22,424.26) | 0.17 (0.05 to 0.34) | 8.20 (10.33 to 6.51) | 7,609.50 (5,707.43 to 9,913.59) | 0.31 (0.11 to 0.52) | 2.84 (3.87 to 1.99) | 2,626.26 (1,814.01 to 3,621.98) | 0.21 (0.06to 0.41) |
| Female | 10.48 (8.67 to 12.70) | 19,627.70 (15,914.84 to 24,455.64) | 0.23 (0.07 to 0.44) | 5.12 (6.47 to 3.98) | 9,587.01 (7,021.34 to 12,745.99) | 0.34 (0.11 to 0.58) | 1.60 (2.23 to 1.10) | 2,989.62 (2,021.96 to 4,176.33) | 0.25 (0.08 to 0.47) |
| Male | 9.41 (8.14 to 11.05) | 17,413.04 (14,700.61 to 20,635.23) | 0.12 (0.02 to 0.25) | 3.08 (3.87 to 2.47) | 5,671.56 (4,277.48 to 7,346.94) | 0.26 (0.07 to 0.48) | 1.23 (1.67 to 0.90) | 2,269.14 (1,590.49 to 3,122.80) | 0.16 (0.02 to 0.32 |
| **Armenia** | | | | | | | | | |
| Total | 0.70 (0.60 to 0.82) | 13,097.32 (10,876.32 to 15,732.71) | 0.17 (0.08 to 0.28) | 0.29 (0.37 to 0.21) | 5,327.84 (3,725.53 to 7,336.27) | 0.39 (0.18 to 0.62) | 0.09 (0.13 to 0.07) | 1,765.90 (1,216.98 to 2,437.38) | 0.22 (0.10 to 0.36) |
| Female | 0.33 (0.27 to 0.39) | 12,935.31 (10,495.43 to 15,913.79) | 0.22 (0.11 to 0.37) | 0.15 (0.19 to 0.11) | 5,756.40 (3,918.30 to 8,201.57) | 0.43 (0.22 to 0.70) | 0.05 (0.06 to 0.03) | 1,846.43 (1,240.25 to 2,592.93) | 0.26 (0.12 to 0.44) |
| Male | 0.38 (0.33 to 0.43) | 13,230.93 (11,198.48 to 15,514.96) | 0.13 (0.06 to 0.22) | 0.14 (0.18 to 0.10) | 4,941.90 (3,491.43 to 6,724.46) | 0.36 (0.15 to 0.59) | 0.05 (0.06 to 0.03) | 1,692.80 (1,171.85 to 2,309.64) | 0.18 (0.06 to 0.32) |
| **Australia** | | | | | | | | | |
| Total | 11.56 (10.20 to 13.26) | 24,324.45 (20,978.30 to 28,136.20) | 0.03 (-0.04 to 0.12) | 5.10 (6.58 to 3.92) | 10,692.66 (7,821.08 to 14,259.90) | 0.09 (-0.07 to 0.30) | 1.61 (2.16 to 1.18) | 3,368.35 (2,408.96 to 4,526.93) | 0.05 (-0.05 to 0.17) |
| Female | 5.56 (4.82 to 6.44) | 23,913.08 (20,206.44 to 28,450.50) | 0.05 (-0.05 to 0.17) | 3.09 (4.04 to 2.33) | 13,278.69 (9,510.37 to 18,010.07) | 0.09 (-0.09 to 0.33) | 0.88 (1.20 to 0.63) | 3,744.19 (2,628.08 to 5,154.97) | 0.06 (-0.06 to 0.19) |
| Male | 6.00 (5.33 to 6.78) | 24,704.59 (21,426.41 to 28,248.57) | 0.02 (-0.04 to 0.09) | 2.01 (2.59 to 1.57) | 8,236.72 (6,141.64 to 10,975.80) | 0.09 (-0.08 to 0.30) | 0.74 (0.98 to 0.55) | 3,010.82 (2,192.44 to 3,999.32) | 0.04 (-0.05 to 0.15) |
| **Austria** | | | | | | | | | |
| Total | 2.90 (2.42 to 3.42) | 20,507.37 (16,824.71 to 24,745.72) | 0.12 (0.00 to 0.26) | 1.01 (1.35 to 0.78) | 7,153.67 (5,179.72 to 9,718.87) | 0.26 (0.07 to 0.46) | 0.40 (0.56 to 0.28) | 2,818.58 (1,903.50 to 3,972.77) | 0.15 (0.01 to 0.29) |
| Female | 1.56 (1.27 to 1.89) | 22,773.37 (18,187.85 to 28,396.40) | 0.14 (-0.01 to 0.31) | 0.64 (0.87 to 0.48) | 9,434.20 (6,667.47 to 13,106.06) | 0.28 (0.08 to 0.50) | 0.23 (0.33 to 0.16) | 3,391.64 (2,217.57 to 4,862.05) | 0.17 (0.01 to 0.35) |
| Male | 1.35 (1.15 to 1.57) | 18,431.74 (15,264.39 to 22,017.80) | 0.09 (0.00 to 0.21) | 0.37 (0.47 to 0.29) | 5,052.62 (3,732.95 to 6,685.24) | 0.23 (0.04 to 0.44) | 0.17 (0.23 to 0.12) | 2,291.63 (1,577.78 to 3,161.96) | 0.12 (-0.01 to 0.27) |
| **Azerbaijan** | | | | | | | | | |
| Total | 2.68 (2.31 to 3.07) | 12,142.91 (10,213.74 to 14,274.25) | 0.14 (0.06 to 0.24) | 1.03 (1.34 to 0.79) | 4,672.95 (3,354.17 to 6,431.29) | 0.33 (0.15 to 0.54) | 0.36 (0.47 to 0.25) | 1,610.06 (1,124.33 to 2,208.45) | 0.18 (0.07 to 0.31) |
| Female | 1.25 (1.06 to 1.48) | 12,019.32 (9,853.79 to 14,684.81) | 0.19 (0.09 to 0.32) | 0.56 (0.76 to 0.41) | 5,377.54 (3,743.99 to 7,573.95) | 0.36 (0.16 to 0.59) | 0.18 (0.24 to 0.12) | 1,713.48 (1,160.63 to 2,423.02) | 0.22 (0.08 to 0.39) |
| Male | 1.43 (1.24 to 1.62) | 12,235.75 (10,369.93 to 14,238.04) | 0.10 (0.04 to 0.18) | 0.47 (0.61 to 0.37) | 4,035.25 (2,923.54 to 5,417.61) | 0.29 (0.12 to 0.50) | 0.18 (0.23 to 0.13) | 1,515.37 (1,071.11 to 2,045.96) | 0.14 (0.05 to 0.25) |
| **Bahamas** | | | | | | | | | |
| Total | 0.18 (0.15 to 0.22) | 18,365.42 (15,214.58 to 21,956.86) | 0.12 (0.04 to 0.23) | 0.06 (0.08 to 0.04) | 5,742.86 (4,167.85 to 7,958.29) | 0.31 (0.14 to 0.52) | 0.02 (0.03 to 0.02) | 2,125.56 (1,469.25 to 2,879.40) | 0.19 (0.08 to 0.34) |
| Female | 0.09 (0.07 to 0.11) | 17,700.43 (14,356.85 to 21,710.10) | 0.17 (0.07 to 0.32) | 0.04 (0.05 to 0.03) | 7,186.68 (5,003.14 to 10,252.31) | 0.35 (0.15 to 0.58) | 0.01 (0.02 to 0.01) | 2,401.25 (1,626.02 to 3,304.22) | 0.23 (0.10 to 0.40) |
| Male | 0.09 (0.08 to 0.11) | 19,034.46 (15,813.67 to 22,738.70) | 0.07 (0.02 to 0.15) | 0.02 (0.03 to 0.02) | 4,278.47 (3,151.08 to 5,631.07) | 0.25 (0.10 to 0.43) | 0.01 (0.01 to 0.01) | 1,845.82 (1,291.20 to 2,503.09) | 0.14 (0.04 to 0.27) |
| **Bahrain** | | | | | | | | | |
| Total | 0.64 (0.54 to 0.77) | 19,014.90 (15,509.46 to 23,149.56) | 0.14 (0.02 to 0.26) | 0.32 (0.43 to 0.23) | 9,205.15 (6,319.78 to 12,861.94) | 0.26 (0.06 to 0.5) | 0.10 (0.13 to 0.07) | 2,804.80 (1,885.04 to 3,902.84) | 0.17 (0.04 to 0.33) |
| Female | 0.30 (0.24 to 0.36) | 20,581.81 (16,439.72 to 25,979.47) | 0.16 (0.02 to 0.31) | 0.16 (0.21 to 0.11) | 10,903.17 (7,262.68 to 15,476.89) | 0.27 (0.07 to 0.53) | 0.05 (0.06 to 0.03) | 3,207.04 (2,119.33 to 4,536.69) | 0.18 (0.02 to 0.36) |
| Male | 0.35 (0.29 to 0.41) | 17,994.84 (14,909.89 to 21,607.53) | 0.12 (0.02 to 0.22) | 0.16 (0.21 to 0.11) | 7,962.53 (5,496.48 to 11,058.85) | 0.24 (0.04 to 0.49) | 0.05 (0.07 to 0.03) | 2,522.43 (1,719.06 to 3,512.69) | 0.15 (0.01 to 0.30) |
| **Bangladesh** | | | | | | | | | |
| Total | 52.26 (44.41 to 61.82) | 11,363.64 (9,383.94 to 13,605.34) | 0.12 (0.02 to 0.22) | 26.95 (35.83 to 19.72) | 5,837.91 (4,085.63 to 8,125.96) | 0.24 (0.05 to 0.46) | 7.45 (10.10 to 5.15) | 1,614.18 (1,094.14 to 2,250.68) | 0.15 (0.03 to 0.27) |
| Female | 27.22 (22.57 to 32.87) | 11,401.94 (9,261.35 to 14,104.53) | 0.15 (0.03 to 0.29) | 16.86 (22.83 to 12.17) | 7,019.63 (4,781.35 to 10,040.88) | 0.26 (0.05 to 0.51) | 4.17 (5.71 to 2.80) | 1,737.17 (1,141.49 to 2,479.05) | 0.18 (0.03 to 0.34) |
| Male | 25.04 (21.55 to 28.81) | 11,273.33 (9,445.64 to 13,338.98) | 0.08 (0.02 to 0.16) | 10.09 (12.97 to 7.54) | 4,543.42 (3,243.40 to 6,154.57) | 0.20 (0.02 to 0.39) | 3.28 (4.47 to 2.31) | 1,476.45 (1,022.10 to 2,042.06) | 0.12 (0.01 to 0.23) |
| **Barbados** | | | | | | | | | |
| Total | 0.11 (0.09 to 0.13) | 18,575.09 (15,495.84 to 22,513.49) | 0.12 (0.05 to 0.22) | 0.03 (0.05 to 0.03) | 5,978.98 (4,309.52 to 8,217.06) | 0.34 (0.13 to 0.57) | 0.01 (0.02 to 0.01) | 2,154.49 (1,491.73 to 3,004.15) | 0.19 (0.08 to 0.33) |
| Female | 0.05 (0.04 to 0.06) | 17,859.89 (14,406.16 to 22,094.59) | 0.18 (0.08 to 0.31) | 0.02 (0.03 to 0.02) | 7,413.90 (5,156.81 to 10,599.44) | 0.38 (0.13 to 0.64) | 0.01 (0.01 to 0.00) | 2,426.44 (1,640.86 to 3,438.79) | 0.24 (0.10 to 0.40) |
| Male | 0.06 (0.05 to 0.07) | 19,244.86 (16,035.23 to 23,327.66) | 0.08 (0.02 to 0.14) | 0.01 (0.02 to 0.01) | 4,588.06 (3,404.63 to 6,056.92) | 0.28 (0.10 to 0.49) | 0.01 (0.01 to 0.00) | 1,890.02 (1,342.21 to 2,573.91) | 0.15 (0.04 to 0.26) |
| **Belarus** | | | | | | | | | |
| Total | 2.27 (1.9 to 2.68) | 16,035.36 (13,031.17 to 19,479.49) | 0.29 (0.16 to 0.44) | 1.00 (1.32 to 0.76) | 7,073.65 (5,067.86 to 9,798.26) | 0.57 (0.32 to 0.87) | 0.32 (0.43 to 0.22) | 2,252.19 (1,541.93 to 3,087.98) | 0.36 (0.21 to 0.55) |
| Female | 1.16 (0.94 to 1.41) | 16,829.04 (13,349.28 to 21,079.74) | 0.36 (0.20 to 0.55) | 0.55 (0.74 to 0.41) | 8,033.68 (5,638.33 to 11,307.70) | 0.62 (0.33 to 0.93) | 0.17 (0.23 to 0.12) | 2,477.34 (1,658.89 to 3,462.27) | 0.42 (0.24 to 0.64) |
| Male | 1.12 (0.96 to 1.30) | 15,288.73 (12,751.65 to 18,086.29) | 0.21 (0.11 to 0.34) | 0.45 (0.58 to 0.34) | 6,170.08 (4,439.56 to 8,461.96) | 0.52 (0.28 to 0.81) | 0.15 (0.20 to 0.11) | 2,040.30 (1,417.05 to 2,760.33) | 0.30 (0.16 to 0.45) |
| **Belgium** | | | | | | | | | |
| Total | 3.82 (3.24 to 4.59) | 19,440.15 (16,059.26 to 23,901.83) | 0.12 (0.02 to 0.24) | 1.57 (2.08 to 1.18) | 7,957.31 (5,635.65 to 10,992.03) | 0.24 (0.06 to 0.45) | 0.55 (0.75 to 0.38) | 2,772.67 (1,911.51 to 3,817.72) | 0.15 (0.04 to 0.28) |
| Female | 1.98 (1.66 to 2.44) | 20,557.82 (16,601.35 to 25,910.85) | 0.15 (0.03 to 0.29) | 0.96 (1.29 to 0.71) | 9,983.65 (6,889.10 to 14,232.12) | 0.26 (0.06 to 0.49) | 0.31 (0.42 to 0.21) | 3,153.16 (2,112.33 to 4,389.21) | 0.17 (0.04 to 0.32) |
| Male | 1.84 (1.57 to 2.18) | 18,362.79 (15,373.34 to 22,160.81) | 0.09 (0.01 to 0.20) | 0.60 (0.77 to 0.47) | 6,011.47 (4,373.60 to 7,984.41) | 0.22 (0.04 to 0.44) | 0.24 (0.33 to 0.17) | 2,406.96 (1,684.10 to 3,275.21) | 0.12 (0.01 to 0.26) |
| **Belize** | | | | | | | | | |
| Total | 0.24 (0.20 to 0.28) | 18,068.01 (14,978.89 to 21,498.89) | 0.09 (0.02 to 0.17) | 0.08 (0.10 to 0.06) | 5,803.35 (4,178.74 to 7,906.44) | 0.26 (0.08 to 0.46) | 0.03 (0.04 to 0.02) | 2,061.63 (1,450.48 to 2,788.48) | 0.15 (0.04 to 0.27) |
| Female | 0.11 (0.10 to 0.14) | 17,088.05 (13,886.15 to 20,833.44) | 0.14 (0.03 to 0.25) | 0.05 (0.06 to 0.03) | 6,972.86 (4,896.92 to 9,712.57) | 0.28 (0.08 to 0.51) | 0.02 (0.02 to 0.01) | 2,274.78 (1,580.37 to 3,149.79) | 0.18 (0.05 to 0.33) |
| Male | 0.13 (0.11 to 0.15) | 19,038.50 (15,938.71 to 22,765.08) | 0.06 (0.01 to 0.11) | 0.03 (0.04 to 0.02) | 4,614.84 (3,399.01 to 6,096.75) | 0.22 (0.06 to 0.45) | 0.01 (0.02 to 0.01) | 1,844.12 (1,297.88 to 2,478.31) | 0.12 (0.01 to 0.23) |
| **Benin** | | | | | | | | | |
| Total | 5.31 (4.50 to 6.32) | 12,191.67 (10,042.55 to 14,827.14) | 0.06 (-0.03 to 0.16) | 2.22 (2.96 to 1.61) | 5,137.76 (3,584.73 to 7,223.45) | 0.11 (-0.03 to 0.30) | 0.75 (1.02 to 0.52) | 1,724.79 (1,181.57 to 2,425.57) | 0.07 (-0.04 to 0.19) |
| Female | 2.74 (2.26 to 3.34) | 12,375.74 (9,924.66 to 15,455.39) | 0.07 (-0.04 to 0.19) | 1.29 (1.77 to 0.92) | 5,832.35 (3,932.79 to 8,376.98) | 0.12 (-0.04 to 0.32) | 0.40 (0.57 to 0.27) | 1,821.22 (1,210.27 to 2,622.54) | 0.08 (-0.04 to 0.21) |
| Male | 2.57 (2.23 to 3.00) | 11,980.79 (10,069.63 to 14,176.22) | 0.04 (-0.03 to 0.12) | 0.93 (1.21 to 0.69) | 4,404.39 (3,142.66 to 6,000.70) | 0.09 (-0.04 to 0.26) | 0.34 (0.46 to 0.25) | 1,621.69 (1,130.84 to 2,224.38) | 0.06 (-0.05 to 0.18) |
| **Bermuda** | | | | | | | | | |
| Total | 0.02 (0.01 to 0.02) | 18,528.80 (15,463.52 to 21,951.62) | 0.11 (0.03 to 0.21) | 0.01 (0.01 to 0.00) | 6,115.77 (4,379.95 to 8,368.54) | 0.30 (0.12 to 0.5) | 0.00 (0.00 to 0.00) | 2,198.13 (1,496.48 to 3,052.54) | 0.17 (0.05 to 0.30) |
| Female | 0.01 (0.01 to 0.01) | 17,852.13 (14,551.26 to 21,785.96) | 0.16 (0.05 to 0.29) | 0.00 (0.00 to 0.00) | 7,518.20 (5,198.52 to 10,529.29) | 0.33 (0.13 to 0.56) | 0.00 (0.00 to 0.00) | 2,478.92 (1,630.34 to 3,487.08) | 0.21 (0.07 to 0.36) |
| Male | 0.01 (0.01 to 0.01) | 19,166.85 (15,998.68 to 22,850.96) | 0.07 (0.01 to 0.14) | 0.00 (0.00 to 0.00) | 4,713.34 (3,463.84 to 6,302.25) | 0.24 (0.08 to 0.43) | 0.00 (0.00 to 0.00) | 1,915.62 (1,328.01 to 2,601.78) | 0.13 (0.02 to 0.25) |
| **Bhutan** | | | | | | | | | |
| Total | 0.22 (0.19 to 0.26) | 10,746.51 (9,015.63 to 12,698.32) | 0.01 (-0.06 to 0.09) | 0.08 (0.11 to 0.06) | 3,980.79 (2,829.83 to 5,436.70) | 0.02 (-0.12 to 0.17) | 0.03 (0.04 to 0.02) | 1,379.17 (955.64 to 1,892.75) | 0.01 (-0.08 to 0.11) |
| Female | 0.10 (0.09 to 0.12) | 10,337.30 (8,500.75 to 12,545.60) | 0.01 (-0.08 to 0.12) | 0.04 (0.06 to 0.03) | 4,337.57 (3,008.19 to 6,132.39) | 0.02 (-0.14 to 0.20) | 0.01 (0.02 to 0.01) | 1,392.31 (952.70 to 1,964.34) | 0.01 (-0.10 to 0.14) |
| Male | 0.12 (0.10 to 0.13) | 11,163.21 (9,454.37 to 12,967.11) | 0.00 (-0.06 to 0.07) | 0.04 (0.05 to 0.03) | 3,647.30 (2,647.49 to 4,942.46) | 0.01 (-0.13 to 0.18) | 0.01 (0.02 to 0.01) | 1,369.02 (951.60 to 1,876.82) | 0.00 (-0.08 to 0.10) |
| **Bolivia (Plurinational State of)** | | | | | | | | | |
| Total | 6.40 (5.43 to 7.71) | 19,708.90 (16,203.07 to 24,066.77) | 0.24 (0.13 to 0.36) | 2.22 (2.94 to 1.68) | 6,818.44 (4,837.41 to 9,305.19) | 0.51 (0.3 to 0.74) | 0.82 (1.10 to 0.57) | 2,501.34 (1,715.39 to 3,471.98) | 0.32 (0.17 to 0.50) |
| Female | 3.32 (2.74 to 4.06) | 20,599.83 (16,551.48 to 25,764.41) | 0.31 (0.18 to 0.46) | 1.36 (1.85 to 0.99) | 8,423.00 (5,887.32 to 11,799.22) | 0.56 (0.32 to 0.82) | 0.47 (0.64 to 0.32) | 2,883.10 (1,934.09 to 4,094.73) | 0.38 (0.21 to 0.58) |
| Male | 3.08 (2.65 to 3.63) | 18,828.80 (15,719.69 to 22,731.00) | 0.17 (0.08 to 0.28) | 0.86 (1.11 to 0.67) | 5,244.79 (3,844.94 to 7,030.81) | 0.43 (0.24 to 0.64) | 0.35 (0.47 to 0.25) | 2,126.80 (1,474.73 to 2,939.11) | 0.26 (0.13 to 0.42) |
| **Bosnia and Herzegovina** | | | | | | | | | |
| Total | 0.76 (0.65 to 0.89) | 13,937.77 (11,541.38 to 16,799.22) | 0.19 (0.09 to 0.31) | 0.28 (0.37 to 0.21) | 5,102.82 (3,630.45 to 6,904.35) | 0.40 (0.20 to 0.63) | 0.10 (0.14 to 0.07) | 1,863.39 (1,283.85 to 2,552.11) | 0.23 (0.10 to 0.37) |
| Female | 0.39 (0.32 to 0.47) | 14,495.14 (11,533.61 to 18,145.27) | 0.25 (0.11 to 0.39) | 0.17 (0.22 to 0.12) | 6,191.28 (4,296.67 to 8,625.57) | 0.44 (0.22 to 0.74) | 0.06 (0.08 to 0.04) | 2,067.75 (1,385.42 to 2,907.82) | 0.28 (0.12 to 0.45) |
| Male | 0.37 (0.32 to 0.43) | 13,404.35 (11,290.36 to 15,677.82) | 0.13 (0.06 to 0.22) | 0.11 (0.14 to 0.09) | 4,066.20 (2,989.01 to 5,357.00) | 0.33 (0.15 to 0.55) | 0.05 (0.06 to 0.03) | 1,668.66 (1,157.98 to 2,265.95) | 0.18 (0.06 to 0.30) |
| **Botswana** | | | | | | | | | |
| Total | 0.92 (0.77 to 1.09) | 14,053.28 (11,396.11 to 17,162.12) | 0.20 (0.09 to 0.34) | 0.44 (0.60 to 0.32) | 6,742.72 (4,721.28 to 9,489.59) | 0.35 (0.16 to 0.60) | 0.14 (0.19 to 0.10) | 2,089.41 (1,434.97 to 2,878.49) | 0.23 (0.10 to 0.39) |
| Female | 0.47 (0.39 to 0.57) | 14,475.84 (11,463.99 to 18,111.36) | 0.24 (0.10 to 0.40) | 0.24 (0.34 to 0.18) | 7,442.78 (5,129.54 to 10,683.31) | 0.38 (0.15 to 0.65) | 0.07 (0.10 to 0.05) | 2,213.77 (1,477.22 to 3,117.68) | 0.26 (0.11 to 0.45) |
| Male | 0.45 (0.38 to 0.52) | 13,629.45 (11,283.38 to 16,317.24) | 0.16 (0.07 to 0.28) | 0.20 (0.27 to 0.15) | 6,048.02 (4,305.11 to 8,430.44) | 0.33 (0.13 to 0.56) | 0.06 (0.09 to 0.05) | 1,965.64 (1,364.70 to 2,682.42) | 0.19 (0.08 to 0.33) |
| **Brazil** | | | | | | | | | |
| Total | 98.59 (87.23 to 111.37) | 20,058.18 (17,243.38 to 23,074.39) | 0.14 (0.10 to 0.19) | 35.49 (43.32 to 28.73) | 7,120.97 (5,552.18 to 8,896.93) | 0.25 (0.17 to 0.33) | 12.92 (17.15 to 9.24) | 2,594.33 (1,824.54 to 3,489.09) | 0.18 (0.13 to 0.22) |
| Female | 53.19 (46.64 to 60.25) | 21,759.92 (18,628.62 to 25,208.67) | 0.18 (0.13 to 0.23) | 22.6 (27.89 to 18.12) | 9,132.18 (7,017.91 to 11,599.43) | 0.26 (0.18 to 0.35) | 7.62 (10.22 to 5.39) | 3,082.62 (2,158.34 to 4,185.65) | 0.20 (0.15 to 0.26) |
| Male | 45.4 (39.65 to 51.63) | 18,385.24 (15,752.63 to 21,261.53) | 0.10 (0.05 to 0.15) | 12.89 (15.36 to 10.6) | 5,155.69 (4,057.03 to 6,371.98) | 0.22 (0.16 to 0.29) | 5.30 (6.98 to 3.81) | 2,116.80 (1,502.88 to 2,810.78) | 0.14 (0.10 to 0.199) |
| **Brunei Darussalam** | | | | | | | | | |
| Total | 0.15 (0.13 to 0.17) | 13,760.24 (11,570.21 to 16,351.58) | 0.07 (0.00 to 0.16) | 0.05 (0.06 to 0.04) | 4,377.46 (3,215.01 to 5,929.56) | 0.15 (0.01 to 0.32) | 0.02 (0.03 to 0.01) | 1,863.40 (1,313.15 to 2,539.44) | 0.08 (-0.01 to 0.19) |
| Female | 0.07 (0.05 to 0.08) | 13,089.13 (10,599.62 to 16,073.10) | 0.09 (-0.01 to 0.21) | 0.03 (0.03 to 0.02) | 5,127.95 (3,671.63 to 7,149.73) | 0.17 (0.01 to 0.38) | 0.01 (0.01 to 0.01) | 1,956.71 (1,326.08 to 2,719.32) | 0.10 (-0.02 to 0.24) |
| Male | 0.08 (0.07 to 0.09) | 14,367.50 (12,185.30 to 16,833.44) | 0.05 (-0.01 to 0.12) | 0.02 (0.03 to 0.02) | 3,732.43 (2,746.40 to 4,946.04) | 0.13 (0.00 to 0.29) | 0.01 (0.01 to 0.01) | 1,785.08 (1,294.43 to 2,385.43) | 0.06 (-0.03 to 0.16) |
| **Bulgaria** | | | | | | | | | |
| Total | 1.43 (1.19 to 1.70) | 14,717.60 (11,969.16 to 17,902.67) | 0.28 (0.16 to 0.43) | 0.53 (0.69 to 0.41) | 5,484.82 (3,950.75 to 7,481.22) | 0.58 (0.35 to 0.84) | 0.19 (0.26 to 0.13) | 1,977.40 (1,353.33 to 2,742.17) | 0.35 (0.21 to 0.54) |
| Female | 0.73 (0.60 to 0.89) | 15,557.58 (12,276.42 to 19,558.45) | 0.37 (0.21 to 0.56) | 0.32 (0.42 to 0.24) | 6,715.66 (4,720.89 to 9,444.19) | 0.66 (0.41 to 0.96) | 0.10 (0.14 to 0.07) | 2,227.72 (1,483.26 to 3,144.53) | 0.43 (0.25 to 0.64) |
| Male | 0.70 (0.59 to 0.81) | 13,928.23 (11,562.79 to 16,495.76) | 0.19 (0.10 to 0.32) | 0.22 (0.27 to 0.17) | 4,327.29 (3,196.54 to 5,647.01) | 0.47 (0.28 to 0.72) | 0.09 (0.12 to 0.06) | 1,742.00 (1,219.34 to 2,372.33) | 0.26 (0.13 to 0.42) |
| **Burkina Faso** | | | | | | | | | |
| Total | 8.72 (7.39 to 10.27) | 12,061.65 (9,907.73 to 14,650.39) | 0.04 (-0.05 to 0.13) | 3.49 (4.65 to 2.62) | 4,866.49 (3,454.15 to 6,825.67) | 0.06 (-0.07 to 0.24) | 1.20 (1.61 to 0.86) | 1,674.79 (1,172.08 to 2,274.92) | 0.05 (-0.05 to 0.17) |
| Female | 4.53 (3.75 to 5.50) | 12,056.73 (9,703.98 to 14,889.94) | 0.05 (-0.05 to 0.16) | 1.99 (2.73 to 1.45) | 5,332.39 (3,673.80 to 7,625.51) | 0.07 (-0.08 to 0.28) | 0.65 (0.89 to 0.46) | 1,735.25 (1,183.23 to 2,417.49) | 0.05 (-0.06 to 0.20) |
| Male | 4.18 (3.59 to 4.86) | 12,037.68 (10,094.51 to 14,290.94) | 0.03 (-0.04 to 0.1) | 1.49 (1.92 to 1.14) | 4,341.84 (3,126.03 to 6,033.88) | 0.06 (-0.07 to 0.21) | 0.55 (0.73 to 0.40) | 1,604.52 (1,128.53 to 2,155.68) | 0.04 (-0.05 to 0.15) |
| **Burundi** | | | | | | | | | |
| Total | 5.87 (4.96 to 6.89) | 13,771.49 (11,357.74 to 16,523.07) | 0.11 (0.01 to 0.22) | 2.58 (3.50 to 1.86) | 6,115.92 (4,221.50 to 8,683.26) | 0.21 (0.01 to 0.42) | 0.82 (1.10 to 0.56) | 1,931.71 (1,317.60 to 2,663.69) | 0.14 (0.02 to 0.27) |
| Female | 3.05 (2.53 to 3.67) | 13,999.83 (11,243.83 to 17,279.62) | 0.13 (0.02 to 0.25) | 1.48 (2.04 to 1.05) | 6,856.51 (4,615.89 to 9,960.12) | 0.22 (0.02 to 0.45) | 0.44 (0.60 to 0.30) | 2,034.83 (1,346.18 to 2,871.20) | 0.16 (0.02 to 0.30) |
| Male | 2.82 (2.46 to 3.26) | 13,519.25 (11,416.46 to 15,932.81) | 0.08 (0.00 to 0.19) | 1.10 (1.47 to 0.8) | 5,329.52 (3,762.76 to 7,465.63) | 0.19 (-0.01 to 0.39) | 0.38 (0.50 to 0.26) | 1,821.37 (1,267.24 to 2,469.03) | 0.11 (-0.00 to 0.25) |
| **Cote d'Ivoire** | | | | | | | | | |
| Total | 10.01 (8.55 to 11.78) | 11,683.04 (9,703.94 to 14,058.00) | 0.04 (-0.05 to 0.14) | 3.78 (4.98 to 2.88) | 4,437.22 (3,182.81 to 6,057.59) | 0.06 (-0.07 to 0.23) | 1.38 (1.87 to 0.99) | 1,624.89 (1,130.22 to 2,221.34) | 0.04 (-0.05 to 0.15) |
| Female | 4.89 (4.07 to 5.93) | 11,815.01 (9,521.96 to 14,755.89) | 0.05 (-0.05 to 0.16) | 2.09 (2.86 to 1.52) | 5,059.13 (3,541.88 to 7,110.14) | 0.06 (-0.09 to 0.25) | 0.71 (0.97 to 0.49) | 1,712.26 (1,163.60 to 2,422.13) | 0.05 (-0.07 to 0.18) |
| Male | 5.12 (4.43 to 5.88) | 11,550.88 (9,692.78 to 13,640.75) | 0.03 (-0.04 to 0.11) | 1.70 (2.13 to 1.32) | 3,849.05 (2,795.34 to 5,090.83) | 0.05 (-0.07 to 0.22) | 0.68 (0.90 to 0.49) | 1,541.70 (1,087.17 to 2,090.78) | 0.04 (-0.05 to 0.15) |
| **Cabo Verde** | | | | | | | | | |
| Total | 0.20 (0.17 to 0.24) | 13,241.99 (10,922.21 to 15,970.99) | 0.14 (0.04 to 0.25) | 0.10 (0.14 to 0.07) | 6,562.78 (4,483.18 to 9,338.20) | 0.27 (0.07 to 0.49) | 0.03 (0.04 to 0.02) | 1,979.25 (1,365.80 to 2,731.00) | 0.16 (0.06 to 0.30) |
| Female | 0.10 (0.08 to 0.12) | 13,324.71 (10,690.24 to 16,494.53) | 0.16 (0.05 to 0.29) | 0.05 (0.07 to 0.04) | 6,875.16 (4,537.30 to 10,093.71) | 0.29 (0.08 to 0.55) | 0.02 (0.02 to 0.01) | 2,043.57 (1,360.74 to 2,874.36) | 0.19 (0.06 to 0.33) |
| Male | 0.10 (0.09 to 0.12) | 13,162.46 (10,990.66 to 15,589.92) | 0.11 (0.03 to 0.21) | 0.05 (0.06 to 0.04) | 6,256.09 (4,382.61 to 8,619.80) | 0.26 (0.06 to 0.48) | 0.01 (0.02 to 0.01) | 1,916.46 (1,348.46 to 2,643.45) | 0.14 (0.03 to 0.29) |
| **Cambodia** | | | | | | | | | |
| Total | 6.30 (5.31 to 7.43) | 13,611.36 (11,178.49 to 16,479.74) | 0.13 (0.03 to 0.26) | 2.47 (3.23 to 1.83) | 5,337.20 (3,757.37 to 7,354.86) | 0.30 (0.10 to 0.52) | 0.83 (1.13 to 0.59) | 1,801.40 (1,230.58 to 2,513.98) | 0.18 (0.05 to 0.34) |
| Female | 3.19 (2.60 to 3.86) | 14,063.05 (11,196.67 to 17,658.60) | 0.16 (0.03 to 0.33) | 1.29 (1.68 to 0.94) | 5,693.10 (3,963.56 to 7,876.35) | 0.32 (0.11 to 0.56) | 0.43 (0.60 to 0.30) | 1,903.84 (1,264.28 to 2,721.15) | 0.21 (0.05 to 0.39) |
| Male | 3.12 (2.66 to 3.60) | 13,175.37 (11,018.86 to 15,532.85) | 0.10 (0.02 to 0.21) | 1.18 (1.56 to 0.87) | 4,997.83 (3,514.42 to 6,990.97) | 0.28 (0.08 to 0.50) | 0.40 (0.54 to 0.29) | 1,703.23 (1,184.13 to 2,353.89) | 0.15 (0.02 to 0.32) |
| **Cameroon** | | | | | | | | | |
| Total | 12.67 (10.61 to 14.81) | 12,276.29 (10,027.69 to 14,874.99) | 0.06 (-0.03 to 0.15) | 5.40 (7.36 to 3.92) | 5,273.50 (3,620.62 to 7,468.75) | 0.10 (-0.06 to 0.27) | 1.79 (2.44 to 1.25) | 1,747.05 (1,193.75 to 2,405.32) | 0.07 (-0.04 to 0.18) |
| Female | 6.44 (5.31 to 7.86) | 12,499.77 (9,956.22 to 15,396.42) | 0.07 (-0.03 to 0.18) | 3.08 (4.27 to 2.15) | 6,006.59 (4,013.45 to 8,770.39) | 0.11 (-0.07 to 0.31) | 0.95 (1.31 to 0.65) | 1,850.74 (1,239.09 to 2,577.64) | 0.08 (-0.04 to 0.21) |
| Male | 6.23 (5.30 to 7.26) | 12,038.59 (10,014.96 to 14,366.26) | 0.04 (-0.03 to 0.12) | 2.32 (3.09 to 1.75) | 4,530.72 (3,204.25 to 6,272.08) | 0.08 (-0.09 to 0.26) | 0.84 (1.13 to 0.60) | 1,641.07 (1,143.90 to 2,250.30) | 0.05 (-0.04 to 0.15) |
| **Canada** | | | | | | | | | |
| Total | 11.74 (10.18 to 13.5) | 17,999.59 (15,134.16 to 21,136.58) | 0.11 (0.03 to 0.21) | 5.66 (7.48 to 4.17) | 8,669.69 (6,128.67 to 11,945.29) | 0.29 (0.08 to 0.53) | 1.67 (2.20 to 1.20) | 2,546.68 (1,791.54 to 3,428.32) | 0.15 (0.03 to 0.28) |
| Female | 5.79 (4.92 to 6.80) | 18,221.94 (14,885.95 to 21,831.77) | 0.15 (0.04 to 0.27) | 3.57 (4.82 to 2.53) | 11,286.64 (7,856.19 to 15,810.60) | 0.31 (0.07 to 0.57) | 0.93 (1.25 to 0.66) | 2,912.90 (1,986.93 to 4,061.65) | 0.19 (0.05 to 0.35) |
| Male | 5.95 (5.17 to 6.80) | 17,796.73 (15,111.64 to 20,767.72) | 0.07 (0.01 to 0.15) | 2.09 (2.74 to 1.58) | 6,211.22 (4,496.41 to 8,495.63) | 0.24 (0.06 to 0.49) | 0.74 (0.96 to 0.54) | 2,203.14 (1,579.31 to 2,928.48) | 0.11 (0.01 to 0.22) |
| **Central African Republic** | | | | | | | | | |
| Total | 2.65 (2.22 to 3.13) | 14,750.91 (12,003.40 to 17,994.05) | 0.06 (-0.04 to 0.16) | 1.45 (2.06 to 1.03) | 8,181.75 (5,515.64 to 11,898.26) | 0.10 (-0.09 to 0.31) | 0.39 (0.53 to 0.27) | 2,163.98 (1,463.59 to 3,034.08) | 0.07 (-0.05 to 0.20) |
| Female | 1.36 (1.12 to 1.64) | 14,925.66 (11,882.61 to 18,502.17) | 0.06 (-0.04 to 0.19) | 0.81 (1.16 to 0.56) | 8,989.67 (5,926.31 to 13,180.27) | 0.10 (-0.09 to 0.34) | 0.21 (0.29 to 0.14) | 2,268.54 (1,508.39 to 3,222.47) | 0.08 (-0.05 to 0.23) |
| Male | 1.29 (1.1 to 1.50) | 14,544.74 (12,049.71 to 17,388.79) | 0.05 (-0.04 to 0.15) | 0.64 (0.90 to 0.46) | 7,331.90 (4,933.02 to 10,620.15) | 0.10 (-0.08 to 0.30) | 0.18 (0.24 to 0.13) | 2,052.40 (1,390.87 to 2,883.92) | 0.06 (-0.05 to 0.20) |
| **Chad** | | | | | | | | | |
| Total | 7.22 (6.17 to 8.49) | 12,502.87 (10,433.15 to 15,171.67) | 0.03 (-0.07 to 0.12) | 3.21 (4.36 to 2.33) | 5,638.97 (3,868.00 to 8,154.16) | 0.04 (-0.10 to 0.21) | 1.01 (1.37 to 0.71) | 1,778.82 (1,223.98 to 2,434.96) | 0.03 (-0.07 to 0.16) |
| Female | 3.72 (3.10 to 4.49) | 12,755.54 (10,361.50 to 15,811.14) | 0.03 (-0.07 to 0.15) | 1.86 (2.56 to 1.32) | 6,466.89 (4,294.27 to 9,487.85) | 0.05 (-0.12 to 0.24) | 0.55 (0.76 to 0.37) | 1,890.41 (1,269.11 to 2,648.17) | 0.04 (-0.08 to 0.17) |
| Male | 3.49 (3.03 to 4.04) | 12,224.63 (10,289.52 to 14,519.22) | 0.02 (-0.05 to 0.10) | 1.34 (1.79 to 1.01) | 4,771.47 (3,309.24 to 6,782.12) | 0.04 (-0.10 to 0.21) | 0.47 (0.63 to 0.33) | 1,660.86 (1,157.14 to 2,272.88) | 0.03 (-0.07 to 0.13) |
| **Chile** | | | | | | | | | |
| Total | 7.95 (6.73 to 9.54) | 20,111.64 (16,683.64 to 24,513.16) | 0.15 (0.05 to 0.29) | 4.12 (5.51 to 2.98) | 10,357.81 (7,144.15 to 14,530.06) | 0.30 (0.07 to 0.56) | 1.20 (1.64 to 0.84) | 3,008.76 (2,046.80 to 4,194.12) | 0.19 (0.06 to 0.36) |
| Female | 4.51 (3.69 to 5.57) | 23,329.10 (18,513.59 to 29,422.75) | 0.19 (0.07 to 0.35) | 2.72 (3.71 to 1.94) | 14,063.61 (9,607.97 to 20,079.17) | 0.32 (0.06 to 0.58) | 0.73 (1.02 to 0.50) | 3,756.00 (2,498.67 to 5,341.23) | 0.22 (0.08 to 0.40) |
| Male | 3.44 (2.96 to 3.97) | 17,063.42 (14,414.82 to 20,156.25) | 0.11 (0.03 to 0.21) | 1.39 (1.84 to 1.04) | 6,844.37 (4,876.74 to 9,418.27) | 0.27 (0.06 to 0.52) | 0.47 (0.63 to 0.33) | 2,300.49 (1,598.40 to 3,122.14) | 0.15 (0.02 to 0.28) |
| **China** | | | | | | | | | |
| Total | 304.34 (264.11 to 345.77) | 13,002.59 (11,069.60 to 15,049.29) | 0.03 (0.00 to 0.07) | 68.24 (79.28 to 57.42) | 2,916.95 (2,286.09 to 3,592.72) | 0.00 (-0.03 to 0.04) | 32.05 (41.96 to 23.30) | 1,371.22 (974.24 to 1,823.16) | 0.02 (-0.01 to 0.052) |
| Female | 126.78 (110.3 to 144.32) | 11,632.89 (9,878.16 to 13,552.96) | 0.03 (-0.01 to 0.07) | 34.98 (41.18 to 29.17) | 3,210.53 (2,521.09 to 3,968.94) | -0.01 (-0.06 to 0.04) | 15.30 (20.38 to 10.95) | 1,405.63 (978.55 to 1,906.92) | 0.02 (-0.03 to 0.06) |
| Male | 177.56 (152.67 to 205.02) | 14,195.88 (12,045.06 to 16,631.95) | 0.03 (0.00 to 0.07) | 33.27 (38.67 to 28.07) | 2,660.49 (2,076.18 to 3,346.46) | 0.02 (0.00 to 0.06) | 16.74 (21.80 to 12.33) | 1,341.00 (971.34 to 1,768.16) | 0.03 (-0.00 to 0.06) |
| **Colombia** | | | | | | | | | |
| Total | 18.80 (15.73 to 22.67) | 15,608.60 (12,763.20 to 19,149.32) | 0.12 (0.01 to 0.25) | 5.46 (6.88 to 4.24) | 4,504.76 (3,380.44 to 5,837.71) | 0.21 (0.05 to 0.39) | 2.41 (3.30 to 1.69) | 1,977.05 (1,346.30 to 2,745.35) | 0.15 (0.02 to 0.31) |
| Female | 9.26 (7.67 to 11.3) | 15,675.26 (12,567.51 to 19,595.99) | 0.15 (0.01 to 0.31) | 2.84 (3.62 to 2.18) | 4,780.72 (3,563.42 to 6,285.88) | 0.21 (0.04 to 0.42) | 1.25 (1.75 to 0.85) | 2,080.64 (1,374.95 to 2,932.22) | 0.17 (0.02 to 0.37) |
| Male | 9.54 (8.08 to 11.32) | 15,544.24 (12,912.25 to 18,769.74) | 0.10 (0.00 to 0.20) | 2.63 (3.33 to 2.07) | 4,242.49 (3,176.55 to 5,550.53) | 0.21 (0.04 to 0.41) | 1.17 (1.58 to 0.83) | 1,878.39 (1,294.83 to 2,594.49) | 0.13 (-0.00 to 0.28) |
| **Comoros** | | | | | | | | | |
| Total | 0.28 (0.24 to 0.33) | 12,876.64 (10,694.48 to 15,609.58) | 0.11 (0.02 to 0.22) | 0.12 (0.17 to 0.09) | 5,545.26 (3,893.58 to 7,757.52) | 0.21 (0.04 to 0.42) | 0.04 (0.05 to 0.03) | 1,842.40 (1,265.95 to 2,536.66) | 0.13 (0.03 to 0.24) |
| Female | 0.14 (0.12 to 0.17) | 13,107.66 (10,677.74 to 16,235.89) | 0.13 (0.02 to 0.26) | 0.07 (0.10 to 0.05) | 6,222.11 (4,274.12 to 9,035.62) | 0.23 (0.05 to 0.45) | 0.02 (0.03 to 0.01) | 1,938.68 (1,309.82 to 2,703.40) | 0.15 (0.03 to 0.30) |
| Male | 0.14 (0.12 to 0.16) | 12,640.39 (10,580.62 to 15,048.72) | 0.09 (0.01 to 0.19) | 0.05 (0.07 to 0.04) | 4,874.91 (3,480.15 to 6,759.46) | 0.19 (0.03 to 0.39) | 0.02 (0.03 to 0.01) | 1,746.38 (1,221.89 to 2,402.79) | 0.11 (0.01 to 0.22) |
| **Congo** | | | | | | | | | |
| Total | 2.34 (1.99 to 2.79) | 14,206.60 (11,633.33 to 17,273.49) | 0.10 (0.00 to 0.22) | 1.35 (1.89 to 0.95) | 8,235.13 (5,556.96 to 12,065.44) | 0.18 (-0.03 to 0.40) | 0.36 (0.48 to 0.25) | 2,175.11 (1,483.44 to 3,048.60) | 0.12 (-0.00 to 0.26) |
| Female | 1.21 (1.00 to 1.49) | 14,570.17 (11,646.29 to 18,249.23) | 0.12 (0.00 to 0.26) | 0.76 (1.08 to 0.53) | 9,219.38 (6,120.79 to 13,737.01) | 0.19 (-0.04 to 0.46) | 0.19 (0.26 to 0.13) | 2,301.52 (1,538.64 to 3,272.09) | 0.14 (-0.01 to 0.30) |
| Male | 1.13 (0.97 to 1.31) | 13,822.37 (11,531.66 to 16,390.63) | 0.08 (-0.02 to 0.18) | 0.58 (0.81 to 0.42) | 7,221.38 (4,911.28 to 10,434.64) | 0.16 (-0.06 to 0.40) | 0.17 (0.22 to 0.12) | 2,044.14 (1,401.48 to 2,827.67) | 0.10 (-0.03 to 0.24) |
| **Cook Islands** | | | | | | | | | |
| Total | 0.01 (0.00 to 0.01) | 13,252.18 (10,878.45 to 16,077.87) | 0.09 (-0.01 to 0.20) | 0.00 (0.00 to 0.00) | 5,040.56 (3,494.84 to 7,131.11) | 0.16 (0.00 to 0.34) | 0.00 (0.00 to 0.00) | 1,771.85 (1,202.05 to 2,502.69) | 0.11 (0.00 to 0.22) |
| Female | 0.00 (0.00 to 0.00) | 13,165.53 (10,539.80 to 16,452.44) | 0.11 (-0.01 to 0.26) | 0.00 (0.00 to 0.00) | 5,335.11 (3,664.86 to 7,541.29) | 0.17 (-0.01 to 0.39) | 0.00 (0.00 to 0.00) | 1,847.33 (1,214.74 to 2,650.76) | 0.13 (-0.02 to 0.27) |
| Male | 0.00 (0.00 to 0.00) | 13,296.25 (11,080.79 to 15,822.23) | 0.06 (-0.01 to 0.14) | 0.00 (0.00 to 0.00) | 4,719.39 (3,275.66 to 6,743.42) | 0.14 (-0.01 to 0.32) | 0.00 (0.00 to 0.00) | 1,686.94 (1,174.30 to 2,349.67) | 0.09 (-0.02 to 0.19) |
| **Costa Rica** | | | | | | | | | |
| Total | 1.75 (1.49 to 2.06) | 15,946.57 (13,214.80 to 19,217.42) | 0.13 (0.03 to 0.23) | 0.65 (0.84 to 0.49) | 5,811.62 (4,222.62 to 7,874.80) | 0.28 (0.10 to 0.49) | 0.23 (0.32 to 0.16) | 2,108.93 (1,450.41 to 2,932.79) | 0.17 (0.04 to 0.29) |
| Female | 0.90 (0.75 to 1.09) | 16,314.38 (13,124.25 to 20,135.56) | 0.17 (0.05 to 0.28) | 0.38 (0.51 to 0.28) | 6,831.78 (4,844.03 to 9,517.30) | 0.30 (0.10 to 0.55) | 0.13 (0.18 to 0.09) | 2,324.32 (1,556.59 to 3,296.62) | 0.20 (0.06 to 0.34) |
| Male | 0.85 (0.73 to 0.98) | 15,557.42 (13,053.95 to 18,398.73) | 0.10 (0.02 to 0.17) | 0.26 (0.33 to 0.21) | 4,783.61 (3,518.17 to 6,299.45) | 0.24 (0.08 to 0.46) | 0.10 (0.14 to 0.07) | 1,890.79 (1,316.42 to 2,592.29) | 0.14 (0.02 to 0.26) |
| **Croatia** | | | | | | | | | |
| Total | 0.90 (0.77 to 1.05) | 13,532.38 (11,206.62 to 16,322.67) | 0.15 (0.05 to 0.26) | 0.33 (0.43 to 0.25) | 4,985.92 (3,605.70 to 6,743.88) | 0.30 (0.11 to 0.52) | 0.12 (0.17 to 0.09) | 1,825.46 (1,267.82 to 2,525.54) | 0.19 (0.07 to 0.32) |
| Female | 0.45 (0.37 to 0.55) | 13,821.45 (11,038.43 to 17,336.78) | 0.19 (0.06 to 0.34) | 0.19 (0.25 to 0.14) | 5,675.21 (3,972.26 to 7,846.88) | 0.33 (0.13 to 0.57) | 0.06 (0.09 to 0.04) | 1,974.45 (1,326.92 to 2,809.11) | 0.22 (0.08 to 0.39) |
| Male | 0.45 (0.39 to 0.52) | 13,255.51 (11,172.05 to 15,617.72) | 0.11 (0.03 to 0.19) | 0.15 (0.19 to 0.11) | 4,330.98 (3,153.10 to 5,813.08) | 0.26 (0.08 to 0.48) | 0.06 (0.08 to 0.04) | 1,683.81 (1,189.32 to 2,277.57) | 0.15 (0.04 to 0.29) |
| **Cuba** | | | | | | | | | |
| Total | 3.67 (3.14 to 4.32) | 18,690.02 (15,606.00 to 22,502.84) | 0.12 (0.04 to 0.21) | 1.23 (1.65 to 0.94) | 6,171.91 (4,499.45 to 8,553.40) | 0.33 (0.13 to 0.53) | 0.43 (0.58 to 0.31) | 2,164.54 (1,509.50 to 2,965.15) | 0.19 (0.07 to 0.32) |
| Female | 1.71 (1.43 to 2.05) | 17,915.94 (14,564.64 to 22,134.92) | 0.17 (0.06 to 0.29) | 0.73 (1.03 to 0.54) | 7,584.75 (5,311.47 to 10,893.14) | 0.35 (0.13 to 0.61) | 0.24 (0.32 to 0.16) | 2,426.48 (1,633.47 to 3,401.86) | 0.22 (0.08 to 0.39) |
| Male | 1.95 (1.66 to 2.29) | 19,418.21 (16,274.14 to 23,311.54) | 0.08 (0.02 to 0.14) | 0.50 (0.64 to 0.39) | 4,851.50 (3,638.48 to 6,422.81) | 0.29 (0.10 to 0.49) | 0.20 (0.26 to 0.14) | 1,919.90 (1,369.69 to 2,601.99) | 0.15 (0.04 to 0.27) |
| **Cyprus** | | | | | | | | | |
| Total | 0.44 (0.36 to 0.52) | 20,099.35 (16,344.13 to 24,606.55) | 0.11 (0.01 to 0.24) | 0.16 (0.21 to 0.12) | 7,461.39 (5,288.88 to 10,336.44) | 0.21 (0.04 to 0.42) | 0.06 (0.09 to 0.04) | 2,826.09 (1,891.68 to 3,990.73) | 0.13 (0.02 to 0.27) |
| Female | 0.23 (0.19 to 0.28) | 22,302.18 (17,591.70 to 27,875.97) | 0.13 (0.01 to 0.27) | 0.10 (0.14 to 0.07) | 9,845.94 (6,844.94 to 13,881.72) | 0.22 (0.04 to 0.46) | 0.04 (0.05 to 0.02) | 3,369.25 (2,199.40 to 4,821.07) | 0.15 (0.02 to 0.30) |
| Male | 0.20 (0.17 to 0.24) | 18,045.04 (15,014.44 to 21,743.79) | 0.09 (0.00 to 0.21) | 0.06 (0.07 to 0.04) | 5,241.28 (3,820.37 to 6,985.66) | 0.19 (0.03 to 0.38) | 0.03 (0.04 to 0.02) | 2,320.13 (1,586.48 to 3,275.42) | 0.11 (-0.01 to 0.24) |
| **Czechia** | | | | | | | | | |
| Total | 2.08 (1.75 to 2.44) | 13,356.42 (10,974.23 to 16,047.02) | 0.16 (0.06 to 0.26) | 0.79 (1.02 to 0.59) | 5,063.43 (3,605.39 to 6,806.18) | 0.34 (0.15 to 0.57) | 0.28 (0.38 to 0.20) | 1,812.37 (1,241.38 to 2,485.41) | 0.20 (0.09 to 0.33) |
| Female | 1.03 (0.84 to 1.26) | 13,666.79 (10,919.48 to 16,962.85) | 0.21 (0.09 to 0.35) | 0.44 (0.59 to 0.33) | 5,884.99 (4,065.02 to 8,222.88) | 0.38 (0.16 to 0.63) | 0.15 (0.21 to 0.10) | 1,973.07 (1,323.19 to 2,766.59) | 0.24 (0.09 to 0.41) |
| Male | 1.04 (0.90 to 1.20) | 13,062.30 (11,008.20 to 15,349.31) | 0.11 (0.04 to 0.19) | 0.34 (0.42 to 0.27) | 4,283.25 (3,083.32 to 5,632.17) | 0.29 (0.12 to 0.49) | 0.13 (0.18 to 0.09) | 1,659.80 (1,158.19 to 2,263.58) | 0.15 (0.05 to 0.27) |
| **Democratic People's Republic of Korea** | | | | | | | | | |
| Total | 6.83 (5.68 to 8.20) | 12,599.61 (10,444.68 to 15,241.31) | 0.03 (-0.05 to 0.14) | 1.66 (2.09 to 1.29) | 3,051.90 (2,280.58 to 3,990.09) | 0.07 (-0.06 to 0.22) | 0.79 (1.07 to 0.56) | 1,442.22 (994.66 to 1,977.33) | 0.04 (-0.05 to 0.17) |
| Female | 3.23 (2.63 to 4.08) | 12,081.71 (9,695.24 to 15,374.14) | 0.04 (-0.07 to 0.2) | 0.97 (1.24 to 0.73) | 3,592.86 (2,609.77 to 4,834.38) | 0.08 (-0.09 to 0.26) | 0.41 (0.56 to 0.29) | 1,520.22 (1,023.02 to 2,151.11) | 0.05 (-0.07 to 0.22) |
| Male | 3.60 (3.05 to 4.23) | 13,093.96 (10,964.22 to 15,582.30) | 0.02 (-0.03 to 0.10) | 0.70 (0.86 to 0.56) | 2,532.79 (1,909.77 to 3,265.49) | 0.06 (-0.05 to 0.21) | 0.38 (0.51 to 0.27) | 1,367.29 (963.35 to 1,844.12) | 0.03 (-0.05 to 0.15) |
| **Democratic Republic of the Congo** | | | | | | | | | |
| Total | 41.94 (35.57 to 48.84) | 14,235.55 (11,727.93 to 17,160.04) | 0.06 (-0.03 to 0.16) | 22.18 (30.87 to 15.97) | 7,626.70 (5,213.22 to 10,940.78) | 0.10 (-0.07 to 0.32) | 6.02 (8.27 to 4.32) | 2,060.95 (1,442.84 to 2,901.39) | 0.07 (-0.04 to 0.19) |
| Female | 20.8 (17.37 to 24.52) | 14,389.01 (11,520.15 to 17,606.15) | 0.07 (-0.04 to 0.18) | 12.14 (16.92 to 8.63) | 8,492.31 (5,722.94 to 12,285.81) | 0.11 (-0.08 to 0.35) | 3.10 (4.28 to 2.20) | 2,160.42 (1,472.27 to 3,036.36) | 0.08 (-0.05 to 0.23) |
| Male | 21.13 (18.09 to 24.41) | 14,086.86 (11,722.64 to 16,788.41) | 0.04 (-0.03 to 0.14) | 10.03 (14.02 to 7.26) | 6,787.15 (4,673.91 to 9,762.89) | 0.09 (-0.08 to 0.32) | 2.92 (4.01 to 2.13) | 1,964.48 (1,379.17 to 2,758.88) | 0.06 (-0.05 to 0.18) |
| **Denmark** | | | | | | | | | |
| Total | 2.00 (1.70 to 2.36) | 19,046.75 (15,699.90 to 23,020.55) | 0.13 (0.03 to 0.24) | 0.80 (1.05 to 0.6) | 7,539.01 (5,380.07 to 10,306.33) | 0.25 (0.07 to 0.45) | 0.29 (0.39 to 0.20) | 2,756.12 (1,906.53 to 3,718.50) | 0.15 (0.03 to 0.289) |
| Female | 1.11 (0.91 to 1.35) | 21,487.12 (17,241.19 to 26,877.78) | 0.15 (0.04 to 0.28) | 0.51 (0.68 to 0.37) | 9,845.59 (6,829.06 to 13,644.43) | 0.26 (0.07 to 0.50) | 0.17 (0.23 to 0.12) | 3,253.38 (2,189.32 to 4,549.90) | 0.17 (0.04 to 0.33) |
| Male | 0.90 (0.77 to 1.04) | 16,722.72 (14,070.72 to 19,711.15) | 0.10 (0.00 to 0.21) | 0.29 (0.37 to 0.22) | 5,343.97 (3,940.71 to 7,119.91) | 0.22 (0.05 to 0.40) | 0.12 (0.16 to 0.09) | 2,282.71 (1,624.29 to 3,048.85) | 0.12 (0.01 to 0.25) |
| **Djibouti** | | | | | | | | | |
| Total | 0.45 (0.39 to 0.52) | 12,661.94 (10,531.78 to 15,189.55) | 0.09 (0.00 to 0.18) | 0.20 (0.27 to 0.15) | 5,714.10 (3,979.15 to 8,001.63) | 0.17 (0.00 to 0.36) | 0.07 (0.09 to 0.05) | 1,834.92 (1,265.83 to 2,514.33) | 0.11 (-0.00 to 0.22) |
| Female | 0.20 (0.17 to 0.25) | 12,916.18 (10,456.27 to 15,827.26) | 0.11 (0.00 to 0.22) | 0.10 (0.14 to 0.07) | 6,528.30 (4,376.17 to 9,363.40) | 0.19 (0.00 to 0.40) | 0.03 (0.04 to 0.02) | 1,947.47 (1,299.27 to 2,722.23) | 0.12 (0.00 to 0.26) |
| Male | 0.25 (0.21 to 0.28) | 12,459.47 (10,444.08 to 14,695.16) | 0.07 (0.00 to 0.15) | 0.10 (0.13 to 0.07) | 5,062.24 (3,564.90 to 6,973.91) | 0.15 (-0.01 to 0.34) | 0.03 (0.05 to 0.03) | 1,744.87 (1,215.77 to 2,379.25) | 0.09 (-0.02 to 0.22) |
| **Dominica** | | | | | | | | | |
| Total | 0.03 (0.03 to 0.04) | 18,296.86 (15,192.50 to 21,857.51) | 0.11 (0.03 to 0.20) | 0.01 (0.01 to 0.01) | 5,630.80 (3,988.59 to 7,664.13) | 0.30 (0.12 to 0.52) | 0.00 (0.00 to 0.00) | 2,075.95 (1,436.61 to 2,842.71) | 0.18 (0.06 to 0.31) |
| Female | 0.01 (0.01 to 0.02) | 17,447.04 (14,039.48 to 21,395.20) | 0.16 (0.05 to 0.29) | 0.01 (0.01 to 0.00) | 6,922.51 (4,814.28 to 9,663.85) | 0.33 (0.13 to 0.57) | 0.00 (0.00 to 0.00) | 2,322.64 (1,562.54 to 3,255.53) | 0.21 (0.07 to 0.37) |
| Male | 0.02 (0.01 to 0.02) | 19,117.53 (15,871.98 to 22,788.87) | 0.07 (0.02 to 0.13) | 0.00 (0.00 to 0.00) | 4,421.24 (3,221.17 to 5,812.71) | 0.25 (0.08 to 0.46) | 0.00 (0.00 to 0.00) | 1,845.68 (1,291.27 to 2,531.22) | 0.14 (0.02 to 0.26) |
| **Dominican Republic** | | | | | | | | | |
| Total | 5.20 (4.46 to 6.12) | 18,141.94 (15,077.09 to 21,750.55) | 0.08 (0.00 to 0.16) | 1.75 (2.36 to 1.31) | 6,015.51 (4,237.56 to 8,419.60) | 0.20 (0.04 to 0.39) | 0.61 (0.84 to 0.43) | 2,095.03 (1,461.71 to 2,925.94) | 0.12 (0.00 to 0.25) |
| Female | 2.50 (2.08 to 2.99) | 17,427.34 (14,067.97 to 21,301.70) | 0.11 (0.01 to 0.22) | 1.10 (1.53 to 0.79) | 7,627.62 (5,230.72 to 11,039.99) | 0.22 (0.04 to 0.45) | 0.34 (0.48 to 0.24) | 2,376.41 (1,613.36 to 3,366.59) | 0.15 (0.02 to 0.30) |
| Male | 2.71 (2.29 to 3.18) | 18,828.80 (15,618.78 to 22,496.57) | 0.05 (0.00 to 0.10) | 0.64 (0.84 to 0.49) | 4,423.48 (3,215.36 to 5,927.48) | 0.17 (0.02 to 0.35) | 0.26 (0.36 to 0.19) | 1,816.54 (1,274.39 to 2,510.23) | 0.09 (-0.01 to 0.20) |
| **Ecuador** | | | | | | | | | |
| Total | 9.07 (7.61 to 10.72) | 18,599.93 (15,293.56 to 22,416.12) | 0.17 (0.06 to 0.31) | 3.39 (4.42 to 2.58) | 6,927.68 (4,938.86 to 9,439.07) | 0.38 (0.16 to 0.63) | 1.17 (1.59 to 0.82) | 2,395.20 (1,631.26 to 3,329.85) | 0.23 (0.09 to 0.41) |
| Female | 4.64 (3.83 to 5.58) | 19,219.48 (15,450.19 to 23,833.86) | 0.22 (0.09 to 0.40) | 2.08 (2.77 to 1.55) | 8,582.54 (6,011.87 to 11,902.45) | 0.41 (0.16 to 0.70) | 0.67 (0.92 to 0.46) | 2,753.19 (1,838.75 to 3,855.15) | 0.27 (0.10 to 0.48) |
| Male | 4.43 (3.76 to 5.20) | 17,988.41 (14,911.79 to 21,597.30) | 0.12 (0.04 to 0.23) | 1.31 (1.71 to 1.01) | 5,311.15 (3,871.03 to 7,180.79) | 0.33 (0.15 to 0.55) | 0.51 (0.69 to 0.36) | 2,045.18 (1,402.38 to 2,819.04) | 0.19 (0.06 to 0.34) |
| **Egypt** | | | | | | | | | |
| Total | 54.24 (45.34 to 64.45) | 18,268.55 (14,841.17 to 21,983.40) | 0.11 (0.02 to 0.23) | 22.44 (30.05 to 16.26) | 7,566.94 (5,183.66 to 10,710.22) | 0.23 (0.05 to 0.44) | 7.42 (10.02 to 5.13) | 2,504.36 (1,683.89 to 3,444.87) | 0.15 (0.03 to 0.28) |
| Female | 27.32 (22.2 to 33.16) | 19,122.20 (15,215.17 to 23,567.00) | 0.14 (0.02 to 0.29) | 13.1 (18.1 to 9.16) | 9,177.34 (6,094.85 to 13,236.57) | 0.25 (0.05 to 0.49) | 4.02 (5.49 to 2.71) | 2,815.75 (1,843.74 to 3,931.23) | 0.17 (0.03 to 0.33) |
| Male | 26.93 (22.79 to 31.52) | 17,457.84 (14,572.99 to 20,870.30) | 0.09 (0.00 to 0.19) | 9.33 (12.16 to 6.89) | 6,064.42 (4,217.26 to 8,387.46) | 0.21 (0.03 to 0.40) | 3.40 (4.63 to 2.39) | 2,212.54 (1,526.81 to 3,047.34) | 0.12 (0.00 to 0.26) |
| **El Salvador** | | | | | | | | | |
| Total | 2.85 (2.43 to 3.41) | 16,230.05 (13,532.88 to 19,610.74) | 0.12 (0.03 to 0.23) | 1.10 (1.45 to 0.84) | 6,219.37 (4,485.80 to 8,515.21) | 0.24 (0.06 to 0.45) | 0.38 (0.52 to 0.27) | 2,147.10 (1,487.14 to 2,963.26) | 0.15 (0.03 to 0.28) |
| Female | 1.49 (1.23 to 1.82) | 16,869.48 (13,616.28 to 20,894.17) | 0.16 (0.04 to 0.28) | 0.70 (0.94 to 0.52) | 7,844.98 (5,529.26 to 10,988.60) | 0.27 (0.05 to 0.51) | 0.22 (0.30 to 0.15) | 2,432.92 (1,622.24 to 3,410.46) | 0.18 (0.03 to 0.34) |
| Male | 1.36 (1.16 to 1.60) | 15,542.09 (18,584.71 to 13,024.80) | 0.09 (0.01 to 0.17) | 0.40 (0.52 to 0.32) | 4,574.48 (3,348.70 to 6,160.98) | 0.21 (0.05 to 0.40) | 0.16 (0.22 to 0.12) | 1,855.32 (1,304.39 to 2,528.94) | 0.12 (0.01 to 0.25) |
| **Equatorial Guinea** | | | | | | | | | |
| Total | 0.76 (0.64 to 0.92) | 13,961.96 (11,358.99 to 17,177.39) | 0.08 (-0.03 to 0.19) | 0.45 (0.62 to 0.33) | 8,200.15 (5,609.70 to 11,755.94) | 0.13 (-0.06 to 0.34) | 0.12 (0.16 to 0.08) | 2,169.31 (1,488.96 to 3,091.25) | 0.09 (-0.03 to 0.23) |
| Female | 0.33 (0.27 to 0.40) | 14,391.68 (11,391.64 to 18,158.05) | 0.09 (-0.03 to 0.23) | 0.21 (0.30 to 0.15) | 9,315.38 (6,219.68 to 13,747.81) | 0.14 (-0.06 to 0.36) | 0.05 (0.08 to 0.04) | 2,317.55 (1,567.55 to 3,380.75) | 0.10 (-0.03 to 0.26) |
| Male | 0.43 (0.37 to 0.51) | 13,660.52 (11,259.42 to 16,434.04) | 0.07 (-0.02 to 0.17) | 0.24 (0.32 to 0.17) | 7,405.69 (5,177.16 to 10,491.74) | 0.12 (-0.07 to 0.35) | 0.07 (0.09 to 0.05) | 2,064.04 (1,420.91 to 2,895.06) | 0.08 (-0.04 to 0.23) |
| **Eritrea** | | | | | | | | | |
| Total | 2.70 (2.32 to 3.15) | 12,999.83 (10,779.14 to 15,517.07) | 0.07 (-0.02 to 0.18) | 1.20 (1.62 to 0.88) | 5,792.87 (4,012.49 to 8,114.80) | 0.14 (-0.02 to 0.36) | 0.39 (0.52 to 0.27) | 1,855.50 (1,281.65 to 2,580.74) | 0.09 (-0.02 to 0.21) |
| Female | 1.32 (1.1 to 1.56) | 13,188.58 (10,622.64 to 16,052.61) | 0.09 (-0.03 to 0.22) | 0.65 (0.89 to 0.46) | 6,456.00 (4,423.45 to 9,393.23) | 0.15 (-0.03 to 0.39) | 0.19 (0.27 to 0.14) | 1,949.13 (1,314.27 to 2,773.89) | 0.10 (-0.03 to 0.25) |
| Male | 1.38 (1.2 to 1.58) | 12,826.26 (10,834.27 to 15,082.82) | 0.06 (-0.02 to 0.14) | 0.56 (0.74 to 0.41) | 5,177.69 (3,609.45 to 7,109.47) | 0.13 (-0.03 to 0.37) | 0.19 (0.25 to 0.14) | 1,768.77 (1,239.91 to 2,416.69) | 0.07 (-0.03 to 0.19) |
| **Estonia** | | | | | | | | | |
| Total | 0.30 (0.25 to 0.35) | 14,778.23 (12,151.89 to 18,002.60) | 0.20 (0.08 to 0.33) | 0.13 (0.17 to 0.09) | 6,236.27 (4,484.49 to 8,590.95) | 0.41 (0.20 to 0.68) | 0.04 (0.06 to 0.03) | 2,057.40 (1,416.19 to 2,883.81) | 0.25 (0.11 to 0.41) |
| Female | 0.15 (0.12 to 0.18) | 15,307.66 (12,277.18 to 19,293.64) | 0.25 (0.10 to 0.43) | 0.07 (0.09 to 0.05) | 7,141.66 (5,029.82 to 10,117.76) | 0.45 (0.21 to 0.73) | 0.02 (0.03 to 0.02) | 2,250.67 (1,512.87 to 3,208.81) | 0.30 (0.11 to 0.48) |
| Male | 0.15 (0.13 to 0.17) | 14,276.48 (11,887.43 to 16,851.02) | 0.15 (0.06 to 0.26) | 0.06 (0.07 to 0.04) | 5,377.13 (3,854.70 to 7,220.64) | 0.37 (0.17 to 0.65) | 0.02 (0.03 to 0.01) | 1,874.04 (1,312.99 to 2,611.03) | 0.21 (0.08 to 0.35) |
| **Eswatini** | | | | | | | | | |
| Total | 0.57 (0.47 to 0.68) | 15,405.36 (12,510.74 to 18,848.54) | 0.30 (0.17 to 0.43) | 0.30 (0.40 to 0.22) | 8,169.84 (5,600.32 to 11,451.96) | 0.57 (0.32 to 0.85) | 0.09 (0.12 to 0.06) | 2,330.36 (1,602.98 to 3,256.47) | 0.36 (0.20 to 0.55) |
| Female | 0.29 (0.23 to 0.35) | 15,959.52 (12,624.02 to 20,004.57) | 0.36 (0.20 to 0.51) | 0.16 (0.22 to 0.11) | 8,952.64 (6,031.36 to 12,790.20) | 0.61 (0.33 to 0.94) | 0.04 (0.06 to 0.03) | 2,471.29 (1,653.43 to 3,502.25) | 0.41 (0.24 to 0.62) |
| Male | 0.28 (0.23 to 0.33) | 14,851.51 (12,272.95 to 17,918.37) | 0.24 (0.13 to 0.36) | 0.14 (0.18 to 0.10) | 7,404.91 (5,144.38 to 10,307.51) | 0.52 (0.27 to 0.79) | 0.04 (0.06 to 0.03) | 2,191.68 (1,500.99 to 3,056.44) | 0.30 (0.15 to 0.48) |
| **Ethiopia** | | | | | | | | | |
| Total | 48.63 (43.33 to 54.56) | 12,941.43 (11,190.61 to 14,824.84) | 0.09 (0.04 to 0.14) | 21.67 (27.14 to 17.46) | 5,784.81 (4,342.92 to 7,581.50) | 0.18 (0.10 to 0.27) | 6.89 (9.02 to 4.99) | 1,840.14 (1,310.18 to 2,460.98) | 0.11 (0.06 to 0.17) |
| Female | 24.25 (21.44 to 27.47) | 13,111.21 (11,247.74 to 15,183.53) | 0.11 (0.05 to 0.17) | 11.26 (14.08 to 9.05) | 6,096.72 (4,553.30 to 7,986.29) | 0.19 (0.10 to 0.30) | 3.52 (4.64 to 2.55) | 1,907.14 (1,337.65 to 2,576.26) | 0.13 (0.06 to 0.19) |
| Male | 24.38 (21.69 to 27.29) | 12,763.75 (11,056.84 to 14,555.20) | 0.07 (0.03 to 0.11) | 10.41 (13.11 to 8.33) | 5,479.26 (4,074.48 to 7,165.42) | 0.17 (0.09 to 0.26) | 3.37 (4.40 to 2.48) | 1,773.59 (1,265.17 to 2,359.66) | 0.10 (0.04 to 0.15) |
| **Fiji** | | | | | | | | | |
| Total | 0.33 (0.27 to 0.39) | 13,639.62 (11,087.11 to 16,570.73) | 0.12 (0.02 to 0.25) | 0.12 (0.16 to 0.09) | 5,087.30 (3,689.83 to 6,916.00) | 0.24 (0.07 to 0.47) | 0.04 (0.06 to 0.03) | 1,791.15 (1,227.79 to 2,529.24) | 0.16 (0.04 to 0.31) |
| Female | 0.16 (0.13 to 0.2) | 13,707.47 (10,762.76 to 17,213.62) | 0.16 (0.02 to 0.33) | 0.06 (0.08 to 0.05) | 5,499.49 (3,916.22 to 7,610.31) | 0.27 (0.08 to 0.53) | 0.02 (0.03 to 0.02) | 1,895.18 (1,266.30 to 2,717.84) | 0.19 (0.03 to 0.38) |
| Male | 0.17 (0.14 to 0.19) | 13,575.56 (11,250.88 to 16,171.78) | 0.09 (0.01 to 0.18) | 0.06 (0.07 to 0.04) | 4,692.22 (3,409.21 to 6,405.76) | 0.22 (0.05 to 0.45) | 0.02 (0.03 to 0.01) | 1,691.49 (1,163.45 to 2,359.13) | 0.13 (0.02 to 0.26) |
| **Finland** | | | | | | | | | |
| Total | 1.83 (1.58 to 2.14) | 19,901.46 (16,759.01 to 23,717.54) | 0.11 (0.01 to 0.21) | 0.91 (1.21 to 0.66) | 9,892.60 (6,936.33 to 13,718.53) | 0.22 (0.03 to 0.47) | 0.27 (0.36 to 0.19) | 2,880.59 (1,988.29 to 3,925.45) | 0.14 (0.02 to 0.27) |
| Female | 0.93 (0.78 to 1.11) | 20,640.98 (16,871.23 to 25,273.99) | 0.14 (0.02 to 0.27) | 0.57 (0.77 to 0.4) | 12,603.87 (8,702.42 to 17,645.62) | 0.24 (0.01 to 0.51) | 0.15 (0.20 to 0.10) | 3,345.27 (2,249.00 to 4,643.96) | 0.16 (0.01 to 0.32) |
| Male | 0.90 (0.78 to 1.04) | 19,205.48 (16,253.34 to 22,604.09) | 0.08 (0.00 to 0.17) | 0.35 (0.45 to 0.25) | 7,318.54 (5,238.56 to 9,934.72) | 0.21 (0.01 to 0.44) | 0.12 (0.15 to 0.08) | 2,439.77 (1,720.89 to 3,304.10) | 0.12 (0.00 to 0.25) |
| **France** | | | | | | | | | |
| Total | 26.77 (22.29 to 32.64) | 21,873.12 (17,797.79 to 26,627.04) | 0.15 (0.04 to 0.27) | 10.63 (13.93 to 7.97) | 8,675.07 (6,268.04 to 11,971.50) | 0.28 (0.10 to 0.50) | 3.81 (5.21 to 2.65) | 3,101.92 (2,108.31 to 4,294.17) | 0.18 (0.05 to 0.32) |
| Female | 15.07 (12.30 to 18.60) | 25,129.05 (19,897.13 to 31,265.05) | 0.18 (0.05 to 0.33) | 6.87 (9.14 to 5.05) | 11,453.32 (8,039.17 to 15,920.99) | 0.29 (0.10 to 0.53) | 2.30 (3.17 to 1.56) | 3,821.46 (2,505.70 to 5,405.93) | 0.20 (0.06 to 0.36) |
| Male | 11.7 (9.93 to 13.86) | 18,751.67 (15,531.17 to 22,448.03) | 0.11 (0.02 to 0.22) | 3.76 (4.86 to 2.89) | 6,013.78 (4,418.26 to 7,990.30) | 0.26 (0.08 to 0.49) | 1.51 (2.03 to 1.07) | 2,412.40 (1,683.09 to 3,300.55) | 0.15 (0.03 to 0.29) |
| **Gabon** | | | | | | | | | |
| Total | 0.82 (0.70 to 0.98) | 14,665.56 (11,971.83 to 17,985.40) | 0.13 (0.03 to 0.25) | 0.49 (0.67 to 0.35) | 8,713.73 (5,968.24 to 12,380.52) | 0.22 (0.03 to 0.48) | 0.13 (0.18 to 0.09) | 2,277.03 (1,543.76 to 3,260.04) | 0.15 (0.03 to 0.30) |
| Female | 0.44 (0.37 to 0.54) | 14,870.42 (11,866.97 to 18,569.25) | 0.15 (0.03 to 0.29) | 0.28 (0.38 to 0.19) | 9,251.05 (6,197.80 to 13,406.77) | 0.23 (0.02 to 0.52) | 0.07 (0.10 to 0.05) | 2,354.12 (1,584.06 to 3,400.15) | 0.17 (0.02 to 0.33) |
| Male | 0.38 (0.33 to 0.45) | 14,420.22 (11,922.82 to 17,434.32) | 0.11 (0.02 to 0.22) | 0.21 (0.29 to 0.15) | 8,104.40 (5,575.67 to 11,343.71) | 0.20 (-0.01 to 0.45) | 0.06 (0.08 to 0.04) | 2,188.49 (1,502.62 to 3,094.44) | 0.13 (0.01 to 0.28) |
| **Gambia** | | | | | | | | | |
| Total | 1.08 (0.92 to 1.27) | 13,305.43 (10,998.57 to 16,234.34) | 0.09 (-0.01 to 0.19) | 0.53 (0.73 to 0.39) | 6,613.59 (4,485.36 to 9,545.98) | 0.16 (-0.02 to 0.34) | 0.16 (0.21 to 0.11) | 1,950.72 (1,329.88 to 2,708.63) | 0.10 (-0.01 to 0.22) |
| Female | 0.60 (0.5 to 0.73) | 14,444.07 (11,506.93 to 18,184.45) | 0.11 (0.00 to 0.23) | 0.37 (0.52 to 0.25) | 8,901.34 (5,787.40 to 13,108.00) | 0.17 (-0.03 to 0.39) | 0.09 (0.13 to 0.06) | 2,263.20 (1,481.00 to 3,220.67) | 0.13 (-0.00 to 0.27) |
| Male | 0.48 (0.42 to 0.55) | 12,094.28 (10,214.01 to 14,274.73) | 0.06 (-0.02 to 0.14) | 0.17 (0.22 to 0.13) | 4,205.60 (3,027.41 to 5,742.35) | 0.13 (-0.02 to 0.29) | 0.06 (0.09 to 0.05) | 1,620.62 (1,132.51 to 2,205.36) | 0.08 (-0.03 to 0.18) |
| **Georgia** | | | | | | | | | |
| Total | 0.79 (0.68 to 0.93) | 12,780.98 (10,634.98 to 15,224.53) | 0.15 (0.07 to 0.25) | 0.34 (0.45 to 0.25) | 5,487.14 (3,815.91 to 7,622.08) | 0.36 (0.17 to 0.62) | 0.11 (0.14 to 0.08) | 1,729.17 (1,186.13 to 2,373.18) | 0.20 (0.09 to 0.33) |
| Female | 0.36 (0.30 to 0.43) | 12,380.65 (10,064.45 to 15,182.13) | 0.20 (0.09 to 0.33) | 0.17 (0.22 to 0.12) | 5,766.06 (3,874.24 to 8,232.58) | 0.39 (0.18 to 0.66) | 0.05 (0.07 to 0.04) | 1,769.94 (1,191.16 to 2,459.97) | 0.24 (0.10 to 0.41) |
| Male | 0.43 (0.37 to 0.50) | 13,137.55 (11,083.09 to 15,490.45) | 0.12 (0.05 to 0.19) | 0.17 (0.23 to 0.13) | 5,240.96 (3,673.73 to 7,218.73) | 0.33 (0.14 to 0.58) | 0.06 (0.07 to 0.04) | 1,693.29 (1,162.36 to 2,311.33) | 0.17 (0.06 to 0.29) |
| **Germany** | | | | | | | | | |
| Total | 25.22 (21.22 to 30.36) | 19,900.09 (16,264.99 to 24,486.06) | 0.17 (0.04 to 0.31) | 10.55 (13.88 to 7.68) | 8,307.03 (5,834.05 to 11,325.56) | 0.29 (0.09 to 0.53) | 3.81 (5.18 to 2.59) | 2,978.84 (2,013.24 to 4,149.70) | 0.19 (0.05 to 0.33) |
| Female | 14.10 (11.53 to 17.33) | 23,411.37 (18,516.75 to 29,334.13) | 0.19 (0.05 to 0.35) | 6.40 (8.62 to 4.61) | 10,621.38 (7,322.90 to 14,765.45) | 0.29 (0.07 to 0.53) | 2.20 (3.03 to 1.47) | 3,634.94 (2,384.31 to 5,153.32) | 0.21 (0.06 to 0.37) |
| Male | 11.12 (9.58 to 13.09) | 16,755.24 (13,917.61 to 20,187.92) | 0.14 (0.03 to 0.28) | 4.15 (5.36 to 3.07) | 6,224.92 (4,463.39 to 8,331.42) | 0.29 (0.10 to 0.55) | 1.61 (2.16 to 1.12) | 2,390.82 (1,635.01 to 3,285.10) | 0.17 (0.04 to 0.32) |
| **Ghana** | | | | | | | | | |
| Total | 12.76 (10.79 to 15.00) | 12,169.03 (9,995.40 to 14,696.93) | 0.07 (-0.02 to 0.17) | 5.54 (7.74 to 4.12) | 5,299.37 (3,707.17 to 7,561.89) | 0.13 (-0.03 to 0.32) | 1.83 (2.42 to 1.27) | 1,747.60 (1,199.97 to 2,387.96) | 0.08 (-0.02 to 0.20) |
| Female | 6.49 (5.37 to 7.85) | 12,373.51 (9,899.94 to 15,222.34) | 0.09 (-0.02 to 0.20) | 3.14 (4.43 to 2.26) | 5,990.54 (4,058.91 to 8,832.33) | 0.14 (-0.04 to 0.35) | 0.97 (1.31 to 0.66) | 1,844.94 (1,228.79 to 2,543.07) | 0.10 (-0.03 to 0.23) |
| Male | 6.26 (5.40 to 7.28) | 11,946.63 (9,980.66 to 14,229.82) | 0.06 (-0.02 to 0.14) | 2.40 (3.24 to 1.82) | 4,597.41 (3,284.44 to 6,479.17) | 0.12 (-0.04 to 0.30) | 0.86 (1.13 to 0.61) | 1,647.64 (1,144.96 to 2,237.34) | 0.07 (-0.02 to 0.18) |
| **Greece** | | | | | | | | | |
| Total | 3.50 (2.94 to 4.17) | 22,625.01 (18,461.51 to 27,652.44) | 0.18 (0.06 to 0.32) | 1.76 (2.43 to 1.30) | 11,367.00 (7,762.38 to 16,167.56) | 0.31 (0.10 to 0.55) | 0.53 (0.72 to 0.36) | 3,404.10 (2,278.57 to 4,784.13) | 0.21 (0.08 to 0.37) |
| Female | 1.94 (1.58 to 2.39) | 25,470.18 (20,297.79 to 32,098.71) | 0.21 (0.08 to 0.37) | 1.13 (1.58 to 0.80) | 14,783.83 (9,809.76 to 21,344.15) | 0.33 (0.10 to 0.56) | 0.31 (0.43 to 0.21) | 4,099.20 (2,672.80 to 5,833.33) | 0.24 (0.10 to 0.41) |
| Male | 1.56 (1.34 to 1.84) | 19,876.11 (16,505.18 to 23,908.64) | 0.13 (0.03 to 0.25) | 0.64 (0.85 to 0.47) | 8,070.34 (5,574.41 to 11,356.66) | 0.27 (0.08 to 0.52) | 0.22 (0.29 to 0.15) | 2,733.11 (1,854.09 to 3,746.25) | 0.17 (0.04 to 0.32) |
| **Greenland** | | | | | | | | | |
| Total | 0.03 (0.02 to 0.03) | 23,615.11 (19,615.74 to 28,600.73) | 0.18 (0.07 to 0.30) | 0.02 (0.03 to 0.01) | 17,425.25 (12,045.83 to 24,524.45) | 0.37 (0.12 to 0.66) | 0.00 (0.01 to 0.00) | 3,790.60 (2,612.22 to 5,383.60) | 0.25 (0.09 to 0.42) |
| Female | 0.01 (0.01 to 0.02) | 25,435.20 (20,640.07 to 31,440.83) | 0.23 (0.09 to 0.38) | 0.01 (0.02 to 0.01) | 22,445.63 (15,184.82 to 31,757.38) | 0.39 (0.11 to 0.69) | 0.00 (0.00 to 0.00) | 4,529.94 (3,026.00 to 6,570.43) | 0.28 (0.09 to 0.47) |
| Male | 0.01 (0.01 to 0.01) | 21,812.44 (18,374.89 to 26,091.04) | 0.13 (0.05 to 0.23) | 0.01 (0.01 to 0.01) | 12,542.29 (8,896.78 to 17,737.99) | 0.35 (0.01 to 0.65) | 0.00 (0.00 to 0.00) | 3,069.30 (2,132.24 to 4,270.90) | 0.21 (0.07 to 0.37) |
| **Grenada** | | | | | | | | | |
| Total | 0.05 (0.04 to 0.06) | 18,385.76 (15,290.18 to 22,253.76) | 0.11 (0.02 to 0.20) | 0.02 (0.02 to 0.01) | 5,866.07 (4,192.46 to 7,962.31) | 0.28 (0.09 to 0.49) | 0.01 (0.01 to 0) | 2,105.22 (1,422.35 to 2,928.04) | 0.16 (0.04 to 0.31) |
| Female | 0.02 (0.02 to 0.03) | 17,449.64 (14,077.35 to 21,777.57) | 0.15 (0.04 to 0.28) | 0.01 (0.01 to 0.01) | 7,033.85 (4,878.05 to 9,868.64) | 0.31 (0.09 to 0.55) | 0.00 (0.00 to 0.00) | 2,338.96 (1,537.81 to 3,311.34) | 0.19 (0.04 to 0.36) |
| Male | 0.03 (0.02 to 0.03) | 19,254.79 (16,060.31 to 23,098.43) | 0.07 (0.01 to 0.14) | 0.01 (0.01 to 0.01) | 4,821.45 (3,497.60 to 6,370.62) | 0.25 (0.06 to 0.45) | 0.00 (0.00 to 0.00) | 1,897.09 (1,306.48 to 2,609.72) | 0.13 (0.02 to 0.27) |
| **Guam** | | | | | | | | | |
| Total | 0.05 (0.04 to 0.06) | 14,118.50 (11,605.31 to 17,126.66) | 0.14 (0.02 to 0.27) | 0.02 (0.03 to 0.02) | 6,112.50 (4,341.91 to 8,492.44) | 0.27 (0.08 to 0.5) | 0.01 (0.01 to 0.00) | 1,951.51 (1,335.64 to 2,725.12) | 0.18 (0.04 to 0.33) |
| Female | 0.02 (0.02 to 0.03) | 13,881.66 (11,160.08 to 17,553.54) | 0.17 (0.02 to 0.34) | 0.01 (0.01 to 0.01) | 5,948.37 (4,226.60 to 8,231.21) | 0.28 (0.06 to 0.52) | 0.00 (0.00 to 0.00) | 1,983.87 (1,327.94 to 2,838.65) | 0.20 (0.02 to 0.37) |
| Male | 0.03 (0.02 to 0.03) | 14,345.99 (11,892.03 to 17,109.67) | 0.11 (0.02 to 0.21) | 0.01 (0.02 to 0.01) | 6,252.58 (4,418.76 to 8,725.27) | 0.27 (0.05 to 0.52) | 0.00 (0.00 to 0.00) | 1,920.90 (1,327.12 to 2,669.44) | 0.16 (0.03 to 0.31) |
| **Guatemala** | | | | | | | | | |
| Total | 8.01 (6.79 to 9.50) | 16,118.46 (13,357.80 to 19,557.24) | 0.13 (0.03 to 0.24) | 3.04 (4.11 to 2.25) | 6,068.15 (4,260.35 to 8,503.51) | 0.28 (0.08 to 0.48) | 1.06 (1.45 to 0.75) | 2,111.11 (1,469.08 to 2,938.24) | 0.17 (0.05 to 0.31) |
| Female | 4.20 (3.53 to 5.09) | 16,700.20 (13,440.34 to 20,755.47) | 0.17 (0.05 to 0.30) | 1.90 (2.65 to 1.36) | 7,497.22 (5,097.30 to 10,866.16) | 0.30 (0.09 to 0.55) | 0.60 (0.83 to 0.43) | 2,371.61 (1,615.21 to 3,372.23) | 0.21 (0.06 to 0.37) |
| Male | 3.80 (3.25 to 4.44) | 15,493.82 (12,935.46 to 18,421.38) | 0.09 (0.02 to 0.19) | 1.13 (1.47 to 0.88) | 4,598.22 (3,351.92 to 6,166.62) | 0.23 (0.06 to 0.44) | 0.45 (0.62 to 0.32) | 1,841.61 (1,290.75 to 2,555.61) | 0.13 (0.02 to 0.25) |
| **Guinea** | | | | | | | | | |
| Total | 5.39 (4.56 to 6.38) | 12,538.42 (10,328.45 to 15,161.25) | 0.08 (0.00 to 0.18) | 2.24 (2.99 to 1.66) | 5,274.02 (3,629.72 to 7,380.47) | 0.14 (-0.01 to 0.35) | 0.75 (1.03 to 0.53) | 1,768.76 (1,230.89 to 2,463.36) | 0.09 (-0.00 to 0.22) |
| Female | 2.84 (2.35 to 3.43) | 12,886.66 (10,354.03 to 16,036.52) | 0.10 (0.00 to 0.22) | 1.35 (1.85 to 0.96) | 6,156.00 (4,156.21 to 8,847.95) | 0.15 (-0.03 to 0.37) | 0.42 (0.58 to 0.29) | 1,897.36 (1,283.88 to 2,683.25) | 0.11 (-0.01 to 0.25) |
| Male | 2.55 (2.19 to 2.96) | 12,133.65 (10,168.22 to 14,550.46) | 0.06 (-0.01 to 0.14) | 0.90 (1.13 to 0.68) | 4,313.15 (3,064.45 to 5,858.01) | 0.12 (0.00 to 0.32) | 0.34 (0.45 to 0.24) | 1,626.83 (1,141.97 to 2,227.34) | 0.07 (-0.02 to 0.20) |
| **Guinea-Bissau** | | | | | | | | | |
| Total | 0.83 (0.71 to 0.98) | 12,396.71 (10,283.05 to 14,973.64) | 0.07 (-0.02 to 0.18) | 0.34 (0.46 to 0.26) | 5,195.44 (3,591.52 to 7,271.55) | 0.12 (-0.03 to 0.32) | 0.12 (0.16 to 0.08) | 1,735.69 (1,189.21 to 2,420.85) | 0.08 (-0.02 to 0.21) |
| Female | 0.43 (0.36 to 0.51) | 12,698.34 (10,283.26 to 15,815.07) | 0.09 (-0.02 to 0.22) | 0.20 (0.28 to 0.15) | 6,114.52 (4,151.74 to 8,793.01) | 0.14 (-0.04 to 0.35) | 0.06 (0.09 to 0.04) | 1,861.33 (1,235.49 to 2,651.06) | 0.10 (-0.02 to 0.25) |
| Male | 0.40 (0.35 to 0.47) | 12,075.97 (10,061.91 to 14,314.05) | 0.05 (-0.02 to 0.15) | 0.14 (0.18 to 0.11) | 4,252.62 (3,027.60 to 5,837.69) | 0.10 (-0.05 to 0.29) | 0.05 (0.07 to 0.04) | 1,606.05 (1,124.43 to 2,209.92) | 0.07 (-0.03 to 0.19) |
| **Guyana** | | | | | | | | | |
| Total | 0.44 (0.37 to 0.51) | 20,697.23 (17,233.72 to 24,688.66) | 0.17 (0.08 to 0.29) | 0.20 (0.28 to 0.15) | 9,431.90 (6,655.18 to 13,281.91) | 0.41 (0.20 to 0.68) | 0.06 (0.08 to 0.04) | 2,614.96 (1,792.41 to 3,674.69) | 0.26 (0.13 to 0.43) |
| Female | 0.22 (0.18 to 0.26) | 20,660.02 (16,758.51 to 25,150.24) | 0.23 (0.11 to 0.38) | 0.13 (0.18 to 0.09) | 11,939.81 (8,220.61 to 17,169.26) | 0.44 (0.18 to 0.74) | 0.03 (0.05 to 0.02) | 3,033.78 (2,013.61 to 4,324.39) | 0.30 (0.14 to 0.48) |
| Male | 0.22 (0.19 to 0.26) | 20,723.38 (17,273.51 to 24,703.95) | 0.11 (0.04 to 0.2) | 0.08 (0.10 to 0.06) | 6,961.62 (4,991.54 to 9,482.25) | 0.38 (0.15 to 0.63) | 0.02 (0.03 to 0.02) | 2,202.06 (1,534.50 to 3,073.76) | 0.21 (0.09 to 0.37) |
| **Haiti** | | | | | | | | | |
| Total | 6.96 (5.86 to 8.30) | 18,595.86 (15,314.55 to 22,287.68) | 0.09 (0.01 to 0.17) | 2.25 (3.02 to 1.69) | 6,014.63 (4,279.43 to 8,372.47) | 0.24 (0.07 to 0.45) | 0.77 (1.06 to 0.54) | 2,071.11 (1,426.84 to 2,860.39) | 0.14 (0.02 to 0.26) |
| Female | 3.35 (2.75 to 4.05) | 17,867.79 (14,389.05 to 22,085.63) | 0.13 (0.01 to 0.24) | 1.43 (1.96 to 1.04) | 7,650.79 (5,328.46 to 10,853.49) | 0.26 (0.07 to 0.5) | 0.44 (0.60 to 0.30) | 2,332.74 (1,559.10 to 3,263.09) | 0.17 (0.03 to 0.32) |
| Male | 3.61 (3.05 to 4.25) | 19,307.17 (16,029.63 to 23,058.51) | 0.05 (0.00 to 0.11) | 0.81 (1.05 to 0.63) | 4,364.15 (3,197.33 to 5,910.92) | 0.20 (0.05 to 0.38) | 0.34 (0.45 to 0.24) | 1,806.49 (1,252.77 to 2,515.38) | 0.11 (-0.01 to 0.22) |
| **Honduras** | | | | | | | | | |
| Total | 5.19 (4.33 to 6.17) | 16,250.33 (13,313.96 to 19,648.93) | 0.17 (0.07 to 0.30) | 1.77 (2.29 to 1.32) | 5,513.52 (3,936.82 to 7,569.94) | 0.37 (0.17 to 0.60) | 0.67 (0.93 to 0.46) | 2,087.40 (1,406.22 to 2,901.60) | 0.23 (0.10 to 0.38) |
| Female | 2.69 (2.19 to 3.26) | 16,757.84 (13,275.17 to 20,820.83) | 0.23 (0.10 to 0.38) | 1.08 (1.43 to 0.79) | 6,707.95 (4,646.53 to 9,434.16) | 0.41 (0.18 to 0.67) | 0.38 (0.53 to 0.25) | 2,322.97 (1,520.01 to 3,286.04) | 0.27 (0.12 to 0.45) |
| Male | 2.50 (2.12 to 2.96) | 15,702.62 (13,042.58 to 18,770.83) | 0.12 (0.04 to 0.22) | 0.68 (0.87 to 0.53) | 4,293.71 (3,137.54 to 5,751.13) | 0.31 (0.13 to 0.52) | 0.29 (0.41 to 0.21) | 1,845.31 (1,274.06 to 2,551.79) | 0.18 (0.06 to 0.32) |
| **Hungary** | | | | | | | | | |
| Total | 1.94 (1.65 to 2.27) | 13,280.13 (10,992.96 to 15,920.04) | 0.15 (0.06 to 0.26) | 0.68 (0.87 to 0.52) | 4,633.37 (3,346.98 to 6,152.36) | 0.31 (0.14 to 0.52) | 0.26 (0.35 to 0.18) | 1,769.96 (1,226.49 to 2,406.11) | 0.19 (0.07 to 0.33) |
| Female | 0.97 (0.80 to 1.17) | 13,614.43 (10,900.46 to 16,816.76) | 0.20 (0.08 to 0.34) | 0.38 (0.50 to 0.28) | 5,372.94 (3,765.40 to 7,320.92) | 0.35 (0.15 to 0.57) | 0.14 (0.19 to 0.10) | 1,929.67 (1,293.77 to 2,701.69) | 0.23 (0.09 to 0.40) |
| Male | 0.97 (0.83 to 1.13) | 12,965.06 (10,920.82 to 15,310.51) | 0.11 (0.04 to 0.20) | 0.30 (0.37 to 0.23) | 3,934.69 (2,891.66 to 5,158.95) | 0.27 (0.11 to 0.47) | 0.12 (0.16 to 0.09) | 1,619.12 (1,135.01 to 2,192.34) | 0.15 (0.04 to 0.28) |
| **Iceland** | | | | | | | | | |
| Total | 0.13 (0.11 to 0.15) | 18,662.05 (15,272.82 to 22,574.61) | 0.06 (-0.03 to 0.15) | 0.04 (0.06 to 0.03) | 6,500.65 (4,673.66 to 8,717.13) | 0.12 (-0.02 to 0.30) | 0.02 (0.02 to 0.01) | 2,552.94 (1,756.57 to 3,546.82) | 0.07 (-0.03 to 0.17) |
| Female | 0.07 (0.05 to 0.08) | 19,950.89 (15,838.71 to 24,626.75) | 0.07 (-0.04 to 0.17) | 0.03 (0.04 to 0.02) | 8,458.49 (5,925.54 to 11,661.73) | 0.12 (-0.03 to 0.33) | 0.01 (0.01 to 0.01) | 2,977.49 (1,978.67 to 4,209.25) | 0.08 (-0.03 to 0.20) |
| Male | 0.06 (0.05 to 0.07) | 17,419.71 (14,443.09 to 20,809.74) | 0.04 (-0.04 to 0.12) | 0.02 (0.02 to 0.01) | 4,624.16 (3,410.67 to 5,959.57) | 0.11 (-0.02 to 0.26) | 0.01 (0.01 to 0.01) | 2,145.56 (1,511.38 to 2,912.81) | 0.05 (-0.04 to 0.15) |
| **India** | | | | | | | | | |
| Total | 562.55 (493.29 to 640.50) | 14,083.81 (12,105.42 to 16,262.33) | 0.09 (0.07 to 0.12) | 197.37 (243.42 to 160.21) | 4,896.00 (3,729.02 to 6,334.60) | 0.29 (0.24 to 0.35) | 66.61 (86.84 to 48.83) | 1,656.27 (1,180.92 to 2,204.45) | 0.15 (0.12 to 0.19) |
| Female | 281.43 (245.81 to 323.62) | 14,640.01 (12,536.10 to 16,999.58) | 0.12 (0.09 to 0.15) | 105.50 (131.78 to 84.90) | 5,434.54 (4,111.08 to 7,064.65) | 0.33 (0.27 to 0.39) | 33.67 (44.01 to 24.21) | 1,738.77 (1,231.44 to 2,323.78) | 0.18 (0.15 to 0.22) |
| Male | 281.12 (245.53 to 317.85) | 13,564.40 (11,639.89 to 15,611.34) | 0.07 (0.05 to 0.09) | 91.88 (112.23 to 74.27) | 4,396.29 (3,344.76 to 5,622.73) | 0.25 (0.20 to 0.31) | 32.94 (42.73 to 24.05) | 1,579.40 (1,134.57 to 2,097.93) | 0.12 (0.09 to 0.16) |
| **Indonesia** | | | | | | | | | |
| Total | 85.87 (76.48 to 96.59) | 12,389.68 (10,710.51 to 14,248.59) | 0.15 (0.11 to 0.18) | 30.57 (37.24 to 24.89) | 4,396.88 (3,350.08 to 5,612.24) | 0.31 (0.25 to 0.37) | 11.23 (14.89 to 8.22) | 1,610.18 (1,143.41 to 2,169.62) | 0.19 (0.15 to 0.24) |
| Female | 43.13 (37.72 to 48.74) | 12,786.49 (10,889.16 to 14,852.36) | 0.18 (0.14 to 0.23) | 16.28 (20.12 to 13.09) | 4,818.18 (3,648.16 to 6,222.48) | 0.34 (0.27 to 0.40) | 5.87 (7.89 to 4.23) | 1,730.46 (1,212.97 to 2,349.10) | 0.22 (0.17 to 0.27) |
| Male | 42.74 (38.07 to 47.73) | 12,014.57 (10,439.03 to 13,747.88) | 0.11 (0.08 to 0.14) | 14.29 (17.30 to 11.75) | 3,999.81 (3,041.26 to 5,103.46) | 0.28 (0.22 to 0.34) | 5.37 (7.07 to 3.95) | 1,496.76 (1,067.47 to 1,995.14) | 0.15 (0.11 to 0.20) |
| **Iran (Islamic Republic of)** | | | | | | | | | |
| Total | 43.33 (38.46 to 48.97) | 24,525.75 (21,325.62 to 28,025.81) | 0.12 (0.08 to 0.15) | 17.57 (22.17 to 13.86) | 9,976.77 (7,326.16 to 13,303.42) | 0.23 (0.18 to 0.28) | 5.66 (7.53 to 4.02) | 3,210.88 (2,255.93 to 4,329.80) | 0.16 (0.12 to 0.20) |
| Female | 21.86 (19.34 to 24.45) | 25,398.11 (21,970.58 to 29,306.70) | 0.14 (0.10 to 0.18) | 10.57 (13.49 to 8.30) | 12,310.37 (8,983.41 to 16,450.58) | 0.25 (0.19 to 0.32) | 3.15 (4.23 to 2.21) | 3,673.22 (2,547.15 to 4,993.04) | 0.18 (0.14 to 0.22) |
| Male | 21.47 (18.99 to 24.43) | 23,690.03 (20,607.68 to 27,321.33) | 0.09 (0.04 to 0.14) | 7.00 (8.75 to 5.59) | 7,751.34 (5,743.43 to 10,238.11) | 0.19 (0.15 to 0.24) | 2.50 (3.31 to 1.82) | 2,769.64 (1,957.88 to 3,723.11) | 0.14 (0.10 to 0.18) |
| **Iraq** | | | | | | | | | |
| Total | 22.43 (18.7 to 26.67) | 17,923.68 (14,672.37 to 21,789.23) | 0.08 (-0.03 to 0.19) | 8.85 (11.63 to 6.55) | 7,082.00 (5,004.60 to 9,814.89) | 0.12 (-0.04 to 0.31) | 3.07 (4.15 to 2.13) | 2,453.07 (1,669.56 to 3,399.94) | 0.10 (-0.02 to 0.22) |
| Female | 11.76 (9.49 to 14.52) | 19,446.25 (15,337.10 to 24,584.90) | 0.10 (-0.03 to 0.24) | 5.03 (6.73 to 3.61) | 8,318.21 (5,777.56 to 11,656.68) | 0.13 (-0.05 to 0.34) | 1.70 (2.33 to 1.16) | 2,810.08 (1,867.94 to 3,934.85) | 0.11 (-0.03 to 0.26) |
| Male | 10.67 (9.12 to 12.41) | 16,503.26 (13,821.43 to 19,619.04) | 0.06 (-0.02 to 0.17) | 3.83 (5.02 to 2.85) | 5,925.61 (4,197.48 to 8,130.36) | 0.11 (-0.06 to 0.33) | 1.37 (1.85 to 0.96) | 2,119.42 (1,447.43 to 2,912.50) | 0.08 (-0.03 to 0.21) |
| **Ireland** | | | | | | | | | |
| Total | 2.32 (1.94 to 2.79) | 23,619.31 (19,252.13 to 28,927.91) | 0.13 (-0.01 to 0.27) | 1.06 (1.41 to 0.78) | 10,773.43 (7,638.39 to 15,057.12) | 0.30 (0.09 to 0.56) | 0.34 (0.47 to 0.23) | 3,424.34 (2,358.10 to 4,854.67) | 0.17 (0.02 to 0.33) |
| Female | 1.25 (1.03 to 1.54) | 25,908.74 (20,525.53 to 32,670.87) | 0.15 (-0.01 to 0.31) | 0.65 (0.89 to 0.47) | 13,587.46 (9,483.08 to 19,228.56) | 0.31 (0.10 to 0.6) | 0.19 (0.27 to 0.13) | 4,034.44 (2,691.55 to 5,847.10) | 0.19 (0.02 to 0.36) |
| Male | 1.07 (0.91 to 1.26) | 21,408.33 (17,784.24 to 25,810.93) | 0.10 (-0.01 to 0.23) | 0.40 (0.53 to 0.30) | 8,061.01 (5,791.17 to 11,059.60) | 0.29 (0.07 to 0.55) | 0.14 (0.19 to 0.10) | 2,835.97 (1,972.97 to 3,970.31) | 0.15 (0.00 to 0.31) |
| **Israel** | | | | | | | | | |
| Total | 4.09 (3.48 to 4.82) | 18,154.40 (15,148.63 to 21,704.03) | 0.10 (0.01 to 0.20) | 1.91 (2.61 to 1.41) | 8,499.37 (5,812.09 to 12,049.97) | 0.21 (0.01 to 0.41) | 0.59 (0.81 to 0.41) | 2,612.72 (1,802.59 to 3,671.58) | 0.12 (0.01 to 0.26) |
| Female | 2.08 (1.71 to 2.51) | 18,896.93 (15,280.22 to 23,355.55) | 0.13 (0.01 to 0.25) | 1.18 (1.64 to 0.83) | 10,743.34 (7,071.04 to 15,673.38) | 0.22 (0.01 to 0.45) | 0.33 (0.46 to 0.22) | 2,990.79 (1,987.12 to 4,267.22) | 0.15 (0.01 to 0.30) |
| Male | 2.01 (1.74 to 2.32) | 17,442.09 (14,776.08 to 20,399.60) | 0.07 (0.00 to 0.15) | 0.73 (0.97 to 0.55) | 6,356.78 (4,520.86 to 8,676.90) | 0.19 (0.00 to 0.40) | 0.26 (0.36 to 0.18) | 2,251.53 (1,575.67 to 3,114.26) | 0.10 (-0.01 to 0.21) |
| **Italy** | | | | | | | | | |
| Total | 17.83 (15.92 to 19.99) | 20,427.80 (17,754.72 to 23,336.54) | 0.17 (0.13 to 0.21) | 7.55 (9.31 to 6.00) | 8,625.33 (6,516.48 to 11,092.73) | 0.33 (0.27 to 0.40) | 2.62 (3.48 to 1.88) | 2,985.12 (2,087.53 to 3,981.66) | 0.20 (0.16 to 0.25) |
| Female | 9.76 (8.65 to 11.07) | 23,103.85 (19,896.52 to 26,730.08) | 0.21 (0.17 to 0.25) | 4.80 (6.01 to 3.77) | 11,343.51 (8,460.63 to 14,819.14) | 0.35 (0.28 to 0.42) | 1.56 (2.08 to 1.10) | 3,667.87 (2,526.11 to 4,962.42) | 0.23 (0.18 to 0.28) |
| Male | 8.07 (7.22 to 9.04) | 17,949.58 (15,644.57 to 20,488.12) | 0.12 (0.09 to 0.16) | 2.75 (3.35 to 2.23) | 6,100.01 (4,686.88 to 7,818.55) | 0.29 (0.24 to 0.36) | 1.07 (1.40 to 0.78) | 2,351.39 (1,688.73 to 3,105.14) | 0.17 (0.12 to 0.22) |
| **Jamaica** | | | | | | | | | |
| Total | 1.30 (1.09 to 1.53) | 18,285.64 (14,971.55 to 21,895.99) | 0.12 (0.03 to 0.21) | 0.41 (0.55 to 0.30) | 5,649.47 (3,945.90 to 7,844.82) | 0.32 (0.13 to 0.54) | 0.15 (0.21 to 0.10) | 2,081.67 (1,408.56 to 2,930.14) | 0.19 (0.06 to 0.33) |
| Female | 0.62 (0.51 to 0.75) | 17,473.84 (13,835.11 to 21,518.29) | 0.17 (0.06 to 0.29) | 0.25 (0.35 to 0.18) | 7,028.88 (4,684.01 to 10,180.64) | 0.35 (0.13 to 0.58) | 0.08 (0.12 to 0.06) | 2,334.06 (1,537.01 to 3,365.35) | 0.23 (0.08 to 0.39) |
| Male | 0.69 (0.57 to 0.81) | 19,068.64 (15,686.40 to 22,860.08) | 0.08 (0.02 to 0.14) | 0.16 (0.20 to 0.12) | 4,317.77 (3,168.41 to 5,676.85) | 0.27 (0.10 to 0.46) | 0.07 (0.09 to 0.05) | 1,837.97 (1,279.48 to 2,503.84) | 0.14 (0.03 to 0.28) |
| **Japan** | | | | | | | | | |
| Total | 25.68 (23.01 to 28.49) | 14,534.87 (12,693.06 to 16,408.12) | 0.12 (0.09 to 0.14) | 10.33 (12.46 to 8.50) | 5,755.45 (4,493.71 to 7,198.31) | 0.24 (0.19 to 0.29) | 3.65 (4.74 to 2.71) | 2,039.15 (1,487.22 to 2,675.87) | 0.14 (0.11 to 0.18) |
| Female | 11.66 (10.47 to 12.98) | 13,430.36 (11,686.52 to 15,218.58) | 0.16 (0.13 to 0.20) | 5.72 (6.98 to 4.68) | 6,506.97 (5,091.98 to 8,167.52) | 0.26 (0.20 to 0.32) | 1.81 (2.40 to 1.32) | 2,065.48 (1,479.04 to 2,759.55) | 0.18 (0.14 to 0.22) |
| Male | 14.02 (12.45 to 15.77) | 15,576.85 (13,533.38 to 17,770.45) | 0.08 (0.06 to 0.10) | 4.60 (5.49 to 3.81) | 5,039.15 (3,927.11 to 6,283.72) | 0.22 (0.17 to 0.27) | 1.83 (2.35 to 1.37) | 2,013.29 (1,482.57 to 2,618.66) | 0.11 (0.08 to 0.14) |
| **Jordan** | | | | | | | | | |
| Total | 6.96 (5.77 to 8.17) | 18,336.78 (14,922.63 to 22,201.71) | 0.10 (0.00 to 0.22) | 2.87 (3.82 to 2.13) | 7,565.52 (5,285.24 to 10,579.03) | 0.16 (0.00 to 0.36) | 0.97 (1.33 to 0.66) | 2,555.06 (1,716.25 to 3,597.82) | 0.12 (0.00 to 0.25) |
| Female | 3.54 (2.82 to 4.28) | 19,661.63 (15,452.31 to 24,488.97) | 0.12 (0.00 to 0.26) | 1.71 (2.31 to 1.22) | 9,475.85 (6,386.06 to 13,682.53) | 0.17 (-0.01 to 0.38) | 0.53 (0.72 to 0.36) | 2,935.36 (1,928.44 to 4,185.98) | 0.14 (-0.00 to 0.29) |
| Male | 3.42 (2.9 to 3.99) | 17,187.61 (14,374.32 to 20,426.15) | 0.07 (-0.01 to 0.18) | 1.17 (1.52 to 0.89) | 5,856.60 (4,195.13 to 7,932.15) | 0.14 (-0.01 to 0.32) | 0.44 (0.61 to 0.31) | 2,218.12 (1,519.13 to 3,083.75) | 0.10 (-0.02 to 0.23) |
| **Kazakhstan** | | | | | | | | | |
| Total | 4.90 (4.23 to 5.58) | 12,029.47 (10,104.90 to 14,091.84) | 0.09 (0.02 to 0.18) | 2.20 (2.94 to 1.68) | 5,485.21 (3,947.22 to 7,662.94) | 0.21 (0.02 to 0.42) | 0.67 (0.88 to 0.48) | 1,658.90 (1,159.77 to 2,270.29) | 0.12 (0.01 to 0.24) |
| Female | 2.34 (1.98 to 2.75) | 11,718.89 (9,665.60 to 14,090.30) | 0.12 (0.02 to 0.23) | 1.20 (1.65 to 0.89) | 6,076.61 (4,260.22 to 8,665.20) | 0.23 (0.01 to 0.47) | 0.34 (0.46 to 0.24) | 1,728.41 (1,181.28 to 2,416.85) | 0.14 (0.00 to 0.29) |
| Male | 2.56 (2.23 to 2.91) | 12,323.08 (10,421.30 to 14,321.46) | 0.07 (0.01 to 0.14) | 1.00 (1.32 to 0.77) | 4,908.38 (3,556.00 to 6,764.68) | 0.19 (0.00 to 0.38) | 0.33 (0.43 to 0.24) | 1,590.49 (1,127.29 to 2,161.96) | 0.10 (-0.01 to 0.22) |
| **Kenya** | | | | | | | | | |
| Total | 21.70 (19.43 to 24.11) | 12,646.51 (10,998.67 to 14,415.14) | 0.09 (0.07 to 0.11) | 9.72 (11.92 to 7.92) | 5,690.01 (4,375.11 to 7,275.99) | 0.16 (0.13 to 0.19) | 3.10 (4.10 to 2.27) | 1,816.77 (1,299.73 to 2,431.98) | 0.11 (0.08 to 0.13) |
| Female | 10.95 (9.71 to 12.35) | 12,745.28 (10,927.71 to 14,694.03) | 0.11 (0.08 to 0.13) | 5.32 (6.59 to 4.29) | 6,206.62 (4,687.39 to 8,022.03) | 0.17 (0.14 to 0.20) | 1.62 (2.16 to 1.17) | 1,888.21 (1,336.82 to 2,546.75) | 0.12 (0.09 to 0.15) |
| Male | 10.75 (9.59 to 11.93) | 12,515.39 (10,945.54 to 14,268.20) | 0.07 (0.05 to 0.09) | 4.39 (5.37 to 3.62) | 5,159.62 (3,964.68 to 6,590.31) | 0.14 (0.11 to 0.18) | 1.48 (1.95 to 1.09) | 1,740.89 (1,253.79 to 2,314.27) | 0.09 (0.06 to 0.11) |
| **Kiribati** | | | | | | | | | |
| Total | 0.05 (0.04 to 0.06) | 13,703.27 (11,229.44 to 16,582.74) | 0.08 (-0.02 to 0.20) | 0.02 (0.02 to 0.01) | 4,700.14 (3,350.53 to 6,511.23) | 0.15 (-0.02 to 0.37) | 0.01 (0.01 to 0.00) | 1,714.09 (1,183.12 to 2,386.39) | 0.11 (-0.01 to 0.25) |
| Female | 0.02 (0.02 to 0.03) | 13,473.68 (10,619.87 to 16,832.04) | 0.10 (-0.02 to 0.26) | 0.01 (0.01 to 0.01) | 4,790.55 (3,386.21 to 6,615.07) | 0.16 (-0.02 to 0.38) | 0.00 (0.00 to 0.00) | 1,760.96 (1,192.57 to 2,468.64) | 0.13 (-0.01 to 0.31) |
| Male | 0.02 (0.02 to 0.03) | 13,921.02 (11,618.44 to 16,497.55) | 0.06 (-0.02 to 0.15) | 0.01 (0.01 to 0.01) | 4,613.16 (3,279.92 to 6,419.86) | 0.14 (-0.03 to 0.36) | 0.00 (0.00 to 0.00) | 1,666.72 (1,157.31 to 2,306.47) | 0.09 (-0.02 to 0.21) |
| **Kuwait** | | | | | | | | | |
| Total | 1.39 (1.17 to 1.66) | 16,643.79 (13,628.99 to 20,277.31) | 0.03 (-0.06 to 0.15) | 0.59 (0.79 to 0.43) | 7,023.65 (4,844.14 to 9,879.48) | 0.05 (-0.11 to 0.20) | 0.20 (0.28 to 0.14) | 2,393.83 (1,630.35 to 3,348.90) | 0.03 (-0.08 to 0.15) |
| Female | 0.71 (0.59 to 0.86) | 17,457.43 (13,899.85 to 21,892.42) | 0.04 (-0.09 to 0.18) | 0.33 (0.45 to 0.24) | 8,095.86 (5,439.03 to 11,725.10) | 0.04 (-0.12 to 0.23) | 0.11 (0.15 to 0.07) | 2,656.79 (1,753.86 to 3,760.43) | 0.03 (-0.09 to 0.18) |
| Male | 0.68 (0.58 to 0.79) | 15,869.74 (13,277.28 to 18,843.86) | 0.03 (-0.05 to 0.13) | 0.26 (0.34 to 0.19) | 6,007.77 (4,215.31 to 8,335.96) | 0.05 (-0.11 to 0.21) | 0.09 (0.13 to 0.07) | 2,144.37 (1,497.92 to 2,985.96) | 0.03 (-0.07 to 0.14) |
| **Kyrgyzstan** | | | | | | | | | |
| Total | 2.23 (1.94 to 2.55) | 12,619.92 (10,718.86 to 14,785.55) | 0.12 (0.04 to 0.20) | 0.95 (1.25 to 0.7) | 5,415.84 (3,815.77 to 7,491.37) | 0.30 (0.01 to 0.54) | 0.29 (0.40 to 0.21) | 1,669.52 (1,147.20 to 2,291.54) | 0.17 (0.06 to 0.29) |
| Female | 1.07 (0.91 to 1.24) | 12,301.65 (10,281.16 to 14,767.51) | 0.16 (0.06 to 0.27) | 0.52 (0.7 to 0.38) | 6,082.71 (4,233.93 to 8,563.91) | 0.33 (0.11 to 0.60) | 0.15 (0.21 to 0.10) | 1,740.62 (1,174.74 to 2,428.93) | 0.20 (0.07 to 0.36) |
| Male | 1.16 (1.02 to 1.32) | 12,923.44 (11,083.14 to 15,005.13) | 0.09 (0.03 to 0.16) | 0.43 (0.55 to 0.32) | 4,768.26 (3,348.81 to 6,560.45) | 0.27 (0.08 to 0.52) | 0.14 (0.19 to 0.10) | 1,600.15 (1,113.54 to 2,167.24) | 0.13 (0.03 to 0.25) |
| **Lao People's Democratic Republic** | | | | | | | | | |
| Total | 2.84 (2.37 to 3.40) | 13,559.50 (11,015.23 to 16,530.25) | 0.09 (-0.03 to 0.22) | 1.05 (1.39 to 0.78) | 5,017.54 (3,575.97 to 6,956.69) | 0.19 (0.01 to 0.40) | 0.38 (0.50 to 0.27) | 1,795.17 (1,239.14 to 2,459.20) | 0.11 (-0.02 to 0.25) |
| Female | 1.41 (1.15 to 1.73) | 13,578.62 (10,772.67 to 16,959.15) | 0.09 (-0.06 to 0.26) | 0.51 (0.67 to 0.38) | 4,917.58 (3,462.28 to 6,811.40) | 0.19 (0.00 to 0.42) | 0.19 (0.26 to 0.13) | 1,816.21 (1,224.14 to 2,528.34) | 0.11 (-0.05 to 0.31) |
| Male | 1.43 (1.21 to 1.68) | 13,536.90 (11,201.73 to 16,232.18) | 0.08 (-0.01 to 0.19) | 0.54 (0.73 to 0.40) | 5,117.48 (3,620.87 to 7,149.54) | 0.18 (0.00 to 0.40) | 0.19 (0.25 to 0.13) | 1,774.43 (1,227.45 to 2,439.36) | 0.11 (-0.02 to 0.24) |
| **Latvia** | | | | | | | | | |
| Total | 0.42 (0.35 to 0.51) | 15,655.47 (12,730.31 to 19,310.88) | 0.26 (0.13 to 0.42) | 0.17 (0.23 to 0.13) | 6,437.72 (4,497.29 to 8,825.78) | 0.49 (0.25 to 0.76) | 0.06 (0.08 to 0.04) | 2,170.83 (1,494.22 to 3,025.72) | 0.32 (0.16 to 0.50) |
| Female | 0.21 (0.17 to 0.27) | 16,285.50 (12,753.89 to 20,640.24) | 0.32 (0.16 to 0.52) | 0.09 (0.13 to 0.07) | 7,101.23 (4,776.09 to 10,034.03) | 0.51 (0.25 to 0.78) | 0.03 (0.04 to 0.02) | 2,359.74 (1,557.95 to 3,360.34) | 0.36 (0.18 to 0.59) |
| Male | 0.21 (0.18 to 0.25) | 15,060.86 (12,551.69 to 17,953.41) | 0.20 (0.10 to 0.32) | 0.08 (0.11 to 0.06) | 5,810.13 (4,146.37 to 7,916.47) | 0.48 (0.23 to 0.76) | 0.03 (0.04 to 0.02) | 1,992.19 (1,401.11 to 2,745.25) | 0.27 (0.13 to 0.45) |
| **Lebanon** | | | | | | | | | |
| Total | 2.77 (2.27 to 3.36) | 23,351.66 (18,679.13 to 28,767.92) | 0.27 (0.13 to 0.42) | 1.30 (1.73 to 0.94) | 10,971.41 (7,400.04 to 15,651.81) | 0.51 (0.27 to 0.84) | 0.41 (0.57 to 0.28) | 3,453.12 (2,325.73 to 4,894.83) | 0.33 (0.18 to 0.51) |
| Female | 1.44 (1.16 to 1.77) | 25,735.18 (20,029.67 to 32,415.26) | 0.32 (0.16 to 0.49) | 0.73 (0.99 to 0.52) | 13,096.18 (8,636.62 to 19,045.68) | 0.54 (0.27 to 0.85) | 0.22 (0.31 to 0.15) | 3,978.44 (2,612.01 to 5,682.51) | 0.37 (0.19 to 0.56) |
| Male | 1.33 (1.09 to 1.59) | 21,239.14 (17,270.30 to 25,725.21) | 0.22 (0.10 to 0.36) | 0.57 (0.76 to 0.41) | 9,081.64 (6,153.28 to 12,960.28) | 0.46 (0.22 to 0.79) | 0.19 (0.26 to 0.13) | 2,986.66 (2,031.17 to 4,243.49) | 0.28 (0.12 to 0.47) |
| **Lesotho** | | | | | | | | | |
| Total | 0.99 (0.83 to 1.18) | 16,172.24 (13,028.48 to 19,663.85) | 0.27 (0.14 to 0.42) | 0.59 (0.8 to 0.42) | 9,641.73 (6,636.27 to 13,707.53) | 0.50 (0.25 to 0.77) | 0.15 (0.21 to 0.11) | 2,489.32 (1,689.28 to 3,447.93) | 0.33 (0.17 to 0.51) |
| Female | 0.53 (0.43 to 0.64) | 17,135.98 (13,470.82 to 21,283.45) | 0.32 (0.17 to 0.50) | 0.35 (0.47 to 0.24) | 11,274.77 (7,553.07 to 16,355.43) | 0.52 (0.25 to 0.87) | 0.08 (0.11 to 0.06) | 2,732.50 (1,805.29 to 3,879.57) | 0.37 (0.18 to 0.60) |
| Male | 0.47 (0.39 to 0.54) | 15,201.81 (12,490.76 to 18,175.52) | 0.22 (0.11 to 0.34) | 0.25 (0.33 to 0.18) | 8,007.73 (5,564.49 to 11,196.07) | 0.46 (0.21 to 0.77) | 0.07 (0.09 to 0.05) | 2,245.49 (1,538.73 to 3,076.75) | 0.28 (0.13 to 0.44) |
| **Liberia** | | | | | | | | | |
| Total | 2.26 (1.90 to 2.62) | 12,608.76 (10,474.35 to 15,101.31) | 0.05 (-0.03 to 0.15) | 0.93 (1.24 to 0.67) | 5,223.09 (3,550.82 to 7,374.97) | 0.08 (-0.06 to 0.26) | 0.31 (0.42 to 0.22) | 1,733.78 (1,196.80 to 2,381.52) | 0.06 (-0.03 to 0.18) |
| Female | 1.18 (0.99 to 1.41) | 13,302.20 (10,786.64 to 16,417.65) | 0.06 (-0.04 to 0.18) | 0.60 (0.83 to 0.42) | 6,817.42 (4,416.76 to 10,002.97) | 0.09 (-0.08 to 0.30) | 0.17 (0.24 to 0.12) | 1,936.32 (1,300.93 to 2,756.84) | 0.07 (-0.04 to 0.22) |
| Male | 1.07 (0.93 to 1.24) | 11,902.26 (10,082.67 to 14,048.49) | 0.03 (-0.03 to 0.12) | 0.33 (0.41 to 0.25) | 3,620.83 (2,604.12 to 4,787.41) | 0.07 (-0.04 to 0.22) | 0.14 (0.18 to 0.10) | 1,529.39 (1,080.51 to 2,059.38) | 0.05 (-0.05 to 0.15) |
| **Libya** | | | | | | | | | |
| Total | 3.38 (2.81 to 4.05) | 19,257.88 (15,737.64 to 23,659.44) | 0.12 (0.01 to 0.25) | 1.44 (1.96 to 1.07) | 8,169.73 (5,788.62 to 11,538.21) | 0.17 (0.00 to 0.38) | 0.47 (0.65 to 0.32) | 2,685.66 (1,809.39 to 3,773.12) | 0.14 (0.01 to 0.28) |
| Female | 1.75 (1.42 to 2.17) | 20,287.88 (16,040.36 to 25,658.51) | 0.14 (0.01 to 0.29) | 0.81 (1.12 to 0.58) | 9,379.12 (6,412.71 to 13,599.56) | 0.18 (0.00 to 0.42) | 0.26 (0.35 to 0.17) | 2,983.32 (1,960.03 to 4,258.33) | 0.15 (0.01 to 0.31) |
| Male | 1.62 (1.39 to 1.91) | 18,257.55 (15,286.54 to 21,958.05) | 0.09 (0.00 to 0.20) | 0.63 (0.84 to 0.47) | 7,004.39 (4,985.25 to 9,781.97) | 0.16 (-0.01 to 0.36) | 0.21 (0.30 to 0.15) | 2,398.17 (1,637.36 to 3,337.13) | 0.12 (-0.01 to 0.27) |
| **Lithuania** | | | | | | | | | |
| Total | 0.70 (0.59 to 0.85) | 16,821.52 (13,828.71 to 20,652.52) | 0.27 (0.15 to 0.41) | 0.31 (0.42 to 0.24) | 7,441.98 (5,333.85 to 10,309.21) | 0.54 (0.29 to 0.81) | 0.10 (0.14 to 0.07) | 2,390.24 (1,627.86 to 3,318.67) | 0.34 (0.19 to 0.50) |
| Female | 0.36 (0.29 to 0.44) | 17,725.21 (13,954.40 to 22,206.53) | 0.34 (0.18 to 0.50) | 0.17 (0.22 to 0.12) | 8,144.46 (5,748.61 to 11,354.44) | 0.57 (0.33 to 0.87) | 0.05 (0.07 to 0.04) | 2,613.63 (1,742.51 to 3,670.66) | 0.39 (0.21 to 0.58) |
| Male | 0.34 (0.29 to 0.40) | 15,966.16 (13,469.87 to 19,202.81) | 0.21 (0.11 to 0.33) | 0.15 (0.19 to 0.11) | 6,775.41 (4,858.66 to 9,340.79) | 0.50 (0.25 to 0.81) | 0.05 (0.06 to 0.03) | 2,178.43 (1,517.94 to 3,021.47) | 0.29 (0.15 to 0.45) |
| **Luxembourg** | | | | | | | | | |
| Total | 0.21 (0.18 to 0.25) | 19,584.36 (16,202.55 to 23,551.73) | 0.11 (0.01 to 0.23) | 0.08 (0.10 to 0.06) | 7,545.85 (5,389.98 to 10,072.76) | 0.23 (0.06 to 0.45) | 0.03 (0.04 to 0.02) | 2,791.51 (1,917.68 to 3,865.91) | 0.14 (0.02 to 0.28) |
| Female | 0.11 (0.09 to 0.14) | 21,543.17 (17,264.95 to 26,758.69) | 0.14 (0.02 to 0.28) | 0.05 (0.07 to 0.04) | 9,655.52 (6,759.10 to 13,199.57) | 0.24 (0.05 to 0.47) | 0.02 (0.02 to 0.01) | 3,297.02 (2,211.57 to 4,634.11) | 0.15 (0.02 to 0.31) |
| Male | 0.10 (0.08 to 0.11) | 17,750.79 (14,897.72 to 21,132.65) | 0.09 (0.00 to 0.19) | 0.03 (0.04 to 0.02) | 5,575.02 (4,063.92 to 7,296.53) | 0.21 (0.03 to 0.39) | 0.01 (0.02 to 0.01) | 2,318.90 (1,608.28 to 3,191.53) | 0.11 (-0.01 to 0.26) |
| **Madagascar** | | | | | | | | | |
| Total | 12.88 (10.9 to 15.00) | 13,504.07 (11,152.06 to 16,198.38) | 0.10 (0.00 to 0.22) | 5.83 (8.00 to 4.26) | 6,138.62 (4,244.25 to 8,606.75) | 0.20 (0.01 to 0.41) | 1.83 (2.48 to 1.28) | 1,930.47 (1,312.20 to 2,658.63) | 0.13 (0.01to 0.26) |
| Female | 6.64 (5.51 to 7.91) | 13,734.15 (11,076.16 to 16,798.57) | 0.12 (0.01 to 0.26) | 3.27 (4.54 to 2.33) | 6,777.81 (4,554.19 to 9,696.05) | 0.21 (0.01 to 0.44) | 0.97 (1.34 to 0.67) | 2,021.98 (1,360.12 to 2,833.82) | 0.14 (0.01 to 0.28) |
| Male | 6.24 (5.34 to 7.23) | 13,255.82 (11,022.09 to 15,742.42) | 0.08 (0.00 to 0.18) | 2.56 (3.42 to 1.90) | 5,475.33 (3,835.46 to 7,601.23) | 0.19 (0.01 to 0.42) | 0.86 (1.15 to 0.60) | 1,834.65 (1,258.72 to 2,503.31) | 0.11 (0.00 to 0.24) |
| **Malawi** | | | | | | | | | |
| Total | 9.42 (7.98 to 11.12) | 13,349.92 (10,960.02 to 16,094.81) | 0.12 (0.02 to 0.23) | 3.93 (5.24 to 2.90) | 5,589.47 (3,916.04 to 7,770.24) | 0.24 (0.06 to 0.45) | 1.31 (1.78 to 0.92) | 1,866.92 (1,295.17 to 2,578.64) | 0.15 (0.04 to 0.27) |
| Female | 4.90 (4.09 to 5.84) | 13,624.82 (10,957.72 to 16,628.33) | 0.15 (0.03 to 0.28) | 2.26 (3.02 to 1.63) | 6,285.33 (4,301.51 to 8,906.98) | 0.27 (0.07 to 0.49) | 0.70 (0.97 to 0.49) | 1,966.74 (1,329.98 to 2,781.52) | 0.18 (0.04 to 0.32) |
| Male | 4.53 (3.9 to 5.25) | 13,045.10 (10,920.96 to 15,407.26) | 0.10 (0.01 to 0.18) | 1.67 (2.19 to 1.26) | 4,856.16 (3,465.14 to 6,613.96) | 0.22 (0.03 to 0.42) | 0.61 (0.81 to 0.43) | 1,760.36 (1,215.70 to 2,390.44) | 0.12 (0.02 to 0.24) |
| **Malaysia** | | | | | | | | | |
| Total | 10.61 (8.79 to 13.02) | 13,017.34 (10,522.78 to 16,228.75) | 0.05 (-0.07 to 0.20) | 4.67 (6.22 to 3.46) | 5,721.51 (4,054.34 to 8,033.82) | -0.09 (-0.28 to 0.12) | 1.57 (2.15 to 1.08) | 1,915.68 (1,288.54 to 2,707.16) | 0.02 (-0.13 to 0.17) |
| Female | 5.50 (4.45 to 6.96) | 13,870.56 (10,878.59 to 17,873.99) | 0.07 (-0.08 to 0.25) | 2.39 (3.30 to 1.73) | 6,037.78 (4,189.52 to 8,583.20) | -0.11 (-0.33 to 0.11) | 0.81 (1.12 to 0.55) | 2,039.40 (1,343.78 to 2,909.57) | 0.02 (-0.14 to 0.19) |
| Male | 5.11 (4.35 to 6.06) | 12,213.35 (10,149.65 to 14,864.08) | 0.04 (-0.08 to 0.16) | 2.28 (3.01 to 1.68) | 5,422.50 (3,816.30 to 7,550.73) | -0.07 (-0.27 to 0.14) | 0.76 (1.04 to 0.53) | 1,798.97 (1,229.86 to 2,524.36) | 0.01 (-0.14 to 0.16) |
| **Maldives** | | | | | | | | | |
| Total | 0.12 (0.11 to 0.15) | 12,431.64 (10,236.88 to 15,116.48) | 0.11 (0.02 to 0.22) | 0.05 (0.07 to 0.04) | 5,161.95 (3,691.80 to 7,202.50) | 0.23 (0.05 to 0.47) | 0.02 (0.02 to 0.01) | 1,707.12 (1,174.56 to 2,335.34) | 0.13 (0.03 to 0.27) |
| Female | 0.06 (0.05 to 0.07) | 12,853.89 (10,262.13 to 16,297.86) | 0.14 (0.01 to 0.28) | 0.03 (0.04 to 0.02) | 5,789.30 (4,000.55 to 8,222.66) | 0.25 (0.05 to 0.49) | 0.01 (0.01 to 0.01) | 1,829.56 (1,221.95 to 2,567.68) | 0.16 (0.01 to 0.32) |
| Male | 0.07 (0.06 to 0.08) | 12,146.06 (10,178.14 to 14,410.33) | 0.09 (0.01 to 0.18) | 0.03 (0.03 to 0.02) | 4,654.80 (3,358.64 to 6,325.28) | 0.21 (0.03 to 0.46) | 0.01 (0.01 to 0.01) | 1,611.72 (1,132.90 to 2,210.05) | 0.11 (0.01 to 0.25) |
| **Mali** | | | | | | | | | |
| Total | 9.13 (7.67 to 10.6) | 11,484.63 (9,441.96 to 13,799.22) | 0.05 (-0.03 to 0.15) | 3.37 (4.46 to 2.54) | 4,263.13 (3,008.05 to 5,874.17) | 0.09 (-0.05 to 0.26) | 1.24 (1.68 to 0.88) | 1,571.62 (1,095.07 to 2,167.26) | 0.06 (-0.03 to 0.17) |
| Female | 4.60 (3.77 to 5.52) | 11,567.57 (9,250.89 to 14,312.74) | 0.07 (-0.03 to 0.19) | 1.93 (2.57 to 1.41) | 4,872.93 (3,347.55 to 6,887.44) | 0.10 (-0.06 to 0.29) | 0.65 (0.90 to 0.45) | 1,650.63 (1,122.14 to 2,323.66) | 0.08 (-0.04 to 0.20) |
| Male | 4.53 (3.88 to 5.22) | 11,386.02 (9,487.79 to 13,461.64) | 0.04 (-0.02 to 0.12) | 1.44 (1.85 to 1.12) | 3,638.12 (2,647.61 to 4,928.05) | 0.08 (-0.04 to 0.23) | 0.59 (0.79 to 0.42) | 1,489.71 (1,057.83 to 2,022.81) | 0.05 (-0.04 to 0.15) |
| **Malta** | | | | | | | | | |
| Total | 0.13 (0.11 to 0.15) | 19,800.40 (16,356.21 to 24,269.83) | 0.10 (-0.01 to 0.23) | 0.05 (0.06 to 0.04) | 7,344.89 (5,278.95 to 10,180.43) | 0.19 (0.02 to 0.38) | 0.02 (0.02 to 0.01) | 2,782.58 (1,887.85 to 3,881.26) | 0.11 (0.00 to 0.26) |
| Female | 0.07 (0.06 to 0.08) | 21,836.36 (17,464.52 to 27,547.46) | 0.12 (-0.01 to 0.27) | 0.03 (0.04 to 0.02) | 9,605.96 (6,742.47 to 13,629.78) | 0.19 (0.02 to 0.41) | 0.01 (0.01 to 0.01) | 3,291.84 (2,169.80 to 4,702.84) | 0.13 (0.00 to 0.29) |
| Male | 0.06 (0.05 to 0.07) | 17,866.04 (15,028.52 to 21,427.93) | 0.08 (-0.01 to 0.19) | 0.02 (0.02 to 0.01) | 5,205.80 (3,833.02 to 6,871.97) | 0.17 (0.02 to 0.36) | 0.01 (0.01 to 0.01) | 2,300.27 (1,595.38 to 3,110.84) | 0.10 (-0.02 to 0.24) |
| **Marshall Islands** | | | | | | | | | |
| Total | 0.02 (0.02 to 0.03) | 13,429.44 (11,022.19 to 16,186.73) | 0.08 (-0.01 to 0.19) | 0.01 (0.01 to 0.01) | 4,628.97 (3,321.36 to 6,435.86) | 0.16 (-0.01 to 0.36) | 0.00 (0.00 to 0.00) | 1,698.12 (1,167.56 to 2,358.59) | 0.11 (-0.02 to 0.24) |
| Female | 0.01 (0.01 to 0.01) | 13,254.00 (10,548.89 to 16,425.27) | 0.11 (-0.02 to 0.26) | 0.00 (0.01 to 0.00) | 4,808.77 (3,372.60 to 6,632.24) | 0.17 (-0.02 to 0.39) | 0.00 (0.00 to 0.00) | 1,754.78 (1,178.87 to 2,506.70) | 0.12 (-0.01 to 0.29) |
| Male | 0.01 (0.01 to 0.01) | 13,599.94 (11,347.65 to 16,108.93) | 0.06 (-0.01 to 0.15) | 0.00 (0.00 to 0.00) | 4,454.28 (3,182.03 to 6,186.09) | 0.15 (-0.02 to 0.35) | 0.00 (0.00 to 0.00) | 1,643.15 (1,141.69 to 2,258.90) | 0.08 (-0.02 to 0.22) |
| **Mauritania** | | | | | | | | | |
| Total | 1.65 (1.39 to 1.95) | 11,513.02 (9,519.24 to 13,887.11) | 0.06 (-0.02 to 0.16) | 0.63 (0.85 to 0.48) | 4,462.50 (3,079.97 to 6,295.83) | 0.12 (-0.03 to 0.31) | 0.23 (0.31 to 0.16) | 1,616.98 (1,118.73 to 2,217.31) | 0.07 (-0.03 to 0.20) |
| Female | 0.83 (0.69 to 1.02) | 11,435.33 (9,285.06 to 14,253.44) | 0.08 (-0.03 to 0.20) | 0.34 (0.47 to 0.25) | 4,753.97 (3,225.71 to 6,772.76) | 0.13 (-0.04 to 0.34) | 0.12 (0.16 to 0.08) | 1,656.17 (1,121.22 to 2,327.79) | 0.09 (-0.04 to 0.23) |
| Male | 0.81 (0.71 to 0.94) | 11,579.84 (9,717.96 to 13,754.66) | 0.05 (-0.02 to 0.13) | 0.29 (0.38 to 0.22) | 4,151.78 (2,934.34 to 5,725.74) | 0.10 (-0.04 to 0.28) | 0.11 (0.14 to 0.08) | 1,574.08 (1,102.76 to 2,163.28) | 0.06 (-0.03 to 0.18) |
| **Mauritius** | | | | | | | | | |
| Total | 0.37 (0.31 to 0.44) | 14,079.65 (11,649.42 to 17,037.50) | 0.16 (0.06 to 0.28) | 0.20 (0.28 to 0.15) | 7,651.24 (5,268.17 to 10,865.24) | 0.32 (0.09 to 0.60) | 0.06 (0.08 to 0.04) | 2,076.70 (1,400.19 to 2,899.64) | 0.21 (0.07 to 0.36) |
| Female | 0.20 (0.16 to 0.24) | 15,073.88 (12,053.69 to 18,713.13) | 0.20 (0.07 to 0.35) | 0.12 (0.17 to 0.09) | 9,273.01 (6,258.54 to 13,466.32) | 0.34 (0.08 to 0.66) | 0.03 (0.04 to 0.02) | 2,341.81 (1,536.54 to 3,357.49) | 0.24 (0.07 to 0.43) |
| Male | 0.17 (0.15 to 0.2) | 13,101.83 (11,080.03 to 15,507.49) | 0.12 (0.04 to 0.22) | 0.08 (0.11 to 0.06) | 6,058.02 (4,227.20 to 8,471.19) | 0.29 (0.07 to 0.57) | 0.02 (0.03 to 0.02) | 1,816.16 (1,246.06 to 2,490.60) | 0.17 (0.03 to 0.33) |
| **Mexico** | | | | | | | | | |
| Total | 49.72 (44.68 to 55.37) | 14,910.52 (12,984.34 to 16,972.17) | 0.16 (0.13 to 0.19) | 21.76 (26.56 to 17.84) | 6,483.92 (5,034.41 to 8,315.26) | 0.32 (0.27 to 0.38) | 7.08 (9.39 to 5.12) | 2,113.44 (1,497.00 to 2,836.34) | 0.20 (0.16 to 0.24) |
| Female | 26.51 (23.51 to 29.9) | 15,912.52 (13,668.49 to 18,331.47) | 0.21 (0.17 to 0.25) | 13.93 (17.62 to 11.14) | 8,306.14 (6,393.15 to 10,736.61) | 0.36 (0.30 to 0.41) | 4.10 (5.46 to 2.94) | 2,446.52 (1,709.42 to 3,313.15) | 0.24 (0.19 to 0.28) |
| Male | 23.21 (20.65 to 25.83) | 13,898.95 (12,136.83 to 15,759.99) | 0.11 (0.09 to 0.14) | 7.83 (9.34 to 6.52) | 4,667.45 (3,647.16 to 5,865.24) | 0.27 (0.22 to 0.33) | 2.99 (3.93 to 2.20) | 1,780.65 (1,281.24 to 2,361.29) | 0.15 (0.11 to 0.19) |
| **Micronesia (Federated States of)** | | | | | | | | | |
| Total | 0.04 (0.04 to 0.05) | 13,442.39 (11,067.40 to 16,216.39) | 0.08 (-0.02 to 0.19) | 0.01 (0.02 to 0.01) | 4,591.63 (3,304.18 to 6,242.22) | 0.15 (-0.03 to 0.34) | 0.01 (0.01 to 0) | 1,698.95 (1,174.16 to 2,348.10) | 0.11 (-0.01 to 0.25) |
| Female | 0.02 (0.02 to 0.02) | 13,252.90 (10,556.84 to 16,561.75) | 0.10 (-0.02 to 0.25) | 0.01 (0.01 to 0.01) | 4,757.50 (3,386.21 to 6,549.03) | 0.16 (-0.03 to 0.37) | 0.00 (0.00 to 0.00) | 1,756.98 (1,174.90 to 2,467.85) | 0.12 (-0.02 to 0.29) |
| Male | 0.02 (0.02 to 0.03) | 13,620.61 (11,376.39 to 16,228.55) | 0.06 (-0.02 to 0.15) | 0.01 (0.01 to 0.01) | 4,435.28 (3,196.77 to 6,101.45) | 0.14 (-0.03 to 0.36) | 0.00 (0.00 to 0.00) | 1,644.20 (1,149.72 to 2,274.05) | 0.09 (-0.02 to 0.22) |
| **Monaco** | | | | | | | | | |
| Total | 0.01 (0.01 to 0.01) | 21,310.06 (17,228.93 to 26,092.72) | 0.15 (0.03 to 0.27) | 0.01 (0.01 to 0.00) | 9,632.95 (6,507.30 to 13,781.80) | 0.30 (0.11 to 0.51) | 0.00 (0.00 to 0.00) | 3,199.70 (2,132.19 to 4,447.30) | 0.18 (0.04 to 0.31) |
| Female | 0.01 (0.01 to 0.01) | 23,859.31 (18,742.70 to 29,522.23) | 0.18 (0.04 to 0.32) | 0.00 (0.00 to 0.00) | 12,332.69 (8,275.13 to 17,836.19) | 0.31 (0.12 to 0.54) | 0.00 (0.00 to 0.00) | 3,839.93 (2,504.19 to 5,371.19) | 0.20 (0.05 to 0.36) |
| Male | 0.01 (0.00 to 0.01) | 18,885.54 (15,644.46 to 22,681.40) | 0.12 (0.01 to 0.23) | 0.00 (0.00 to 0.00) | 7,060.33 (4,856.59 to 9,977.29) | 0.27 (0.08 to 0.49) | 0.00 (0.00 to 0.00) | 2,590.04 (1,746.57 to 3,596.30) | 0.15 (0.02 to 0.28) |
| **Mongolia** | | | | | | | | | |
| Total | 0.89 (0.78 to 1.03) | 11,846.86 (9,951.12 to 13,915.61) | 0.03 (-0.03 to 0.11) | 0.4 (0.53 to 0.31) | 5,379.97 (3,828.62 to 7,394.96) | 0.05 (-0.11 to 0.24) | 0.12 (0.16 to 0.09) | 1,602.03 (1,109.78 to 2,225.77) | 0.03 (-0.06 to 0.14) |
| Female | 0.42 (0.36 to 0.49) | 11,278.07 (9,298.31 to 13,554.66) | 0.04 (-0.04 to 0.15) | 0.21 (0.28 to 0.16) | 5,669.14 (3,992.19 to 7,934.92) | 0.05 (-0.12 to 0.27) | 0.06 (0.08 to 0.04) | 1,619.59 (1,100.03 to 2,266.68) | 0.04 (-0.08 to 0.17) |
| Male | 0.48 (0.41 to 0.54) | 12,392.52 (10,474.80 to 14,493.69) | 0.02 (-0.03 to 0.08) | 0.19 (0.25 to 0.15) | 5,096.92 (3,666.30 to 7,090.60) | 0.05 (-0.11 to 0.25) | 0.06 (0.08 to 0.04) | 1,584.24 (1,113.45 to 2,188.26) | 0.03 (-0.07 to 0.13) |
| **Montenegro** | | | | | | | | | |
| Total | 0.17 (0.14 to 0.20) | 14,219.18 (11,772.88 to 17,264.44) | 0.21 (0.11 to 0.34) | 0.06 (0.08 to 0.05) | 5,297.71 (3,799.64 to 7,220.07) | 0.44 (0.25 to 0.71) | 0.02 (0.03 to 0.02) | 1,914.29 (1,314.91 to 2,636.84) | 0.27 (0.13 to 0.43) |
| Female | 0.08 (0.07 to 0.10) | 14,882.39 (11,835.39 to 18,582.87) | 0.28 (0.15 to 0.44) | 0.04 (0.05 to 0.03) | 6,449.09 (4,489.44 to 9,085.08) | 0.50 (0.26 to 0.81) | 0.01 (0.02 to 0.01) | 2,135.80 (1,425.33 to 2,994.41) | 0.33 (0.17 to 0.51) |
| Male | 0.08 (0.07 to 0.10) | 13,602.84 (11,417.74 to 16,203.81) | 0.15 (0.07 to 0.25) | 0.03 (0.03 to 0.02) | 4,232.42 (3,126.31 to 5,538.98) | 0.37 (0.2 to 0.61) | 0.01 (0.01 to 0.01) | 1,709.22 (1,199.85 to 2,348.85) | 0.20 (0.08 to 0.35) |
| **Morocco** | | | | | | | | | |
| Total | 18.56 (15.48 to 22.10) | 19,822.90 (16,115.80 to 24,050.62) | 0.14 (0.04 to 0.28) | 8.75 (11.85 to 6.17) | 9,328.23 (6,301.60 to 13,288.51) | 0.27 (0.07 to 0.50) | 2.65 (3.65 to 1.88) | 2,820.86 (1,945.23 to 3,968.25) | 0.18 (0.06 to 0.34) |
| Female | 9.67 (7.80 to 11.85) | 21,002.97 (16,677.54 to 26,135.73) | 0.17 (0.04 to 0.34) | 5.03 (6.94 to 3.55) | 10,910.28 (7,235.78 to 15,783.93) | 0.29 (0.06 to 0.55) | 1.46 (2.05 to 1.02) | 3,156.33 (2,141.75 to 4,494.85) | 0.21 (0.06 to 0.38) |
| Male | 8.89 (7.57 to 10.43) | 18,670.84 (15,601.00 to 22,285.79) | 0.11 (0.02 to 0.23) | 3.72 (5.02 to 2.67) | 7,799.28 (5,317.31 to 10,987.87) | 0.25 (0.05 to 0.48) | 1.19 (1.62 to 0.84) | 2,495.57 (1,715.24 to 3,442.26) | 0.16 (0.03 to 0.30) |
| **Mozambique** | | | | | | | | | |
| Total | 14.55 (12.34 to 17.19) | 13,924.04 (11,589.05 to 16,773.86) | 0.13 (0.03 to 0.23) | 6.78 (9.14 to 4.96) | 6,543.62 (4,525.93 to 9,410.52) | 0.25 (0.05 to 0.47) | 2.06 (2.77 to 1.44) | 1,983.76 (1,364.80 to 2,723.43) | 0.16 (0.04 to 0.28) |
| Female | 7.71 (6.42 to 9.28) | 14,164.72 (11,547.92 to 17,448.97) | 0.15 (0.04 to 0.26) | 3.90 (5.35 to 2.83) | 7,182.85 (4,885.24 to 10,539.13) | 0.26 (0.06 to 0.50) | 1.12 (1.52 to 0.77) | 2,058.19 (1,380.11 to 2,871.22) | 0.18 (0.04 to 0.31) |
| Male | 6.84 (5.85 to 7.95) | 13,618.23 (11,446.68 to 16,196.01) | 0.10 (0.01 to 0.19) | 2.89 (3.81 to 2.14) | 5,831.33 (4,046.29 to 8,201.35) | 0.23 (0.04 to 0.46) | 0.94 (1.27 to 0.67) | 1,897.57 (1,332.71 to 2,601.91) | 0.13 (0.02 to 0.26) |
| **Myanmar** | | | | | | | | | |
| Total | 19.51 (16.43 to 23.23) | 12,780.80 (10,505.64 to 15,423.07) | 0.17 (0.05 to 0.30) | 6.31 (8.22 to 4.85) | 4,131.96 (2,991.15 to 5,583.55) | 0.39 (0.19 to 0.63) | 2.50 (3.34 to 1.76) | 1,635.73 (1,116.47 to 2,253.02) | 0.22 (0.08 to 0.39) |
| Female | 10 (8.13 to 12.25) | 13,063.02 (10,368.45 to 16,404.46) | 0.21 (0.06 to 0.39) | 3.22 (4.18 to 2.43) | 4,213.15 (3,029.21 to 5,687.54) | 0.41 (0.20 to 0.65) | 1.31 (1.77 to 0.89) | 1,701.14 (1,122.86 to 2,363.60) | 0.26 (0.09 to 0.46) |
| Male | 9.51 (8.18 to 11.07) | 12,487.04 (10,509.49 to 14,839.44) | 0.13 (0.04 to 0.24) | 3.09 (4 to 2.38) | 4,050.14 (2,919.49 to 5,482.14) | 0.36 (0.18 to 0.61) | 1.20 (1.60 to 0.85) | 1,569.35 (1,087.32 to 2,151.56) | 0.19 (0.06 to 0.35) |
| **Namibia** | | | | | | | | | |
| Total | 1.06 (0.89 to 1.25) | 14,266.12 (11,661.51 to 17,324.67) | 0.25 (0.13 to 0.40) | 0.49 (0.64 to 0.37) | 6,539.29 (4,556.56 to 9,084.03) | 0.48 (0.28 to 0.71) | 0.16 (0.21 to 0.11) | 2,096.27 (1,434.67 to 2,885.46) | 0.30 (0.17 to 0.44) |
| Female | 0.55 (0.45 to 0.66) | 14,610.22 (11,633.49 to 18,206.99) | 0.31 (0.16 to 0.47) | 0.26 (0.35 to 0.19) | 7,005.53 (4,793.43 to 9,941.86) | 0.52 (0.30 to 0.78) | 0.08 (0.11 to 0.06) | 2,199.11 (1,480.72 to 3,067.32) | 0.34 (0.19 to 0.53) |
| Male | 0.51 (0.44 to 0.59) | 13,913.49 (11,609.60 to 16,566.57) | 0.20 (0.11 to 0.33) | 0.22 (0.29 to 0.17) | 6,064.36 (4,325.85 to 8,321.53) | 0.43 (0.23 to 0.64) | 0.07 (0.10 to 0.05) | 1,991.33 (1,361.89 to 2,708.09) | 0.25 (0.12 to 0.38) |
| **Nauru** | | | | | | | | | |
| Total | 0.00 (0.00 to 0.01) | 13,303.90 (10,889.71 to 16,231.70) | 0.09 (-0.02 to 0.20) | 0.00 (0.00 to 0.00) | 4,953.64 (3,378.73 to 7,055.57) | 0.16 (0.00 to 0.36) | 0.00 (0.00 to 0.00) | 1,741.23 (1,169.34 to 2,466.63) | 0.11 (-0.02 to 0.24) |
| Female | 0.00 (0.00 to 0.00) | 13,240.46 (10,518.43 to 16,794.65) | 0.11 (-0.02 to 0.26) | 0.00 (0.00 to 0.00) | 5,274.80 (3,563.23 to 7,584.54) | 0.17 (0.01 to 0.38) | 0.00 (0.00 to 0.00) | 1,825.13 (1,197.48 to 2,640.66) | 0.13 (-0.02 to 0.31) |
| Male | 0.00 (0.00 to 0.00) | 13,355.94 (11,142.90 to 15,970.39) | 0.06 (-0.01 to 0.15) | 0.00 (0.00 to 0.00) | 4,649.81 (3,199.86 to 6,640.07) | 0.15 (-0.02 to 0.34) | 0.00 (0.00 to 0.00) | 1,661.46 (1,134.14 to 2,332.67) | 0.09 (-0.02 to 0.21) |
| **Nepal** | | | | | | | | | |
| Total | 12.83 (10.90 to 15.19) | 13,460.10 (11,185.85 to 16,157.06) | 0.14 (0.04 to 0.26) | 5.97 (8.2 to 4.22) | 6,197.25 (4,169.55 to 8,864.63) | 0.36 (0.12 to 0.62) | 1.70 (2.36 to 1.19) | 1,768.89 (1,206.04 to 2,503.32) | 0.21 (0.07 to 0.37) |
| Female | 6.55 (5.44 to 7.93) | 13,395.33 (10,878.07 to 16,412.30) | 0.18 (0.06 to 0.34) | 3.52 (4.92 to 2.44) | 7,105.45 (4,665.84 to 10,344.13) | 0.40 (0.12 to 0.70) | 0.92 (1.31 to 0.62) | 1,859.42 (1,225.21 to 2,679.21) | 0.25 (0.08 to 0.47) |
| Male | 6.29 (5.39 to 7.27) | 13,483.30 (11,311.52 to 15,969.19) | 0.10 (0.03 to 0.19) | 2.45 (3.36 to 1.78) | 5,231.61 (3,648.14 to 7,409.26) | 0.31 (0.08 to 0.54) | 0.78 (1.06 to 0.56) | 1,669.72 (1,149.10 to 2,334.97) | 0.16 (0.05 to 0.30) |
| **Netherlands** | | | | | | | | | |
| Total | 6.58 (5.50 to 7.77) | 21,791.46 (17,744.32 to 26,483.77) | 0.15 (0.04 to 0.28) | 2.53 (3.33 to 1.86) | 8,353.39 (5,953.25 to 11,407.63) | 0.31 (0.13 to 0.55) | 0.94 (1.27 to 0.66) | 3,094.65 (2,140.24 to 4,292.68) | 0.19 (0.06 to 0.33) |
| Female | 3.57 (2.90 to 4.36) | 24,103.61 (18,944.93 to 30,156.46) | 0.21 (0.06 to 0.37) | 1.56 (2.09 to 1.14) | 10,488.90 (7,267.92 to 14,735.14) | 0.34 (0.13 to 0.60) | 0.54 (0.75 to 0.37) | 3,613.94 (2,427.69 to 5,107.29) | 0.24 (0.09 to 0.40) |
| Male | 3.01 (2.57 to 3.50) | 19,578.07 (16,273.37 to 23,526.35) | 0.10 (-0.01 to 0.21) | 0.97 (1.26 to 0.74) | 6,310.76 (4,581.70 to 8,462.16) | 0.27 (0.08 to 0.50) | 0.40 (0.55 to 0.28) | 2,597.82 (1,781.64 to 3,613.50) | 0.14 (0.01 to 0.28) |
| **New Zealand** | | | | | | | | | |
| Total | 2.35 (2.09 to 2.67) | 23,035.28 (19,955.00 to 26,501.57) | 0.01 (-0.06 to 0.07) | 1.00 (1.29 to 0.79) | 9,794.26 (7,417.75 to 12,949.49) | 0.01 (-0.11 to 0.13) | 0.34 (0.45 to 0.24) | 3,269.81 (2,306.82 to 4,362.54) | 0.01 (-0.07 to 0.09) |
| Female | 1.20 (1.06 to 1.37) | 24,263.75 (20,926.12 to 28,273.85) | 0.01 (-0.07 to 0.08) | 0.62 (0.80 to 0.48) | 12,433.34 (9,279.92 to 16,531.51) | 0.00 (-0.12 to 0.13) | 0.19 (0.25 to 0.13) | 3,757.27 (2,608.75 to 5,090.86) | 0.01 (-0.07 to 0.10) |
| Male | 1.15 (1.01 to 1.31) | 21,911.85 (18,880.77 to 25,358.09) | 0.00 (-0.06 to 0.07) | 0.39 (0.50 to 0.31) | 7,342.95 (5,556.92 to 9,598.10) | 0.01 (-0.10 to 0.14) | 0.15 (0.20 to 0.11) | 2,818.50 (2,000.13 to 3,754.08) | 0.01 (-0.07 to 0.09) |
| **Nicaragua** | | | | | | | | | |
| Total | 3.06 (2.60 to 3.61) | 16,246.73 (13,513.22 to 19,601.23) | 0.13 (0.03 to 0.23) | 1.15 (1.48 to 0.87) | 6,053.92 (4,414.92 to 8,143.49) | 0.25 (0.06 to 0.47) | 0.40 (0.55 to 0.29) | 2,131.15 (1,473.49 to 2,942.25) | 0.16 (0.03 to 0.30) |
| Female | 1.56 (1.29 to 1.91) | 16,832.04 (13,664.04 to 20,899.14) | 0.16 (0.03 to 0.29) | 0.70 (0.93 to 0.51) | 7,503.69 (5,338.83 to 10,383.56) | 0.28 (0.06 to 0.54) | 0.22 (0.31 to 0.16) | 2,400.48 (1,637.89 to 3,389.32) | 0.20 (0.05 to 0.36) |
| Male | 1.51 (1.30 to 1.75) | 15,670.71 (13,172.37 to 18,595.67) | 0.09 (0.01 to 0.17) | 0.45 (0.58 to 0.35) | 4,655.84 (3,481.51 to 6,213.16) | 0.21 (0.02 to 0.40) | 0.18 (0.24 to 0.13) | 1,870.64 (1,315.21 to 2,554.12) | 0.13 (0.01 to 0.25) |
| **Niger** | | | | | | | | | |
| Total | 9.78 (8.31 to 11.42) | 11,686.75 (9,769.25 to 13,994.38) | 0.01 (-0.07 to 0.10) | 3.71 (4.93 to 2.80) | 4,488.35 (3,087.24 to 6,177.63) | 0.01 (-0.12 to 0.16) | 1.32 (1.76 to 0.94) | 1,596.07 (1,107.57 to 2,166.24) | 0.01 (-0.08 to 0.11) |
| Female | 4.98 (4.16 to 5.93) | 11,777.75 (9,664.52 to 14,459.79) | 0.01 (-0.08 to 0.12) | 2.18 (2.95 to 1.57) | 5,212.37 (3,492.83 to 7,386.12) | 0.01 (-0.14 to 0.19) | 0.71 (0.97 to 0.49) | 1,684.84 (1,138.28 to 2,331.40) | 0.01 (-0.10 to 0.13) |
| Male | 4.80 (4.11 to 5.51) | 11,576.65 (9,788.82 to 13,683.95) | 0.01 (-0.06 to 0.08) | 1.53 (1.99 to 1.17) | 3,728.85 (2,678.90 to 5,026.67) | 0.01 (-0.11 to 0.14) | 0.61 (0.81 to 0.44) | 1,501.88 (1,053.92 to 2,027.18) | 0.01 (-0.08 to 0.11) |
| **Nigeria** | | | | | | | | | |
| Total | 87.07 (77.55 to 97.25) | 11,227.85 (9,768.15 to 12,762.15) | 0.03 (0.01 to 0.05) | 33.09 (39.79 to 27.16) | 4,298.41 (3,297.83 to 5,479.81) | 0.04 (0.02 to 0.07) | 11.98 (15.75 to 8.74) | 1,558.39 (1,116.81 to 2,063.57) | 0.03 (0.01to 0.05) |
| Female | 45.38 (40.23 to 51) | 11,185.38 (9,624.22 to 12,833.79) | 0.03 (0.01 to 0.06) | 19.01 (23.49 to 15.43) | 4,711.41 (3,563.34 to 6,065.52) | 0.05 (0.02 to 0.08) | 6.52 (8.65 to 4.71) | 1,617.86 (1,141.92 to 2,166.68) | 0.04 (0.01 to 0.06) |
| Male | 41.69 (37.36 to 46.32) | 11,257.47 (9,834.31 to 12,763.44) | 0.02 (0.01 to 0.04) | 14.08 (16.52 to 11.81) | 3,833.05 (2,952.67 to 4,839.61) | 0.04 (0.01 to 0.06) | 5.46 (7.11 to 4.01) | 1,490.15 (1,074.63 to 1,967.96) | 0.02 (0.00 to 0.04) |
| **Niue** | | | | | | | | | |
| Total | 0.00 (0.00 to 0.00) | 13,291.57 (10,893.63 to 16,212.80) | 0.09 (-0.01 to 0.20) | 0.00 (0.00 to 0.00) | 4,929.31 (3,422.26 to 6,900.95) | 0.16 (0.00 to 0.35) | 0.00 (0.00 to 0.00) | 1,742.09 (1,179.19 to 2,419.75) | 0.11 (-0.00 to 0.24) |
| Female | 0.00 (0.00 to 0.00) | 13,188.96 (10,471.89 to 16,743.84) | 0.11 (-0.01 to 0.27) | 0.00 (0.00 to 0.00) | 5,254.70 (3,650.40 to 7,452.66) | 0.17 (-0.01 to 0.38) | 0.00 (0.00 to 0.00) | 1,824.15 (1,195.65 to 2,610.17) | 0.13 (-0.01 to 0.30) |
| Male | 0.00 (0.00 to 0.00) | 13,362.90 (11,134.45 to 15,952.59) | 0.07 (-0.01 to 0.15) | 0.00 (0.00 to 0.00) | 4,647.92 (3,214.87 to 6,478.65) | 0.15 (-0.01 to 0.34) | 0.00 (0.00 to 0.00) | 1,669.66 (1,139.29 to 2,308.85) | 0.10 (-0.00 to 0.21) |
| **North Macedonia** | | | | | | | | | |
| Total | 0.54 (0.45 to 0.64) | 14,433.25 (11,765.05 to 17,481.98) | 0.24 (0.13 to 0.37) | 0.20 (0.25 to 0.15) | 5,237.51 (3,757.66 to 7,064.43) | 0.51 (0.30 to 0.76) | 0.07 (0.10 to 0.05) | 1,930.45 (1,325.35 to 2,646.90) | 0.30 (0.16 to 0.46) |
| Female | 0.27 (0.22 to 0.33) | 15,214.05 (12,015.40 to 19,030.62) | 0.32 (0.18 to 0.47) | 0.12 (0.16 to 0.09) | 6,434.25 (4,439.98 to 8,990.09) | 0.58 (0.33 to 0.85) | 0.04 (0.05 to 0.03) | 2,167.86 (1,435.06 to 3,038.22) | 0.37 (0.20 to 0.56) |
| Male | 0.26 (0.22 to 0.31) | 13,707.86 (11,437.33 to 16,339.88) | 0.17 (0.08 to 0.27) | 0.08 (0.10 to 0.06) | 4,122.87 (3,040.18 to 5,406.86) | 0.43 (0.25 to 0.66) | 0.03 (0.04 to 0.02) | 1,709.43 (1,181.51 to 2,337.63) | 0.23 (0.11 to 0.37) |
| **Northern Mariana Islands** | | | | | | | | | |
| Total | 0.02 (0.01 to 0.02) | 13,822.10 (11,296.58 to 17,012.62) | 0.15 (0.04 to 0.27) | 0.01 (0.01 to 0.00) | 5,311.44 (3,853.76 to 7,283.61) | 0.30 (0.12 to 0.55) | 0.00 (0.00 to 0.00) | 1,854.76 (1,258.76 to 2,584.05) | 0.19 (0.06 to 0.36) |
| Female | 0.01 (0.01 to 0.01) | 13,577.19 (10,672.28 to 17,286.43) | 0.18 (0.04 to 0.34) | 0.00 (0.00 to 0.00) | 5,082.85 (3,638.39 to 7,043.96) | 0.31 (0.12 to 0.55) | 0.00 (0.00 to 0.00) | 1,882.45 (1,248.34 to 2,685.68) | 0.22 (0.06 to 0.41) |
| Male | 0.01 (0.01 to 0.01) | 14,047.70 (11,620.97 to 16,868.81) | 0.12 (0.03 to 0.21) | 0.00 (0.00 to 0.00) | 5,525.39 (3,956.05 to 7,645.97) | 0.29 (0.1 to 0.55) | 0.00 (0.00 to 0.00) | 1,829.07 (1,248.24 to 2,523.98) | 0.17 (0.04 to 0.32) |
| **Norway** | | | | | | | | | |
| Total | 1.99 (1.75 to 2.24) | 20,043.83 (17,250.48 to 23,099.03) | 0.08 (0.04 to 0.13) | 0.85 (1.06 to 0.68) | 8,526.67 (6,454.51 to 10,997.58) | 0.12 (0.06 to 0.19) | 0.29 (0.39 to 0.21) | 2,929.66 (2,055.53 to 3,957.93) | 0.09 (0.04 to 0.14) |
| Female | 1.08 (0.94 to 1.22) | 22,317.26 (18,979.65 to 26,053.60) | 0.10 (0.05 to 0.15) | 0.51 (0.64 to 0.4) | 10,530.92 (7,931.71 to 13,696.35) | 0.13 (0.06 to 0.21) | 0.17 (0.22 to 0.12) | 3,420.07 (2,360.48 to 4,678.34) | 0.10 (0.05 to 0.16) |
| Male | 0.91 (0.81 to 1.03) | 17,909.23 (15,538.39 to 20,503.15) | 0.07 (0.02 to 0.11) | 0.34 (0.42 to 0.27) | 6,642.06 (5,030.60 to 8,537.93) | 0.11 (0.04 to 0.18) | 0.13 (0.17 to 0.09) | 2,468.82 (1,769.21 to 3,304.95) | 0.08 (0.02 to 0.14) |
| **Oman** | | | | | | | | | |
| Total | 1.70 (1.40 to 2.03) | 18,379.91 (14,869.11 to 22,463.10) | 0.13 (0.01 to 0.26) | 0.76 (1.02 to 0.55) | 8,214.89 (5,624.24 to 11,711.11) | 0.23 (0.02 to 0.42) | 0.24 (0.34 to 0.16) | 2,645.11 (1,765.01 to 3,729.41) | 0.15 (0.02 to 0.30) |
| Female | 0.84 (0.68 to 1.03) | 19,618.01 (15,366.02 to 24,570.31) | 0.15 (0.01 to 0.29) | 0.41 (0.56 to 0.29) | 9,565.61 (6,387.69 to 13,910.95) | 0.24 (0.03 to 0.44) | 0.13 (0.18 to 0.08) | 2,977.99 (1,936.28 to 4,269.48) | 0.16 (0.01 to 0.33) |
| Male | 0.86 (0.73 to 1.01) | 17,408.16 (14,411.79 to 20,925.25) | 0.11 (0.01 to 0.23) | 0.35 (0.46 to 0.26) | 7,053.86 (4,939.34 to 9,761.91) | 0.21 (0.01 to 0.42) | 0.12 (0.16 to 0.08) | 2,367.42 (1,610.20 to 3,296.49) | 0.13 (0.02 to 0.29) |
| **Pakistan** | | | | | | | | | |
| Total | 92.42 (80.8 to 105.15) | 12,757.65 (10,919.10 to 14,828.69) | 0.06 (0.02 to 0.11) | 33.13 (41.88 to 26.45) | 4,588.81 (3,421.90 to 6,034.59) | 0.13 (0.04 to 0.23) | 11.08 (14.71 to 8.04) | 1,533.17 (1,092.14 to 2,058.91) | 0.08 (0.02 to 0.15) |
| Female | 42.06 (36.31 to 48.74) | 12,050.72 (10,155.03 to 14,196.69) | 0.08 (0.03 to 0.14) | 17.96 (23.12 to 14.07) | 5,154.48 (3,775.86 to 6,831.51) | 0.15 (0.05 to 0.27) | 5.42 (7.23 to 3.91) | 1,554.04 (1,096.30 to 2,101.94) | 0.10 (0.03 to 0.19) |
| Male | 50.36 (43.94 to 56.99) | 13,403.34 (11,515.01 to 15,457.28) | 0.04 (0.01 to 0.08) | 15.17 (18.91 to 12.18) | 4,055.77 (3,041.91 to 5,290.04) | 0.11 (0.03 to 0.20) | 5.66 (7.50 to 4.13) | 1,512.34 (1,078.47 to 2,027.13) | 0.06 (0.01 to 0.13) |
| **Palau** | | | | | | | | | |
| Total | 0.00 (0.00 to 0.01) | 13,213.57 (10,752.35 to 15,901.58) | 0.08 (-0.01 to 0.20) | 0.00 (0.00 to 0.01) | 4,953.53 (3,362.89 to 7,000.01) | 0.16 (0.00 to 0.36) | 0.00 (0.00 to 0.00) | 1,739.84 (1,179.54 to 2,447.63) | 0.11 (-0.01 to 0.25) |
| Female | 0.00 (0.00 to 0.00) | 13,160.09 (10,354.83 to 16,346.99) | 0.11 (-0.02 to 0.26) | 0.00 (0.00 to 0.00) | 5,302.15 (3,531.11 to 7,650.22) | 0.18 (0.00 to 0.39) | 0.00 (0.00 to 0.00) | 1,825.92 (1,206.37 to 2,615.26) | 0.13 (-0.02 to 0.29) |
| Male | 0.00 (0.00 to 0.00) | 13,316.20 (11,010.66 to 15,754.77) | 0.06 (-0.01 to 0.16) | 0.00 (0.00 to 0.00) | 4,654.08 (3,175.15 to 6,495.34) | 0.15 (-0.02 to 0.33) | 0.00 (0.00 to 0.00) | 1,669.37 (1,140.03 to 2,333.83) | 0.09 (-0.01 to 0.22) |
| **Palestine** | | | | | | | | | |
| Total | 3.60 (3.02 to 4.33) | 21,846.99 (17,700.40 to 26,579.23) | 0.13 (0.02 to 0.26) | 1.91 (2.64 to 1.39) | 11,627.35 (7,925.94 to 16,866.59) | 0.24 (0.04 to 0.47) | 0.53 (0.74 to 0.36) | 3,216.26 (2,146.02 to 4,560.04) | 0.17 (0.03 to 0.33) |
| Female | 1.80 (1.46 to 2.22) | 22,392.98 (17,661.93 to 28,240.55) | 0.15 (0.02 to 0.31) | 0.97 (1.36 to 0.69) | 12,032.45 (7,984.45 to 17,611.40) | 0.25 (0.04 to 0.50) | 0.27 (0.38 to 0.18) | 3,404.27 (2,219.51 to 4,859.22) | 0.19 (0.03 to 0.37) |
| Male | 1.80 (1.53 to 2.13) | 21,328.47 (17,688.64 to 25,698.51) | 0.11 (0.02 to 0.23) | 0.95 (1.3 to 0.68) | 11,241.87 (7,640.65 to 16,442.51) | 0.23 (0.01 to 0.53) | 0.26 (0.36 to 0.18) | 3,037.45 (2,033.06 to 4,321.43) | 0.16 (0.02 to 0.31) |
| **Panama** | | | | | | | | | |
| Total | 1.70 (1.43 to 2) | 15,365.54 (12,692.98 to 18,586.52) | 0.13 (0.03 to 0.24) | 0.6 (0.79 to 0.46) | 5,426.25 (3,943.46 to 7,384.05) | 0.28 (0.10 to 0.49) | 0.22 (0.30 to 0.16) | 2,010.46 (1,377.33 to 2,725.03) | 0.16 (0.04 to 0.30) |
| Female | 0.85 (0.70 to 1.04) | 15,730.44 (12,701.89 to 19,669.22) | 0.16 (0.04 to 0.32) | 0.35 (0.47 to 0.26) | 6,480.16 (4,589.75 to 9,146.56) | 0.32 (0.11 to 0.54) | 0.12 (0.16 to 0.08) | 2,231.53 (1,492.27 to 3,078.52) | 0.20 (0.05 to 0.37) |
| Male | 0.85 (0.72 to 0.98) | 15,016.00 (12,619.98 to 17,734.57) | 0.09 (0.02 to 0.18) | 0.25 (0.32 to 0.20) | 4,421.53 (3,265.95 to 5,843.96) | 0.24 (0.07 to 0.45) | 0.10 (0.13 to 0.07) | 1,799.60 (1,261.82 to 2,420.67) | 0.13 (0.02 to 0.26) |
| **Papua New Guinea** | | | | | | | | | |
| Total | 4.02 (3.37 to 4.70) | 13,159.57 (10,787.64 to 15,868.62) | 0.05 (-0.05 to 0.15) | 1.44 (1.94 to 1.09) | 4,724.95 (3,381.76 to 6,520.50) | 0.08 (-0.08 to 0.27) | 0.51 (0.70 to 0.35) | 1,677.11 (1,141.79 to 2,342.48) | 0.06 (-0.06 to 0.19) |
| Female | 1.87 (1.53 to 2.29) | 12,922.74 (10,296.72 to 16,201.81) | 0.06 (-0.07 to 0.21) | 0.71 (0.95 to 0.54) | 4,921.52 (3,483.68 to 6,826.53) | 0.08 (-0.10 to 0.29) | 0.25 (0.35 to 0.17) | 1,732.95 (1,143.51 to 2,470.74) | 0.07 (-0.07 to 0.23) |
| Male | 2.15 (1.81 to 2.49) | 13,368.10 (11,184.40 to 15,937.04) | 0.04 (-0.04 to 0.12) | 0.73 (0.99 to 0.55) | 4,548.29 (3,264.64 to 6,348.48) | 0.08 (-0.09 to 0.27) | 0.26 (0.35 to 0.18) | 1,626.48 (1,135.51 to 2,266.89) | 0.05 (-0.07 to 0.17) |
| **Paraguay** | | | | | | | | | |
| Total | 3.97 (3.30 to 4.73) | 19,869.09 (16,218.10 to 24,045.98) | 0.16 (0.06 to 0.28) | 1.47 (1.92 to 1.11) | 7,305.25 (5,318.38 to 9,926.57) | 0.34 (0.15 to 0.57) | 0.52 (0.72 to 0.37) | 2,605.13 (1,795.85 to 3,667.16) | 0.22 (0.09 to 0.36) |
| Female | 2.15 (1.75 to 2.66) | 21,917.48 (17,381.81 to 27,369.05) | 0.21 (0.08 to 0.35) | 0.98 (1.31 to 0.72) | 9,948.46 (7,053.94 to 13,987.79) | 0.39 (0.16 to 0.67) | 0.31 (0.44 to 0.22) | 3,171.18 (2,141.48 to 4,494.64) | 0.26 (0.11 to 0.42) |
| Male | 1.82 (1.55 to 2.12) | 17,896.40 (14,808.01 to 21,371.47) | 0.11 (0.03 to 0.20) | 0.49 (0.61 to 0.38) | 4,766.10 (3,577.31 to 6,230.74) | 0.26 (0.11 to 0.47) | 0.21 (0.29 to 0.15) | 2,061.05 (1,439.00 to 2,875.91) | 0.16 (0.04 to 0.28) |
| **Peru** | | | | | | | | | |
| Total | 17.01 (14.13 to 20.29) | 18,455.34 (15,081.98 to 22,547.61) | 0.21 (0.10 to 0.34) | 4.78 (6.09 to 3.72) | 5,168.19 (3,788.56 to 6,773.73) | 0.44 (0.26 to 0.65) | 2.10 (2.92 to 1.46) | 2,258.10 (1,537.89 to 3,151.82) | 0.28 (0.14 to 0.44) |
| Female | 8.39 (6.78 to 10.39) | 19,010.34 (15,032.64 to 24,020.67) | 0.28 (0.13 to 0.43) | 2.76 (3.57 to 2.09) | 6,242.12 (4,459.18 to 8,382.38) | 0.49 (0.30 to 0.75) | 1.14 (1.61 to 0.78) | 2,570.53 (1,710.52 to 3,636.31) | 0.32 (0.16 to 0.51) |
| Male | 8.62 (7.25 to 10.17) | 17,933.22 (14,878.25 to 21,816.34) | 0.15 (0.06 to 0.26) | 2.02 (2.52 to 1.62) | 4,189.91 (3,102.34 to 5,403.43) | 0.37 (0.21 to 0.57) | 0.96 (1.30 to 0.68) | 1,972.42 (1,344.52 to 2,723.76) | 0.23 (0.09 to 0.39) |
| **Philippines** | | | | | | | | | |
| Total | 43.22 (38.91 to 48.42) | 13,284.55 (11,509.19 to 15,223.12) | 0.15 (0.12 to 0.18) | 16.98 (21.17 to 13.66) | 5,211.50 (3,937.15 to 6,729.58) | 0.32 (0.27 to 0.37) | 5.77 (7.63 to 4.19) | 1,770.86 (1,254.19 to 2,382.95) | 0.20 (0.16 to 0.23) |
| Female | 21.68 (19.25 to 24.50) | 13,649.80 (11,693.91 to 15,796.30) | 0.19 (0.15 to 0.22) | 8.74 (10.94 to 7.00) | 5,500.07 (4,146.66 to 7,069.09) | 0.34 (0.30 to 0.39) | 2.98 (3.98 to 2.14) | 1,870.70 (1,314.22 to 2,525.39) | 0.23 (0.19 to 0.27) |
| Male | 21.54 (19.31 to 24.03) | 12,932.03 (11,235.48 to 14,737.76) | 0.12 (0.09 to 0.14) | 8.24 (10.23 to 6.62) | 4,936.81 (3,711.58 to 6,430.16) | 0.29 (0.25 to 0.34) | 2.80 (3.65 to 2.05) | 1,675.46 (1,195.80 to 2,242.13) | 0.16 (0.13 to 0.20) |
| **Poland** | | | | | | | | | |
| Total | 7.46 (6.65 to 8.34) | 12,806.61 (11,087.05 to 14,651.09) | 0.15 (0.11 to 0.18) | 2.45 (2.95 to 2.05) | 4,192.32 (3,251.84 to 5,279.54) | 0.30 (0.23 to 0.36) | 0.97 (1.29 to 0.71) | 1,666.66 (1,192.01 to 2,224.96) | 0.18 (0.13 to 0.23) |
| Female | 3.61 (3.20 to 4.08) | 12,684.10 (10,884.52 to 14,713.86) | 0.19 (0.15 to 0.23) | 1.23 (1.49 to 1.02) | 4,305.60 (3,319.26 to 5,415.70) | 0.32 (0.25 to 0.39) | 0.50 (0.66 to 0.35) | 1,737.29 (1,222.08 to 2,336.94) | 0.21 (0.16 to 0.27) |
| Male | 3.85 (3.43 to 4.30) | 12,922.21 (11,217.53 to 14,809.17) | 0.11 (0.08 to 0.14) | 1.22 (1.48 to 1.02) | 4,084.46 (3,146.41 to 5,168.84) | 0.27 (0.21 to 0.34) | 0.48 (0.63 to 0.35) | 1,599.33 (1,147.19 to 2,118.65) | 0.15 (0.10 to 0.19) |
| **Portugal** | | | | | | | | | |
| Total | 4.09 (3.40 to 4.89) | 24,836.21 (19,975.85 to 30,611.01) | 0.20 (0.08 to 0.35) | 1.76 (2.37 to 1.28) | 10,670.85 (7,476.98 to 14,849.61) | 0.39 (0.17 to 0.63) | 0.61 (0.82 to 0.41) | 3,645.25 (2,445.73 to 5,069.82) | 0.25 (0.11 to 0.41) |
| Female | 2.33 (1.90 to 2.85) | 28,705.19 (22,664.14 to 36,071.31) | 0.24 (0.11 to 0.40) | 1.19 (1.63 to 0.85) | 14,678.59 (9,989.13 to 21,007.99) | 0.41 (0.18 to 0.69) | 0.37 (0.51 to 0.24) | 4,507.63 (2,951.89 to 6,363.80) | 0.28 (0.13 to 0.45) |
| Male | 1.76 (1.47 to 2.07) | 21,116.44 (17,304.99 to 25,632.43) | 0.15 (0.05 to 0.29) | 0.57 (0.73 to 0.44) | 6,821.33 (4,960.27 to 9,085.88) | 0.34 (0.15 to 0.57) | 0.24 (0.32 to 0.17) | 2,816.57 (1,918.75 to 3,824.43) | 0.20 (0.07 to 0.39) |
| **Puerto Rico** | | | | | | | | | |
| Total | 1.09 (0.92 to 1.28) | 17,662.54 (14,670.64 to 21,276.77) | 0.08 (0.00 to 0.16) | 0.31 (0.40 to 0.24) | 4,995.06 (3,694.76 to 6,690.14) | 0.20 (0.04 to 0.39) | 0.12 (0.17 to 0.09) | 1,997.45 (1,369.85 to 2,730.66) | 0.12 (0.01 to 0.23) |
| Female | 0.51 (0.42 to 0.61) | 16,550.51 (13,433.15 to 20,407.04) | 0.11 (0.00 to 0.23) | 0.18 (0.24 to 0.14) | 5,845.07 (4,213.90 to 8,059.85) | 0.21 (0.04 to 0.44) | 0.07 (0.09 to 0.05) | 2,190.69 (1,444.01 to 3,049.21) | 0.14 (0.01 to 0.28) |
| Male | 0.58 (0.49 to 0.69) | 18,725.49 (15,531.42 to 22,674.81) | 0.05 (0.00 to 0.11) | 0.13 (0.16 to 0.10) | 4,163.86 (3,112.07 to 5,427.00) | 0.17 (0.02 to 0.36) | 0.06 (0.08 to 0.04) | 1,807.47 (1,285.26 to 2,433.09) | 0.09 (-0.01 to 0.20) |
| **Qatar** | | | | | | | | | |
| Total | 0.76 (0.64 to 0.90) | 17,007.35 (14,065.29 to 20,595.74) | 0.06 (-0.04 to 0.19) | 0.34 (0.46 to 0.25) | 7,567.09 (5,232.69 to 10,773.04) | 0.13 (-0.05 to 0.33) | 0.11 (0.15 to 0.08) | 2,487.87 (1,703.08 to 3,460.67) | 0.07 (-0.04 to 0.20) |
| Female | 0.31 (0.25 to 0.37) | 18,346.95 (14,617.67 to 22,934.52) | 0.07 (-0.05 to 0.22) | 0.15 (0.21 to 0.11) | 8,950.03 (6,049.59 to 13,133.27) | 0.12 (-0.07 to 0.32) | 0.05 (0.07 to 0.03) | 2,848.48 (1,895.22 to 4,074.56) | 0.08 (-0.06 to 0.23) |
| Male | 0.45 (0.38 to 0.53) | 16,433.46 (13,782.32 to 19,575.62) | 0.05 (-0.04 to 0.17) | 0.19 (0.26 to 0.14) | 6,680.95 (4,689.44 to 9,313.90) | 0.11 (-0.07 to 0.34) | 0.06 (0.09 to 0.05) | 2,285.24 (1,599.67 to 3,159.43) | 0.06 (-0.05 to 0.19) |
| **Republic of Korea** | | | | | | | | | |
| Total | 10.91 (9.32 to 12.56) | 14,087.27 (11,861.86 to 16,686.00) | 0.06 (-0.02 to 0.15) | 3.98 (5.03 to 3.04) | 5,091.00 (3,751.26 to 6,752.67) | 0.14 (-0.02 to 0.33) | 1.54 (2.05 to 1.10) | 1,951.10 (1,384.81 to 2,650.17) | 0.07 (-0.03 to 0.17) |
| Female | 5.19 (4.30 to 6.19) | 13,660.07 (11,128.33 to 16,769.46) | 0.08 (-0.03 to 0.21) | 2.23 (2.91 to 1.71) | 5,810.13 (4,267.58 to 7,746.25) | 0.16 (-0.03 to 0.37) | 0.79 (1.07 to 0.56) | 2,043.74 (1,404.82 to 2,821.62) | 0.09 (-0.03 to 0.22) |
| Male | 5.72 (4.98 to 6.53) | 14,498.90 (12,388.76 to 16,770.01) | 0.05 (-0.02 to 0.13) | 1.74 (2.19 to 1.34) | 4,423.82 (3,231.50 to 5,862.96) | 0.12 (-0.04 to 0.30) | 0.75 (0.98 to 0.55) | 1,865.89 (1,338.03 to 2,492.29) | 0.05 (-0.04 to 0.16) |
| **Republic of Moldova** | | | | | | | | | |
| Total | 0.82 (0.69 to 0.97) | 14,939.32 (12,220.43 to 18,133.85) | 0.17 (0.06 to 0.29) | 0.29 (0.38 to 0.22) | 5,315.03 (3,859.65 to 7,177.95) | 0.37 (0.18 to 0.60) | 0.11 (0.14 to 0.08) | 1,970.02 (1,369.58 to 2,693.14) | 0.22 (0.09 to 0.37) |
| Female | 0.41 (0.34 to 0.51) | 15,454.33 (12,381.28 to 19,300.99) | 0.22 (0.09 to 0.37) | 0.16 (0.22 to 0.12) | 6,128.70 (4,320.61 to 8,479.55) | 0.39 (0.18 to 0.64) | 0.06 (0.08 to 0.04) | 2,154.55 (1,441.39 to 2,983.58) | 0.26 (0.10 to 0.43) |
| Male | 0.40 (0.35 to 0.47) | 14,450.42 (12,115.92 to 17,160.39) | 0.13 (0.04 to 0.23) | 0.13 (0.16 to 0.10) | 4,544.29 (3,321.54 to 6,064.31) | 0.33 (0.14 to 0.57) | 0.05 (0.07 to 0.04) | 1,795.18 (1,258.48 to 2,444.50) | 0.18 (0.06 to 0.32) |
| **Romania** | | | | | | | | | |
| Total | 4.24 (3.59 to 4.94) | 13,659.49 (11,352.05 to 16,307.11) | 0.19 (0.09 to 0.31) | 1.55 (1.99 to 1.20) | 4,979.35 (3,625.78 to 6,742.00) | 0.41 (0.22 to 0.65) | 0.57 (0.77 to 0.40) | 1,842.60 (1,279.07 to 2,509.95) | 0.24 (0.11 to 0.39) |
| Female | 2.12 (1.75 to 2.55) | 14,074.86 (11,348.79 to 17,418.70) | 0.25 (0.12 to 0.40) | 0.88 (1.17 to 0.67) | 5,838.55 (4,081.81 to 8,058.01) | 0.46 (0.25 to 0.71) | 0.31 (0.41 to 0.21) | 2,020.83 (1,354.81 to 2,798.30) | 0.30 (0.15 to 0.46) |
| Male | 2.12 (1.82 to 2.42) | 13,267.80 (11,174.10 to 15,464.93) | 0.13 (0.06 to 0.22) | 0.67 (0.84 to 0.52) | 4,168.24 (3,084.68 to 5,429.60) | 0.35 (0.18 to 0.58) | 0.27 (0.36 to 0.19) | 1,674.37 (1,174.91 to 2,263.02) | 0.19 (0.06 to 0.33) |
| **Russian Federation** | | | | | | | | | |
| Total | 33.34 (29.95 to 37.11) | 14,202.36 (12,343.57 to 16,179.07) | 0.18 (0.15 to 0.22) | 13.30 (16.28 to 10.79) | 5,701.36 (4,306.07 to 7,329.25) | 0.37 (0.32 to 0.43) | 4.49 (5.91 to 3.25) | 1,923.39 (1,363.25 to 2,573.29) | 0.23 (0.19 to 0.27) |
| Female | 16.47 (14.69 to 18.57) | 14,404.35 (12,376.02 to 16,629.04) | 0.23 (0.20 to 0.27) | 7.03 (8.64 to 5.66) | 6,178.80 (4,632.07 to 7,952.65) | 0.40 (0.35 to 0.45) | 2.34 (3.09 to 1.68) | 2,052.75 (1,428.60 to 2,764.59) | 0.27 (0.22 to 0.31) |
| Male | 16.87 (15.07 to 18.87) | 14,007.70 (12,198.61 to 16,022.02) | 0.14 (0.11 to 0.17) | 6.27 (7.63 to 5.11) | 5,245.60 (3,978.92 to 6,738.42) | 0.34 (0.28 to 0.40) | 2.16 (2.83 to 1.58) | 1,799.80 (1,287.94 to 2,388.60) | 0.19 (0.15 to 0.24) |
| **Rwanda** | | | | | | | | | |
| Total | 5.88 (4.98 to 6.92) | 13,646.56 (11,321.89 to 16,468.60) | 0.11 (0.03 to 0.22) | 2.81 (3.8 to 2.08) | 6,524.70 (4,503.73 to 9,187.57) | 0.23 (0.06 to 0.43) | 0.85 (1.16 to 0.60) | 1,985.25 (1,357.04 to 2,761.35) | 0.15 (0.04 to 0.27) |
| Female | 3.11 (2.59 to 3.72) | 14,306.25 (11,605.45 to 17,572.30) | 0.14 (0.04 to 0.26) | 1.72 (2.41 to 1.23) | 7,926.71 (5,335.87 to 11,405.61) | 0.25 (0.06 to 0.47) | 0.47 (0.67 to 0.32) | 2,181.21 (1,447.52 to 3,101.93) | 0.17 (0.05 to 0.32) |
| Male | 2.77 (2.41 to 3.21) | 12,964.30 (10,939.15 to 15,492.40) | 0.09 (0.01 to 0.17) | 1.09 (1.44 to 0.83) | 5,090.09 (3,603.99 to 7,079.51) | 0.20 (0.03 to 0.41) | 0.38 (0.51 to 0.27) | 1,784.05 (1,246.74 to 2,438.71) | 0.11 (0.01 to 0.23) |
| **Saint Kitts and Nevis** | | | | | | | | | |
| Total | 0.02 (0.02 to 0.03) | 18,230.34 (15,026.97 to 21,830.21) | 0.09 (0.02 to 0.18) | 0.01 (0.01 to 0.01) | 6,146.51 (4,203.33 to 8,822.51) | 0.24 (0.06 to 0.44) | 0.00 (0.00 to 0.00) | 2,129.09 (1,446.00 to 2,937.07) | 0.14 (0.03 to 0.26) |
| Female | 0.01 (0.01 to 0.01) | 17,485.91 (14,074.16 to 21,357.79) | 0.13 (0.03 to 0.25) | 0.00 (0.01 to 0.00) | 7,647.78 (5,047.56 to 11,225.47) | 0.26 (0.06 to 0.48) | 0.00 (0.00 to 0.00) | 2,401.13 (1,584.58 to 3,393.73) | 0.17 (0.04 to 0.32) |
| Male | 0.01 (0.01 to 0.01) | 18,959.69 (15,781.89 to 22,757.14) | 0.06 (0.00 to 0.12) | 0.00 (0.00 to 0.00) | 4,638.58 (3,269.14 to 6,404.56) | 0.21 (0.06 to 0.40) | 0.00 (0.00 to 0.00) | 1,855.25 (1,295.41 to 2,541.14) | 0.11 (0.02 to 0.22) |
| **Saint Lucia** | | | | | | | | | |
| Total | 0.07 (0.06 to 0.08) | 18,989.82 (15,945.40 to 22,530.45) | 0.14 (0.06 to 0.24) | 0.02 (0.03 to 0.02) | 6,492.50 (4,654.72 to 8,978.99) | 0.38 (0.18 to 0.64) | 0.01 (0.01 to 0.01) | 2,225.71 (1,521.17 to 3,087.27) | 0.22 (0.10 to 0.36) |
| Female | 0.03 (0.03 to 0.04) | 18,208.80 (14,842.58 to 22,265.61) | 0.20 (0.09 to 0.33) | 0.01 (0.02 to 0.01) | 7,760.54 (5,382.80 to 10,969.87) | 0.42 (0.19 to 0.71) | 0.00 (0.01 to 0.00) | 2,477.80 (1,645.97 to 3,508.90) | 0.26 (0.12 to 0.42) |
| Male | 0.04 (0.03 to 0.04) | 19,729.95 (16,563.53 to 23,608.71) | 0.09 (0.03 to 0.18) | 0.01 (0.01 to 0.01) | 5,289.47 (3,853.03 to 7,119.20) | 0.34 (0.13 to 0.58) | 0.00 (0.01 to 0.00) | 1,986.34 (1,404.14 to 2,712.58) | 0.18 (0.06 to 0.31) |
| **Saint Vincent and the Grenadines** | | | | | | | | | |
| Total | 0.05 (0.04 to 0.06) | 18,573.11 (15,610.56 to 21,944.27) | 0.11 (0.03 to 0.20) | 0.02 (0.02 to 0.01) | 6,208.40 (4,473.55 to 8,585.19) | 0.30 (0.13 to 0.52) | 0.01 (0.01 to 0.00) | 2,152.68 (1,491.72 to 2,946.94) | 0.18 (0.06 to 0.30) |
| Female | 0.02 (0.02 to 0.03) | 17,545.89 (14,402.48 to 21,318.17) | 0.16 (0.04 to 0.28) | 0.01 (0.01 to 0.01) | 7,159.95 (4,990.60 to 10,168.72) | 0.33 (0.13 to 0.55) | 0.00 (0.00 to 0.00) | 2,353.55 (1,596.84 to 3,324.51) | 0.21 (0.07 to 0.36) |
| Male | 0.03 (0.02 to 0.03) | 19,557.50 (16,438.64 to 23,169.78) | 0.07 (0.02 to 0.14) | 0.01 (0.01 to 0.01) | 5,311.79 (3,887.63 to 7,215.69) | 0.27 (0.08 to 0.52) | 0.00 (0.00 to 0.00) | 1,964.03 (1,384.30 to 2,685.74) | 0.14 (0.04 to 0.28) |
| **Samoa** | | | | | | | | | |
| Total | 0.08 (0.07 to 0.10) | 13,294.96 (10,838.18 to 16,231.52) | 0.08 (-0.02 to 0.19) | 0.03 (0.04 to 0.02) | 4,569.67 (3,240.09 to 6,215.38) | 0.16 (-0.02 to 0.34) | 0.01 (0.01 to 0.01) | 1,696.61 (1,170.03 to 2,355.85) | 0.11 (-0.02 to 0.24) |
| Female | 0.04 (0.03 to 0.05) | 13,147.77 (10,407.97 to 16,555.45) | 0.11 (-0.02 to 0.25) | 0.01 (0.02 to 0.01) | 4,737.20 (3,346.76 to 6,468.02) | 0.17 (0.00 to 0.36) | 0.01 (0.01 to 0.00) | 1,756.95 (1,179.55 to 2,488.14) | 0.13 (-0.03 to 0.30) |
| Male | 0.04 (0.04 to 0.05) | 13,434.14 (11,152.57 to 16,035.15) | 0.06 (-0.01 to 0.15) | 0.01 (0.02 to 0.01) | 4,412.06 (3,083.89 to 6,117.19) | 0.14 (-0.03 to 0.33) | 0.01 (0.01 to 0.00) | 1,639.94 (1,143.51 to 2,263.40) | 0.09 (-0.03 to 0.21) |
| **San Marino** | | | | | | | | | |
| Total | 0.01 (0.01 to 0.01) | 21,170.94 (17,158.89 to 25,999.99) | 0.17 (0.05 to 0.29) | 0.01 (0.01 to 0.00) | 9,587.85 (6,456.06 to 13,811.63) | 0.33 (0.12 to 0.56) | 0.00 (0.00 to 0.00) | 3,111.23 (2,076.33 to 4,404.56) | 0.21 (0.07 to 0.35) |
| Female | 0.01 (0.01 to 0.01) | 23,509.90 (18,441.71 to 29,853.79) | 0.20 (0.07 to 0.35) | 0.00 (0.00 to 0.00) | 12,284.22 (8,212.21 to 18,172.03) | 0.35 (0.12 to 0.58) | 0.00 (0.00 to 0.00) | 3,691.68 (2,417.11 to 5,357.50) | 0.24 (0.08 to 0.39) |
| Male | 0.01 (0.00 to 0.01) | 18,945.23 (15,669.32 to 22,839.42) | 0.13 (0.03 to 0.24) | 0.00 (0.00 to 0.00) | 7,037.08 (4,847.28 to 9,904.57) | 0.30 (0.12 to 0.52) | 0.00 (0.00 to 0.00) | 2,561.08 (1,758.11 to 3,546.21) | 0.17 (0.06 to 0.31) |
| **Sao Tome and Principe** | | | | | | | | | |
| Total | 0.08 (0.07 to 0.10) | 11,834.94 (9,805.70 to 14,145.12) | 0.05 (-0.03 to 0.14) | 0.03 (0.04 to 0.02) | 4,719.17 (3,293.72 to 6,564.42) | 0.09 (-0.07 to 0.26) | 0.01 (0.02 to 0.01) | 1,671.85 (1,148.16 to 2,315.80) | 0.06 (-0.03 to 0.17) |
| Female | 0.04 (0.04 to 0.05) | 12,042.22 (9,737.90 to 14,813.62) | 0.07 (-0.04 to 0.18) | 0.02 (0.03 to 0.01) | 5,500.91 (3,699.58 to 7,954.06) | 0.1 (-0.08 to 0.28) | 0.01 (0.01 to 0.00) | 1,779.38 (1,175.28 to 2,489.95) | 0.08 (-0.04 to 0.20) |
| Male | 0.04 (0.04 to 0.05) | 11,624.18 (9,778.04 to 13,670.49) | 0.04 (-0.03 to 0.11) | 0.01 (0.02 to 0.01) | 3,930.94 (2,819.72 to 5,305.48) | 0.07 (-0.07 to 0.24) | 0.01 (0.01 to 0.00) | 1,563.31 (1,102.07 to 2,131.25) | 0.05 (-0.04 to 0.15) |
| **Saudi Arabia** | | | | | | | | | |
| Total | 14.02 (11.71 to 16.86) | 16,883.98 (13,728.30 to 20,680.95) | 0.06 (-0.05 to 0.19) | 6.33 (8.52 to 4.85) | 7,588.79 (5,418.38 to 10,571.87) | 0.12 (-0.04 to 0.31) | 2.07 (2.88 to 1.43) | 2,469.13 (1,655.06 to 3,472.89) | 0.08 (-0.04 to 0.22) |
| Female | 6.60 (5.36 to 8.1) | 17,741.32 (14,062.53 to 22,565.58) | 0.08 (-0.06 to 0.22) | 3.35 (4.59 to 2.51) | 9,010.68 (6,289.16 to 12,862.22) | 0.13 (-0.04 to 0.33) | 1.05 (1.47 to 0.71) | 2,791.70 (1,835.30 to 3,968.65) | 0.08 (-0.05 to 0.24) |
| Male | 7.42 (6.30 to 8.79) | 16,255.65 (13,453.07 to 19,470.91) | 0.05 (-0.04 to 0.17) | 2.98 (4.07 to 2.17) | 6,463.23 (4,489.10 to 9,041.32) | 0.12 (-0.05 to 0.31) | 1.03 (1.42 to 0.72) | 2,217.37 (1,504.68 to 3,092.30) | 0.07 (-0.05 to 0.21) |
| **Senegal** | | | | | | | | | |
| Total | 6.47 (5.48 to 7.57) | 12,402.02 (10,245.91 to 14,892.46) | 0.12 (0.02 to 0.23) | 2.67 (3.58 to 2.00) | 5,144.91 (3,636.13 to 7,179.91) | 0.22 (0.06 to 0.42) | 0.91 (1.24 to 0.63) | 1,750.42 (1,182.26 to 2,431.26) | 0.14 (0.03 to 0.28) |
| Female | 3.16 (2.62 to 3.79) | 12,572.81 (10,136.72 to 15,562.83) | 0.15 (0.03 to 0.28) | 1.43 (1.97 to 1.03) | 5,708.77 (3,935.33 to 8,224.51) | 0.24 (0.06 to 0.47) | 0.46 (0.63 to 0.31) | 1,837.01 (1,213.65 to 2,589.26) | 0.16 (0.02 to 0.31) |
| Male | 3.31 (2.82 to 3.80) | 12,236.19 (10,267.26 to 14,528.20) | 0.09 (0.01 to 0.18) | 1.24 (1.62 to 0.93) | 4,616.26 (3,321.60 to 6,345.84) | 0.21 (0.05 to 0.41) | 0.45 (0.61 to 0.31) | 1,668.80 (1,147.47 to 2,299.44) | 0.12 (0.01 to 0.25) |
| **Serbia** | | | | | | | | | |
| Total | 2.15 (1.81 to 2.49) | 13,248.71 (10,946.39 to 15,877.30) | 0.15 (0.05 to 0.26) | 0.74 (0.97 to 0.58) | 4,575.29 (3,340.01 to 6,195.07) | 0.31 (0.12 to 0.51) | 0.28 (0.38 to 0.20) | 1,748.32 (1,202.10 to 2,399.91) | 0.19 (0.06 to 0.32) |
| Female | 1.04 (0.85 to 1.26) | 13,571.97 (10,840.84 to 16,768.56) | 0.20 (0.07 to 0.34) | 0.42 (0.57 to 0.31) | 5,434.94 (3,790.97 to 7,610.64) | 0.35 (0.12 to 0.58) | 0.15 (0.20 to 0.10) | 1,915.03 (1,281.06 to 2,706.26) | 0.23 (0.07 to 0.40) |
| Male | 1.10 (0.94 to 1.27) | 12,939.91 (10,858.54 to 15,261.72) | 0.11 (0.04 to 0.19) | 0.32 (0.41 to 0.26) | 3,793.62 (2,813.03 to 4,971.84) | 0.26 (0.09 to 0.46) | 0.14 (0.18 to 0.10) | 1,596.09 (1,123.20 to 2,169.52) | 0.15 (0.05 to 0.27) |
| **Seychelles** | | | | | | | | | |
| Total | 0.03 (0.02 to 0.03) | 12,796.55 (10,479.36 to 15,523.14) | 0.17 (0.07 to 0.30) | 0.01 (0.02 to 0.01) | 5,170.60 (3,600.00 to 7,146.35) | 0.35 (0.15 to 0.59) | 0.00 (0.01 to 0.00) | 1,766.89 (1,184.78 to 2,472.57) | 0.21 (0.08 to 0.37) |
| Female | 0.01 (0.01 to 0.02) | 13,013.56 (10,353.01 to 16,305.55) | 0.21 (0.07 to 0.39) | 0.01 (0.01 to 0.00) | 5,309.46 (3,634.91 to 7,572.75) | 0.37 (0.17 to 0.63) | 0.00 (0.00 to 0.00) | 1,842.18 (1,202.49 to 2,624.02) | 0.24 (0.08 to 0.46) |
| Male | 0.01 (0.01 to 0.02) | 12,607.29 (10,546.51 to 14,987.93) | 0.13 (0.05 to 0.25) | 0.01 (0.01 to 0.00) | 5,033.93 (3,489.65 to 6,988.76) | 0.32 (0.12 to 0.57) | 0.00 (0.00 to 0.00) | 1,696.62 (1,158.89 to 2,342.04) | 0.18 (0.05 to 0.33) |
| **Sierra Leone** | | | | | | | | | |
| Total | 3.52 (2.97 to 4.13) | 12,104.85 (9,981.93 to 14,583.01) | 0.01 (-0.07 to 0.11) | 1.38 (1.85 to 1.02) | 4,763.80 (3,296.72 to 6,644.98) | 0.01 (-0.13 to 0.19) | 0.49 (0.66 to 0.34) | 1,672.28 (1,144.64 to 2,314.70) | 0.01 (-0.08 to 0.12) |
| Female | 1.83 (1.52 to 2.20) | 12,305.41 (9,930.13 to 15,090.32) | 0.02 (-0.08 to 0.13) | 0.83 (1.13 to 0.59) | 5,565.69 (3,743.66 to 8,022.55) | 0.02 (-0.14 to 0.21) | 0.26 (0.36 to 0.18) | 1,779.98 (1,192.19 to 2,503.34) | 0.02 (-0.10 to 0.15) |
| Male | 1.69 (1.45 to 1.95) | 11,882.37 (9,920.26 to 14,107.41) | 0.01 (-0.06 to 0.09) | 0.56 (0.71 to 0.43) | 3,918.21 (2,783.49 to 5,304.11) | 0.01 (-0.12 to 0.17) | 0.22 (0.3 to 0.16) | 1,557.91 (1,086.74 to 2,142.94) | 0.01 (-0.08 to 0.11) |
| **Singapore** | | | | | | | | | |
| Total | 1.03 (0.89 to 1.18) | 14,096.40 (12,030.27 to 16,531.54) | 0.04 (-0.02 to 0.11) | 0.39 (0.49 to 0.30) | 5,353.48 (3,886.12 to 7,037.41) | 0.10 (-0.06 to 0.27) | 0.15 (0.20 to 0.11) | 2,000.15 (1,405.95 to 2,711.02) | 0.05 (-0.03 to 0.13) |
| Female | 0.48 (0.41 to 0.56) | 13,280.50 (10,930.72 to 16,004.93) | 0.05 (-0.04 to 0.14) | 0.22 (0.28 to 0.16) | 6,025.78 (4,339.00 to 8,072.85) | 0.09 (-0.07 to 0.30) | 0.07 (0.10 to 0.05) | 2,058.30 (1,406.58 to 2,834.53) | 0.05 (-0.05 to 0.15) |
| Male | 0.55 (0.48 to 0.63) | 14,905.26 (12,832.95 to 17,385.78) | 0.04 (-0.02 to 0.09) | 0.18 (0.22 to 0.14) | 4,716.62 (3,453.10 to 6,192.08) | 0.10 (-0.06 to 0.29) | 0.07 (0.10 to 0.05) | 1,946.29 (1,398.01 to 2,613.62) | 0.05 (-0.03 to 0.14) |
| **Slovakia** | | | | | | | | | |
| Total | 1.15 (0.97 to 1.37) | 13,748.93 (11,397.42 to 16,565.81) | 0.20 (0.10 to 0.31) | 0.42 (0.54 to 0.32) | 4,982.92 (3,592.30 to 6,758.62) | 0.42 (0.23 to 0.62) | 0.16 (0.21 to 0.11) | 1,849.58 (1,264.63 to 2,564.51) | 0.24 (0.11 to 0.39) |
| Female | 0.59 (0.48 to 0.71) | 14,344.38 (11,481.62 to 17,775.75) | 0.26 (0.13 to 0.4) | 0.25 (0.33 to 0.18) | 6,051.65 (4,229.79 to 8,555.99) | 0.47 (0.24 to 0.70) | 0.08 (0.12 to 0.06) | 2,061.81 (1,345.82 to 2,946.41) | 0.30 (0.14 to 0.47) |
| Male | 0.57 (0.49 to 0.65) | 13,183.49 (11,119.98 to 15,522.37) | 0.14 (0.06 to 0.23) | 0.17 (0.21 to 0.14) | 3,968.71 (2,952.33 to 5,195.41) | 0.34 (0.18 to 0.54) | 0.07 (0.10 to 0.05) | 1,648.17 (1,156.62 to 2,261.39) | 0.18 (0.06 to 0.31) |
| **Slovenia** | | | | | | | | | |
| Total | 0.39 (0.34 to 0.46) | 13,303.31 (11,048.21 to 15,923.26) | 0.13 (0.04 to 0.24) | 0.15 (0.20 to 0.12) | 5,068.22 (3,657.68 to 6,950.58) | 0.28 (0.10 to 0.52) | 0.05 (0.07 to 0.04) | 1,805.32 (1,253.70 to 2,501.00) | 0.16 (0.05 to 0.29) |
| Female | 0.20 (0.17 to 0.24) | 13,807.98 (11,162.39 to 17,137.63) | 0.17 (0.06 to 0.30) | 0.09 (0.12 to 0.07) | 6,285.91 (4,413.34 to 8,955.23) | 0.30 (0.10 to 0.59) | 0.03 (0.04 to 0.02) | 2,016.09 (1,350.92 to 2,848.48) | 0.20 (0.06 to 0.35) |
| Male | 0.20 (0.17 to 0.22) | 12,831.40 (10,761.15 to 15,019.73) | 0.09 (0.03 to 0.18) | 0.06 (0.08 to 0.05) | 3,925.04 (2,917.30 to 5,172.60) | 0.24 (0.07 to 0.45) | 0.02 (0.03 to 0.02) | 1,607.56 (1,141.92 to 2,169.27) | 0.12 (0.02 to 0.24) |
| **Solomon Islands** | | | | | | | | | |
| Total | 0.29 (0.24 to 0.34) | 13,784.90 (11,359.00 to 16,689.38) | 0.08 (-0.01 to 0.21) | 0.1 (0.13 to 0.07) | 4,734.60 (3,345.38 to 6,547.64) | 0.16 (-0.02 to 0.35) | 0.04 (0.05 to 0.03) | 1,723.51 (1,177.52 to 2,406.28) | 0.11 (-0.01 to 0.25) |
| Female | 0.14 (0.11 to 0.17) | 13,649.80 (10,925.03 to 17,110.93) | 0.10 (-0.02 to 0.27) | 0.05 (0.07 to 0.04) | 5,003.19 (3,482.67 to 6,987.91) | 0.17 (-0.02 to 0.38) | 0.02 (0.03 to 0.01) | 1,792.25 (1,193.55 to 2,548.14) | 0.13 (-0.02 to 0.30) |
| Male | 0.15 (0.13 to 0.18) | 13,900.07 (11,627.80 to 16,575.61) | 0.06 (-0.01 to 0.15) | 0.05 (0.06 to 0.04) | 4,483.32 (3,156.63 to 6,139.92) | 0.14 (-0.02 to 0.34) | 0.02 (0.02 to 0.01) | 1,658.37 (1,151.15 to 2,309.36) | 0.09 (-0.02 to 0.21) |
| **Somalia** | | | | | | | | | |
| Total | 10.88 (9.24 to 12.71) | 15,068.26 (12,549.94 to 17,979.87) | 0.14 (0.05 to 0.26) | 4.70 (6.33 to 3.35) | 6,564.09 (4,433.82 to 9,308.15) | 0.33 (0.11 to 0.59) | 1.45 (1.93 to 1.02) | 2,018.70 (1,388.59 to 2,746.30) | 0.19 (0.07 to 0.33) |
| Female | 5.24 (4.38 to 6.27) | 15,234.43 (12,295.82 to 18,634.14) | 0.18 (0.06 to 0.33) | 2.61 (3.56 to 1.80) | 7,635.33 (5,058.17 to 11,120.17) | 0.36 (0.12 to 0.66) | 0.74 (1.00 to 0.51) | 2,153.08 (1,436.05 to 2,974.17) | 0.23 (0.08 to 0.40) |
| Male | 5.64 (4.88 to 6.50) | 14,917.61 (12,615.25 to 17,529.17) | 0.11 (0.02 to 0.22) | 2.09 (2.74 to 1.52) | 5,583.03 (3,829.45 to 7,768.66) | 0.29 (0.10 to 0.54) | 0.71 (0.94 to 0.51) | 1,895.73 (1,311.90 to 2,582.54) | 0.16 (0.04 to 0.30) |
| **South Africa** | | | | | | | | | |
| Total | 20.53 (18.26 to 23.03) | 14,300.78 (12,340.86 to 16,491.87) | 0.20 (0.15 to 0.25) | 10.23 (12.55 to 8.30) | 7,114.81 (5,462.32 to 9,141.55) | 0.35 (0.26 to 0.45) | 3.05 (4.05 to 2.24) | 2,124.38 (1,508.89 to 2,864.37) | 0.23 (0.17 to 0.28) |
| Female | 10.68 (9.41 to 12.17) | 15,109.87 (12,896.70 to 17,624.24) | 0.23 (0.18 to 0.30) | 5.67 (6.97 to 4.57) | 8,014.23 (6,094.07 to 10,362.68) | 0.37 (0.28 to 0.49) | 1.63 (2.17 to 1.18) | 2,303.18 (1,610.40 to 3,131.68) | 0.26 (0.19 to 0.33) |
| Male | 9.84 (8.88 to 10.95) | 13,517.73 (11,771.41 to 15,478.16) | 0.16 (0.11 to 0.20) | 4.56 (5.60 to 3.70) | 6,242.53 (4,767.59 to 7,976.38) | 0.32 (0.24 to 0.42) | 1.42 (1.88 to 1.04) | 1,951.09 (1,397.37 to 2,631.95) | 0.19 (0.14 to 0.25) |
| **South Sudan** | | | | | | | | | |
| Total | 4.42 (3.74 to 5.18) | 13,201.17 (10,915.17 to 15,953.28) | 0.07 (-0.02 to 0.16) | 1.89 (2.49 to 1.40) | 5,703.50 (4,008.36 to 7,955.91) | 0.11 (-0.05 to 0.30) | 0.62 (0.84 to 0.43) | 1,875.90 (1,283.20 to 2,592.47) | 0.08 (-0.03 to 0.21) |
| Female | 2.21 (1.82 to 2.66) | 13,534.48 (10,893.18 to 16,679.02) | 0.08 (-0.02 to 0.20) | 1.06 (1.42 to 0.76) | 6,475.82 (4,463.11 to 9,332.87) | 0.13 (-0.04 to 0.34) | 0.32 (0.44 to 0.22) | 1,992.63 (1,338.76 to 2,798.77) | 0.10 (-0.04 to 0.25) |
| Male | 2.21 (1.89 to 2.54) | 12,824.34 (10,698.72 to 15,282.47) | 0.05 (-0.03 to 0.14) | 0.84 (1.09 to 0.64) | 4,933.05 (3,516.31 to 6,738.35) | 0.09 (-0.05 to 0.27) | 0.30 (0.40 to 0.21) | 1,755.25 (1,211.75 to 2,406.30) | 0.06 (-0.04 to 0.18) |
| **Spain** | | | | | | | | | |
| Total | 16.91 (14.48 to 19.70) | 24,161.57 (20,327.34 to 28,633.57) | 0.11 (0.01 to 0.21) | 7.21 (9.31 to 5.50) | 10,288.26 (7,377.18 to 14,096.80) | 0.23 (0.03 to 0.49) | 2.26 (3.07 to 1.62) | 3,224.95 (2,264.04 to 4,416.05) | 0.16 (0.04 to 0.29) |
| Female | 9.11 (7.67 to 10.94) | 26,687.54 (21,832.44 to 32,450.02) | 0.14 (0.03 to 0.27) | 4.92 (6.47 to 3.73) | 14,407.26 (10,131.57 to 20,096.37) | 0.22 (-0.02 to 0.51) | 1.39 (1.92 to 0.97) | 4,056.22 (2,765.01 to 5,690.72) | 0.17 (0.02 to 0.34) |
| Male | 7.80 (6.73 to 9.04) | 21,753.94 (18,371.40 to 25,505.78) | 0.07 (-0.01 to 0.16) | 2.29 (2.96 to 1.73) | 6,371.31 (4,686.31 to 8,589.48) | 0.27 (0.07 to 0.50) | 0.87 (1.16 to 0.64) | 2,434.12 (1,740.28 to 3,280.45) | 0.14 (0.03 to 0.27) |
| **Sri Lanka** | | | | | | | | | |
| Total | 6.96 (5.91 to 8.23) | 13,283.49 (10,933.43 to 15,979.27) | 0.16 (0.05 to 0.30) | 3.06 (3.96 to 2.31) | 5,816.61 (4,211.55 to 7,903.25) | 0.34 (0.13 to 0.59) | 0.98 (1.33 to 0.69) | 1,857.87 (1,289.42 to 2,593.31) | 0.21 (0.07 to 0.37) |
| Female | 3.58 (2.94 to 4.36) | 13,677.02 (10,928.43 to 16,980.98) | 0.20 (0.06 to 0.38) | 1.67 (2.18 to 1.25) | 6,367.89 (4,537.24 to 8,851.59) | 0.37 (0.12 to 0.66) | 0.52 (0.73 to 0.35) | 1,975.47 (1,326.76 to 2,814.01) | 0.24 (0.07 to 0.44) |
| Male | 3.38 (2.91 to 3.90) | 12,885.63 (10,785.25 to 15,309.53) | 0.12 (0.03 to 0.22) | 1.39 (1.82 to 1.07) | 5,268.50 (3,788.31 to 7,116.72) | 0.31 (0.10 to 0.56) | 0.46 (0.62 to 0.33) | 1,740.38 (1,216.75 to 2,401.19) | 0.17 (0.04 to 0.33) |
| **Sudan** | | | | | | | | | |
| Total | 28.03 (23.78 to 33.84) | 19,566.55 (16,257.30 to 24,015.04) | 0.11 (0.01 to 0.24) | 11.75 (15.90 to 8.63) | 8,229.95 (5,643.25 to 11,720.80) | 0.21 (0.02 to 0.44) | 3.78 (5.19 to 2.70) | 2,649.79 (1,848.38 to 3,699.32) | 0.14 (0.02 to 0.29) |
| Female | 13.93 (11.59 to 17.53) | 20,118.81 (16,145.58 to 25,477.92) | 0.13 (0.00 to 0.28) | 6.26 (8.59 to 4.53) | 9,059.21 (6,159.31 to 13,006.69) | 0.22 (0.00 to 0.45) | 1.98 (2.77 to 1.39) | 2,867.60 (1,976.55 to 4,071.31) | 0.16 (0.00 to 0.32) |
| Male | 14.10 (12.17 to 16.63) | 19,040.24 (16,088.19 to 22,722.63) | 0.09 (0.00 to 0.19) | 5.49 (7.42 to 4.04) | 7,449.83 (5,129.53 to 10,547.46) | 0.20 (0.01 to 0.44) | 1.80 (2.44 to 1.29) | 2,444.35 (1,700.80 to 3,383.59) | 0.13 (0.02 to 0.27) |
| **Suriname** | | | | | | | | | |
| Total | 0.30 (0.25 to 0.35) | 20,783.95 (17,283.99 to 24,913.62) | 0.18 (0.09 to 0.30) | 0.14 (0.19 to 0.10) | 9,608.84 (6,838.36 to 13,603.22) | 0.48 (0.24 to 0.76) | 0.04 (0.05 to 0.03) | 2,643.09 (1,802.36 to 3,661.64) | 0.30 (0.16 to 0.47) |
| Female | 0.14 (0.12 to 0.17) | 20,797.09 (16,713.66 to 25,658.05) | 0.26 (0.13 to 0.41) | 0.09 (0.12 to 0.06) | 12,236.32 (8,510.75 to 17,505.93) | 0.52 (0.26 to 0.86) | 0.02 (0.03 to 0.01) | 3,076.49 (2,060.32 to 4,344.03) | 0.35 (0.18 to 0.55) |
| Male | 0.15 (0.13 to 0.18) | 20,737.05 (17,357.49 to 24,706.52) | 0.12 (0.05 to 0.21) | 0.05 (0.07 to 0.04) | 7,099.09 (5,058.43 to 9,827.06) | 0.42 (0.19 to 0.71) | 0.02 (0.02 to 0.01) | 2,227.32 (1,552.01 to 3,060.47) | 0.23 (0.11 to 0.39) |
| **Sweden** | | | | | | | | | |
| Total | 3.49 (3.03 to 3.99) | 19,659.77 (16,797.01 to 22,744.07) | 0.11 (0.02 to 0.20) | 1.74 (2.16 to 1.35) | 9,805.44 (7,273.38 to 12,737.30) | 0.19 (0.05 to 0.35) | 0.52 (0.68 to 0.37) | 2,896.43 (2,031.12 to 3,946.57) | 0.13 (0.03 to 0.24) |
| Female | 1.90 (1.62 to 2.20) | 22,235.22 (18,661.11 to 26,282.06) | 0.13 (0.03 to 0.24) | 1.20 (1.51 to 0.91) | 13,978.23 (10,189.32 to 18,346.00) | 0.20 (0.04 to 0.37) | 0.30 (0.41 to 0.21) | 3,562.00 (2,447.37 to 4,932.88) | 0.15 (0.04 to 0.28) |
| Male | 1.59 (1.38 to 1.80) | 17,284.01 (14,853.19 to 19,895.79) | 0.08 (0.01 to 0.16) | 0.55 (0.68 to 0.43) | 5,942.70 (4,501.73 to 7,655.49) | 0.18 (0.04 to 0.34) | 0.21 (0.27 to 0.15) | 2,281.25 (1,630.64 to 3,024.94) | 0.10 (0.01 to 0.20) |
| **Switzerland** | | | | | | | | | |
| Total | 2.93 (2.44 to 3.51) | 21,520.99 (17,488.32 to 26,394.39) | 0.08 (-0.05 to 0.22) | 1.11 (1.45 to 0.83) | 8,096.12 (5,829.32 to 10,952.15) | 0.24 (0.06 to 0.50) | 0.41 (0.56 to 0.28) | 2,995.34 (2,052.45 to 4,156.66) | 0.11 (-0.03 to 0.26) |
| Female | 1.56 (1.26 to 1.94) | 23,682.58 (18,638.92 to 29,860.47) | 0.10 (-0.07 to 0.26) | 0.67 (0.90 to 0.50) | 10,202.77 (7,238.16 to 14,120.45) | 0.26 (0.06 to 0.56) | 0.23 (0.33 to 0.16) | 3,527.92 (2,349.03 to 4,943.93) | 0.12 (-0.03 to 0.31) |
| Male | 1.37 (1.16 to 1.61) | 19,515.56 (16,127.02 to 23,363.70) | 0.06 (-0.04 to 0.18) | 0.43 (0.55 to 0.33) | 6,134.65 (4,481.24 to 8,168.83) | 0.22 (0.03 to 0.43) | 0.18 (0.24 to 0.13) | 2,499.99 (1,747.70 to 3,429.66) | 0.09 (-0.04 to 0.24) |
| **Syrian Arab Republic** | | | | | | | | | |
| Total | 9.21 (7.60 to 10.99) | 19,835.27 (16,221.82 to 24,232.11) | 0.13 (0.01 to 0.26) | 3.76 (5.15 to 2.68) | 8,096.94 (5,557.87 to 11,359.25) | 0.23 (0.04 to 0.44) | 1.26 (1.71 to 0.88) | 2,704.74 (1,842.50 to 3,786.86) | 0.17 (0.04 to 0.33) |
| Female | 5.03 (4.05 to 6.11) | 20,718.72 (16,457.52 to 25,839.85) | 0.16 (0.02 to 0.32) | 2.26 (3.14 to 1.57) | 9,297.39 (6,280.47 to 13,338.07) | 0.24 (0.04 to 0.47) | 0.73 (0.99 to 0.50) | 2,983.35 (1,989.26 to 4,207.01) | 0.20 (0.04 to 0.37) |
| Male | 4.17 (3.55 to 4.95) | 18,705.39 (15,625.29 to 22,253.47) | 0.09 (-0.01 to 0.20) | 1.49 (1.98 to 1.08) | 6,735.28 (4,680.92 to 9,325.69) | 0.21 (0.03 to 0.42) | 0.53 (0.72 to 0.37) | 2,376.81 (1,642.68 to 3,302.86) | 0.15 (0.02 to 0.32) |
| **Taiwan (Province of China)** | | | | | | | | | |
| Total | 4.05 (3.36 to 4.92) | 11,650.89 (9,501.45 to 14,335.25) | 0.04 (-0.06 to 0.16) | 1.08 (1.36 to 0.85) | 3,101.71 (2,300.40 to 4,040.82) | 0.07 (-0.07 to 0.22) | 0.53 (0.72 to 0.38) | 1,503.15 (1,040.23 to 2,088.08) | 0.04 (-0.07 to 0.17) |
| Female | 1.95 (1.56 to 2.45) | 11,579.24 (9,100.58 to 14,915.13) | 0.05 (-0.08 to 0.20) | 0.61 (0.80 to 0.47) | 3,631.03 (2,633.74 to 4,904.78) | 0.07 (-0.08 to 0.25) | 0.27 (0.38 to 0.19) | 1,590.94 (1,068.67 to 2,272.71) | 0.05 (-0.08 to 0.21) |
| Male | 2.10 (1.79 to 2.49) | 11,716.45 (9,710.50 to 14,124.81) | 0.03 (-0.05 to 0.13) | 0.47 (0.57 to 0.37) | 2,615.49 (1,960.54 to 3,390.96) | 0.06 (-0.05 to 0.18) | 0.26 (0.35 to 0.19) | 1,422.50 (999.15 to 1,939.50) | 0.03 (-0.07 to 0.16) |
| **Tajikistan** | | | | | | | | | |
| Total | 3.42 (2.93 to 3.98) | 12,332.09 (10,372.36 to 14,663.17) | 0.11 (0.03 to 0.21) | 1.26 (1.63 to 0.94) | 4,536.64 (3,206.49 to 6,195.20) | 0.30 (0.12 to 0.50) | 0.44 (0.59 to 0.31) | 1,569.78 (1,085.15 to 2,183.04) | 0.16 (0.04 to 0.29) |
| Female | 1.63 (1.38 to 1.94) | 12,088.88 (9,887.51 to 14,812.04) | 0.15 (0.04 to 0.28) | 0.71 (0.95 to 0.52) | 5,270.15 (3,678.94 to 7,402.38) | 0.33 (0.14 to 0.57) | 0.22 (0.31 to 0.16) | 1,657.59 (1,118.77 to 2,359.20) | 0.20 (0.06 to 0.35) |
| Male | 1.79 (1.55 to 2.05) | 12,556.26 (10,646.35 to 14,640.04) | 0.08 (0.02 to 0.15) | 0.55 (0.7 to 0.42) | 3,839.10 (2,765.14 to 5,168.32) | 0.26 (0.08 to 0.46) | 0.21 (0.28 to 0.15) | 1,485.89 (1,051.59 to 2,022.81) | 0.12 (0.02 to 0.24) |
| **Thailand** | | | | | | | | | |
| Total | 16.14 (13.79 to 19.03) | 13,718.50 (11,491.49 to 16,529.84) | 0.07 (0.00 to 0.18) | 5.22 (6.72 to 3.87) | 4,401.23 (3,146.24 to 5,908.89) | 0.19 (0.04 to 0.39) | 1.92 (2.61 to 1.38) | 1,609.54 (1,112.38 to 2,188.82) | 0.11 (0.01 to 0.24) |
| Female | 7.39 (6.17 to 8.97) | 12,591.62 (10,162.54 to 15,568.73) | 0.10 (-0.01 to 0.25) | 2.78 (3.73 to 2.03) | 4,730.21 (3,313.70 to 6,540.82) | 0.21 (0.04 to 0.42) | 0.98 (1.35 to 0.69) | 1,652.29 (1,111.91 to 2,299.10) | 0.13 (-0.00 to 0.30) |
| Male | 8.75 (7.49 to 10.26) | 14,806.22 (12,443.83 to 17,562.85) | 0.05 (-0.01 to 0.13) | 2.44 (3.11 to 1.83) | 4,077.94 (2,918.22 to 5,402.00) | 0.17 (0.01 to 0.36) | 0.94 (1.25 to 0.69) | 1,566.57 (1,106.98 to 2,118.87) | 0.09 (-0.02 to 0.21) |
| **Timor-Leste** | | | | | | | | | |
| Total | 0.59 (0.50 to 0.70) | 12,533.75 (10,303.56 to 15,005.94) | 0.11 (0.02 to 0.23) | 0.23 (0.30 to 0.17) | 4,815.11 (3,400.88 to 6,616.28) | 0.25 (0.08 to 0.46) | 0.08 (0.11 to 0.05) | 1,658.39 (1,134.21 to 2,263.00) | 0.15 (0.03 to 0.29) |
| Female | 0.30 (0.25 to 0.36) | 12,790.05 (10,298.45 to 15,758.47) | 0.14 (0.01 to 0.29) | 0.12 (0.16 to 0.09) | 5,165.91 (3,625.41 to 7,183.49) | 0.27 (0.08 to 0.53) | 0.04 (0.06 to 0.03) | 1,745.80 (1,169.33 to 2,474.15) | 0.18 (0.04 to 0.33) |
| Male | 0.30 (0.25 to 0.34) | 12,275.03 (10,255.23 to 14,468.29) | 0.09 (0.01 to 0.18) | 0.11 (0.14 to 0.08) | 4,478.46 (3,150.52 to 6,166.34) | 0.23 (0.06 to 0.45) | 0.04 (0.05 to 0.03) | 1,573.43 (1,076.08 to 2,148.71) | 0.13 (0.01 to 0.26) |
| **Togo** | | | | | | | | | |
| Total | 3.23 (2.75 to 3.77) | 12,402.20 (10,310.64 to 14,824.09) | 0.05 (-0.04 to 0.15) | 1.30 (1.7 to 0.95) | 5,021.81 (3,465.67 to 6,958.25) | 0.08 (-0.07 to 0.27) | 0.45 (0.59 to 0.31) | 1,724.62 (1,184.61 to 2,336.85) | 0.05 (-0.05 to 0.18) |
| Female | 1.60 (1.33 to 1.91) | 12,615.01 (10,162.96 to 15,571.75) | 0.06 (-0.05 to 0.18) | 0.72 (0.98 to 0.52) | 5,734.19 (3,812.38 to 8,279.36) | 0.09 (-0.09 to 0.30) | 0.23 (0.31 to 0.16) | 1,823.66 (1,224.56 to 2,534.76) | 0.06 (-0.06 to 0.21) |
| Male | 1.63 (1.41 to 1.87) | 12,190.26 (10,241.75 to 14,361.33) | 0.04 (-0.04 to 0.12) | 0.58 (0.74 to 0.44) | 4,338.70 (3,096.97 to 5,876.82) | 0.07 (-0.07 to 0.24) | 0.22 (0.28 to 0.16) | 1,628.96 (1,138.38 to 2,197.48) | 0.04 (-0.05 to 0.16) |
| **Tokelau** | | | | | | | | | |
| Total | 0.00 (0.00 to 0.00) | 13,370.21 (10,960.45 to 16,095.15) | 0.08 (-0.02 to 0.19) | 0.00 (0.00 to 0.00) | 4,920.22 (3,351.78 to 7,033.47) | 0.16 (0.00 to 0.37) | 0.00 (0.00 to 0.00) | 1,742.01 (1,176.41 to 2,420.69) | 0.11 (-0.02 to 0.25) |
| Female | 0.00 (0.00 to 0.00) | 13,285.21 (10,628.03 to 16,531.09) | 0.11 (-0.03 to 0.26) | 0.00 (0.00 to 0.00) | 5,248.88 (3,497.83 to 7,513.54) | 0.17 (-0.01 to 0.40) | 0.00 (0.00 to 0.00) | 1,823.83 (1,198.34 to 2,590.13) | 0.13 (-0.02 to 0.30) |
| Male | 0.00 (0.00 to 0.00) | 13,437.74 (11,164.03 to 15,923.78) | 0.06 (-0.01 to 0.16) | 0.00 (0.00 to 0.00) | 4,627.15 (3,180.23 to 6,627.37) | 0.15 (-0.01 to 0.33) | 0.00 (0.00 to 0.00) | 1,668.54 (1,146.08 to 2,297.68) | 0.09 (-0.02 to 0.21) |
| **Tonga** | | | | | | | | | |
| Total | 0.04 (0.03 to 0.05) | 13,250.08 (10,892.17 to 16,151.97) | 0.08 (-0.01 to 0.19) | 0.01 (0.02 to 0.01) | 4,383.21 (3,194.75 to 5,995.38) | 0.16 (-0.01 to 0.34) | 0.01 (0.01 to 0.00) | 1,676.24 (1,156.45 to 2,347.19) | 0.11 (-0.02 to 0.24) |
| Female | 0.02 (0.02 to 0.02) | 13,214.16 (10,502.13 to 16,642.63) | 0.11 (-0.02 to 0.24) | 0.01 (0.01 to 0.01) | 4,740.55 (3,408.54 to 6,570.64) | 0.17 (-0.01 to 0.36) | 0.00 (0.00 to 0.00) | 1,760.98 (1,182.69 to 2,497.96) | 0.13 (-0.02 to 0.28) |
| Male | 0.02 (0.02 to 0.02) | 13,267.87 (11,054.50 to 15,892.66) | 0.06 (-0.01 to 0.14) | 0.01 (0.01 to 0.00) | 4,043.13 (2,938.61 to 5,501.98) | 0.14 (-0.02 to 0.32) | 0.00 (0.00 to 0.00) | 1,594.63 (1,104.67 to 2,198.92) | 0.09 (-0.02 to 0.20) |
| **Trinidad and Tobago** | | | | | | | | | |
| Total | 0.54 (0.46 to 0.63) | 19,771.92 (16,371.21 to 23,602.77) | 0.17 (0.09 to 0.27) | 0.22 (0.29 to 0.16) | 8,021.37 (5,750.62 to 11,204.37) | 0.46 (0.23 to 0.75) | 0.07 (0.09 to 0.05) | 2,444.67 (1,692.32 to 3,381.47) | 0.27 (0.14 to 0.43) |
| Female | 0.26 (0.22 to 0.31) | 19,424.21 (15,770.65 to 23,838.28) | 0.24 (0.12 to 0.38) | 0.13 (0.18 to 0.10) | 9,899.18 (6,955.82 to 14,088.80) | 0.49 (0.23 to 0.83) | 0.04 (0.05 to 0.03) | 2,794.83 (1,888.19 to 3,914.62) | 0.32 (0.16 to 0.51) |
| Male | 0.28 (0.24 to 0.33) | 20,103.54 (16,689.70 to 24,104.53) | 0.11 (0.05 to 0.19) | 0.09 (0.11 to 0.07) | 6,205.72 (4,504.00 to 8,518.12) | 0.40 (0.19 to 0.69) | 0.03 (0.04 to 0.02) | 2,105.92 (1,487.75 to 2,915.07) | 0.22 (0.10 to 0.37) |
| **Tunisia** | | | | | | | | | |
| Total | 5.53 (4.61 to 6.61) | 22,051.30 (17,904.14 to 26,918.47) | 0.24 (0.11 to 0.40) | 2.88 (3.98 to 2.08) | 11,496.61 (7,818.36 to 16,502.26) | 0.45 (0.21 to 0.71) | 0.82 (1.14 to 0.55) | 3,281.90 (2,167.45 to 4,624.61) | 0.31 (0.15 to 0.49) |
| Female | 2.89 (2.34 to 3.52) | 23,583.61 (18,545.56 to 29,379.77) | 0.29 (0.13 to 0.47) | 1.61 (2.26 to 1.14) | 13,151.45 (8,769.67 to 19,327.81) | 0.47 (0.22 to 0.74) | 0.45 (0.63 to 0.30) | 3,669.11 (2,378.19 to 5,268.08) | 0.34 (0.16 to 0.54) |
| Male | 2.65 (2.26 to 3.12) | 20,556.92 (17,164.18 to 24,795.56) | 0.20 (0.08 to 0.34) | 1.27 (1.71 to 0.93) | 9,916.71 (6,823.61 to 13,918.44) | 0.42 (0.17 to 0.69) | 0.37 (0.51 to 0.25) | 2,908.94 (1,907.86 to 4,040.62) | 0.27 (0.12 to 0.44) |
| **Turkey** | | | | | | | | | |
| Total | 36.50 (30.81 to 43.61) | 18,913.70 (15,630.39 to 22,983.15) | 0.16 (0.05 to 0.29) | 17.03 (22.54 to 12.27) | 8,812.60 (6,024.04 to 12,288.56) | 0.32 (0.10 to 0.54) | 5.23 (7.19 to 3.56) | 2,695.66 (1,812.04 to 3,755.58) | 0.21 (0.07 to 0.36) |
| Female | 19.19 (15.74 to 23.26) | 20,330.68 (16,258.27 to 25,259.45) | 0.20 (0.07 to 0.36) | 10.27 (13.89 to 7.22) | 10,894.33 (7,186.10 to 15,623.85) | 0.33 (0.10 to 0.61) | 2.94 (4.13 to 1.97) | 3,099.20 (2,029.76 to 4,429.06) | 0.23 (0.08 to 0.41) |
| Male | 17.31 (14.76 to 20.59) | 17,563.87 (14,811.52 to 21,118.54) | 0.12 (0.03 to 0.23) | 6.76 (8.67 to 5.00) | 6,833.83 (4,863.33 to 9,313.94) | 0.30 (0.09 to 0.55) | 2.29 (3.08 to 1.60) | 2,311.75 (1,587.02 to 3,184.30) | 0.17 (0.05 to 0.32) |
| **Turkmenistan** | | | | | | | | | |
| Total | 1.60 (1.38 to 1.86) | 11,996.39 (10,077.09 to 14,257.56) | 0.11 (0.03 to 0.19) | 0.66 (0.90 to 0.50) | 4,965.74 (3,549.24 to 6,939.96) | 0.25 (0.06 to 0.48) | 0.22 (0.29 to 0.15) | 1,608.36 (1,104.49 to 2,212.65) | 0.14 (0.04 to 0.27) |
| Female | 0.75 (0.62 to 0.89) | 11,702.22 (9,571.18 to 14,281.73) | 0.14 (0.04 to 0.25) | 0.35 (0.49 to 0.25) | 5,493.78 (3,801.69 to 7,851.67) | 0.28 (0.05 to 0.53) | 0.11 (0.15 to 0.07) | 1,677.79 (1,130.99 to 2,348.33) | 0.17 (0.04 to 0.33) |
| Male | 0.86 (0.74 to 0.98) | 12,276.20 (10,400.81 to 14,368.39) | 0.08 (0.02 to 0.15) | 0.31 (0.41 to 0.24) | 4,488.53 (3,254.00 to 6,199.84) | 0.23 (0.04 to 0.43) | 0.11 (0.14 to 0.08) | 1,546.42 (1,076.36 to 2,084.28) | 0.11 (0.01 to 0.24) |
| **Tuvalu** | | | | | | | | | |
| Total | 0.00 (0.00 to 0.01) | 13,644.67 (11,226.14 to 16,581.17) | 0.08 (-0.01 to 0.20) | 0.00 (0.00 to 0.01) | 4,864.91 (3,336.51 to 6,935.81) | 0.16 (0.01 to 0.34) | 0.00 (0.00 to 0.00) | 1,735.41 (1,175.82 to 2,401.84) | 0.11 (-0.01 to 0.25) |
| Female | 0.00 (0.00 to 0.00) | 13,543.63 (10,773.09 to 17,053.58) | 0.11 (-0.01 to 0.27) | 0.00 (0.00 to 0.00) | 5,190.47 (3,528.78 to 7,443.74) | 0.18 (0.00 to 0.38) | 0.00 (0.00 to 0.00) | 1,815.64 (1,178.75 to 2,554.06) | 0.13 (-0.02 to 0.30) |
| Male | 0.00 (0.00 to 0.00) | 13,734.24 (11,455.08 to 16,291.10) | 0.06 (-0.01 to 0.15) | 0.00 (0.00 to 0.00) | 4,577.43 (3,123.07 to 6,549.42) | 0.15 (-0.01 to 0.35) | 0.00 (0.00 to 0.00) | 1,664.51 (1,139.12 to 2,286.01) | 0.09 (-0.02 to 0.22) |
| **Uganda** | | | | | | | | | |
| Total | 22.16 (18.77 to 26.22) | 14,802.97 (12,188.20 to 17,967.05) | 0.13 (0.03 to 0.24) | 12.55 (17.06 to 8.89) | 8,447.56 (5,656.99 to 12,077.37) | 0.24 (0.04 to 0.49) | 3.35 (4.70 to 2.31) | 2,255.19 (1,520.09 to 3,186.68) | 0.16 (0.04 to 0.31) |
| Female | 11.15 (9.24 to 13.44) | 14,951.99 (11,971.39 to 18,530.31) | 0.15 (0.04 to 0.29) | 6.88 (9.47 to 4.83) | 9,263.93 (6,149.85 to 13,526.92) | 0.26 (0.05 to 0.52) | 1.74 (2.47 to 1.18) | 2,348.77 (1,543.51 to 3,391.49) | 0.18 (0.04 to 0.36) |
| Male | 11.01 (9.48 to 12.85) | 14,625.47 (12,236.88 to 17,528.73) | 0.10 (0.01 to 0.20) | 5.66 (7.68 to 4.05) | 7,621.89 (5,121.48 to 10,776.36) | 0.22 (0.01 to 0.47) | 1.61 (2.20 to 1.12) | 2,158.03 (1,485.96 to 3,020.56) | 0.14 (0.01 to 0.27) |
| **Ukraine** | | | | | | | | | |
| Total | 9.39 (8.16 to 10.85) | 14,303.70 (12,194.94 to 16,715.28) | 0.17 (0.08 to 0.27) | 4.02 (5.16 to 3.09) | 6,162.32 (4,393.11 to 8,276.43) | 0.36 (0.18 to 0.59) | 1.27 (1.73 to 0.91) | 1,935.86 (1,358.21 to 2,674.60) | 0.22 (0.11 to 0.36) |
| Female | 4.57 (3.90 to 5.37) | 14,350.37 (11,991.00 to 17,219.45) | 0.21 (0.09 to 0.35) | 2.14 (2.78 to 1.63) | 6,748.09 (4,804.46 to 9,025.95) | 0.38 (0.19 to 0.62) | 0.65 (0.90 to 0.46) | 2,051.28 (1,413.62 to 2,883.75) | 0.25 (0.11 to 0.42) |
| Male | 4.82 (4.23 to 5.48) | 14,257.69 (12,264.17 to 16,558.04) | 0.13 (0.06 to 0.22) | 1.89 (2.43 to 1.45) | 5,608.26 (4,009.59 to 7,518.94) | 0.33 (0.15 to 0.59) | 0.62 (0.83 to 0.44) | 1,826.61 (1,280.16 to 2,495.78) | 0.18 (0.08 to 0.33) |
| **United Arab Emirates** | | | | | | | | | |
| Total | 1.86 (1.55 to 2.25) | 16,699.04 (13,440.13 to 20,711.46) | 0.13 (0.00 to 0.27) | 0.86 (1.15 to 0.63) | 7,705.83 (5,287.45 to 10,816.45) | 0.23 (0.04 to 0.43) | 0.28 (0.38 to 0.19) | 2,533.70 (1,700.61 to 3,500.55) | 0.15 (0.01 to 0.29) |
| Female | 0.96 (0.78 to 1.18) | 17,943.52 (14,012.98 to 22,829.73) | 0.15 (0.01 to 0.31) | 0.46 (0.62 to 0.33) | 8,523.57 (5,784.67 to 12,292.91) | 0.23 (0.05 to 0.46) | 0.15 (0.21 to 0.10) | 2,774.93 (1,814.37 to 3,900.65) | 0.16 (0.01 to 0.32) |
| Male | 0.90 (0.76 to 1.06) | 15,560.80 (12,829.39 to 18,866.46) | 0.11 (0.00 to 0.23) | 0.40 (0.53 to 0.30) | 6,950.06 (4,822.72 to 9,704.65) | 0.22 (0.03 to 0.42) | 0.13 (0.18 to 0.09) | 2,311.91 (1,570.79 to 3,166.12) | 0.13 (0.00 to 0.26) |
| **United Kingdom** | | | | | | | | | |
| Total | 23.70 (21.18 to 26.46) | 19,551.77 (16,929.30 to 22,312.34) | 0.15 (0.13 to 0.17) | 11.15 (13.92 to 9.00) | 9,152.96 (6,891.37 to 11,920.83) | 0.31 (0.27 to 0.35) | 3.44 (4.57 to 2.45) | 2,817.93 (1,986.31 to 3,790.56) | 0.19 (0.16 to 0.22) |
| Female | 12.46 (11.04 to 14.07) | 20,830.66 (17,891.94 to 23,976.27) | 0.18 (0.16 to 0.20) | 6.62 (8.31 to 5.29) | 11,039.50 (8,295.43 to 14,520.50) | 0.32 (0.29 to 0.36) | 1.93 (2.57 to 1.37) | 3,204.82 (2,236.89 to 4,311.76) | 0.21 (0.18 to 0.24) |
| Male | 11.24 (10.00 to 12.55) | 18,301.24 (15,900.30 to 20,819.83) | 0.11 (0.10 to 0.14) | 4.52 (5.60 to 3.69) | 7,324.05 (5,520.89 to 9,447.81) | 0.29 (0.25 to 0.34) | 1.51 (1.99 to 1.09) | 2,441.96 (1,733.60 to 3,251.95) | 0.16 (0.13 to 0.19) |
| **United Republic of Tanzania** | | | | | | | | | |
| Total | 25.06 (21.15 to 29.52) | 13,101.50 (10,743.13 to 15,818.55) | 0.09 (-0.01 to 0.21) | 11.27 (14.89 to 8.40) | 5,915.50 (4,072.96 to 8,322.82) | 0.17 (-0.01 to 0.36) | 3.59 (4.83 to 2.54) | 1,884.72 (1,306.51 to 2,593.91) | 0.11 (-0.01 to 0.24) |
| Female | 13.14 (10.88 to 15.83) | 13,372.11 (10,682.01 to 16,546.11) | 0.11 (0.00 to 0.25) | 6.51 (8.92 to 4.68) | 6,633.06 (4,465.55 to 9,371.79) | 0.19 (-0.01 to 0.41) | 1.94 (2.65 to 1.35) | 1,984.00 (1,344.00 to 2,778.28) | 0.13 (-0.01 to 0.30) |
| Male | 11.91 (10.28 to 13.79) | 12,778.36 (10,621.53 to 15,195.85) | 0.07 (-0.01 to 0.17) | 4.76 (6.25 to 3.62) | 5,139.27 (3,611.36 to 7,049.17) | 0.16 (0.00 to 0.35) | 1.64 (2.24 to 1.17) | 1,774.64 (1,235.24 to 2,438.72) | 0.09 (-0.02 to 0.22) |
| **United States of America** | | | | | | | | | |
| Total | 144.66 (130.83 to 159.83) | 22,093.38 (19,456.88 to 24,942.71) | 0.15 (0.12 to 0.18) | 83.67 (99.86 to 69.33) | 12,770.84 (10,107.28 to 15,920.96) | 0.34 (0.29 to 0.39) | 21.37 (28.20 to 15.52) | 3,238.46 (2,319.50 to 4,282.42) | 0.22 (0.18 to 0.25) |
| Female | 74.98 (67.57 to 83.06) | 23,281.57 (20,448.77 to 26,356.23) | 0.20 (0.17 to 0.24) | 53.72 (64.36 to 44.36) | 16,780.67 (13,227.81 to 21,042.46) | 0.35 (0.30 to 0.40) | 12.43 (16.56 to 8.92) | 3,844.91 (2,718.86 to 5,140.81) | 0.25 (0.21 to 0.29) |
| Male | 69.68 (61.90 to 78.64) | 20,952.27 (18,233.83 to 24,021.78) | 0.10 (0.06 to 0.14) | 29.95 (35.97 to 24.76) | 8,927.97 (7,062.75 to 11,102.42) | 0.31 (0.26 to 0.37) | 8.93 (11.65 to 6.52) | 2,656.96 (1,928.34 to 3,496.74) | 0.17 (0.13 to 0.21) |
| **United States Virgin Islands** | | | | | | | | | |
| Total | 0.03 (0.02 to 0.03) | 18,454.53 (15,416.91 to 22,052.95) | 0.11 (0.03 to 0.20) | 0.01 (0.01 to 0.01) | 6,026.65 (4,332.90 to 8,233.86) | 0.26 (0.08 to 0.44) | 0.00 (0.00 to 0.00) | 2,170.54 (1,494.21 to 2,997.29) | 0.16 (0.05 to 0.28) |
| Female | 0.01 (0.01 to 0.01) | 17,718.51 (14,534.59 to 21,758.24) | 0.15 (0.04 to 0.27) | 0.01 (0.01 to 0.00) | 7,340.58 (5,136.92 to 10,352.31) | 0.28 (0.08 to 0.49) | 0.00 (0.00 to 0.00) | 2,438.84 (1,629.13 to 3,391.74) | 0.19 (0.05 to 0.33) |
| Male | 0.01 (0.01 to 0.02) | 19,160.82 (15,892.75 to 23,198.92) | 0.07 (0.02 to 0.13) | 0.00 (0.00 to 0.00) | 4,717.51 (3,482.12 to 6,308.26) | 0.22 (0.07 to 0.41) | 0.00 (0.00 to 0.00) | 1,902.07 (1,339.56 to 2,605.74) | 0.13 (0.03 to 0.24) |
| **Uruguay** | | | | | | | | | |
| Total | 1.39 (1.18 to 1.64) | 19,144.68 (15,871.14 to 23,058.64) | 0.18 (0.07 to 0.31) | 0.61 (0.81 to 0.46) | 8,390.50 (5,916.01 to 11,559.13) | 0.37 (0.15 to 0.61) | 0.20 (0.28 to 0.14) | 2,754.90 (1,890.91 to 3,817.88) | 0.23 (0.09 to 0.38) |
| Female | 0.76 (0.63 to 0.92) | 21,180.88 (16,860.60 to 26,255.94) | 0.23 (0.09 to 0.38) | 0.39 (0.53 to 0.29) | 10,911.69 (7,647.84 to 15,338.80) | 0.39 (0.15 to 0.67) | 0.12 (0.16 to 0.08) | 3,270.51 (2,205.98 to 4,634.95) | 0.27 (0.11 to 0.45) |
| Male | 0.63 (0.54 to 0.72) | 17,154.50 (14,492.58 to 20,228.21) | 0.13 (0.04 to 0.23) | 0.22 (0.29 to 0.17) | 5,944.75 (4,273.80 to 8,028.81) | 0.33 (0.12 to 0.58) | 0.08 (0.11 to 0.06) | 2,253.13 (1,586.17 to 3,065.18) | 0.18 (0.04 to 0.31) |
| **Uzbekistan** | | | | | | | | | |
| Total | 9.44 (8.18 to 10.83) | 11,790.59 (9,987.24 to 13,817.56) | 0.08 (0.01 to 0.16) | 3.87 (5.31 to 2.90) | 4,825.75 (3,449.61 to 6,757.27) | 0.20 (0.02 to 0.4) | 1.24 (1.68 to 0.91) | 1,548.25 (1,091.65 to 2,126.15) | 0.11 (0.01 to 0.23) |
| Female | 4.42 (3.77 to 5.20) | 11,300.52 (9,384.37 to 13,591.08) | 0.10 (0.02 to 0.21) | 2.05 (2.92 to 1.52) | 5,243.84 (3,680.16 to 7,514.74) | 0.21 (0.02 to 0.44) | 0.62 (0.84 to 0.45) | 1,578.12 (1,098.45 to 2,209.94) | 0.13 (0.01 to 0.26) |
| Male | 5.02 (4.39 to 5.69) | 12,250.98 (10,441.26 to 14,210.83) | 0.06 (0.00 to 0.13) | 1.82 (2.40 to 1.39) | 4,424.89 (3,177.97 to 6,072.39) | 0.18 (0.01 to 0.39) | 0.62 (0.83 to 0.46) | 1,519.10 (1,072.08 to 2,080.06) | 0.09 (-0.01 to 0.22) |
| **Vanuatu** | | | | | | | | | |
| Total | 0.13 (0.11 to 0.15) | 13,724.94 (11,244.44 to 16,710.42) | 0.09 (-0.01 to 0.20) | 0.05 (0.06 to 0.03) | 4,864.72 (3,459.55 to 6,725.07) | 0.16 (-0.01 to 0.36) | 0.02 (0.02 to 0.01) | 1,739.09 (1,193.59 to 2,402.04) | 0.11 (-0.00 to 0.25) |
| Female | 0.06 (0.05 to 0.08) | 13,552.99 (10,798.93 to 16,963.18) | 0.11 (-0.02 to 0.26) | 0.02 (0.03 to 0.02) | 5,090.29 (3,582.54 to 7,145.37) | 0.17 (-0.02 to 0.39) | 0.01 (0.01 to 0.01) | 1,802.95 (1,204.71 to 2,541.91) | 0.13 (-0.02 to 0.30) |
| Male | 0.07 (0.06 to 0.08) | 13,871.73 (11,576.57 to 16,493.80) | 0.06 (-0.01 to 0.15) | 0.02 (0.03 to 0.02) | 4,650.35 (3,304.11 to 6,438.32) | 0.15 (-0.01 to 0.38) | 0.01 (0.01 to 0.01) | 1,676.67 (1,170.22 to 2,292.31) | 0.10 (-0.01 to 0.23) |
| **Venezuela (Bolivarian Republic of)** | | | | | | | | | |
| Total | 9.76 (8.13 to 11.43) | 16,500.61 (13,614.57 to 19,711.08) | 0.05 (-0.02 to 0.14) | 2.99 (3.83 to 2.31) | 5,115.16 (3,738.12 to 6,880.40) | 0.12 (-0.04 to 0.3) | 1.13 (1.52 to 0.81) | 1,938.50 (1,339.76 to 2,644.23) | 0.07 (-0.03 to 0.20) |
| Female | 4.77 (3.95 to 5.66) | 15,925.14 (12,987.24 to 19,477.80) | 0.07 (-0.02 to 0.18) | 1.71 (2.26 to 1.30) | 5,737.48 (4,095.30 to 7,894.73) | 0.13 (-0.04 to 0.33) | 0.61 (0.83 to 0.43) | 2,066.55 (1,391.50 to 2,889.20) | 0.08 (-0.04 to 0.23) |
| Male | 5.00 (4.19 to 5.88) | 17,034.09 (14,213.99 to 20,164.11) | 0.04 (-0.01 to 0.11) | 1.28 (1.59 to 1.00) | 4,454.35 (3,307.69 to 5,786.76) | 0.10 (-0.06 to 0.28) | 0.52 (0.68 to 0.37) | 1,798.88 (1,274.74 to 2,416.51) | 0.06 (-0.04 to 0.16) |
| **VietNam** | | | | | | | | | |
| Total | 22.08 (19.18 to 25.62) | 10,407.54 (8,790.78 to 12,327.46) | 0.09 (0.02 to 0.19) | 8.06 (10.61 to 6.03) | 3,802.53 (2,709.88 to 5,195.40) | 0.25 (0.06 to 0.44) | 2.91 (3.96 to 2.07) | 1,375.55 (956.93 to 1,881.73) | 0.12 (0.01 to 0.25) |
| Female | 11.05 (9.37 to 13.25) | 10,783.89 (8,880.37 to 13,182.76) | 0.12 (0.02 to 0.26) | 4.66 (6.31 to 3.40) | 4,551.60 (3,173.11 to 6,374.38) | 0.29 (0.07 to 0.5) | 1.51 (2.09 to 1.06) | 1,480.02 (997.96 to 2,063.73) | 0.16 (0.02 to 0.32) |
| Male | 11.03 (9.62 to 12.64) | 10,049.13 (8,597.31 to 11,763.80) | 0.07 (0.00 to 0.13) | 3.40 (4.40 to 2.60) | 3,098.90 (2,232.09 to 4,143.09) | 0.20 (0.05 to 0.38) | 1.40 (1.85 to 1.00) | 1,277.02 (896.29 to 1,722.58) | 0.09 (-0.02 to 0.19) |
| **Yemen** | | | | | | | | | |
| Total | 19.95 (16.73 to 23.64) | 18,271.18 (15,054.06 to 22,150.27) | 0.04 (-0.06 to 0.15) | 8.22 (11.03 to 6.05) | 7,608.18 (5,181.14 to 10,874.45) | 0.04 (-0.11 to 0.21) | 2.65 (3.61 to 1.85) | 2,454.51 (1,693.37 to 3,391.01) | 0.04 (-0.07 to 0.17) |
| Female | 10.09 (8.29 to 12.14) | 18,922.51 (15,164.38 to 23,323.00) | 0.04 (-0.07 to 0.18) | 4.74 (6.48 to 3.39) | 8,953.09 (6,000.38 to 13,141.49) | 0.04 (-0.13 to 0.23) | 1.43 (1.97 to 0.98) | 2,695.65 (1,820.36 to 3,756.00) | 0.04 (-0.08 to 0.18) |
| Male | 9.86 (8.38 to 11.52) | 17,614.86 (14,708.25 to 20,946.18) | 0.04 (-0.05 to 0.14) | 3.48 (4.53 to 2.63) | 6,295.07 (4,367.87 to 8,793.67) | 0.04 (-0.11 to 0.21) | 1.23 (1.64 to 0.87) | 2,217.54 (1,562.50 to 3,054.60) | 0.04 (-0.07 to 0.17) |
| **Zambia** | | | | | | | | | |
| Total | 8.73 (7.33 to 10.36) | 13,336.65 (10,960.05 to 16,271.16) | 0.14 (0.03 to 0.25) | 3.76 (5.02 to 2.75) | 5,753.62 (4,070.07 to 7,938.24) | 0.25 (0.08 to 0.45) | 1.24 (1.67 to 0.87) | 1,900.55 (1,317.32 to 2,628.65) | 0.16 (0.04 to 0.29) |
| Female | 4.53 (3.70 to 5.52) | 13,559.25 (10,854.36 to 16,929.36) | 0.16 (0.03 to 0.30) | 2.08 (2.77 to 1.47) | 6,222.88 (4,288.34 to 8,747.16) | 0.27 (0.08 to 0.50) | 0.66 (0.91 to 0.45) | 1,981.35 (1,349.21 to 2,782.14) | 0.18 (0.05 to 0.34) |
| Male | 4.21 (3.60 to 4.87) | 13,085.90 (10,934.63 to 15,643.22) | 0.11 (0.02 to 0.22) | 1.68 (2.21 to 1.26) | 5,258.54 (3,730.87 to 7,273.29) | 0.23 (0.05 to 0.43) | 0.58 (0.78 to 0.42) | 1,814.10 (1,277.16 to 2,488.34) | 0.13 (0.01 to 0.26) |
| **Zimbabwe** | | | | | | | | | |
| Total | 6.28 (5.29 to 7.47) | 12,352.00 (10,156.37 to 15,048.56) | 0.13 (0.03 to 0.24) | 2.32 (3.04 to 1.74) | 4,584.77 (3,229.12 to 6,252.82) | 0.24 (0.08 to 0.43) | 0.86 (1.18 to 0.61) | 1,705.99 (1,178.26 to 2,338.85) | 0.15 (0.04 to 0.28) |
| Female | 3.15 (2.61 to 3.82) | 12,171.75 (9,783.95 to 15,111.44) | 0.16 (0.03 to 0.29) | 1.28 (1.68 to 0.93) | 4,958.44 (3,455.34 to 6,957.82) | 0.26 (0.08 to 0.47) | 0.45 (0.62 to 0.31) | 1,735.19 (1,181.98 to 2,426.04) | 0.17 (0.03 to 0.34) |
| Male | 3.13 (2.68 to 3.66) | 12,527.75 (10,499.89 to 15,042.89) | 0.10 (0.02 to 0.20) | 1.04 (1.33 to 0.80) | 4,192.51 (2,986.53 to 5,691.98) | 0.21 (0.06 to 0.40) | 0.42 (0.56 to 0.30) | 1,674.29 (1,171.00 to 2,304.55) | 0.13 (0.01 to 0.25) |

* All results are rounded to two decimal places using the rounding half up method.

** 0.00 means that the number of people who suffered from mental disorders is less than 1,000 under this order of magnitude (100,000).

**Table S24.** Prevalence, Incidence, and Years Lived with Disability of Mental Disorders Among Adolescents and Young Adults (**Aged 10-14**) in 2021, and Percentage Change from 2019 to 2021 According to GBD.

|  | Prevalence (95% uncertainty interval) | | | Incidence (95% uncertainty interval) | | | Years lived with disability (95% uncertainty interval) | | |
| --- | --- | --- | --- | --- | --- | --- | --- | --- | --- |
| Cause | Counts in million | Rates per 100,000 people | Percentage rate change (2019-2021) | Counts in million | Rates per 100,000 people | Percentage rate change (2019-2021) | Counts in million | Rates per 100,000 people | Percentage rate change (2019-2021) |
| Mental disorders | | | | | | | | | |
| Total | 88.78 (75.27 to 102.79) | 13,316.91(11,290.45 to 15,418.93) | 0.07 (0.05 to 0.09) | 29.07 (22.34 to 36.09) | 4,361.27 (3,351.53 to 5,414.35) | 0.17 (0.14 to 0.20) | 9.80 (6.85 to 13.40) | 1,469.96 (1,027.23 to 2,010.78) | 0.11 (0.09 to 0.14) |
| Female | 40.45 (34.21 to 47.61) | 12,527.45(10,594.86 to 14,744.53) | 0.10 (0.08 to 0.12) | 15.56 (11.82 to 19.66) | 4,819.01 (3,660.77 to 6,087.34) | 0.20 (0.17 to 0.24) | 4.83 (3.34 to 6.66) | 1,497.02 (1,032.89 to 2,062.69) | 0.14 (0.11 to 0.17) |
| Male | 48.32 (41.38 to 55.92) | 14,058.64(12,039.66 to 16,270.74) | 0.05 (0.03 to 0.07) | 13.51 (10.49 to 16.63) | 3,931.22 (3,052.75 to 4,837.18) | 0.14 (0.11 to 0.17) | 4.97 (3.48 to 6.69) | 1,444.54 (1,012.70 to 1,945.95) | 0.08 (0.06 to 0.11) |
| Schizophrenia | | | | | | | | | |
| Total | 0.06 (0.03 to 0.10) | 8.60 (4.45 to 14.69) | 0.01 (-0.01 to 0.02) | 0.03 (0.02 to 0.06) | 5.22 (2.70 to 8.93) | 0.01 (-0.01 to 0.02) | 0.04 (0.02 to 0.07) | 6.09 (3.13 to 10.60) | 0.01 (-0.01 to 0.03) |
| Female | 0.03 (0.01 to 0.04) | 7.95 (4.06 to 13.72) | 0.01 (0.00 to 0.02) | 0.02 (0.01 to 0.03) | 4.83 (2.47 to 8.34) | 0.01 (0.00 to 0.02) | 0.02 (0.01 to 0.03) | 5.63 (2.85 to 9.92) | 0.01 (-0.02 to 0.03) |
| Male | 0.03 (0.02 to 0.05) | 9.21 (4.82 to 15.60) | 0.00 (-0.01 to 0.02) | 0.02 (0.01 to 0.03) | 5.59 (2.93 to 9.47) | 0.00 (-0.01 to 0.02) | 0.02 (0.01 to 0.04) | 6.52 (3.39 to 11.30) | 0.00 (-0.02 to 0.03) |
| Depressive disorders | | | | | | | | | |
| Total | 8.43 (5.58 to 11.83) | 1,264.95 (837.50 to 1,774.73) | 0.25 (0.20 to 0.29) | 13.42 (8.55 to 19.70) | 2,013.71 (1,282.04 to 2,954.95) | 0.30 (0.27 to 0.32) | 1.63 (0.96 to 2.50) | 244.44 (143.73 to 374.30) | 0.28 (0.24 to 0.31) |
| Female | 5.20 (3.47 to 7.27) | 1,608.79 (1,075.54 to 2,250.10) | 0.25 (0.21 to 0.29) | 8.39 (5.39 to 12.28) | 2,597.25 (1,669.85 to 3,803.43) | 0.30 (0.27 to 0.33) | 1.01 (0.59 to 1.54) | 312.17 (183.84 to 476.40) | 0.28 (0.24 to 0.32) |
| Male | 3.24 (2.14 to 4.53) | 941.89 (623.01 to 1,319.13) | 0.24 (0.20 to 0.28) | 5.04 (3.15 to 7.40) | 1,465.45 (915.41 to 2,151.87) | 0.29 (0.26 to 0.32) | 0.62 (0.36 to 0.96) | 180.81 (105.47 to 278.61) | 0.27 (0.22 to 0.32) |
| Bipolar disorder | | | | | | | | | |
| Total | 0.66 (0.45 to 0.95) | 99.71 (68.24 to 142.10) | 0.00 (0.00 to 0.00) | 0.41 (0.28 to 0.58) | 61.45 (41.79 to 87.48) | 0.00 (0.00 to 0.00) | 0.15 (0.08 to 0.24) | 22.53 (12.73 to 36.14) | 0.00 (-0.05 to 0.04) |
| Female | 0.34 (0.23 to 0.48) | 104.11 (70.88 to 149.16) | 0.00 (0.00 to 0.00) | 0.21 (0.14 to 0.30) | 64.16 (43.42 to 91.64) | 0.00 (0.00 to 0.00) | 0.08 (0.04 to 0.12) | 23.46 (13.00 to 37.99) | 0.00 (-0.06 to 0.06) |
| Male | 0.33 (0.26 to 0.46) | 95.57 (65.74 to 135.17) | 0.00 (0.00 to 0.00) | 0.20 (0.14 to 0.29) | 58.91 (40.26 to 83.43) | 0.00 (0.00 to 0.00) | 0.07 (0.04 to 0.12) | 21.66 (12.33 to 34.46) | 0.00 (-0.07 to 0.06) |
| Anxiety disorders | | | | | | | | | |
| Total | 27.08 (18.73 to 37.52) | 4,061.65 (2,809.75 to 5,627.88) | 0.23 (0.20 to 0.26) | 6.17 (4.69 to 8.09) | 925.07 (704.08 to 1,213.41) | 0.24 (0.21 to 0.27) | 3.36 (2.01 to 5.07) | 503.74 (302.03 to 760.86) | 0.23 (0.20 to 0.26) |
| Female | 16.26 (11.20 to 22.49) | 5,034.75 (3,469.35 to 6,965.67) | 0.24 (0.21 to 0.26) | 3.58 (2.73 to 4.70) | 1,107.98 (846.66 to 1,454.45) | 0.24 (0.21 to 0.27) | 2.01 (1.21 to 3.02) | 622.99 (373.84 to 936.54) | 0.23 (0.20 to 0.27) |
| Male | 10.82 (7.43 to 15.08) | 3,147.39 (2,162.95 to 4,387.49) | 0.23 (0.20 to 0.26) | 2.59 (1.96 to 3.43) | 753.22 (569.77 to 998.24) | 0.23 (0.21 to 0.26) | 1.35 (0.80 to 2.05) | 391.70 (233.80 to 596.03) | 0.23 (0.19 to 0.26) |
| Eating disorders | | | | | | | | | |
| Total | 0.77 (0.51 to 1.20) | 115.11 (76.32 to 179.73) | 0.00 (-0.01 to 0.01) | 1.43 (0.81 to 2.40) | 214.30 (120.91 to 360.45) | 0.00 (0.00 to 0.01) | 0.17 (0.09 to 0.29) | 24.88 (14.24 to 43.90) | 0.00 (-0.03 to 0.04) |
| Female | 0.47 (0.32 to 0.71) | 144.22 (97.75 to 220.03) | 0.00 (-0.01 to 0.01) | 0.63 (0.38 to 1.01) | 194.80 (117.13 to 312.98) | 0.00 (-0.01 to 0.01) | 0.10 (0.06 to 0.17) | 31.12 (18.19 to 53.69) | 0.00 (-0.04 to 0.04) |
| Male | 0.30 (0.19 to 0.49) | 87.77 (55.71 to 142.63) | 0.01 (0.00 to 0.01) | 0.80 (0.42 to 1.40) | 232.61 (122.89 to 406.97) | 0.01 (0.00 to 0.01) | 0.07 (0.04 to 0.12) | 19.02 (10.51 to 34.60) | 0.01 (-0.05 to 0.07) |
| Autism spectrum disorders | | | | | | | | | |
| Total | 5.61 (4.73 to 6.58) | 840.95 (709.37 to 987.31) | 0.00 (0.00 to 0.01) | 0.00 (0.00 to 0.00) | 0.00 (0.00 to 0.00) | 0.00 (0.00 to 0.00) | 1.07 (0.72 to 1.51) | 161.21 (108.67 to 227.02) | 0.00 (-0.02 to 0.02) |
| Female | 1.79 (1.50 to 2.12) | 553.31 (464.18 to 657.28) | 0.00 (0.00 to 0.01) | 0.00 (0.00 to 0.00) | 0.00 (0.00 to 0.00) | 0.00 (0.00 to 0.00) | 0.34 (0.23 to 0.48) | 105.66 (72.24 to 148.86) | 0.00 (-0.02 to 0.02) |
| Male | 3.82 (3.23 to 4.46) | 1,111.19 (939.19 to 1,298.58) | 0.00 (-0.01 to 0.01) | 0.00 (0.00 to 0.00) | 0.00 (0.00 to 0.00) | 0.00 (0.00 to 0.00) | 0.73 (0.50 to 1.04) | 213.40 (144.28 to 301.16) | 0.00 (-0.02 to 0.02) |
| Attention-deficit/hyperactivity disorder | | | | | | | | | |
| Total | 18.09 (12.39 to 26.06) | 2,713.36 (1,858.21 to 3,908.68) | 0.00 (-0.02 to 0.02) | 0.22 (0.15 to 0.32) | 33.66 (22.87 to 48.49) | 0.00 (-0.03 to 0.02) | 0.22 (0.11 to 0.38) | 33.28 (16.93 to 57.56) | 0.00 (-0.03 to 0.03) |
| Female | 4.89 (3.32 to 7.09) | 1,513.79 (1,028.01 to 2,195.90) | 0.00 (-0.03 to 0.03) | 0.06 (0.04 to 0.09) | 18.84 (12.65 to 27.42) | -0.01 (-0.04 to 0.02) | 0.06 (0.03 to 0.10) | 18.52 (9.57 to 32.13) | 0.00 (-0.04 to 0.04) |
| Male | 13.20 (9.03 to 19.08) | 3,840.39 (2,626.19 to 5,552.31) | 0.00 (-0.03 to 0.03) | 0.16 (0.11 to 0.24) | 47.59 (32.41 to 68.56) | 0.00 (-0.03 to 0.03) | 0.16 (0.08 to 0.28) | 47.16 (23.83 to 81.10) | 0.00 (-0.03 to 0.04) |
| Conduct disorder | | | | | | | | | |
| Total | 21.73 (14.73 to 29.30) | 3,259.41 (2,209.58 to 4,395.18) | 0.00 (0.00 to 0.00) | 7.39 (4.59 to 10.14) | 1,107.86 (688.06 to 1,521.62) | 0.00 (0.00 to 0.00) | 2.65 (1.40 to 4.38) | 398.17 (210.23 to 657.32) | 0.00 (-0.01 to 0.01) |
| Female | 8.01 (5.12 to 11.18) | 2,481.68 (1,585.09 to 3,462.18) | 0.00 (0.00 to 0.00) | 2.68 (1.59 to 3.88) | 831.14 (493.77 to 1,201.85) | 0.00 (0.00 to 0.00) | 0.87 (0.50 to 1.64) | 301.78 (155.32 to 509.18) | 0.00 (-0.02 to 0.01) |
| Male | 13.71 (9.48 to 18.34) | 3,990.11 (2,757.07 to 5,336.05) | 0.00 (0.00 to 0.00) | 4.70 (2.91 to 6.40) | 1,367.84 (845.85 to 1,863.18) | 0.00 (0.00 to 0.00) | 1.68 (0.90 to 2.72) | 488.74 (261.22 to 792.38) | 0.00 (-0.01 to 0.01) |
| Idiopathic developmental intellectual disability | | | | | | | | | |
| Total | 10.97 (6.20 to 15.55) | 1,645.28 (929.32 to 2,332.43) | -0.02 (-0.05 to 0.01) | NA | NA | NA | 0.47 (0.22 to 0.79) | 70.84 (33.47 to 119.16) | -0.01 (-0.03 to 0.01) |
| Female | 5.43 (3.25 to 7.52) | 1,680.90 (1,005.95 to 2,328.10) | -0.02 (-0.04 to 0.00) | NA | NA | NA | 0.23 (0.12 to 0.39) | 72.04 (36.66 to 119.35) | -0.01 (-0.04 to 0.01) |
| Male | 5.54 (2.95 to 8.03) | 1,611.81 (857.02 to 2,336.49) | -0.02 (-0.05 to 0.01) | NA | NA | NA | 0.24 (0.10 to 0.41) | 69.72 (30.41 to 120.17) | -0.01 (-0.04 to 0.02) |
| Other mental disorders | | | | | | | | | |
| Total | 0.40 (0.25 to 0.57) | 60.01 (37.19 to 86.24) | 0.00 (0.00 to 0.00) | 0.00 (0.00 to 0.00) | 0.00 (0.00 to 0.00) | 0.00 (0.00 to 0.00) | 0.03 (0.02 to 0.05) | 4.77 (2.67 to 7.64) | 0.00 (-0.02 to 0.03) |
| Female | 0.15 (0.09 to 0.21) | 45.99 (28.26 to 66.40) | 0.00 (0.00 to 0.00) | 0.00 (0.00 to 0.00) | 0.00 (0.00 to 0.00) | 0.00 (0.00 to 0.00) | 0.01 (0.01 to 0.02) | 3.66 (1.92 to 5.98) | 0.00 (-0.02 to 0.02) |
| Male | 0.25 (0.16 to 0.36) | 73.19 (45.63 to 105.22) | 0.00 (0.00 to 0.00) | 0.00 (0.00 to 0.00) | 0.00 (0.00 to 0.00) | 0.00 (0.00 to 0.00) | 0.02 (0.01 to 0.03) | 5.81 (3.32 to 9.27) | 0.00 (-0.03 to 0.04) |

**Table S25.** Prevalence, Incidence, and Years Lived with Disability of Mental Disorders Among Adolescents and Young Adults (**Aged 15-19**) in 2021, and Percentage Change from 2019 to 2021 According to GBD.

|  | Prevalence (95% uncertainty interval) | | | Incidence (95% uncertainty interval) | | | Years lived with disability (95% uncertainty interval) | | |
| --- | --- | --- | --- | --- | --- | --- | --- | --- | --- |
| Cause | Counts in million | Rates per 100,000 people | Percentage rate change (2019-2021) | Counts in million | Rates per 100,000 people | Percentage rate change (2019-2021) | Counts in million | Rates per 100,000 people | Percentage rate change (2019-2021) |
| Mental disorders | | | | | | | | | |
| Total | 96.10 (85.18 to 108.97) | 15,401.12(13,651.36 to 17,463.36) | 0.10 (0.09 to 0.12) | 38.47 (28.81 to 48.33) | 6,165.89 (4,617.92 to 7,746.16) | 0.24 (0.20 to 0.26) | 12.72 (9.15 to 16.82) | 2,038.17 (1,465.73 to 2,695.11) | 0.14 (0.12 to 0.16) |
| Female | 48.13 (42.12 to 54.98) | 15,851.52(13,871.07 to 18,105.93) | 0.13 (0.11 to 0.15) | 22.25 (16.66 to 28.28) | 7,326.88 (5,486.80 to 9,313.34) | 0.25 (0.22 to 0.28) | 6.80 (4.81 to 9.04) | 2,239.12 (1,584.75 to 2,977.66) | 0.17 (0.14 to 0.19) |
| Male | 47.97 (42.31 to 53.76) | 14,974.17(13,208.99 to 16,783.23) | 0.08 (0.06 to 0.09) | 16.23 (12.16 to 20.27) | 5,065.35 (3,796.00 to 6,329.13) | 0.22 (0.18 to 0.25) | 5.92 (4.28 to 7.80) | 1,847.67 (1,335.76 to 2,435.69) | 0.11 (0.09 to 0.14) |
| Schizophrenia | | | | | | | | | |
| Total | 0.44 (0.28 to 0.66) | 70.81 (45.10 to 105.17) | 0.00 (-0.01 to 0.01) | 0.16 (0.09 to 0.25) | 25.86 (14.40 to 40.69) | 0.00 (-0.01 to 0.01) | 0.30 (0.18 to 0.46) | 47.59 (28.95 to 73.42) | 0.00 (-0.05 to 0.05) |
| Female | 0.20 (0.13 to 0.30) | 65.83 (41.50 to 98.24) | 0.00 (-0.02 to 0.01) | 0.07 (0.04 to 0.12) | 24.28 (13.35 to 38.48) | 0.00 (-0.01 to 0.02) | 0.13 (0.08 to 0.21) | 43.81 (26.47 to 67.85) | 0.00 (-0.06 to 0.07) |
| Male | 0.24 (0.15 to 0.36) | 75.54 (48.21 to 112.26) | 0.00 (-0.02 to 0.01) | 0.09 (0.05 to 0.14) | 27.36 (15.39 to 42.80) | 0.00 (-0.01 to 0.01) | 0.16 (0.10 to 0.25) | 51.17 (31.00 to 78.96) | 0.00 (-0.06 to 0.06) |
| Depressive disorders | | | | | | | | | |
| Total | 21.09 (15.61 to 27.68) | 3,380.02 (2,501.25 to 4,435.82) | 0.23 (0.19 to 0.26) | 27.85 (19.03 to 37.54) | 4,463.10 (3,049.17 to 6,015.60) | 0.29 (0.26 to 0.31) | 3.98 (2.49 to 6.05) | 637.70 (399.78 to 969.35) | 0.26 (0.22 to 0.29) |
| Female | 12.84 (9.49 to 16.84) | 4,228.78 (3,125.15 to 5,544.86) | 0.23 (0.19 to 0.26) | 16.96 (11.66 to 22.81) | 5,583.94 (3,839.30 to 7,512.47) | 0.29 (0.26 to 0.32) | 2.42 (1.52 to 3.65) | 797.36 (501.51 to 1,202.02) | 0.26 (0.22 to 0.29) |
| Male | 8.25 (6.06 to 10.87) | 2,575.45 (1,892.94 to 3,392.19) | 0.22 (0.18 to 0.26) | 10.89 (7.36 to 14.92) | 3,400.62 (2,297.90 to 4,657.67) | 0.29 (0.26 to 0.31) | 1.56 (0.97 to 2.38) | 486.36 (303.62 to 741.64) | 0.26 (0.22 to 0.29) |
| Bipolar disorder | | | | | | | | | |
| Total | 3.10 (2.25 to 4.32) | 497.56 (360.11 to 691.95) | 0.00 (-0.01 to 0.00) | 0.54 (0.39 to 0.73) | 86.68 (62.94 to 117.60) | 0.00 (0.00 to 0.00) | 0.69 (0.42 to 1.09) | 111.09 (66.80 to 174.64) | -0.01 (-0.02 to 0.01) |
| Female | 1.57 (1.13 to 2.20) | 517.74 (373.04 to 723.92) | 0.00 (-0.01 to 0.00) | 0.27 (0.20 to 0.37) | 89.77 (65.12 to 121.90) | 0.00 (0.00 to 0.00) | 0.35 (0.21 to 0.55) | 114.74 (68.33 to 181.23) | -0.01 (-0.03 to 0.02) |
| Male | 1.53 (1.12 to 2.12) | 478.43 (349.02 to 661.60) | 0.00 (-0.01 to 0.00) | 0.27 (0.20 to 0.36) | 83.75 (60.97 to 113.38) | 0.00 (0.00 to 0.00) | 0.34 (0.21 to 0.54) | 107.64 (65.03 to 169.92) | -0.01 (-0.03 to 0.02) |
| Anxiety disorders | | | | | | | | | |
| Total | 33.04 (25.16 to 42.79) | 5,295.79 (4,031.42 to 6,858.02) | 0.24 (0.21 to 0.27) | 5.43 (3.45 to 7.62) | 870.27 (553.66 to 1,221.54) | 0.26 (0.23 to 0.29) | 4.05 (2.55 to 6.00) | 649.72 (407.89 to 962.34) | 0.24 (0.21 to 0.27) |
| Female | 20.12 (15.32 to 25.92) | 6,625.56 (5,045.43 to 8,535.00) | 0.24 (0.22 to 0.27) | 3.19 (2.02 to 4.52) | 1,051.35 (665.04 to 1,488.87) | 0.26 (0.23 to 0.29) | 2.46 (1.54 to 3.60) | 808.75 (508.67 to 1,184.24) | 0.24 (0.21 to 0.27) |
| Male | 12.93 (9.82 to 16.81) | 4,035.27 (3,066.99 to 5,248.54) | 0.23 (0.20 to 0.26) | 2.24 (1.44 to 3.09) | 698.61 (449.53 to 965.98) | 0.25 (0.22 to 0.28) | 1.60 (0.99 to 2.36) | 498.97 (309.22 to 736.26) | 0.23 (0.20 to 0.26) |
| Eating disorders | | | | | | | | | |
| Total | 2.58 (1.62 to 4.10) | 412.71 (259.80 to 657.21) | 0.00 (-0.01 to 0.00) | 3.26 (1.70 to 5.74) | 522.10 (272.15 to 919.20) | 0.00 (-0.01 to 0.01) | 0.55 (0.31 to 0.96) | 88.54 (49.41 to 153.70) | -0.01 (-0.02 to 0.01) |
| Female | 1.61 (1.03 to 2.55) | 531.10 (339.40 to 838.56) | -0.01 (-0.02 to 0.00) | 1.35 (0.77 to 2.26) | 445.93 (252.76 to 745.78) | 0.00 (-0.01 to 0.01) | 0.34 (0.20 to 0.58) | 113.41 (64.99 to 191.95) | -0.01 (-0.03 to 0.01) |
| Male | 0.96 (0.58 to 1.62) | 300.49 (182.36 to 505.63) | 0.00 (-0.01 to 0.01) | 1.90 (0.93 to 3.48) | 594.30 (291.23 to 1,086.18) | 0.01 (-0.01 to 0.02) | 0.21 (0.11 to 0.38) | 64.97 (34.01 to 117.71) | 0.00 (-0.03 to 0.04) |
| Autism spectrum disorders | | | | | | | | | |
| Total | 5.19 (4.37 to 6.09) | 831.01 (700.72 to 975.39) | 0.00 (0.00 to 0.01) | 0.00 (0.00 to 0.00) | 0.00 (0.00 to 0.00) | 0.00 (0.00 to 0.00) | 0.99 (0.67 to 1.39) | 158.35 (107.00 to 222.73) | 0.00 (-0.01 to 0.01) |
| Female | 1.66 (1.39 to 1.97) | 547.28 (458.67 to 650.18) | 0.00 (0.00 to 0.01) | 0.00 (0.00 to 0.00) | 0.00 (0.00 to 0.00) | 0.00 (0.00 to 0.00) | 0.31 (0.22 to 0.44) | 103.43 (70.96 to 146.55) | 0.00 (-0.02 to 0.03) |
| Male | 3.52 (2.98 to 4.12) | 1,099.98 (928.88 to 1,285.29) | 0.00 (-0.01 to 0.01) | 0.00 (0.00 to 0.00) | 0.00 (0.00 to 0.00) | 0.00 (0.00 to 0.00) | 0.67 (0.46 to 0.95) | 210.41 (143.02 to 296.22) | 0.00 (-0.02 to 0.02) |
| Attention-deficit/hyperactivity disorder | | | | | | | | | |
| Total | 13.46 (9.22 to 18.80) | 2,157.35 (1,478.22 to 3,013.00) | 0.00 (-0.02 to 0.02) | 0.00 (0.00 to 0.00) | 0.00 (0.00 to 0.00) | 0.00 (0.00 to 0.00) | 0.16 (0.09 to 0.27) | 26.33 (13.75 to 43.37) | 0.00 (-0.02 to 0.03) |
| Female | 3.71 (2.54 to 5.24) | 1,223.42 (837.70 to 1,724.68) | 0.00 (-0.03 to 0.02) | 0.00 (0.00 to 0.00) | 0.00 (0.00 to 0.00) | 0.00 (0.00 to 0.00) | 0.05 (0.02 to 0.08) | 14.86 (7.72 to 25.06) | 0.00 (-0.04 to 0.03) |
| Male | 9.75 (6.70 to 13.57) | 3,042.65 (2,090.62 to 4,235.12) | 0.00 (-0.02 to 0.03) | 0.00 (0.00 to 0.00) | 0.00 (0.00 to 0.00) | 0.00 (0.00 to 0.00) | 0.12 (0.06 to 0.19) | 37.20 (19.46 to 60.81) | 0.00 (-0.02 to 0.04) |
| Conduct disorder | | | | | | | | | |
| Total | 11.33 (7.82 to 16.17) | 1,815.94 (1,253.88 to 2,590.68) | 0.00 (0.00 to 0.00) | 1.23 (0.57 to 2.13) | 197.88 (91.21 to 341.38) | 0.00 (0.00 to 0.00) | 1.37 (0.71 to 2.14) | 219.60 (113.05 to 343.39) | 0.00 (-0.01 to 0.01) |
| Female | 3.80 (2.54 to 5.76) | 1,251.86 (835.01 to 1,896.22) | 0.00 (0.00 to 0.00) | 0.40 (0.16 to 0.83) | 131.62 (54.32 to 273.26) | 0.00 (0.00 to 0.00) | 0.46 (0.23 to 0.76) | 149.87 (75.52 to 249.00) | 0.00 (-0.02 to 0.02) |
| Male | 7.53 (5.24 to 10.42) | 2,350.65 (1,637.16 to 3,252.15) | 0.00 (0.00 to 0.00) | 0.84 (0.39 to 1.38) | 260.70 (123.02 to 430.54) | 0.00 (0.00 to 0.00) | 0.92 (0.47 to 1.44) | 285.70 (148.19 to 451.07) | 0.00 (-0.02 to 0.01) |
| Idiopathic developmental intellectual disability | | | | | | | | | |
| Total | 9.96 (5.65 to 14.15) | 1,596.64 (905.22 to 2,268.25) | -0.01 (-0.04 to 0.02) | NA | NA | NA | 0.42 (0.20 to 0.72) | 67.72 (31.38 to 114.88) | 0.00 (-0.02 to 0.01) |
| Female | 4.98 (2.99 to 6.91) | 1,639.24 (986.20 to 2,274.21) | -0.01 (-0.04 to 0.01) | NA | NA | NA | 0.21 (0.10 to 0.35) | 68.91 (34.39 to 114.00) | 0.00 (-0.03 to 0.02) |
| Male | 4.99 (2.65 to 7.25) | 1,556.26 (827.92 to 2,262.60) | -0.01 (-0.05 to 0.02) | NA | NA | NA | 0.21 (0.09 to 0.37) | 66.59 (28.83 to 115.34) | -0.01 (-0.03 to 0.02) |
| Other mental disorders | | | | | | | | | |
| Total | 2.55 (1.59 to 3.69) | 409.20 (254.05 to 590.74) | 0.00 (0.00 to 0.00) | 0.00 (0.00 to 0.00) | 0.00 (0.00 to 0.00) | 0.00 (0.00 to 0.00) | 0.20 (0.11 to 0.32) | 31.53 (17.59 to 50.78) | 0.00 (-0.04 to 0.03) |
| Female | 0.95 (0.58 to 1.37) | 313.73 (192.53 to 452.62) | 0.00 (0.00 to 0.00) | 0.00 (0.00 to 0.00) | 0.00 (0.00 to 0.00) | 0.00 (0.00 to 0.00) | 0.07 (0.04 to 0.12) | 24.00 (12.83 to 38.61) | 0.00 (-0.06 to 0.05) |
| Male | 1.60 (1.00 to 2.31) | 499.69 (312.39 to 721.96) | 0.00 (0.00 to 0.00) | 0.00 (0.00 to 0.00) | 0.00 (0.00 to 0.00) | 0.00 (0.00 to 0.00) | 0.12 (0.07 to 0.20) | 38.67 (22.11 to 62.37) | 0.00 (-0.05 to 0.04) |

**Table S26.** Prevalence, Incidence, and Years Lived with Disability of Mental Disorders Among Adolescents and Young Adults (**Aged 20-24**) in 2021, and Percentage Change from 2019 to 2021 According to GBD.

|  | Prevalence (95% uncertainty interval) | | | Incidence (95% uncertainty interval) | | | Years lived with disability (95% uncertainty interval) | | |
| --- | --- | --- | --- | --- | --- | --- | --- | --- | --- |
| Cause | Counts in million | Rates per 100,000 people | Percentage rate change (2019-2021) | Counts in million | Rates per 100,000 people | Percentage rate change (2019-2021) | Counts in million | Rates per 100,000 people | Percentage rate change (2019-2021) |
| Mental disorders | | | | | | | | | |
| Total | 94.10 (81.52 to 107.70) | 15,758.32(13,651.34 to 18,034.86) | 0.12 (0.10 to 0.14) | 40.66 (30.86 to 54.90) | 6,809.14 (5,166.99 to 9,193.45) | 0.25 (0.23 to 0.28) | 13.80 (9.94 to 18.29) | 2,310.18 (1,664.59 to 3,062.30) | 0.15 (0.13 to 0.17) |
| Female | 50.19 (43.04 to 57.80) | 17,084.64(14,65166 to 19,675.31) | 0.14 (0.13 to 0.16) | 23.85 (17.94 to 32.36) | 8,117.48 (6,106.38 to 11,015.30) | 0.26 (0.24 to 0.29) | 7.65 (5.46 to 10.25) | 2,605.13 (1,858.74 to 3,487.70) | 0.17 (0.14 to 0.19) |
| Male | 43.92 (38.35 to 49.85) | 14,474.20(12,640.05 to 16,430.37) | 0.10 (0.08 to 0.12) | 16.82 (12.89 to 22.43) | 5,542.42 (4,248.84 to 7,392.41) | 0.24 (0.21 to 0.27) | 6.14 (4.50 to 8.02) | 2,024.61 (1,482.06 to 2,642.55) | 0.13 (0.10 to 0.16) |
| Schizophrenia | | | | | | | | | |
| Total | 1.50 (0.97 to 2.19) | 250.54 (162.22 to 367.08) | 0.00 (-0.01 to 0.00) | 0.29 (0.18 to 0.41) | 48.05 (30.48 to 68.32) | 0.00 (-0.01 to 0.00) | 1.00 (0.63 to 1.53) | 166.66 (105.56 to 256.80) | -0.01 (-0.03 to 0.02) |
| Female | 0.69 (0.45 to 1.02) | 234.56 (151.81 to 345.88) | 0.00 (-0.02 to 0.01) | 0.13 (0.08 to 0.19) | 45.26 (28.58 to 64.50) | 0.00 (-0.01 to 0.01) | 0.45 (0.28 to 0.70) | 154.13 (96.63 to 238.08) | -0.01 (-0.05 to 0.03) |
| Male | 0.81 (0.52 to 1.17) | 266.01 (172.36 to 386.95) | -0.01 (-0.02 to 0.01) | 0.15 (0.10 to 0.22) | 50.75 (32.26 to 72.04) | 0.00 (-0.02 to 0.01) | 0.54 (0.34 to 0.84) | 178.78 (112.76 to 277.24) | -0.01 (-0.05 to 0.02) |
| Depressive disorders | | | | | | | | | |
| Total | 27.97 (21.04 to 38.57) | 4,683.11 (3,523.62 to 6,459.52) | 0.21 (0.17 to 0.24) | 32.54 (23.81 to 46.63) | 5,448.60 (3,987.76 to 7,808.03) | 0.28 (0.25 to 0.31) | 5.11 (3.22 to 7.65) | 855.64 (538.75 to 1,281.30) | 0.24 (0.21 to 0.28) |
| Female | 16.72 (12.61 to 23.08) | 5,691.25 (4,291.75 to 7,856.27) | 0.21 (0.17 to 0.25) | 19.52 (14.28 to 28.19) | 6,646.48 (4,862.94 to 9,596.22) | 0.28 (0.26 to 0.31) | 3.04 (1.92 to 4.55) | 1,035.05 (654.07 to 1,549.83) | 0.24 (0.21 to 0.28) |
| Male | 11.25 (8.39 to 15.39) | 3,707.05 (2,764.25 to 5,072.45) | 0.21 (0.17 to 0.24) | 13.01 (9.49 to 18.41) | 4,288.83 (3,128.68 to 6,069.29) | 0.28 (0.25 to 0.30) | 2.07 (1.29 to 3.11) | 681.93 (425.37 to 1,024.53) | 0.24 (0.21 to 0.28) |
| Bipolar disorder | | | | | | | | | |
| Total | 4.04 (2.96 to 5.52) | 677.27 (495.05 to 924.47) | 0.00 (0.00 to 0.00) | 0.26 (0.12 to 0.44) | 43.31 (20.62 to 74.24) | 0.01 (0.00 to 0.01) | 0.89 (0.56 to 1.36) | 149.59 (93.97 to 227.39) | 0.00 (-0.02 to 0.02) |
| Female | 2.06 (1.50 to 2.82) | 702.91 (512.08 to 961.65) | 0.00 (0.00 to 0.00) | 0.13 (0.06 to 0.22) | 44.49 (21.12 to 76.53) | 0.00 (0.00 to 0.01) | 0.45 (0.28 to 0.69) | 153.69 (96.12 to 233.82) | 0.00 (-0.02 to 0.02) |
| Male | 1.98 (1.45 to 2.70) | 652.44 (478.55 to 888.47) | 0.00 (0.00 to 0.01) | 0.13 (0.06 to 0.22) | 42.15 (20.13 to 72.03) | 0.01 (0.01 to 0.01) | 0.44 (0.28 to 0.67) | 145.62 (91.06 to 221.77) | 0.00 (-0.03 to 0.03) |
| Anxiety disorders | | | | | | | | | |
| Total | 33.83 (24.96 to 44.70) | 5,664.51 (4,179.26 to 7,486.07) | 0.25 (0.22 to 0.28) | 5.07 (2.90 to 7.43) | 849.64 (485.17 to 1,243.39) | 0.27 (0.24 to 0.30) | 4.11 (2.58 to 6.11) | 688.51 (432.21 to 1,023.28) | 0.24 (0.21 to 0.28) |
| Female | 20.95 (15.48 to 27.60) | 7,132.57 (5,270.13 to 9,395.55) | 0.25 (0.23 to 0.28) | 3.01 (1.71 to 4.41) | 1,025.17 (582.00 to 1,502.34) | 0.27 (0.24 to 0.31) | 2.53 (1.59 to 3.75) | 860.75 (540.02 to 1,276.94) | 0.25 (0.22 to 0.28) |
| Male | 12.87 (9.39 to 17.17) | 4,243.16 (3,093.26 to 5,660.23) | 0.24 (0.21 to 0.27) | 2.06 (1.18 to 2.98) | 679.71 (388.06 to 981.44) | 0.26 (0.23 to 0.29) | 1.58 (0.99 to 2.39) | 521.75 (325.21 to 788.32) | 0.24 (0.20 to 0.27) |
| Eating disorders | | | | | | | | | |
| Total | 3.40 (2.11 to 5.13) | 569.14 (353.48 to 859.19) | 0.00 (-0.01 to 0.01) | 2.51 (1.37 to 4.33) | 419.54 (229.08 to 724.74) | 0.01 (-0.01 to 0.02) | 0.72 (0.37 to 1.20) | 121.05 (62.11 to 200.98) | 0.00 (-0.02 to 0.02) |
| Female | 2.23 (1.43 to 3.21) | 758.39 (485.28 to 1,094.23) | 0.00 (-0.01 to 0.01) | 1.05 (0.61 to 1.68) | 356.08 (205.97 to 573.20) | 0.00 (-0.01 to 0.01) | 0.47 (0.25 to 0.76) | 160.28 (84.42 to 260.30) | -0.01 (-0.02 to 0.01) |
| Male | 1.17 (0.67 to 1.94) | 385.91 (219.37 to 640.12) | 0.01 (0.00 to 0.02) | 1.46 (0.75 to 2.61) | 480.99 (248.69 to 860.86) | 0.01 (-0.01 to 0.02) | 0.25 (0.12 to 0.45) | 83.06 (39.25 to 149.79) | 0.01 (-0.02 to 0.04) |
| Autism spectrum disorders | | | | | | | | | |
| Total | 4.90 (4.13 to 5.75) | 820.52 (691.85 to 962.19) | 0.00 (0.00 to 0.01) | 0.00 (0.00 to 0.00) | 0.00 (0.00 to 0.00) | 0.00 (0.00 to 0.00) | 0.93 (0.63 to 1.31) | 155.45 (105.17 to 219.08) | 0.00 (-0.01 to 0.02) |
| Female | 1.58 (1.32 to 1.88) | 539.52 (451.02 to 640.53) | 0.01 (0.00 to 0.01) | 0.00 (0.00 to 0.00) | 0.00 (0.00 to 0.00) | 0.00 (0.00 to 0.00) | 0.30 (0.20 to 0.42) | 101.07 (69.12 to 141.80) | 0.00 (-0.02 to 0.03) |
| Male | 3.31 (2.80 to 3.87) | 1,092.58 (921.73 to 1,276.16) | 0.00 (-0.01 to 0.01) | 0.00 (0.00 to 0.00) | 0.00 (0.00 to 0.00) | 0.00 (0.00 to 0.00) | 0.63 (0.43 to 0.89) | 208.11 (141.42 to 293.92) | 0.00 (-0.02 to 0.02) |
| Attention-deficit/hyperactivity disorder | | | | | | | | | |
| Total | 9.48 (6.55 to 13.21) | 1,587.64 (1,097.13 to 2,212.28) | -0.01 (-0.03 to 0.01) | 0.00 (0.00 to 0.00) | 0.00 (0.00 to 0.00) | 0.00 (0.00 to 0.00) | 0.12 (0.06 to 0.19) | 19.31 (10.46 to 31.74) | -0.01 (-0.03 to 0.02) |
| Female | 2.70 (1.82 to 3.76) | 919.16 (620.21 to 1,280.17) | -0.01 (-0.04 to 0.01) | 0.00 (0.00 to 0.00) | 0.00 (0.00 to 0.00) | 0.00 (0.00 to 0.00) | 0.03 (0.02 to 0.05) | 11.09 (6.02 to 18.26) | -0.02 (-0.05 to 0.02) |
| Male | 6.78 (4.73 to 9.44) | 2,234.85 (1,563.29 to 3,112.23) | -0.01 (-0.03 to 0.02) | 0.00 (0.00 to 0.00) | 0.00 (0.00 to 0.00) | 0.00 (0.00 to 0.00) | 0.08 (0.04 to 0.14) | 27.26 (14.73 to 44.94) | -0.01 (-0.04 to 0.02) |
| Conduct disorder | | | | | | | | | |
| Total | 0.66 (0.37 to 1.04) | 109.88 (61.50 to 173.79) | 0.00 (0.00 to 0.00) | NA | NA | NA | 0.08 (0.04 to 0.14) | 13.15 (6.27 to 23.20) | 0.00 (-0.04 to 0.05) |
| Female | 0.14 (0.06 to 0.25) | 48.11 (19.89 to 85.28) | 0.00 (0.00 to 0.01) | NA | NA | NA | 0.02 (0.01 to 0.03) | 5.65 (2.19 to 11.02) | 0.00 (-0.09 to 0.10) |
| Male | 0.51 (0.31 to 0.78) | 169.69 (101.00 to 257.74) | 0.00 (0.00 to 0.00) | NA | NA | NA | 0.06 (0.03 to 0.11) | 20.42 (9.94 to 35.52) | 0.00 (-0.05 to 0.05) |
| Idiopathic developmental intellectual disability | | | | | | | | | |
| Total | 8.85 (4.95 to 12.69) | 1,481.24 (829.43 to 2,125.70) | 0.01 (-0.03 to 0.04) | NA | NA | NA | 0.37 (0.17 to 0.64) | 62.77 (29.26 to 106.52) | 0.01 (-0.01 to 0.03) |
| Female | 4.49 (2.67 to 6.28) | 1,527.34 (907.87 to 2,136.20) | 0.01 (-0.02 to 0.04) | NA | NA | NA | 0.19 (0.09 to 0.31) | 63.84 (31.80 to 105.39) | 0.02 (-0.01 to 0.04) |
| Male | 4.36 (2.28 to 6.40) | 1,436.61 (753.08 to 2,110.27) | 0.01 (-0.03 to 0.04) | NA | NA | NA | 0.19 (0.08 to 0.33) | 61.73 (26.88 to 107.47) | 0.01 (-0.02 to 0.04) |
| Other mental disorders | | | | | | | | | |
| Total | 6.09 (3.99 to 8.63) | 1,019.79 (667.65 to 1,444.56) | 0.00 (0.00 to 0.00) | 0.00 (0.00 to 0.00) | 0.00 (0.00 to 0.00) | 0.00 (0.00 to 0.00) | 0.47 (0.27 to 0.73) | 78.06 (45.23 to 122.97) | 0.00 (-0.02 to 0.02) |
| Female | 2.31 (1.49 to 3.27) | 786.71 (508.04 to 1,113.41) | 0.00 (0.00 to 0.00) | 0.00 (0.00 to 0.00) | 0.00 (0.00 to 0.00) | 0.00 (0.00 to 0.00) | 0.17 (0.10 to 0.28) | 59.57 (32.90 to 94.43) | 0.00 (-0.04 to 0.03) |
| Male | 3.78 (2.49 to 5.37) | 1,245.46 (819.72 to 1,770.63) | 0.00 (0.00 to 0.00) | 0.00 (0.00 to 0.00) | 0.00 (0.00 to 0.00) | 0.00 (0.00 to 0.00) | 0.29 (0.17 to 0.46) | 95.95 (56.94 to 150.31) | 0.00 (-0.03 to 0.03) |

**Figure S1**. Prevalence, incidence and years lived with disability (YLDs) of mental disorders by age and sex in 2021. (A) Prevalence; (B) Incidence; (C) YLDs.


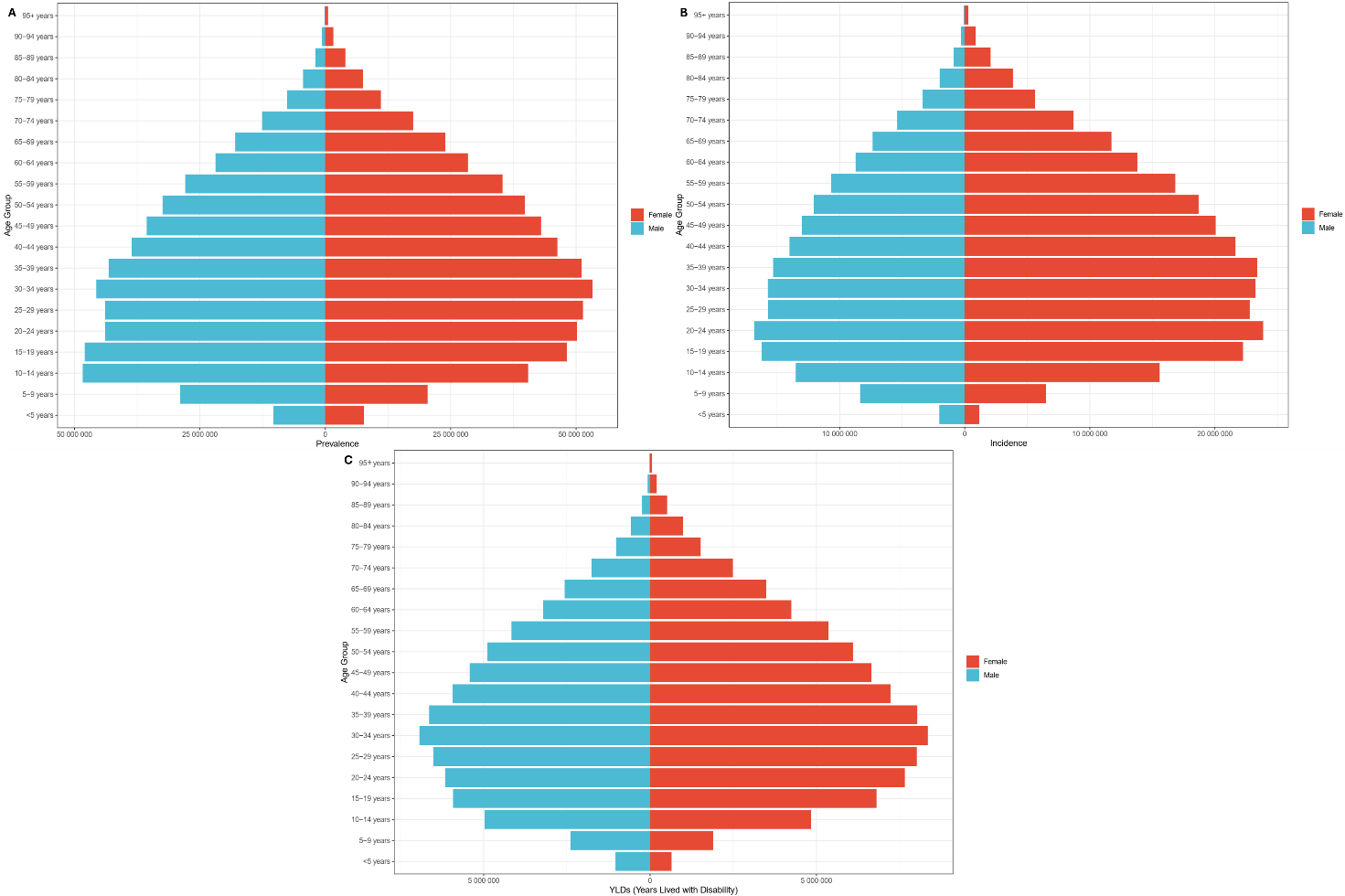


**Figure S2**. Rankings of YLDs numbers for mental disorders by 10-24 age group for both sexes combined, 1990, 2019, and 2021.


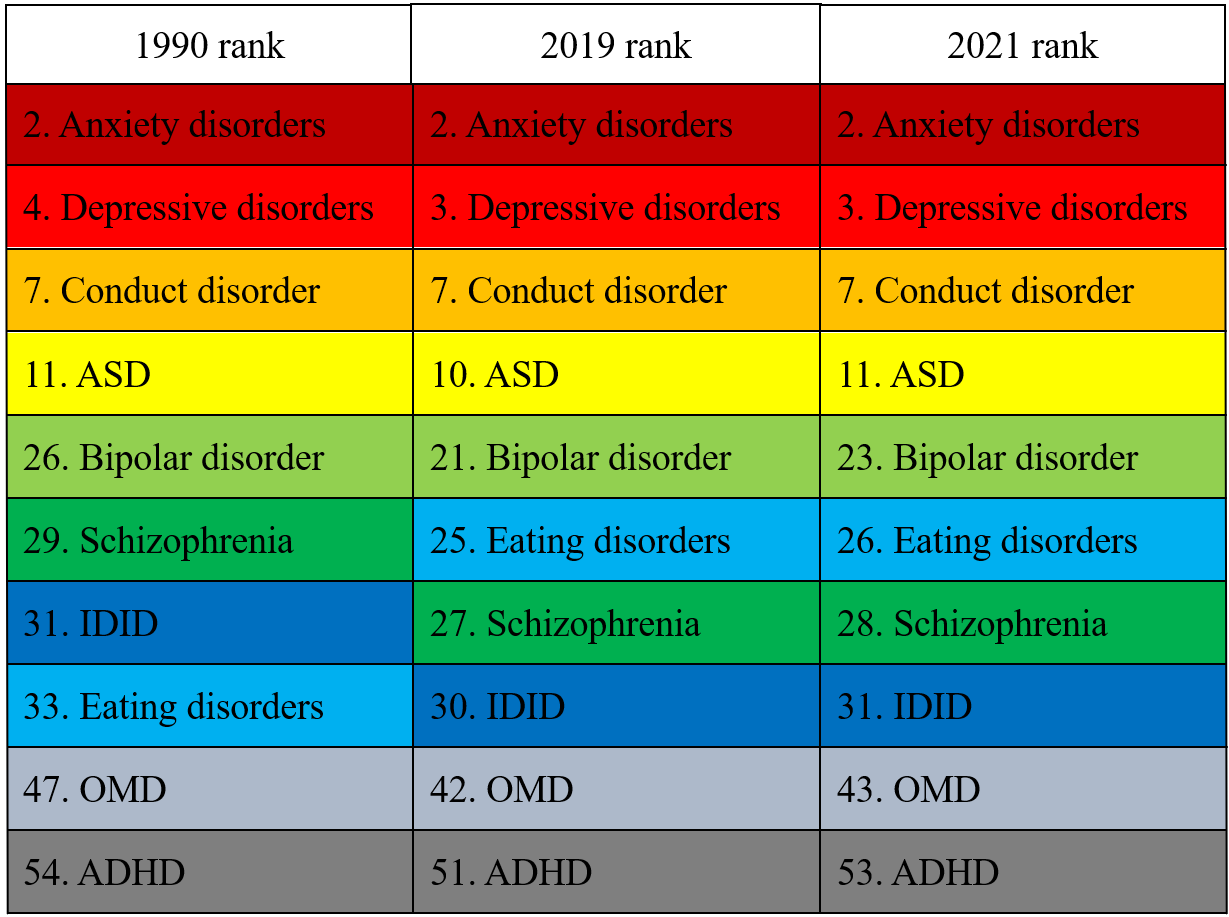


Mental disorders were ranked out of all Level 3 causes within the Global Burden of Diseases, Injuries, and Risk Factors Study. Disorders are ordered from highest to lowest ranking. Each colour represents a different mental disorder. YLDs, years lived with disability; ADHD, attention-deficit/hyperactivity disorder; ASD, autism spectrum disorder; IDID, idiopathic developmental intellectual disability; OMD, other mental disorders.

**Figure S3**. Global and regional trends in the **prevalence numbers** of mental disorders among individuals aged 10-24 (1990-2021).


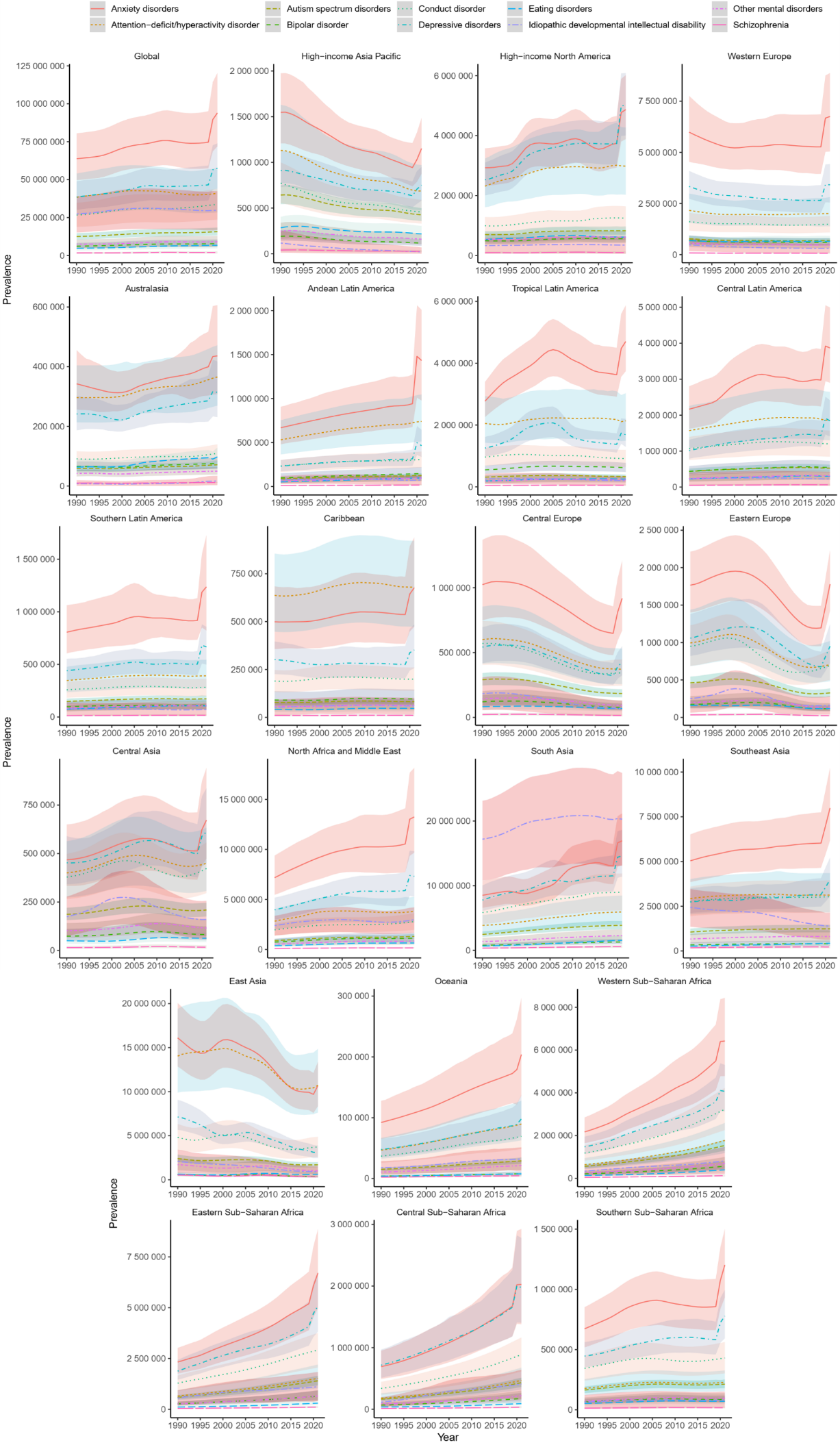


**Figure S4**. Global and regional trends in the **age-standardized prevalence rates** of mental disorders among individuals aged 10-24 years (1990-2021).


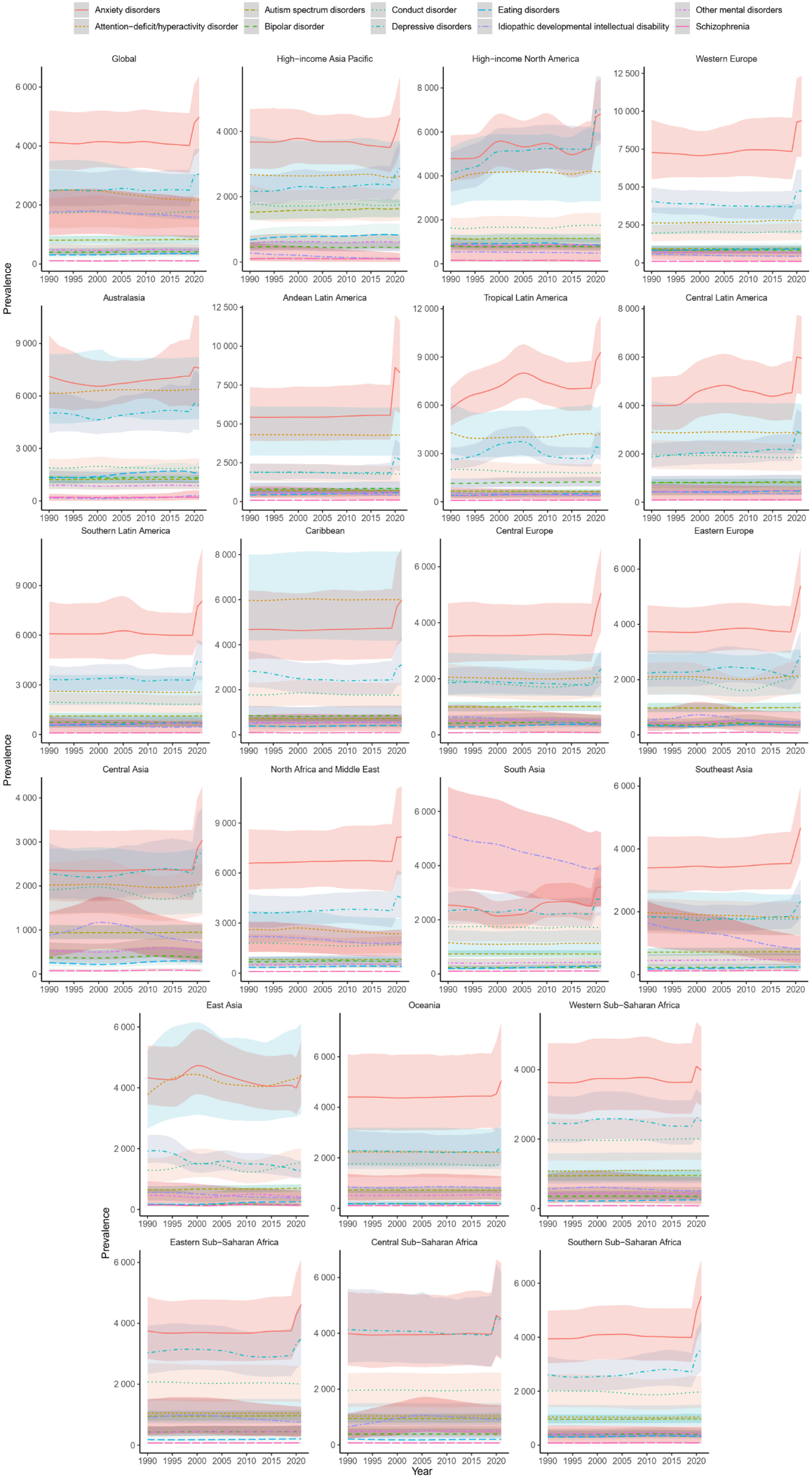


**Figure S5**. Global and regional trends in the **incidence numbers** of mental disorders among individuals aged 10-24 years (1990-2021).


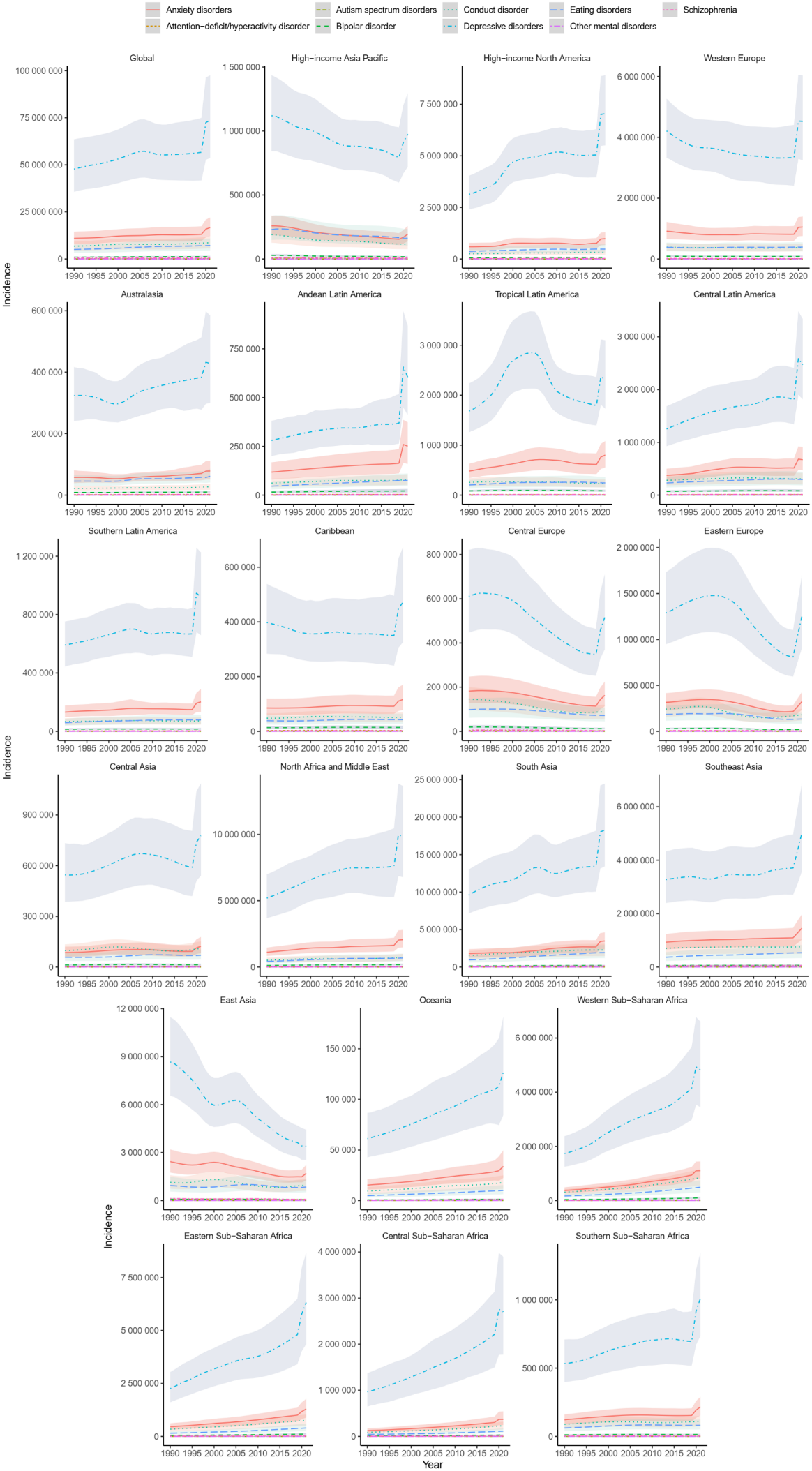


**Figure S6**. Global and regional trends in the **age-standardized incidence rates** of mental disorders among individuals aged 10-24 years (1990-2021).


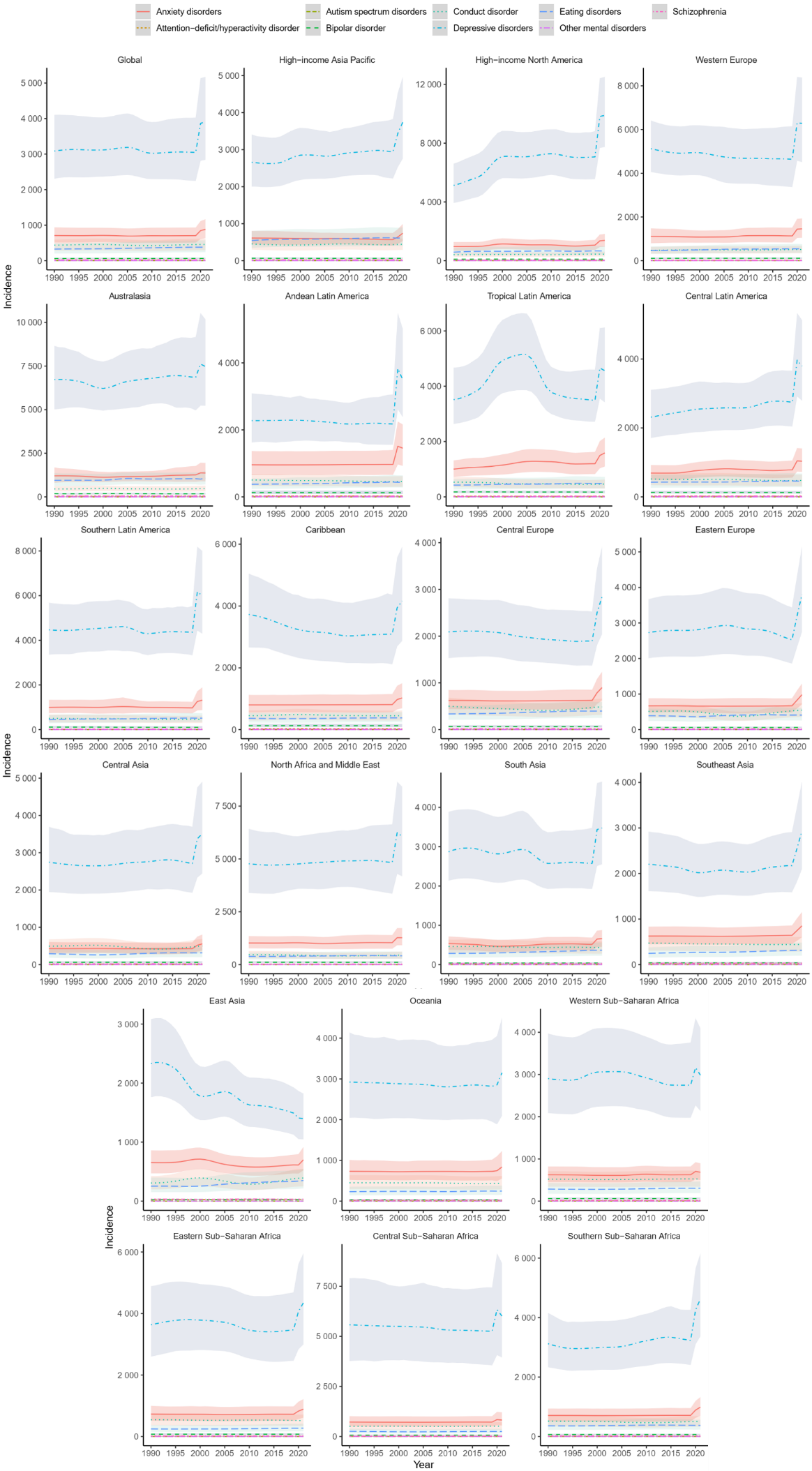


**Figure S7**. Global and regional trends in the **years lived with disability (YLDs) numbers** of mental disorders among individuals aged 10-24 years (1990-2021).


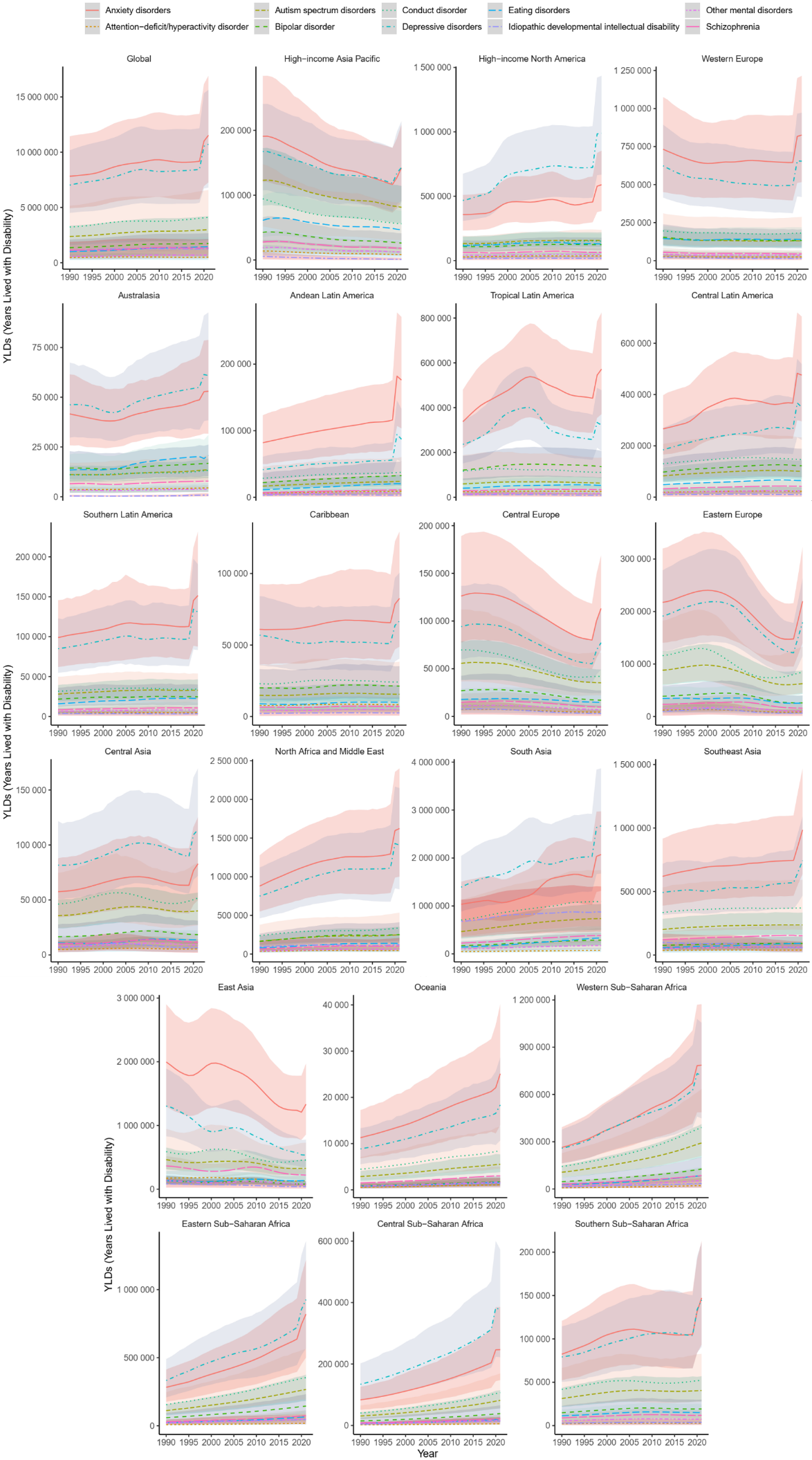


**Figure S8**. Global and regional trends in the **age-standardized years lived with disability (YLDs) rates** of mental disorders among individuals aged 10-24 years (1990-2021).


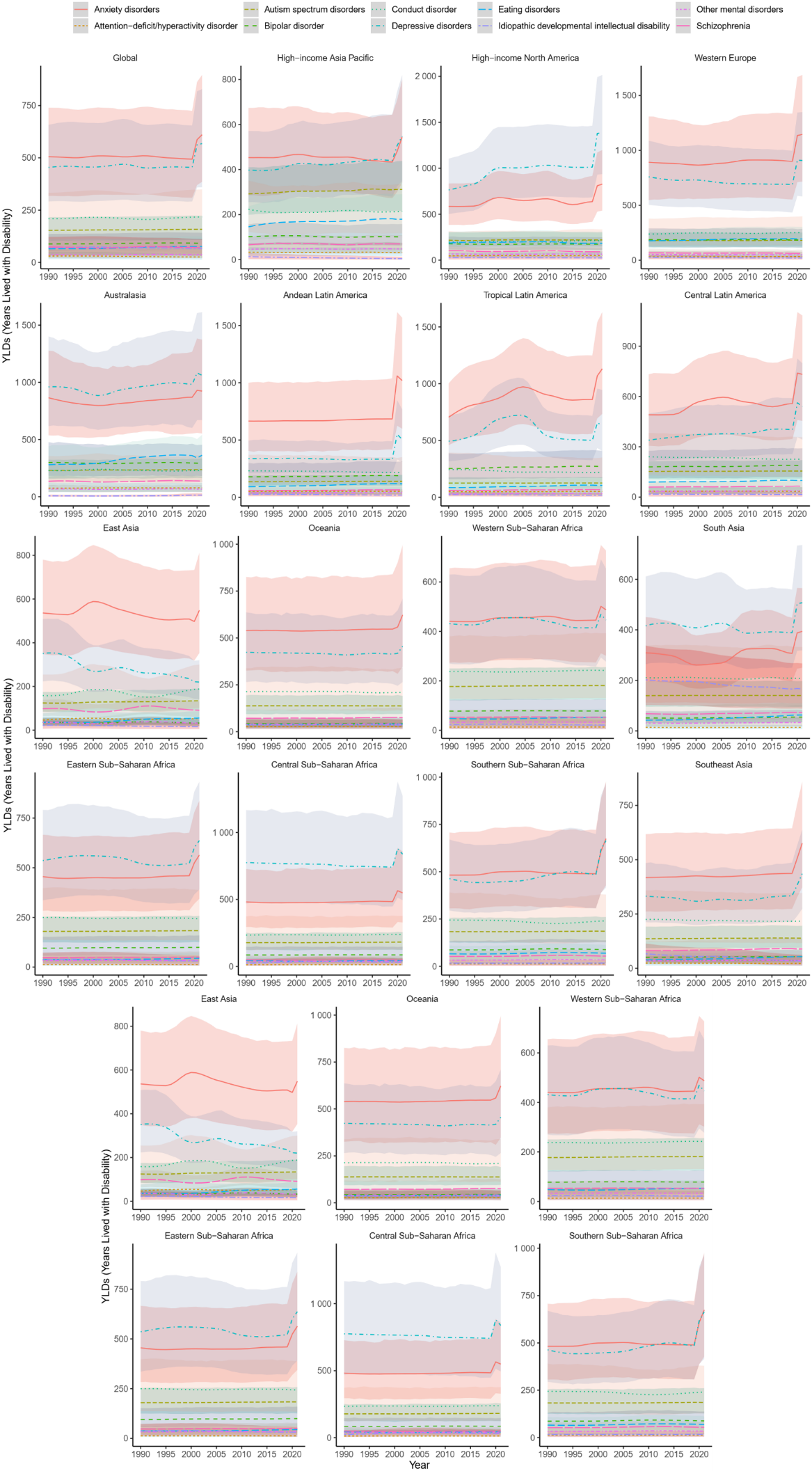


**Figure S9**. Trends in age-standardized prevalence rates of depressive disorders and anxiety disorders among individuals aged 10-24 across global and 21 regions from 2019 to 2021. (A) Age-standardized prevalence rates of deoressive disorders; (B) Age-standardized prevalence rates of anxiety disorders.


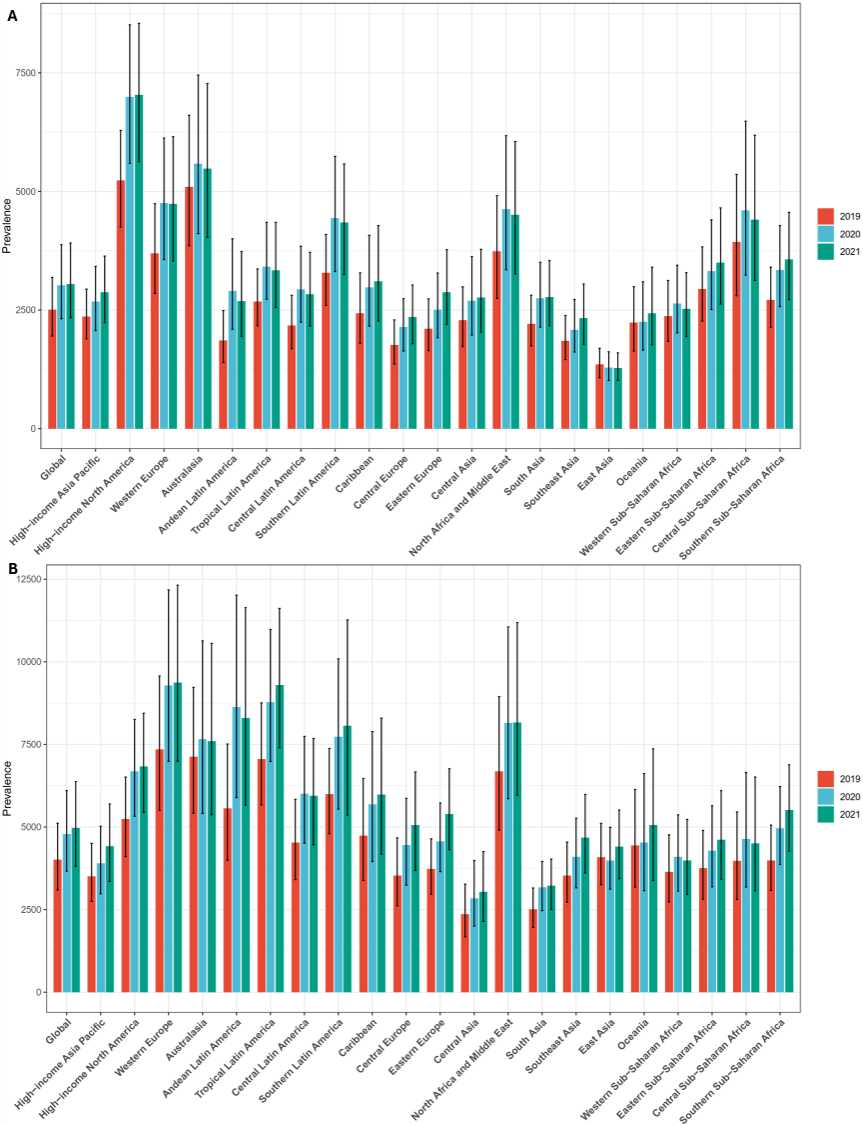


Lines indicate prevalent case with 95% uncertainty intervals for men and women.

**Figure S10**. Distribution of age-standardized prevalence, incidence, and years lived with disability (YLDs) rates of mental disorders among individuals aged 10-24 years across 21 regions in 2021. (A) Prevalence; (B) Incidence; (C) YLDs.


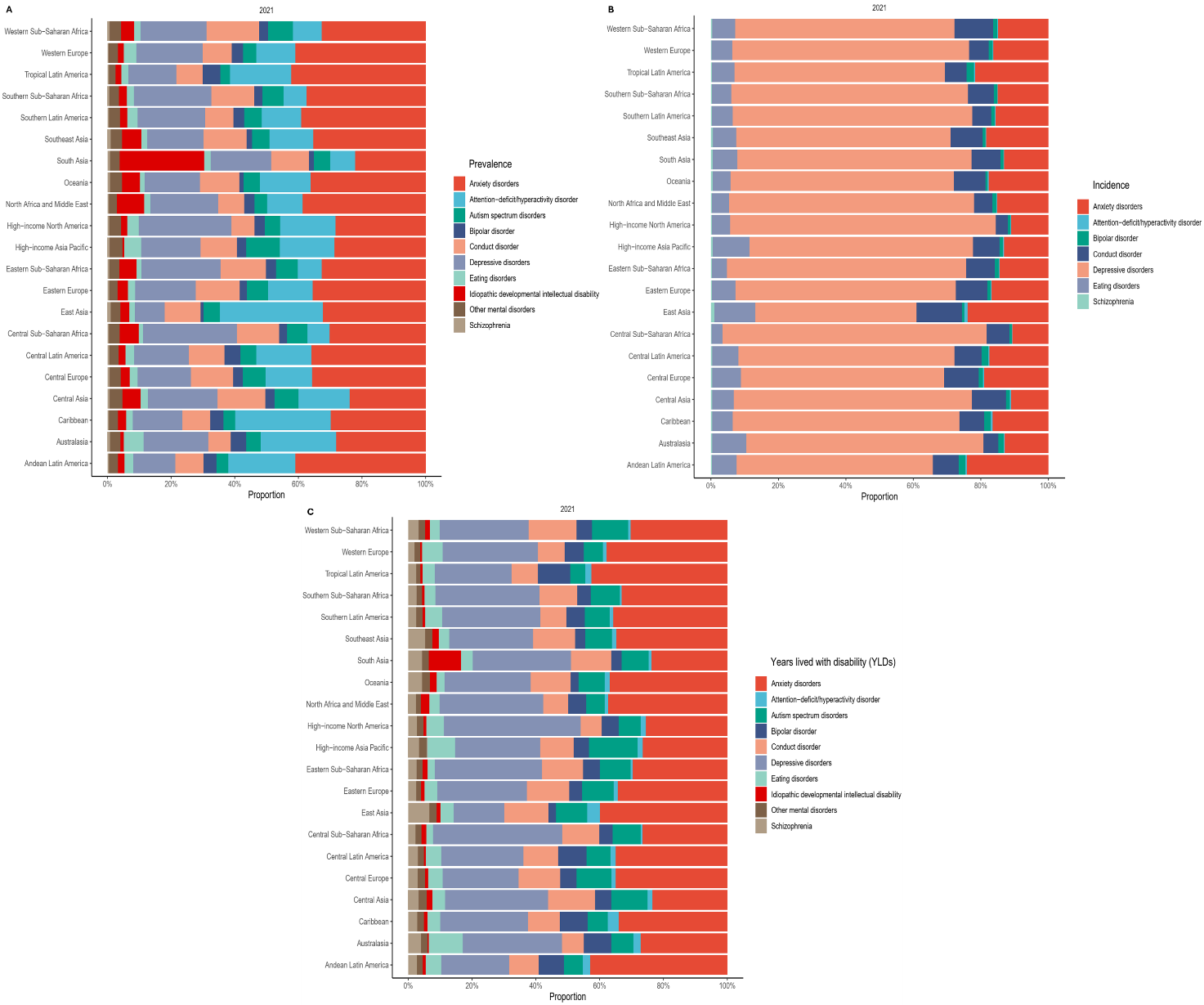


**Figure S11**. Prevalence, incidence, and years lived with disability (YLDs) numbers of mental disorders among individuals aged 10-24 years across 204 countries and territories in 2021, along with percentage changes from 2019 to 2021. (A) Prevalence number; (B) Percentage change in prevalence number; (C) Incidence number; (D) Percentage change in incidence number; (E) YLDs number; (F) Percentage change in YLDs number.


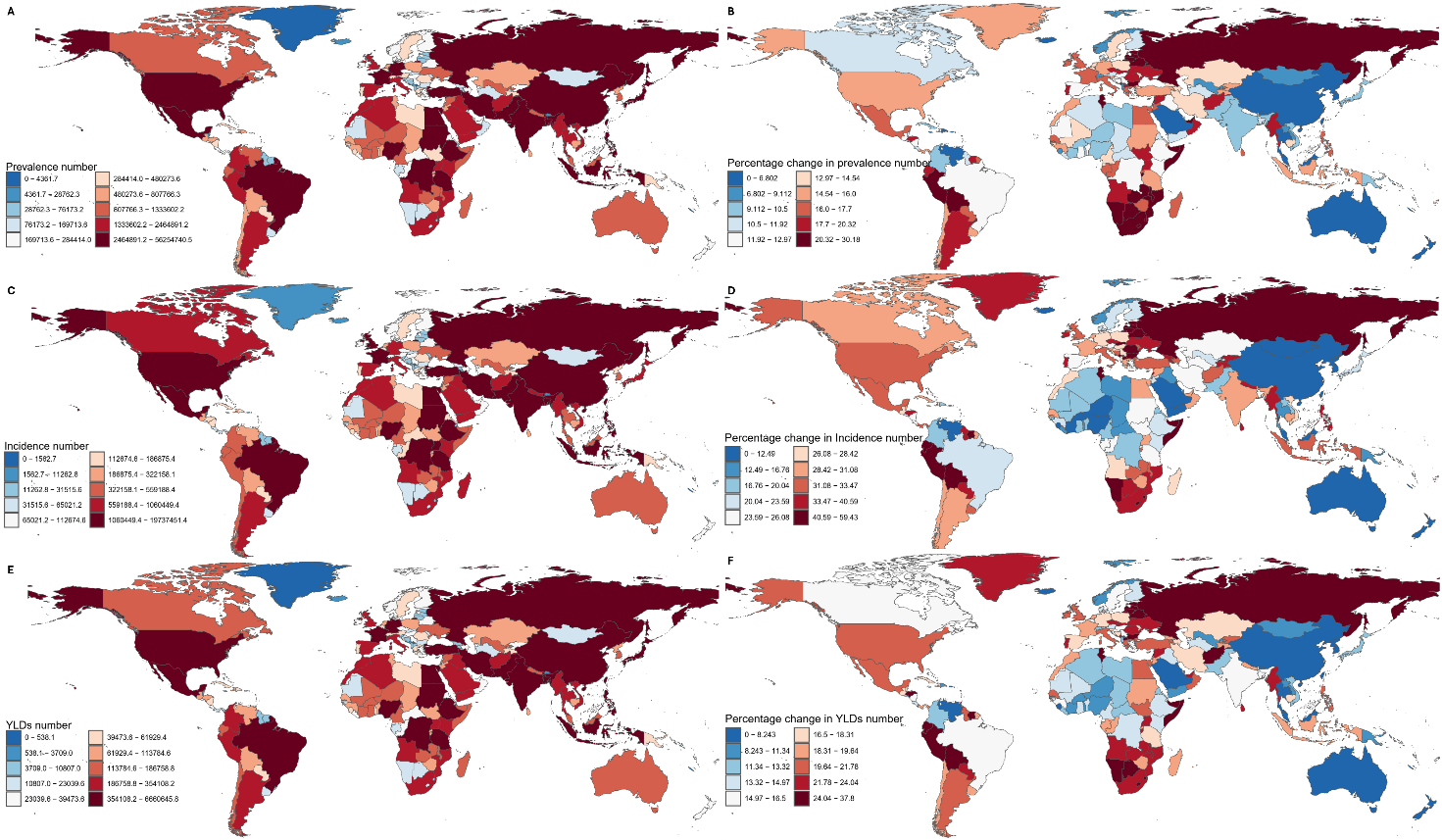


**Figure S12**. Global incidence of mental disorders by sex and age group (10-24 years) in 2021.


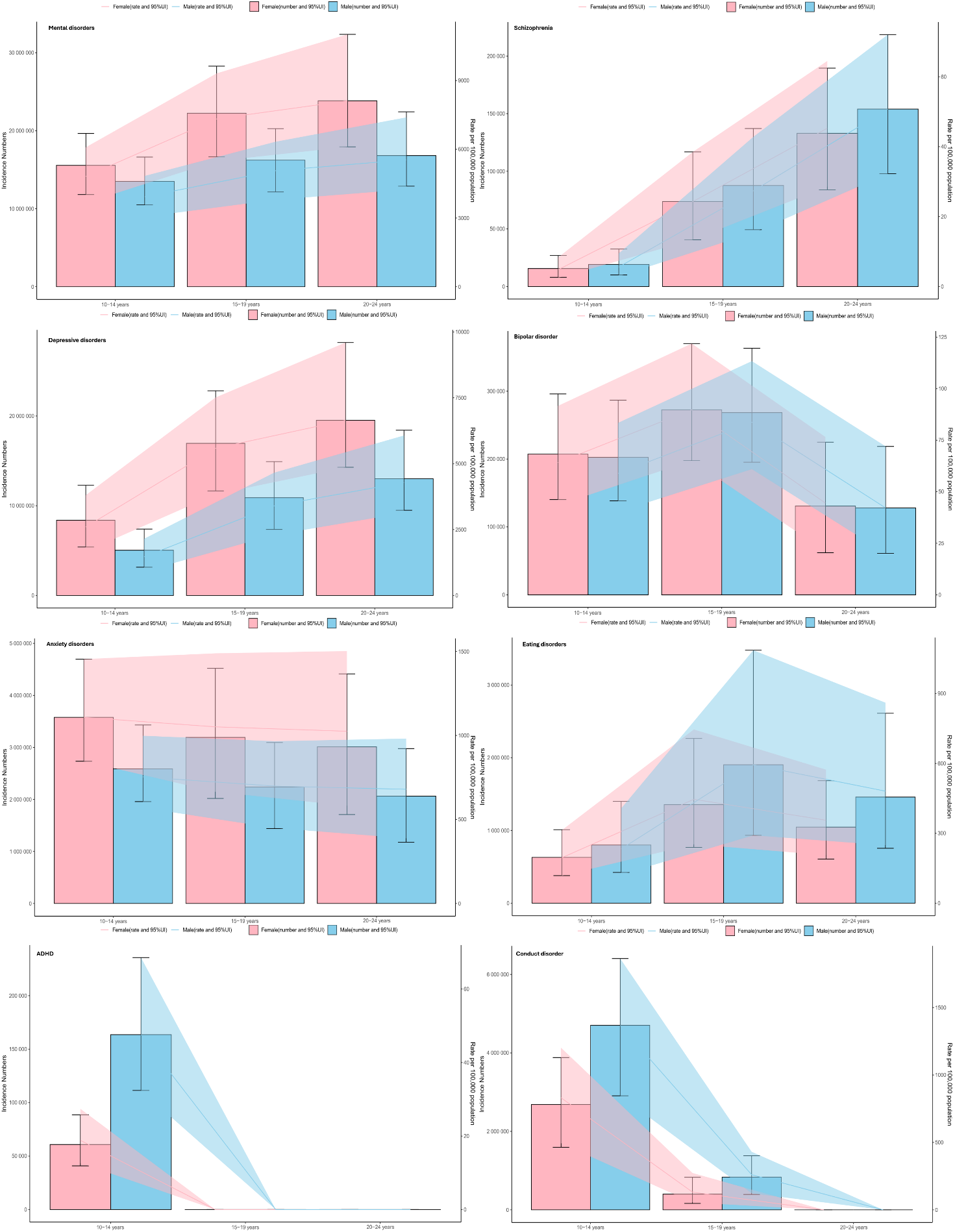


Lines indicate prevalent case with 95% uncertainty intervals for men and women.

**Figure S13**. Global years lived with disability (YLDs) of mental disorders by sex and age group (10-24 years) in 2021.


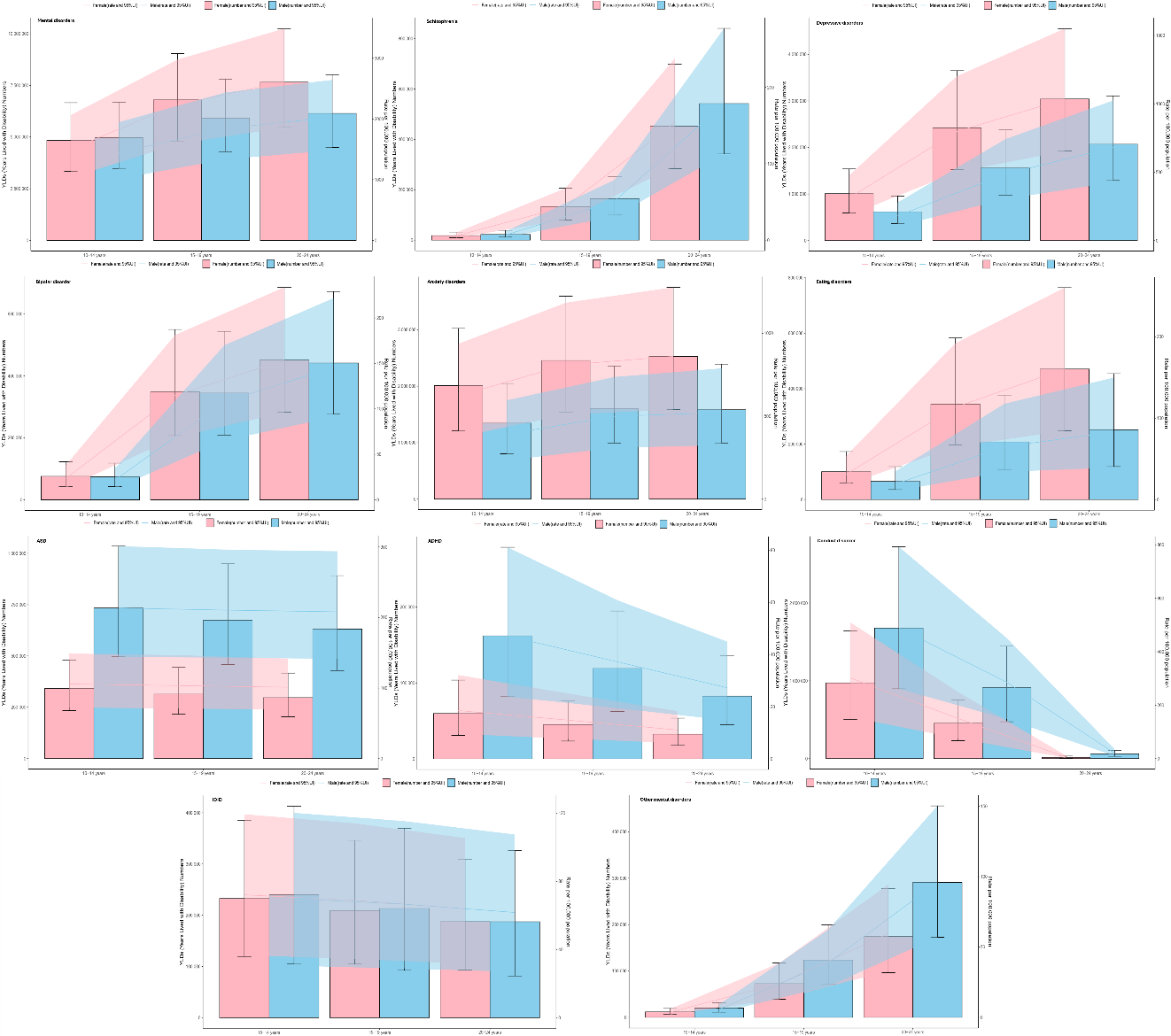


Lines indicate prevalent case with 95% uncertainty intervals for men and women.

**Figure S14**. Temporal trends in overall mental disorders across different age groups (10-24 years) from 1990 to 2021. (A) Prevalence numbers; (B) Prevalence rates; (C) Incidence numbers; (D) Incidence rates; (E) YLDs numbers; (F) YLDs rates.


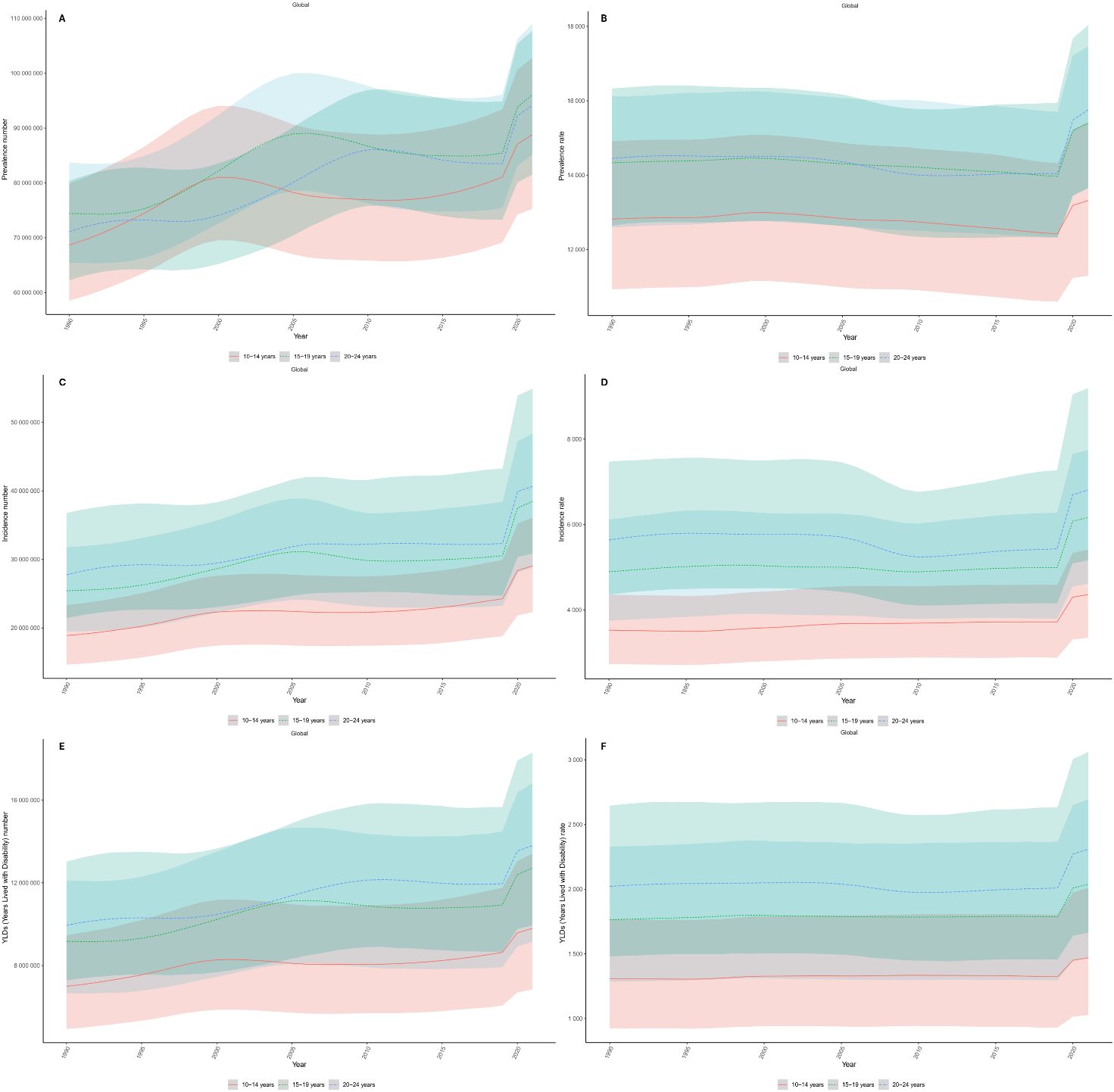


**Figure S15**. Sex differences in overall mental disorders among individuals aged 10-24 years across 21 regions in 2021. (A) Prevalence numbers; (B) Prevalence age-standardized rates; (C) Incidence numbers; (D) Incidence age-standardized rates; (E) years lived with disability (YLDs) numbers; (F) YLDs age-standardized rates.


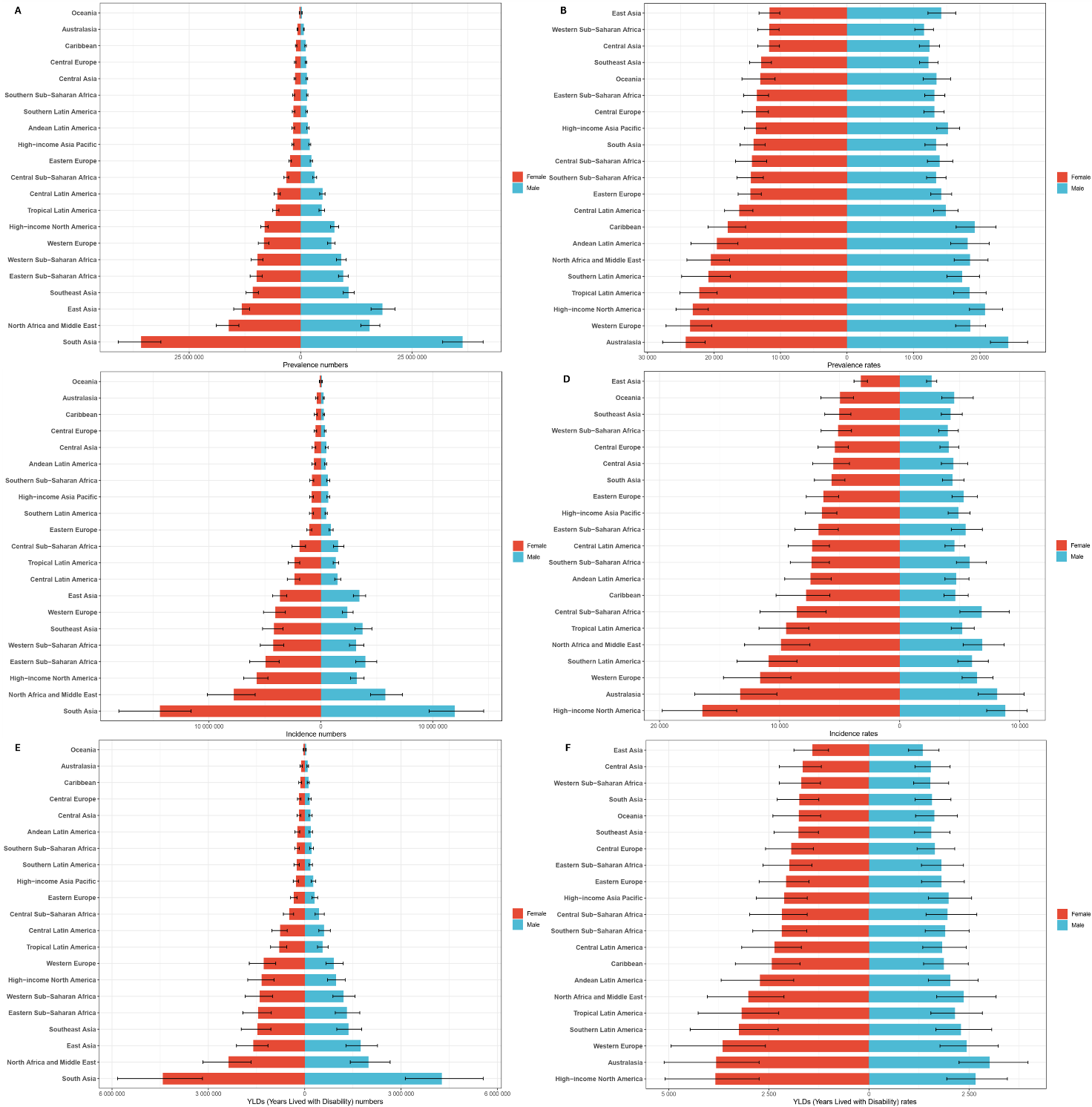


Lines indicate prevalent case with 95% uncertainty intervals for men and women.

**Figure S16**. Joinpoint regression analysis in the age-standardized prevalence rate of mental disorders among individuals aged 10-24 (1990-2021). (A) Schizophrenia; (B) Depressive disorders; (C) Bipolar disorder; (D) Anxiety disorders; (E) Eating disorders; (F) Autism spectrum disorders (ASD); (G) Attention-deficit/hyperactivity disorder (ADHD); (H) Conduct disorder; (I) Idiopathic developmental intellectual disability (IDID); (J) Other mental disorders (OMD).


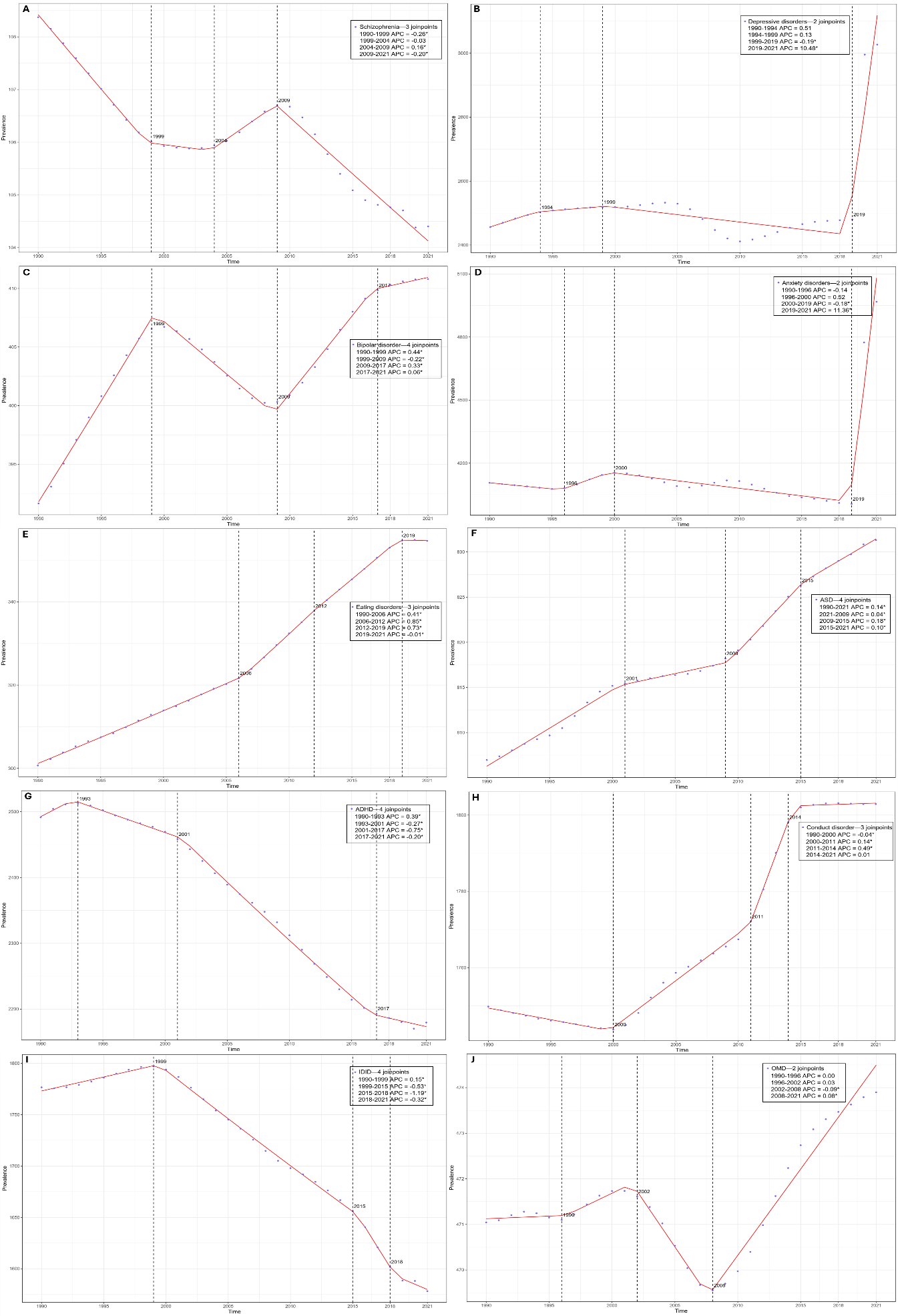


**Figure S17**. Joinpoint regression analysis in the age-standardized incidence rate of mental disorders among individuals aged 10-24 (1990-2021). (A) Schizophrenia; (B) Depressive disorders; (C) Bipolar disorder; (D) Anxiety disorders; (E) Eating disorders; (F) Attention-deficit/hyperactivity disorder (ADHD); (G) Conduct disorder.


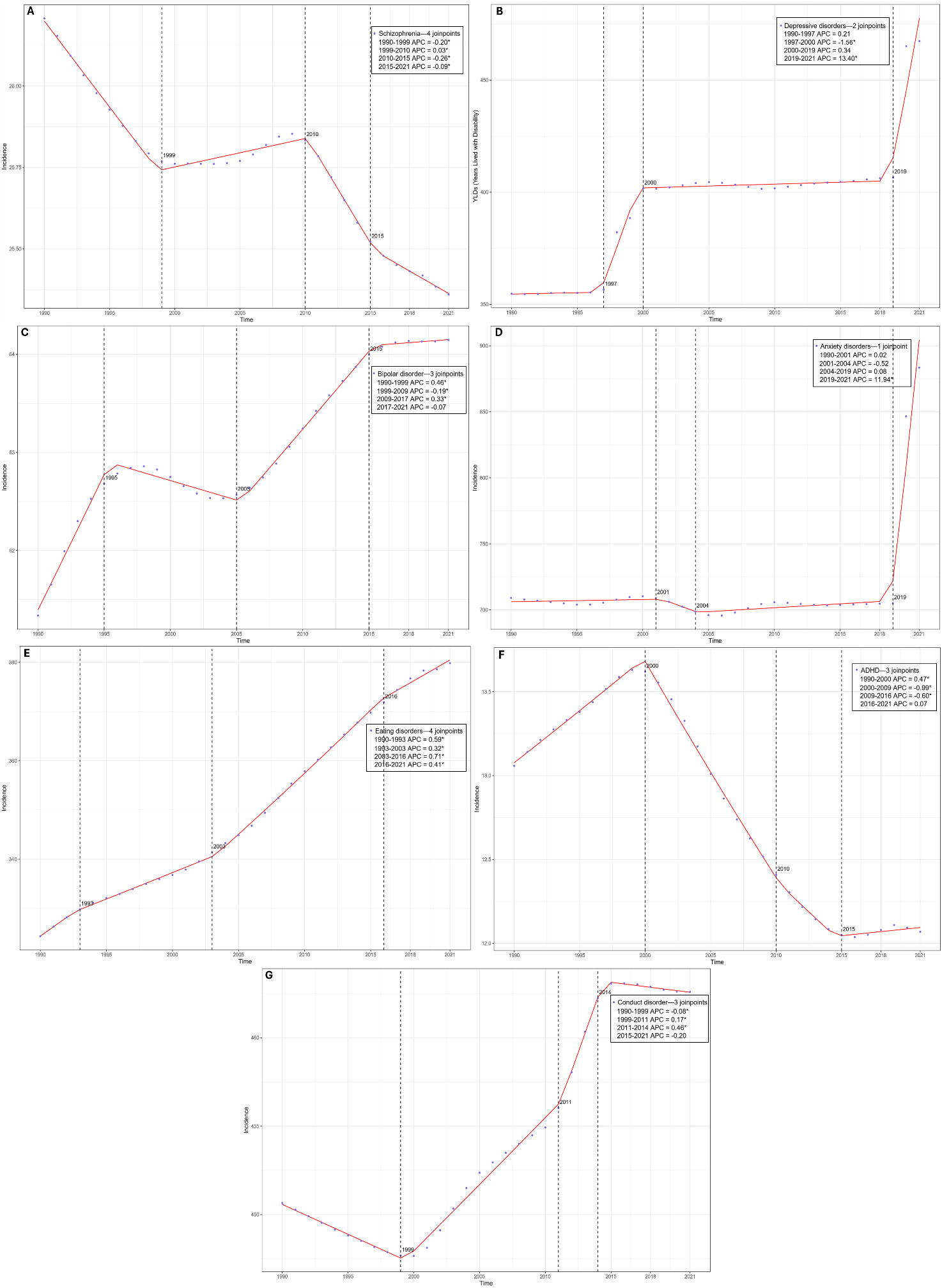

Supplement: Supplementary file 1 — Supplemental material [file 41398_2025_3623_MOESM1_ESM.docx]
